# Cobalt-catalyzed deoxygenative triborylation of allylic ethers to access 1,1,3-triborylalkanes

**DOI:** 10.1038/s41467-020-19039-7

**Published:** 2020-10-15

**Authors:** Wei Jie Teo, Xiaoxu Yang, Yeng Yeng Poon, Shaozhong Ge

**Affiliations:** Department of Chemistry, National University of Singapore, 3 Science Drive 3, 117543 Singapore, Singapore

**Keywords:** Homogeneous catalysis, Synthetic chemistry methodology, Reaction mechanisms

## Abstract

Polyborylated organic compounds have been emerging as versatile building blocks in chemical synthesis. Here we report a selective cobalt-catalyzed deoxygenative 1,1,3-triborylation reaction of allylic ethers with pinacolborane to prepare 1,1,3-triborylalkane compounds. With naturally abundant and/or synthetic cinnamic methyl ethers as starting materials, we have achieved the synthesis of a variety of 1,1,3-triborylalkanes (25 examples). The synthetic utility of these 1,1,3-triborylalkanes is demonstrated through site-selective allylation, protodeborylation, and consecutive carbon-carbon bond-forming reactions. Mechanistic studies including deuterium-labeling and control experiments suggest that this 1,1,3-triborylation reaction proceeds through initial cobalt-catalyzed deoxygenative borylation of allylic ethers to form allylic boronates followed by cobalt-catalyzed 1,1-diborylation of the resulting allylic boronates.

## Abstract (web summary)

Organoboron compounds are versatile intermediates in chemical synthesis. Here, the authors report a selective cobalt-catalyzed deoxygenative triborylation of allylic ethers with pinacolborane to prepare 1,1,3-triborylalkane compounds.

## Introduction

**Fig. 1:**
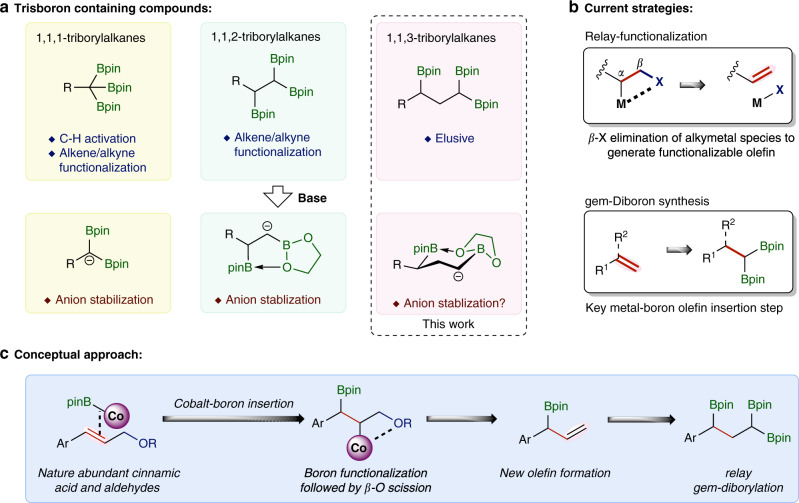
Introduction and the proposed synthesis of triborylalkane compounds. **a** Triborylalkane compounds. **b** Current strategies of relay functionalization and *gem*-diborylation. **c** Conceptual design of cobalt-catalyzed deoxygenative 1,1,3-triborylation.

Organoboron compounds are important reactive intermediates in chemical synthesis because of their high stability, low toxicity, and versatile reactivity in numerous carbon–carbon or carbon–heteroatom bond-forming reactions^1^. Compared with monoboronate compounds that are widely employed in Suzuki-Miyaura cross-coupling reactions^2^, the synthesis and synthetic potential of polyboron compounds have remained much less explored. *gem*-Diborylalkanes have recently been emerging as useful reagents for various C–C bond-forming reactions, which in turn has stimulated various methods for their synthesis^3–5^. As another important family of polyboron compounds, triborylalkanes have yet to receive equal attention in organic synthesis principally because straightforward synthetic approaches to prepare them are limited. For example, 1,1,1-triborylalkanes, prepared by the borylation of activated C–H bonds^6^ including the benzylic^7,8^ and homobenzylic^9^ C–H bonds or the borylation/hydroboration of alkenes^10–12^ and alkynes^13,14^, can form stabilized *gem*-diboryl carbanions through base-induced deborylation under milder basic conditions (Fig. 1a)^8^. These 1,1,1-triborylalkanes can undergo deborylative Boron–Wittig olefination, alkylation, and conjugated addition reactions^8,11,15^. 1,1,2-Triborylalkanes, typically synthesized by multi-borylation of alkenes^16,17^, alkynes^18^ and carbonyl groups^19^, can generate α-boryl carbanions stabilized by five-membered internal chelation through base-induced deborylation of an adjacent boryl group in the *gem*-diborylalkane unit (Fig. 1a)^20^, and can subsequently undergo diastereoselective alkylation reactions^16^. In this regard, we consider that 1,1,3-triborylalkanes can also generate α-boryl carbanions stabilized by six-membered internal chelation (Fig. 1a) and can have similar or even greater synthetic potential as they may undergo sequential C–C bond-forming reactions to allow the installation of three new C–C bonds. However, general approaches to synthesize such 1,1,3-triborylalkanes are lacking and their synthetic utility has remained to be explored.

*β*-*X* (*X* = heteroatoms) elimination from alkylmetal species is involved in numerous transition metal-catalyzed organic transformations^21–27^ and this classic elementary reaction of organometallic species can produce functionalized alkenes (Fig. 1b), which may undergo further transformations in a tandem manner^17,28–31^. For example, copper-catalyzed allylic borylation of allylic electrophiles is a well-developed method to prepare allylic boronates and mechanistic studies suggest that these allylic borylation reactions proceed with copper-boron intermediates as boron nucleophiles^32^. However, boryl-containing alkenes generated in these allylic borylation reactions do not undergo further borylation to form polyboron compounds under the conditions identified for copper-catalyzed allylic borylation reactions. Recently, catalytic 1,1-diborylation of alkenes has been developed into a straightforward approach to access synthetically versatile *gem*-diborylalkanes with nickel, cobalt, or zirconium catalysts^33–36^. These metal-catalyzed 1,1-diborylation reactions occurs through metal-boryl intermediates and migratory insertion of alkenes into such metal-boryl species is the key step to achieve this diborylation transformation. To develop a general approach to access 1,1,3-triborylalkane compounds, we envisioned that metal-catalyzed 1,1-diborylation of branched allylic boronates that are generated in situ via catalytic allylic borylation of allylic electrophiles could afford 1,1,3-triborylalkane products, provided that one transition metal compound can effectively catalyze both 1,1-diborylation and allylic borylation reactions under identical reaction conditions.

Allylic alcohol derivatives, such as allyl carbonates, acetates, and phosphates, have been extensively employed as electrophiles for catalytic carbon–carbon and carbon–heteroatom bond-forming reactions^37^. Compared with these commonly used allylic substrates, allyl ethers are considered less reactive and have been explored much less for catalytic organic reactions whereas they are readily accessible, cheap, and easy to handle. Recently, we have reported cobalt-catalyzed 1,1-diborylation of terminal alkenes in the presence of a hydrogen acceptor, and mechanistic studies suggest the competence of a Co-Bpin intermediate^33^. Conceptually, applying this cobalt-boron chemistry to functionalize allylic ethers, which can be derived from naturally abundant cinnamic acid and aldehyde compounds, here we developed a cobalt-catalyzed deoxygenative triborylation reaction of allylic ethers to prepare 1,1,3-triborylalkanes (Fig. 1c). Migratory insertion of allylic ether into a Co-Bpin species forms an alkylcobalt species containing a *β*-alkoxy group, and this alkylcobalt species can undergo facile *β*-alkoxy elimination to produce a branched allyl boronate due to the relatively high oxophilicity of cobalt^38,39^. Subsequent 1,1-diborylation of the resulting allyl boronate in the presence of the same cobalt catalyst can afford the desired 1,1,3-triborylalkane product.

## Results

### Evaluation of reaction conditions

To initiate the study on this cobalt-catalyzed deoxygenative triborylation reaction, we chose the reaction of allylic compounds (**2a**–**5**), derived from naturally abundant cinnamyl alcohol (**1**), with pinacolborane (HBpin) to identify reaction conditions that favor the formation of 1,1,3-triborylalkane product. The cobalt catalyst employed in this study was generated from bench-stable Co(acac)_3_ and xantphos and activated in situ by the reaction with HBpin. These experiments were conducted with allylic substrates as limiting reagents with six equivalents of HBpin in the presence of 5 mol% cobalt catalyst and five equivalents of norbonene (nbe) as a hydrogen acceptor in cyclohexane at 100 °C. The results of selected examples of these experiments are summarized in Table 1. In general, this reaction produces several organoboron compounds, such as monoborylalkane **6**, 1,3-diborylalkane **7a**, 1,1-diborylalkane **7b**, 1,1,3-triborylalkane **8a**, and 1,1,2-triborylalkane **8a**′.

**Table 1:** Evaluation of conditions for the cobalt-catalyzed triborylation^a^.


Entry	R	Concentration	Temperature (°C)	Conversion (%)	Ratio of 6:7a : 7b: 8a: 8a′	Yield of 8a (%)
1	−H (**1**)	0.03 M	100	>99	–:21:18:61:–	56
2	−CH_3_ (**2a**)	0.03 M	100	>99	9:5:3:80:3	70
3	−C(O)Ph (**3**)	0.03 M	100	<5	– : – : – : – : –	–
4	−P(O)(OEt)_2_ (**4**)	0.03 M	100	<5	– : – : – : – : –	–
5	−Si(^*t*^Bu)Me_2_ (**5**)	0.03 M	100	>99	14 : 6 : 5 : 71 : 4	53
6	−CH_3_ (**2a**)	0.03 M	rt	<5	– : – : – : – : –	–
7	−CH_3_ (**2a**)	0.03 M	50	<5	– : – : – : – : –	–
8	−CH_3_ (**2a**)	0.03 M	80	>99	11 : 5 : 3 : 75 : 6	60
9	−CH_3_ (**2a**)	0.1 M	100	>99	11 : 11 : 5 : 67 : 7	64
10	−CH_3_ (**2a**)	1.0 M	100	>99	13 : 20 : 8 : 52 : 6	49
11^b^	−CH_3_ (**2a**)	0.03 M	100	>99	85 : 5 : 5 : 5 : –	–
12^c^	−CH_3_ (**2a**)	0.03 M	100	>99	11 : 5 : 4 : 75 : 5	61
13^d^	−CH_3_ (**2a**)	0.03 M	100	>99	28 : 18 : 10 : 44 : –	38
14^e^	−CH_3_ (**2a**)	0.03 M	100	>99	14 : 13 : 8 : 62 : 1	55

Reaction conditions:

^a^Co(acac)_3_ (15.0 μmol), xantphos (15.0 μmol), allylic substrates (0.300 mmol), HBpin (1.80 mmol), nbe (1.50 mmol), cyclohexane (10 mL), 100 °C, 2 h, conversion, product ratio, and GC yield were determined by GC analysis with 1,3,5-trimethoxybenzene as the internal standard.

^b^No nbe.

^c^nbe (0.900 mmol).

^d^HBpin (0.900 mmol).

^e^HBpin (1.350 mmol).

Cinnamyl alcohol **1** reacted to afford a mixture of diborylalkanes (**7a** and **7b**) and 1,1,3-triborylalkane (**8a**) as a major product (entry 1 in Table 1). Subsequently, we tested several cinnamyl alcohol derivatives (**2a**–**5**) containing various leaving groups for this deoxygenative triborylation reaction (entries 2–5 in Table 1). The reaction conducted with cinnamyl methyl ether **2a** occurred to full conversion and the desired product 1,1,3-triborylalkane (**8a**) was formed in 70% GC yield (entry 2 in Table 1). Benzoate **3** and phosphate **4** of cinnamyl alcohol did not react with HBpin under identified conditions (entries 3 and 4 in Table 1). Silyl ether **5** of cinnamyl alcohol also reacted to give 1,1,3-triborylalkane **8a** as the major product (entry 5 in Table 1). We also tested various temperatures for the reaction of methyl ether **2a** (entries 2 and **6**–**8** in Table 1) and found that the reaction at 100 °C produced **8a** with a high selectivity. In addition, we found that concentrations of **2a** also had noticeable influence on the selectivity of this deoxygenative triborylation and reactions run with more concentrated (0.1 M or 1.0 M) solutions of **2a** yielded the target product **8a** in lower yields (entries 9 and 10 in Table 1). Furthermore, this cobalt-catalyzed deoxygenative triborylation of cinnamyl methyl ether **2a** did not occur in the absence of norbornene, and the reaction afforded monoborylalkane **6** as a major product with a high selectivity (>85%), together with trace amounts (<5%) of triborylalkane products (entry 11 in Table 1). Monoborylalkane **6** did not undergo further reactions with HBpin to yield 1,1,3-triborylalkane **8a** in the presence of norbornene and the cobalt catalyst. Diminished selectivity and yields of **8a** were obtained when the reactions were conducted with fewer equivalents of norbornene (entry 12 in Table 1) or with fewer equivalents of HBpin (entries 13 and 14 in Table 1).

**Fig. 2:**
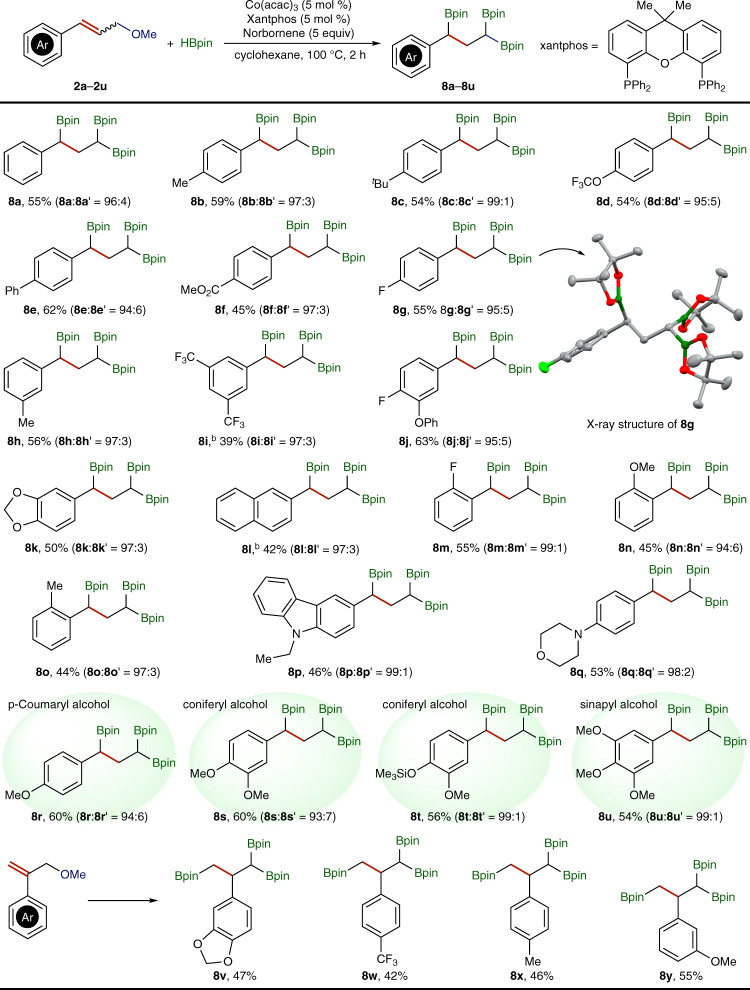
Substrate scope of allylic ethers for this cobalt-catalyzed triborylation reaction. Conditions: ^a^Co(acac)_3_ (15.0 μmol), xantphos (15.0 μmol), allylic methyl ether (0.300 mmol), HBpin (1.80 mmol), nbe (1.50 mmol), cyclohexane (10 mL), 100 °C, 2 h, isolated yields, regioselectivity (**8** **:** **8**′) in parentheses; ^b^Solvent cyclohexane/THF (1 : 1).

With the identified conditions (entry 2 in Table 1) for the synthesis of 1,1,3-triborylalkanes, we explored the scope of cinnamic methyl ethers for this cobalt-catalyzed deoxygenative triborylation, and the results are shown in Fig. 2. In general, a variety of aryl-substituted (*E*)-allyl or (*E*,*Z*)-allyl methyl ethers reacted with HBpin in the presence of norbornene to afford the corresponding 1,1,3-triborylalkanes (**8a**–**8u**) in moderate to good isolated yield and high regioselectivity, and the stereochemistry of allyl ether starting materials is inconsequential to the outcome of these reactions. The structure of triborylalkane **8g** was confirmed by single crystal X-ray analysis. 1,1,3-Triborylalkane products could be purified by column chromatography on silica, however, their instability on silica columns significantly lowered their isolated yields.

The data in Fig. 2 showed that both the steric and electronic properties of the aryl substituents in allyl ethers had significant influence on the isolated yields of 1,1,3-triborylalkane products. For example, steric bulkiness at the *para*-position of aryl groups (**8a**–**8c**) did not have a noticeable influence on the isolated yields, but increasing the steric hindrance at the *ortho*-position of aryl groups (**8n** and **8o**) decreased the isolated yields. In addition, the substrates containing electron-rich aryl groups (**8r**–**8u**) reacted to provide the desired products in higher isolated yields than the substrates containing electron-deficient aryl groups (**8i** and **8l**). More interestingly, allyl ethers derived from pulp waste lignin components, *p-*coumaryl (**8r**), coniferyl (**8s** and **8t**), and sinapyl (**8u**) monolignols also underwent this cobalt-catalyzed deoxygenative triborylation to produce the corresponding 1,1,3-triborylalkanes in good isolated yields. Aliphatic allylic methyl ethers reacted under identified conditions to give a complex mixture of mono-, di-, and triborylalkane products with the corresponding regioisomers. In addition, allyl methyl ethers with aryl substituents on the 2-position also underwent this cobalt-catalyzed reaction to afford desired 1,1,3-triborylalkane products (**8v**–**8y)**, albeit in modest isolated yields.

### Synthetic utility

**Fig. 3:**
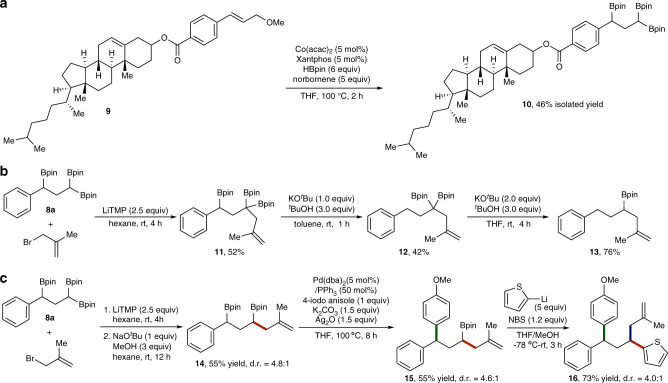
Synthetic applications of the deoxygenative triborylation method. **a** Triborylation of a cholesterol derived allylic ether. **b** Allylation of **8a** and sequential deborylation reactions. **c** Stepwise functionalization of 1,1,3-triborylalkane **8a** with sequential three new carbon-carbon bond-forming reactions.

To highlight the reliability of this deoxygenative triborylation, we tested this protocol with complex substrate **9**, a cholesterol derived allylic ether, and this reaction provided the desired 1,1,3-triborylalkane **10** in a modest yield (Fig. 3a). We also showed that triborylalkane **8a** underwent several further transformations. For example, allylation of **8a** with 3-bromo-2-methyl-prop-1-ene in the presence of a non-nucleophilic base LiTMP (TMP = tetramethylpiperidide) to afford a new 1,1,3-triborylalkane **11** in good isolated yield^40^, and compound **11** could undergo stepwise protodeborylation reactions to yield diborylalkane **12** and monoborylalkane **13** in synthetically useful yields (Fig. 3b). In addition, we also demonstrated that three new C–C bonds could be sequentially constructed by selective conversion of three C–B bonds of 1,1,3-triborylalkanes. For example, allylation of **8a** with 3-bromo-2-methyl-prop-1-ene followed by protodeborylation in the presence of NaO^*t*^Bu as a base and MeOH as a proton source afforded 1,3-diborylalkane **14** in 55% isolated yield (Fig. 3c). The relative stereochemistry of **14** was assigned anti by oxidizing **14** to a 1,3-diol and comparing the stereochemistry of the 1,3-diol with the reported data (see Supplementary Fig. 7 for a plausible explanation of this obtained diastereoselectivity)^41^. The benzylic boronate in **14** selectively underwent palladium-catalyzed cross-coupling with 4-iodoanisole to afford alkylboronate **15**, which could further react with 2-thienyllithium to produce compound **16** in high isolated yield (Fig. 3c)^42^.

### Mechanistic considerations

**Fig. 4:**
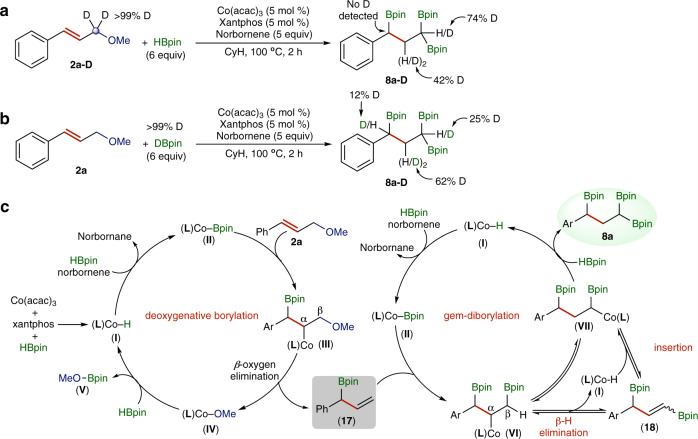
Mechanistic studies. **a** The reaction of **2a-D** with HBpin. **b** The reaction of **1a** with DBpin. **c** The proposed catalytic pathways for this cobalt-catalyzed deoxygenative triborylation reaction.

First, we conducted deuterium-labeling studies to get some preliminary understanding of this cobalt-catalyzed deoxygenative triborylation. To find out whether this transformation proceeds through *β-*O elimination pathway or allylic substitution via *π*-allyl metal species, we tested this deoxygenative triborylation reaction with **2a**-**D** under standard conditions (Fig. 4a). One would expect a scrambling of deuterium across the allyl carbons in presences of *π*-allyl metal intermediates^43,44^. The absence of deuterium on the benzylic carbon in **8a**-**D** suggests that the reaction proceed through a *β-*O elimination pathway instead of an allylic substitution pathway. We also conducted 1,1,3-triborylation of **1a** with DBpin in the presence of norbornene under standard conditions, and found that deuterium atoms were incorporated onto all three non-aromatic carbons in substrate **2a** (Fig. 4b). This observed deuterium incorporation can be explained by reversible insertion *β-*H elimination via a Co-H intermediate. In addition, gas chromatography-mass spectrometry analysis on the crude reaction mixture also revealed the incorporation of deuterium into norbornane molecules.

Based on the results of these deuterium-labeling experiments and the precedent of cobalt-catalyzed hydroboration or borylation reactions^7,10,11^, we proposed plausible catalytic pathways for this cobalt-catalyzed deoxygenative triborylation of allylic ethers. This reaction proceeds through two collaborative catalytic cycles, namely deoxygenative borylation cycle and *gem*-diborylation cycle (Fig. 4c). In the deoxygenative borylation cycle, activation of Co(acac)_3_ by HBpin in the presence of xantphos generates a cobalt hydride species (**L**)Co-H (**I**), which is subsequently converted to a cobalt-boryl species (**L**)Co-Bpin (**II**) in the presence of norbornene as a hydrogen acceptor. During this conversion, norbornene is hydrogenated to norbornane. Migratory insertion of cinnamyl methyl ether **2a** into (**L**)Co-Bpin (**II**) forms an alkylcobalt intermediate (**III**), which undergoes *β*-OMe elimination to form an allyl boronate **17** and a methoxycobalt species (**L**)Co-OMe (**IV**). This methoxycobalt intermediate then reacts with HBpin to regenerate the cobalt hydride (**L**)Co-H (**I**) and release a tris(alkoxy)borate MeOBpin (**V**), which can be detected by ^11^B NMR spectroscopic analysis with a resonance signal at 22.3 ppm. The *gem*-diborylation cycle also starts with the conversion of (**L**)Co-H (**I**) to (**L**)Co-Bpin (**II**) in the presence of norbornene as a hydrogen acceptor. Allyl boronate **17** undergoes migratory insertion into (**L**)Co-Bpin (**II**) generates an alkylcobalt species **VI**, which then isomerizes to a new alkylcobalt species **VII** via *β*-H elimination followed by reversible migratory insertion. The alkylcobalt intermediate **VII** subsequently reacts with HBpin to release 1,1,3-triborylalkane **8a** and regenerate the catalytically active (**L**)Co-H (**I**). The alkylcobalt species **VII** contains an α-boryl alkyl group and is thermodynamically stable, which may be caused by the interaction of the *d*-electrons of Co(I) species with the empty *p*-orbital on boron. Similar boron-directed isomerization of metal alkyl species was observed previously for other metal-catalyzed chain-walking functionalization of boryl-containing alkenes^45–47^.

**Fig. 5:**
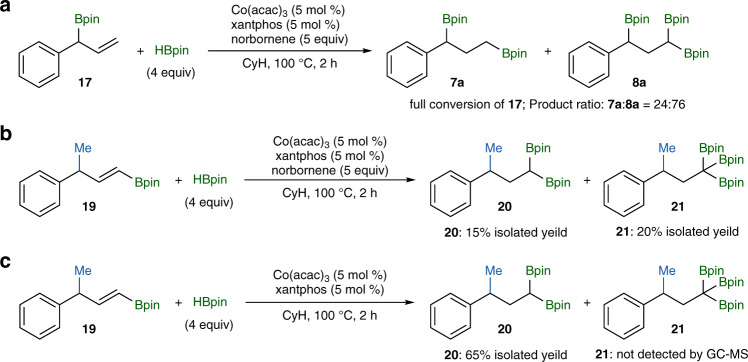
Control experiments to confirm possible reaction intermediates. **a** *gem*-diborylation reaction of **17** with HBpin under standard reaction conditions. **b** The reaction of **19** with HBpin in the presence of norbornene as a hydrogen acceptor. **c** The reaction of **19** with HBpin in the absence of norbornene.

As shown in Fig. 4c, allyl boronate **17** and vinyl boronate **18** are key intermediates for this cobalt-catalyzed deoxygenative borylation. To support their intermediacy, we then prepared allyl boronate **17** independently and subjected it to the standard reaction conditions, and found that compound **17** was indeed converted to the desired 1,1,3-triborylalkane product with a selectivity of 76% (Fig. 5a). We also attempted to prepare vinyl boronate **18**, but all the synthetic approaches we have employed did not lead to the formation of compound **18**. Alternatively, we synthesized vinyl boronate **19**, which has a structure similar to vinyl boronate **18**, and tested it for this cobalt-catalyzed transformation (Fig. 5b, c). The reaction of vinyl boronate **19** with excess of HBpin in the presence of norbornene as a hydrogen acceptor yielded *gem*-diborylalkane **20** and and 1,1,1-triborylalkane **21** with similar isolated yields (Fig. 5b). However, the corresponding reaction of **19** with HBpin in the absence of norbornene produced only *gem*-diborylalkane **20** in 65% isolated yield (Fig. 5c). The results of these two experiments suggest that the isomerization of alkylcobalt species **VI** to **VII**, as shown in Fig. 4c, likely proceeds through *β*-H elimination to form vinyl boronate **18** followed by re-insertion of **18** into cobalt hydride species (**L**)Co-H.

In summary, we have developed an effective protocol for the facile synthesis of 1,1,3-triborylalkanes by cobalt-catalyzed deoxygenative 1,1,3-triborylation of allylic methyl ethers with HBpin. A variety of allylic ethers reacted with HBpin in the presence of norbornene as a hydrogen acceptor to afford synthetically versatile, otherwise challenging to prepare, 1,1,3-triborylalkanes in modest to good isolated yields with high chemo- and regioselectivity. The cobalt catalyst for this deoxygenative triborylation reaction was generated in situ from readily available and bench-stable Co(acac)_3_ and xantphos and activated by the reaction with HBpin. Mechanistic studies suggest that this 1,1,3-triborylation reaction proceeds through initial cobalt-catalyzed deoxygenative borylation of allylic ethers to form allyl boronates followed by cobalt-catalyzed *gem*-diborylation of the resulting allyl boronates.

## Methods

### General procedure for dehydrogenative triborylation of allylic ethers

In an Argon-filled glovebox, a 20 mL screw-capped vial was charged with Co(acac)_3_ (5.3 mg, 15.0 µmol), xantphos (8.7 mg, 15.0 µmol), norbornene (141 mg, 1.50 mmol), allyl methyl ether (0.300 mmol), cyclohexane (10 mL), and a magnetic stirring bar. The solution was stirred and HBpin (261 µL, 1.80 mmol) was added to the vial. The vial was then sealed with a cap containing a PTFE (polytetrafluoroethylene) septum and removed from the glovebox. The mixture was allowed to react at 100 °C for 2 h. Subsequently, the solvent was removed under reduced pressure and the residue was purified by flash chromatography on silica (column I.D. 13.4 mm, eluent:  hexane/ethyl acetate) to afford the corresponding 1,1,3-triborylalkane products. See the Supplementary Information for detailed experimental procedures and the characterization data of all the products.

## Supplementary information

Supplementary Information

## Data Availability

The X-ray crystallographic data for compound **8g** reported in this study have been deposited at the Cambridge Crystallographic Data Centre (CCDC) under deposition number CCDC-2027345. These data can be obtained free of charge from CCDC via www.ccdc.cam.ac.uk/data_request/cif. The authors declare that all other data supporting the findings of this study are available within the article and Supplementary Information files, and also are available from the corresponding author upon reasonable request.
