# Supplementary material for: Cobalt-catalyzed deoxygenative triborylation of allylic ethers to access 1,1,3-triborylalkanes
Source: Nat Commun. 2020 Oct 15;11:5193. doi: 10.1038/s41467-020-19039-7 (PMC7562742; doi:10.1038/s41467-020-19039-7)
Supplement: Supplementary file 1 — Supplementary Information [file 41467_2020_19039_MOESM1_ESM.pdf]

Supplementary Information

Cobalt-Catalyzed Deoxygenative Triborylation of Allylic Ethers to Access 1,1,3-Triborylalkanes

Wei Jie Teo, Xiaoxu Yang, Yeng Yeng Poon and Shaozhong Ge

Supplementary Methods

General Remarks

All the manipulations were performed in an argon-filled glovebox, unless mentioned otherwise. THF, toluene, and hexane were purified by passing the degassed solvents (N_2) through a column of activated alumina (solvent purification system purchased from Innovative Technologies, Newburyport, MA). The following chemicals were purchased and used as received: Cobalt(III) acetylacetonate (99.99% trace metals basis, Sigma-Aldrich), pinacolborane (98%, Oakwood Chemical), norbornene (99%, Sigma-Aldrich), and Xantphos (97.0%, Oakwood Chemical). Cyclohexane (anhydrous, 99.5%, Sigma-Aldrich) was used as received. All other chemicals were used as received. The silica gel (300-400 mesh) used for isolation was purchased from Sanpont and used as received.

^{1}H and ^{13}C spectra were recorded using Bruker 400 MHz, or 500 MHz NMR spectrometers and done in $CDCl_3$ unless otherwise staed. ^{1}H NMR and ^{13}C NMR spectra were referenced to resonances of the residual signals of the deuterated solvents. Multiplicities are recorded as: s = singlet, d = doublet, t = triplet, dd = doublet of doublets, dt = doublet of triplets and m = multiplet. GC analysis was acquired on Agilent 6850 gas chromatograph equipped with a flame-ionization detector. GC-MS analysis was performed on Shimadzu GC-2010 gas chromatograph coupled to a Shimadzu QP2010 mass selective detector. HR-MS analyses were performed using Bruker micrOTOFQII (ESI).

(*E*)-Cinnamyl methyl ether (**2a**)[1], (*E*)-methyl *p*-methylcinnamyl ether (**2b**)[1] and [1-(methoxymethyl)ethenyl]benzene[2] were synthesized based on the literatures. Visualization of -trisboronate compounds on TLC is done by staining it with “Seebach’s staining solution” and applied with heat. The stained spots turned blue with a light yellow background.

General Screening

General Procedure for Screening Reactions of Development of Catalytic 1,1,3-Triboronates Synthesis

In an Ar-filled glovebox, a 20-mL screw-capped vial was charged with cobalt(III) acetylacetonate (5.3 mg, 15.0 μmol), xantphos (8.7 mg, 15.0 μmol), norbornene, **2a** (44.4 mg, 0.300 mmol), 1,3,5-trimethoxybenzene (16.8 mg, 0.100 mmol), cyclohexane and a magnetic stirring bar. The solution was stirred and pincolborane was charged in the vial and sealed with a cap containing a PTFE septum. The vial was removed from the glovebox and stirred at the stated temperature for 2 h, after which, GC analysis was done.

Identification of side products 6, 7a, 7b and 8a'

6 was synthesized and isolated using condition below:

In an Ar-filled glovebox, a 20-mL screw-capped vial was charged with cobalt(III) acetylacetonate (5.3 mg, 15.0 μmol), xantphos (8.7 mg, 15.0 μmol), **2a** (44.4 mg, 0.300 mmol), cyclohexane (10 mL) and a magnetic stirring bar. The solution was stirred and pincolborane (261.2 μL, 1.800 mmol) was charged in the vial and sealed with a cap containing a PTFE septum. The vial was removed from the glovebox and stirred at 100 ˚c for 2 h, after which, the solvent was removed under reduce pressure. The crude product was purified and isolated as a colourless oil using silica gel flash column chromatography with hexane/EA (100:1) as eluent.

2-(3-methoxy-1-phenylpropyl)-4,4,5,5-tetramethyl-1,3,2-dioxaborolane (6, R = Me)

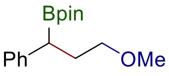

6

^{1}H NMR (500 MHz, CDCl$_3$) δ 7.26 – 7.19 (m, 4H), 7.16 – 7.10 (m, 1H), 3.42 – 3.35 (m, 1H), 3.33 – 3.27 (m, 4H), 2.41 (t, *J* = 7.9 Hz, 1H), 2.22 – 2.12 (m, 1H), 1.93 – 1.80 (m, 1H), 1.19 (s, 6H), 1.18 (s, 6H). ^{13}C NMR (126 MHz, CDCl$_3$) δ 143.0, 128.5, 128.4, 125.4, 83.4, 72.1, 58.6, 32.5, 24.7, 24.7. (the resonance of the carbon with boron attached was not observed). GC-MS (EI) m/z: calcd for $C_{16}H_{25}BO_3$ ([M]$^+$): 276.20; Found 276.20.

7a was synthesized and isolated using general procedure.

2,2'-(1-phenylpropane-1,3-diyl)bis(4,4,5,5-tetramethyl-1,3,2-dioxaborolane) (7a)

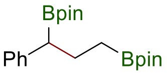

7a

^{1}H NMR (500 MHz, CDCl$_3$) δ 7.26 – 7.16 (m, 4H), 7.10 (t, *J* = 7.0 Hz, 1H), 2.25 (t, *J* = 8.0 Hz, 1H), 1.99 – 1.88 (m, 1H), 1.80 – 1.69 (m, 1H), 1.22 (s, 12H), 1.20 (s, 6H), 1.18 (s, 6H), 0.80 – 0.72 (m, 2H). ^{13}C NMR (126 MHz, CDCl$_3$) δ 140.4, 128.8, 128.3, 125.2, 83.3, 83.0, 27.2, 25.0, 24.8, 24.8. (the resonance of the carbon with boron attached was not observed). GC-MS (EI) m/z: calcd for $C_{16}H_{25}BO_3$ ([M]$^+$): 372.26; Found 372.25.

7b[3] and **8a'** [4] were synthesized and isolated for GC analysis of the retention time.

Crude mixture of **7a**+**7b** were isolated for NMR analysis shown below:

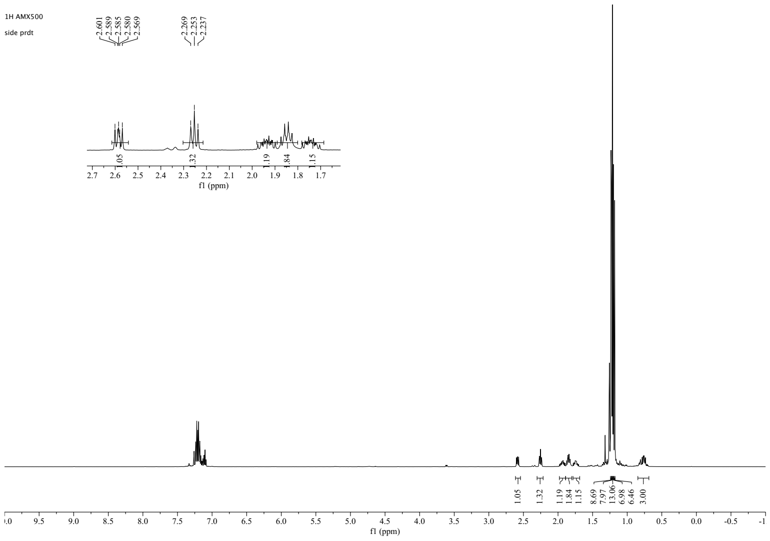

Supplementary Figure 1. 1H NMR of the mixture of **7a** and **7b**.

Substrate Synthesis

Cinnamic methyl ether substrates were prepared from modified conditions of the literature.[5]

(*E*)-Cinnamalcohol (**1**) was commercial available.

(*E*)-Cinnamyl benzoate[6] (**3**), (*E*)-cinnamyl diethyl phosphate[7] (**4**) and (*E*)-1-*tert*-butyldimethylsilyloxy-3-phenyl-2-propene[8] (**5**) were synthesized according to literatures.

General Procedure for the Preparartion of (*E*)-Cinnamic Methyl Ether

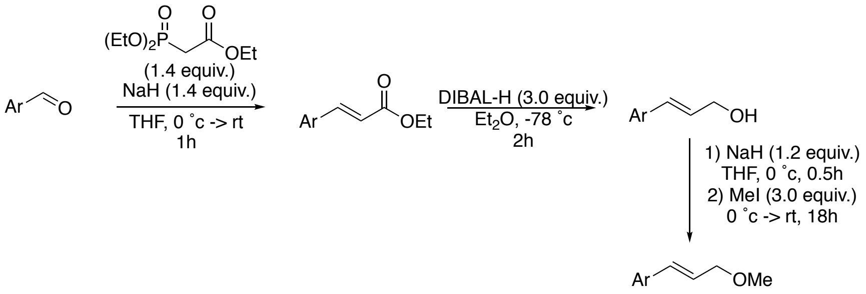

Supplementary Equation 1. Preparartion of (*E*)-Cinnamic Methyl Ether.

To a solution of triethyl phosphonoacetate (2.27 mL, 14.0 mmol) in THF (50 mL), NaH (60% dispersion in mineral oil, 0.56 g, 14.0 mmol) was added portion-wise at 0 ˚c. After 30 minutes, benzaldehyde (10 mmol) was added dropwise at 0 ˚c and stirred at room temperature for 1h. The reaction was quenched with saturated aq. NH_4Cl solution and extracted with diethyl ether (3 x 50 mL). The organic phases were combined and washed with brine and dried over Na_2SO_4, after which, the solvent was removed under reduce pressure. The crude product was purified and isolated as a colourless oil using silica gel flash column chromatography with hexane/EA as eluent. The purified acrylate was diluted in Et_2O (100 mL) and DIBAL-H (1.0 M in toluene, 30 mL, 30 mmol) was added dropwise at -78 ˚c and stirred for 2h. The reaction was quenched with a saturated aq. solution of Rochelle salt and stirred for 1 h. The aqueous phase was extracted with diethyl ether (3 x 50 mL) and the organic phases were combined and washed with brine and dried over Na_2SO_4, after which, the solvent was removed under reduce pressure. The crude allylic alcohol was engaged in the next step without further purification, dissolving in THF (50 mL) and cooled to 0 ˚c before NaH (60% dispersion in mineral oil, 0.48 g, 12.0 mmol) was added portion-wise. Methyl iodide (1.90 mL, 30 mmol) was added dropwise at 0 ˚c and stirred at room temperature for 18h. The reaction was quenched with saturated aq. NH_4Cl solution and extracted with diethyl ether (3 x 50 mL). The organic phases were combined and washed with brine and dried over Na_2SO_4, after which, the solvent was removed under reduce pressure. The crude product was purified and isolated as a colourless oil using silica gel flash column chromatography with hexane/EA as eluent to give (*E*)-cinnamic methyl ethers.

(*E*)-4-(3-methoxyprop-1-en-1-yl)-1,1'-biphenyl (2e)

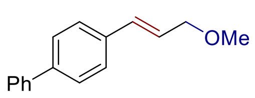

2e

^{1}H NMR (500 MHz, $CDCl_3$) δ 7.64 – 7.55 (m, 4H), 7.51 – 7.41 (m, 4H), 7.38 – 7.32 (m, 1H), 6.66 (d, *J* = 16.0 Hz, 1H), 6.34 (dt, *J* = 16.0, 6.0 Hz, 1H), 4.13 (dd, *J* = 6.0, 1.2 Hz, 2H), 3.42 (s, 3H). ^{13}C NMR (126 MHz, $CDCl_3$) δ 140.8, 140.6, 135.9,

132.1, 128.9, 127.5, 127.4, 127.1, 127.0, 126.2, 73.3, 58.2. GC-MS (EI) m/z: calcd for $C_{11}H_{14}O_2$ ($[M]^+$): 178.10; Found 178.10.

(*E*)-1-(3-methoxyprop-1-en-1-yl)-3,5-bis(trifluoromethyl)benzene (2i)

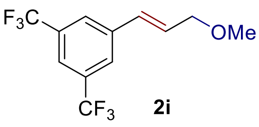

2i

^1H NMR (500 MHz, $CDCl_3$) δ 7.82 (s, 2H), 7.76 (s, 1H), 6.72 (dt, *J* = 15.9, 1.7 Hz, 1H), 6.47 (dt, *J* = 16.0, 5.4 Hz, 1H), 4.16 (dd, *J* = 5.4, 1.7 Hz, 2H), 3.45 (s, 3H). ^{13}C NMR (126 MHz, $CDCl_3$) δ 138.9, 131.9 (q, *J* = 33.3 Hz), 130.5, 128.7, 126.2 (q, *J* = 4.6 Hz), 123.3 (q, *J* = 272.6 Hz), 121.0 (q, *J* = 4.1 Hz), 72.3, 58.4. GC-MS (EI) m/z: calcd for $C_{12}H_{10}F_6O$ ($[M]^+$): 284.10; Found 284.05.

(*E*)-2-(3-methoxyprop-1-en-1-yl)naphthalene (2l)

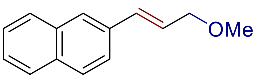

2l

^1H NMR (500 MHz, $CDCl_3$) δ 7.86 – 7.79 (m, 3H), 7.77 (s, 1H), 7.66 – 7.62 (m, 1H), 7.52 – 7.44 (m, 2H), 6.80 (d, *J* = 15.9 Hz, 1H), 6.44 (dt, *J* = 15.9, 6.0 Hz, 1H), 4.17 (dd, *J* = 6.0, 1.2 Hz, 2H), 3.45 (s, 3H). ^{13}C NMR (126 MHz, $CDCl_3$) δ 134.3, 133.7, 133.1, 132.6, 128.3, 128.1, 127.7, 126.6, 126.4, 126.3, 126.0, 123.7, 73.2, 58.1. GC-MS (EI) m/z: calcd for $C_{14}H_{14}O$ ($[M]^+$): 198.10; Found 198.10.

(*E*)-1-methoxy-2-(3-methoxyprop-1-en-1-yl)benzene (2n)

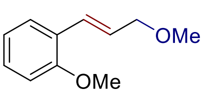

2n

^1H NMR (500 MHz, $CDCl_3$) δ 7.48 – 7.42 (m, 1H), 7.26 – 7.20 (m, 1H), 6.98 – 6.90 (m, 2H), 6.87 (d, *J* = 8.3 Hz, 1H), 6.30 (dt, *J* = 16.1, 6.2 Hz, 1H), 4.11 (d, *J* = 6.2 Hz, 2H), 3.85 (s, 3H), 3.39 (s, 3H). ^{13}C NMR (126 MHz, $CDCl_3$) δ 156.9, 128.9, 127.7, 127.2, 126.7, 120.8, 111.0, 73.8, 58.0, 55.6. GC-MS (EI) m/z: calcd for $C_{11}H_{14}O_2$ ($[M]^+$): 178.10; Found 178.10.

(*E*)-1-methoxy-4-(3-methoxyprop-1-en-1-yl)benzene (2r)

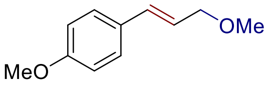

2r

^1H NMR (500 MHz, $CDCl_3$) δ 7.33 (d, *J* = 8.8 Hz, 2H), 6.85 (d, *J* = 8.6 Hz, 2H), 6.55 (d, *J* = 15.9 Hz, 1H), 6.15 (dt, *J* = 15.8, 6.2 Hz, 1H), 4.07 (dd, *J* = 6.2, 1.4 Hz, 2H), 3.81 (s, 3H), 3.38 (s, 3H). ^{13}C NMR (126 MHz, $CDCl_3$) δ 159.4, 132.4, 129.6, 127.8, 123.8, 114.1, 73.4, 58.0, 55.4. GC-MS (EI) m/z: calcd for $C_{11}H_{14}O_2$ ($[M]^+$): 178.10; Found 178.10.

(*E*)-*tert*-butyl(2-methoxy-4-(3-methoxyprop-1-en-1-yl)phenoxy)dimethylsilane (2t)

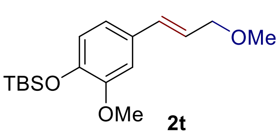

2t

^1H NMR (400 MHz, $CDCl_3$) δ 6.91 (d, *J* = 2.0 Hz, 1H), 6.86 – 6.82 (m, 1H), 6.80 – 6.77 (m, 1H), 6.53 (dt, *J* = 15.9, 1.4 Hz, 1H), 6.14 (dt, *J* = 15.9, 6.2 Hz, 1H), 4.07 (dd, *J* = 6.3, 1.4 Hz, 2H), 3.81 (s, 3H), 3.38 (s, 3H), 0.99 (s, 9H), 0.15 (s, 6H). ^{13}C NMR (101 MHz, $CDCl_3$) δ 151.0, 145.1, 132.7, 130.7, 123.9, 120.9, 119.7, 109.9, 73.2, 57.9, 55.4, 25.7, 18.5, -4.6. GC-MS (EI) m/z: calcd for $C_{17}H_{28}O_3Si$ ($[M]^+$): 308.20; Found 308.20.

(*E*)-1,2,3-trimethoxy-5-(3-methoxyprop-1-en-1-yl)benzene (2u)

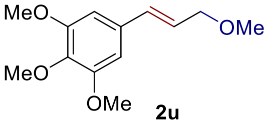

2u

^1H NMR (400 MHz, $CDCl_3$) δ 6.60 (s, 2H), 6.51 (d, *J* = 15.8 Hz, 1H), 6.18 (dt, *J* = 15.9, 6.0 Hz, 1H), 4.06 (dd, *J* = 6.0, 1.5 Hz, 2H), 3.84 (s, 6H), 3.82 (s, 3H), 3.37 (s, 3H). ^{13}C NMR (101 MHz, $CDCl_3$) δ 153.3, 137.9, 132.5, 132.4, 125.5, 103.6,

73.0, 60.9, 58.0, 56.1. GC-MS (EI) m/z: calcd for $C_{13}H_{18}O_4$ ($[M]^+$): 238.10; Found 238.15.

General Procedure for the Preparartion of *E/Z*-Mixture Cinnamic Methyl Ether

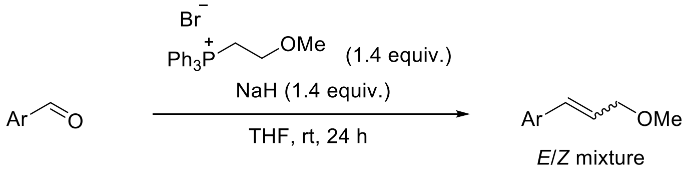

Supplementary Equation 2. Preparartion of *E/Z*-Mixture Cinnamic Methyl Ether.

(2-Methoxyethyl)triphenylphosphonium bromide (5.61 g, 14.0 mmol) was suspended in THF (100 mL) and NaH (60% dispersion in mineral oil, 0.56 g, 14.0 mmol) was added portion-wise at room temperature. After 1h, benzaldehyde (10 mmol) was added slowly and stirred for 24h at the same temperature. The reaction was quenched with saturated aq. NH_4Cl solution and extracted with diethyl ether (3 x 50 mL). The organic phases were combined and washed with brine and dried over Na_2SO_4, after which, the solvent was removed under reduce pressure. The crude product was purified and isolated as a colourless oil using silica gel flash column chromatography with hexane/EA as eluent to give cinnamic methyl ethers of *E/Z* mixture.

1-(*tert*-butyl)-4-(3-methoxyprop-1-en-1-yl)benzene (2c)

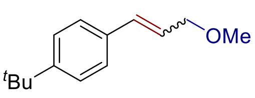

2c

E/Z ratio (15:85). Only NMR of the *Z*-isomer indicated. ^{1}H NMR (400 MHz, $CDCl_3$) δ 7.39 – 7.34 (m, 2H), 7.20 – 7.13 (m, 2H), 6.57 (d, *J* = 11.8 Hz, 1H), 5.80 (dt, *J* = 12.1, 6.2 Hz, 1H), 4.23 (dd, *J* = 6.3, 1.7 Hz, 2H), 3.37 (s, 3H), 1.33 (s, 9H). ^{13}C NMR (101 MHz, $CDCl_3$) δ 150.3, 134.0, 131.5, 128.7, 128.5, 125.3, 69.6, 58.3, 34.7, 31.4. GC-MS (EI) m/z: calcd for $C_{14}H_{20}O$ ($[M]^+$): 204.15; Found 204.15.

1-(3-methoxyprop-1-en-1-yl)-4-(trifluoromethoxy)benzene (2d)

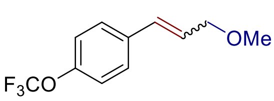

2d

E/Z ratio (11:89). Only NMR of the *Z*-isomer indicated. ^{1}H NMR (400 MHz, $CDCl_3$) δ 7.26 – 7.15 (m, 4H), 6.58 (d, *J* = 11.8 Hz, 1H), 5.89 (dt, *J* = 11.8, 6.4 Hz, 1H), 4.16 (dd, *J* = 6.4, 1.7 Hz, 2H), 3.37 (s, 3H). ^{13}C NMR (101 MHz, $CDCl_3$) δ 148.4, 135.5, 130.5, 130.3, 129.9, 120.8, 120.6 (q, *J* = 258.1 Hz), 69.2, 58.3. GC-MS (EI) m/z: calcd for $C_{11}H_{11}F_3O_2$ ($[M]^+$): 232.10; Found 232.10.

methyl 4-(3-methoxyprop-1-en-1-yl)benzoate (2f)

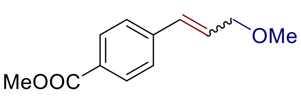

2f

E/Z ratio (9:91). Only NMR of the *Z*-isomer indicated. ^{1}H NMR (400 MHz, $CDCl_3$) δ 8.05 – 7.99 (m, 2H), 7.31 – 7.26 (m, 2H), 6.63 (d, *J* = 11.8 Hz, 1H), 5.95 (dt, *J* = 12.0, 6.3 Hz, 1H), 4.19 (dd, *J* = 6.3, 1.8 Hz, 2H), 3.92 (s, 3H), 3.36 (s, 3H). ^{13}C NMR (101 MHz, $CDCl_3$) δ 166.9, 141.2, 131.1, 130.8, 129.6, 128.8, 128.7, 69.2, 58.3, 52.1. GC-MS (EI) m/z: calcd for $C_{12}H_{14}O_3$ ($[M]^+$): 206.10; Found 206.10.

1-fluoro-4-(3-methoxyprop-1-en-1-yl)benzene (2g)

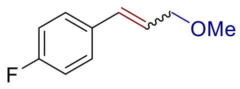

2g

E/Z ratio (38:62). Only NMR of the *Z*-isomer indicated. ^{1}H NMR (400 MHz, $CDCl_3$) δ 7.21 – 7.15 (m, 2H), 7.07 – 7.01 (m, 2H), 6.57 (d, *J* = 11.5 Hz, 1H), 5.83 (dt, *J* = 12.2, 6.4 Hz, 1H), 4.15 (dd, *J* = 6.4, 1.7 Hz, 2H), 3.36 (s, 3H). ^{13}C NMR (101 MHz, $CDCl_3$) δ 159.7 (d, *J* = 232.6 Hz), 130.9, 130.6 (d, *J* = 8.0 Hz), 128.9, 125.9 (d, *J* = 2.1 Hz), 115.3 (d, *J* = 21.5 Hz), 69.2, 58.4. GC-MS (EI) m/z: calcd for $C_{10}H_{11}FO$ ($[M]^+$): 166.10; Found 166.10.

1-(3-methoxyprop-1-en-1-yl)-3-methylbenzene (2h)

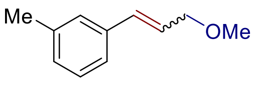

2h

E/Z ratio (17:83). Only NMR of the *Z*-isomer indicated. ^{1}H NMR (500 MHz, $CDCl_3$) δ 7.24 (t, *J* = 7.4 Hz, 1H), 7.08 (d, *J* = 7.8 Hz, 1H), 7.03 – 6.99 (m, 2H), 6.58 (d, *J* = 12.0 Hz, 1H), 5.82 (dt, *J* = 12.1, 6.3 Hz, 1H), 4.21 (dd, *J* = 6.3, 1.5 Hz, 2H), 3.36 (s, 3H), 2.36 (s, 3H). ^{13}C NMR (126 MHz, $CDCl_3$) δ 137.8, 136.7, 131.7, 129.5, 128.9, 128.1, 127.9, 125.8, 69.4, 58.1, 21.4. GC-MS (EI) m/z: calcd for $C_{11}H_{14}O$ ($[M]^+$): 162.10; Found 162.10.

1-fluoro-4-(3-methoxyprop-1-en-1-yl)-2-phenoxybenzene (2j)

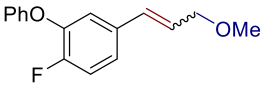

2j

E/Z ratio (12:88). Only NMR of the *Z*-isomer indicated. ^{1}H NMR (400 MHz, $CDCl_3$) δ 7.34 (dd, *J* = 8.7, 7.4 Hz, 2H), 7.18 – 7.07 (m, 2H), 7.04 – 6.97 (m, 2H), 7.00 – 6.95 (m, 1H), 6.91 (dd, *J* = 7.8, 2.2 Hz, 1H), 6.49 (d, *J* = 12.3 Hz, 1H), 5.82 (dt, *J* = 12.1, 6.4 Hz, 1H), 4.08 (dd, *J* = 6.4, 1.7 Hz, 2H), 3.30 (s, 3H). ^{13}C NMR (101 MHz, $CDCl_3$) δ 158.4 (d, *J* = 244.2 Hz), 158.1, 143.5, 133.6, 130.4 (d, *J* = 0.8 Hz), 129.8, 129.3 (d, *J* = 1.4 Hz), 125.1 (d, *J* = 6.9 Hz), 123.4, 121.9 (d, *J* = 1.2 Hz), 117.6, 116.8 (d, *J* = 18.6 Hz), 69.0, 58.2. GC-MS (EI) m/z: calcd for $C_{16}H_{15}FO_2$ ($[M]^+$): 258.10; Found 258.10.

5-(3-methoxyprop-1-en-1-yl)benzo[*d*][1,3]dioxole (2k)

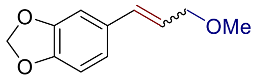

2k

E/Z ratio (12:88). Only NMR of the *Z*-isomer indicated. ^{1}H NMR (400 MHz, $CDCl_3$) δ 6.81 – 6.78 (m, 1H), 6.74 – 6.67 (m, 2H), 6.50 (dt, *J* = 11.9, 1.7 Hz, 1H), 5.97 (s, 2H), 5.75 (dt, *J* = 11.8, 6.3 Hz, 1H), 4.17 (dd, *J* = 6.3, 1.7 Hz, 2H), 3.37 (s, 3H). ^{13}C NMR (101 MHz, $CDCl_3$) δ 147.6, 146.7, 131.4, 130.9, 127.7, 122.7, 109.0, 108.1, 101.1, 69.3, 58.2. GC-MS (EI) m/z: calcd for $C_{11}H_{12}O_3$ ($[M]^+$): 192.10; Found 192.10.

1-fluoro-2-(3-methoxyprop-1-en-1-yl)benzene (2m)

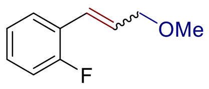

2m

E/Z ratio (10:90). Only NMR of the *Z*-isomer indicated. ^{1}H NMR (400 MHz, $CDCl_3$) δ 7.29 – 7.15 (m, 2H), 7.15 – 7.01 (m, 2H), 6.65 (dd, *J* = 11.7, 1.6 Hz, 1H), 5.96 (dt, *J* = 11.8, 6.4 Hz, 1H), 4.11 (dd, *J* = 6.3, 0.9 Hz, 2H), 3.34 (s, 3H). ^{13}C NMR (101 MHz, $CDCl_3$) δ 160.2 (d, *J* = 241.9 Hz), 131.0, 130.6 (d, *J* = 3.3 Hz), 129.2 (d, *J* = 8.3 Hz), 124.4 (d, *J* = 3.7 Hz), 124.4 (d, *J* = 14.5 Hz), 123.8 (d, *J* = 3.7 Hz), 115.5 (d, *J* = 22.1 Hz), 69.4 (d, *J* = 2.2 Hz), 58.2. GC-MS (EI) m/z: calcd for $C_{10}H_{11}FO$ ($[M]^+$): 166.10; Found 166.10.

1-(3-methoxyprop-1-en-1-yl)-2-methylbenzene (2o)

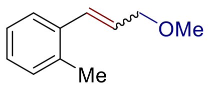
2o

E/Z ratio (12:88). Only NMR of the *Z*-isomer indicated. ^{1}H NMR (400 MHz, $CDCl_3$) δ 7.20 – 7.15 (m, 3H), 7.10 – 7.05 (m, 1H), 6.67 (d, *J* = 11.6 Hz, 1H), 5.89 (dt, *J* = 11.7, 6.6 Hz, 1H), 4.06 (dd, *J* = 6.5, 1.6 Hz, 2H), 3.32 (s, 3H), 2.28 (s, 3H). ^{13}C NMR (101 MHz, $CDCl_3$) δ 135.8, 131.1, 130.0, 129.2, 128.8, 127.6, 125.6, 69.3, 58.2, 20.0. GC-MS (EI) m/z: calcd for $C_{11}H_{14}O$ ($[M]^+$): 162.10; Found 162.10.

9-ethyl-3-(3-methoxyprop-1-en-1-yl)-9*H*-carbazole (2p)

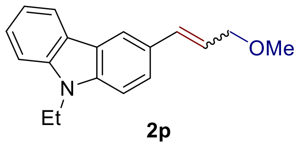
2p

E/Z ratio (12:88). Only NMR of the *Z*-isomer indicated. ^{1}H NMR (400 MHz, $CDCl_3$) δ 8.12 – 8.08 (m, 1H), 7.98 – 7.92 (m, 1H), 7.50 – 7.35 (m, 4H), 7.26 – 7.21 (m, 1H), 6.83 (d, *J* = 11.7 Hz, 1H), 5.84 (dt, *J* = 11.6, 6.4 Hz, 1H), 4.38 (q, *J* = 7.3 Hz, 2H), 4.32 (dd, *J* = 6.4, 1.6 Hz, 2H), 3.41 (s, 3H), 1.45 (t, *J* = 7.2 Hz, 3H). ^{13}C NMR (101 MHz, $CDCl_3$) δ 150.8, 140.5, 139.3, 132.9, 127.8, 127.0, 126.8, 126.0, 123.0, 120.9, 120.6, 119.1, 108.7, 108.3, 69.7, 58.3, 37.8, 14.0. GC-MS (EI) m/z: calcd for $C_{18}H_{19}NO$ ($[M]^+$): 265.15; Found 265.15.

4-(4-(3-methoxyprop-1-en-1-yl)phenyl)morpholine (2q)

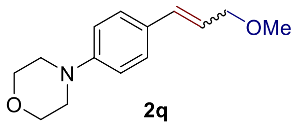
2q

E/Z ratio (10:90). Only NMR of the *Z*-isomer indicated. ^{1}H NMR (400 MHz, $CDCl_3$) δ 7.15 (d, *J* = 8.7 Hz, 2H), 6.88 (d, *J* = 8.8 Hz, 2H), 6.52 (d, *J* = 11.8 Hz, 1H), 5.72 (dt, *J* = 11.8, 6.2 Hz, 1H), 4.21 (dd, *J* = 6.2, 1.7 Hz, 2H), 3.91 – 3.82 (m, 4H), 3.37 (s, 3H), 3.20 – 3.16 (m, 4H). ^{13}C NMR (101 MHz, $CDCl_3$) δ 150.3, 131.4, 130.0, 127.6, 127.0, 115.2, 69.6, 67.0, 58.3, 49.1. GC-MS (EI) m/z: calcd for $C_{14}H_{19}NO_2$ ($[M]^+$): 233.15; Found 233.10.

1,2-dimethoxy-4-(3-methoxyprop-1-en-1-yl)benzene (2s)

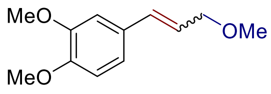
2s

E/Z ratio (11:89). Only NMR of the *Z*-isomer indicated. ^{1}H NMR (500 MHz, $CDCl_3$) δ 6.84 (d, *J* = 8.2 Hz, 1H), 6.81 – 6.74 (m, 2H), 6.54 (d, *J* = 11.6 Hz, 1H), 5.76 (dt, *J* = 12.3, 6.4 Hz, 1H), 4.17 (dd, *J* = 6.7, 1.9 Hz, 2H), 3.87 (s, 6H), 3.36 (s, 3H). ^{13}C NMR (126 MHz, $CDCl_3$) δ 148.7, 148.4, 132.0, 129.7, 127.4, 121.6, 112.2, 110.9, 69.4, 58.2, 56.0, 55.9. GC-MS (EI) m/z: calcd for $C_{12}H_{16}O_3$ ($[M]^+$): 208.10; Found 208.10.

cholesteryl 4-(3-methoxyprop-1-en-1-yl)benzoate (9)

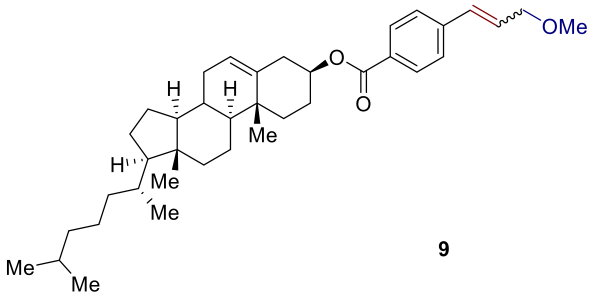
9

E/Z ratio (>95:5). Only NMR of the *E*-isomer indicated. ^{1}H NMR (500 MHz, $CDCl_3$) δ 7.98 (d, *J* = 8.4 Hz, 2H), 7.43 (d, *J* = 8.4 Hz, 2H), 6.65 (d, *J* = 16.0 Hz, 1H), 6.39 (dt, *J* = 16.0, 5.7 Hz, 1H), 5.44 – 5.38 (m, 1H), 4.90 – 4.79 (m, 1H), 4.11 (dd, *J* = 5.7, 1.5 Hz, 2H), 3.41 (s, 3H), 2.46 (d, *J* = 7.6 Hz, 2H), 2.06 – 1.95 (m, 3H), 1.91 (dt, *J* = 13.3, 3.4 Hz, 1H), 1.88 – 1.80 (m, 1H), 1.73 (qd, *J* = 12.5, 3.7 Hz, 1H), 1.58 – 1.43 (m, 6H), 1.38 – 1.09 (m, 12H), 1.06 (s, 3H), 1.04 – 0.96 (m, 2H), 0.92 (d, *J* = 6.5 Hz, 3H), 0.87 (d,

J = 6.6 Hz, 3H), 0.86 (d, *J* = 6.6 Hz, 3H), 0.69 (s, 3H). ^{13}C NMR (126 MHz, $CDCl_3$) δ 165.9, 141.2, 139.8, 131.3, 130.0, 129.9, 128.8, 126.4, 122.9, 74.7, 73.0, 58.4, 56.9, 56.3, 50.2, 42.5, 39.9, 39.7, 38.4, 37.2, 36.8, 36.3, 36.0, 32.1, 32.0, 28.4, 28.2, 28.0, 24.4, 24.0, 23.0, 22.7, 21.2, 19.5, 18.9, 12.0. GC-MS (EI) m/z: calcd for $C_{38}H_{56}O_3$ ($[M]^+$): 560.42; Found 560.40.

General Procedure for the Preparartion of 2-Aryl-allylmethyl Ether

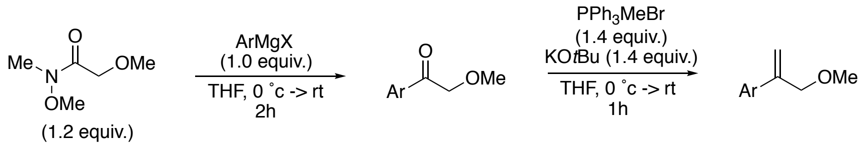

Supplementary Equation 3. Preparartion of 2-Aryl-allylmethyl Ether.

To a solution of N,2-Dimethoxy-N-methylacetamide (1.60 g, 12.0 mmol) synthesized by the literature reported method in THF (50 mL), Grignard solution (10 mmol) was added portion-wise at 0 ˚c. The reaction was stirred at room temperature and monitored with TLC for the completion of reaction. The reaction was quenched with saturated aq. NH_4Cl solution and extracted with diethyl ether (3 x 50 mL). The organic phases were combined and washed with brine and dried over Na_2SO_4, after which, the solvent was removed under reduce pressure. The crude product was purified and isolated as a colourless oil using silica gel flash column chromatography with hexane/EA as eluent and used for the next step. The ketone was then dissolved in THF (10 mL) and slowly added to a stirring mixture of methyltriphenylphosphonium bromide (5.00 g, 14.0 mmol) and potassium *tert*-butoxide (1.57 g, 14.0 mmol) in THF (20 mL) at 0 ˚c. The mixture was warm to room temperature and stirred for three hours. The reaction was quenched with saturated aq. NH_4Cl solution and extracted with diethyl ether (3 x 50 mL). The organic phases were combined and washed with brine and dried over Na_2SO_4, after which, the solvent was removed under reduce pressure. The crude product was purified and isolated as a colourless oil using silica gel flash column chromatography with hexane/EA as eluent to give the 2-aryl-allylmethyl ether.

5-(3-methoxyprop-1-en-2-yl)benzo[*d*][1,3]dioxole (2v)

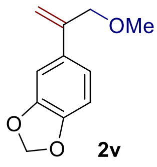

2v

^{1}H NMR (400 MHz, $CDCl_3$) δ 7.00 – 6.94 (m, 2H), 6.78 (d, *J* = 7.9 Hz, 1H), 5.95 (s, 2H), 5.42 (d, *J* = 1.3 Hz, 1H), 5.24 (d, *J* = 1.2 Hz, 1H), 4.26 (d, *J* = 1.2 Hz, 2H), 3.37 (s, 3H). ^{13}C NMR (101 MHz, $CDCl_3$) δ 147.9, 147.4, 143.8, 133.2, 119.8, 113.8, 108.3, 106.8, 101.2, 75.0, 58.0. GC-MS (EI) m/z: calcd for $C_{11}H_{12}O_3$ ($[M]^+$): 192.10; Found 192.05.

1-(3-methoxyprop-1-en-2-yl)-4-(trifluoromethyl)benzene (2w)

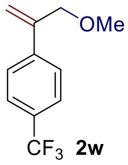

2w

^{1}H NMR (400 MHz, $CDCl_3$) δ 7.62 – 7.54 (m, 4H), 5.61 (d, *J* = 1.1 Hz, 1H), 5.43 (d, *J* = 1.2 Hz, 1H), 4.33 (d, *J* = 1.2 Hz, 2H), 3.38 (s, 3H). ^{13}C NMR (101 MHz, $CDCl_3$) δ 143.4, 142.4, 128.4, 126.6, 125.5 (q, *J* = 3.7 Hz), 124.3 (q, *J* = 271.1 Hz), 116.9, 74.6, 58.1. GC-MS (EI) m/z: calcd for $C_{11}H_{11}F_3O$ ($[M]^+$): 216.10; Found 216.10.

1-(3-methoxyprop-1-en-2-yl)-4-methylbenzene (2x)

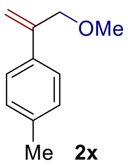
2x

^{1}H NMR (400 MHz, $CDCl_3$) δ 7.39 – 7.34 (m, 2H), 7.19 – 7.13 (m, 2H), 5.50 (d, *J* = 1.5 Hz, 1H), 5.28 (d, *J* = 1.3 Hz, 1H), 4.31 (t, *J* = 0.9 Hz, 2H), 3.38 (s, 3H), 2.35 (s, 3H). ^{13}C NMR (101 MHz, $CDCl_3$) δ 144.1, 137.7, 136.0, 129.2, 126.1, 113.8, 74.8, 58.0, 21.3. GC-MS (EI) m/z: calcd for $C_{11}H_{14}O$ ($[M]^+$): 162.10; Found 162.10.

1-methoxy-3-(3-methoxyprop-1-en-2-yl)benzene (2y)

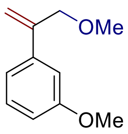
2y

^{1}H NMR (400 MHz, $CDCl_3$) δ 7.27 – 7.24 (m, 1H), 7.06 (ddd, *J* = 7.8, 1.7, 0.9 Hz, 1H), 7.01 (dd, *J* = 2.7, 1.7 Hz, 1H), 6.84 (ddd, *J* = 8.2, 2.5, 0.9 Hz, 1H), 5.53 (d, *J* = 1.4 Hz, 1H), 5.33 (d, *J* = 1.4 Hz, 1H), 4.30 (d, *J* = 1.4 Hz, 2H), 3.82 (s, 3H), 3.39 (s, 3H). ^{13}C NMR (101 MHz, $CDCl_3$) δ 159.8, 144.3, 140.5, 129.5, 118.7, 114.9, 113.2, 112.2, 74.8, 58.1, 55.4. GC-MS (EI) m/z: calcd for $C_{11}H_{14}O_2$ ($[M]^+$): 178.10; Found 178.10.

General Procedure for the Catalytic 1,1,3-Triboronates Synthesis from Cinnamic Methyl Ethers

In an Ar-filled glovebox, a 20-mL screw-capped vial was charged with cobalt(III) acetylacetonate (5.3 mg, 15.0 μmol), xantphos (8.7 mg, 15.0 μmol), nbe (141.2 mg, 1.500 mmol), cinnamyl methyl ether (0.300 mmol), cyclohexane (10 mL) and a magnetic stirring bar. The solution was stirred and pincolborane (261.2 μL, 1.800 mmol) was charged in the vial and sealed with a cap containing a PTFE septum. The vial was removed from the glovebox and stirred at 100 ˚c for 2 h, after which, the crude product was purified using silica gel flash column chromatography (column I.D. 13.4 mm) with gradient increase of ethyl acetate in hexanes as eluent. The conditions for chromatography and data for characterization of the products are given below.

2,2',2''-(3-phenylpropane-1,1,3-triyl)tris(4,4,5,5-tetramethyl-1,3,2-dioxaborolane) (8a)

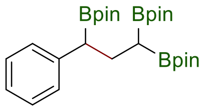

8a

The title compound was isolated (82.2 mg, 0.165 mmol, 55%, 96:4) as a colourless oil after chromatography on silica gel (100:1 to 20:1 hexane/EtOAc). **R_f** = 0.6 (hexane/EtOAc 5:1). ^{1}H NMR (400 MHz, $CDCl_3$) δ 7.23 – 7.15 (m, 4H), 7.10 – 7.05 (m, 1H), 2.36 (t, *J* = 8.2 Hz, 1H), 2.05 (dt, *J* = 13.7, 7.9 Hz, 1H), 1.91 (dt, *J* = 13.6, 8.4 Hz, 1H), 1.22 (s, 12H), 1.21 (s, 6H), 1.20 (s, 12H), 1.18 (s, 6H), 0.84 (t, *J* = 8.2 Hz, 1H). ^{13}C NMR (101 MHz, $CDCl_3$) δ 143.4, 128.8, 128.1, 125.0, 83.1, 82.9, 28.5, 24.9, 24.9, 24.7, 24.6 (the resonance of the carbon with borons attached was not observed). HR-MS (ESI) m/z: calcd for $C_{27}H_{46}B_3O_6$ ([M+H]$^+$): 499.3582; Found 499.3560.

2,2',2''-(3-(*p*-tolyl)propane-1,1,3-triyl)tris(4,4,5,5-tetramethyl-1,3,2-dioxaborolane) (8b)

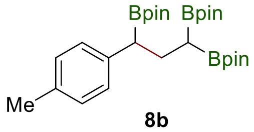

8b

The title compound was isolated (90.6 mg, 0.177 mmol, 59%, 97:3) as a colourless oil after chromatography on silica gel (100:1 to 20:1 hexane/EtOAc). **R_f** = 0.6 (hexane/EtOAc 5:1). ^{1}H NMR (400 MHz, $CDCl_3$) δ 7.06 (d, *J* = 8.1 Hz, 2H), 7.00 (d, *J* = 7.9 Hz, 2H), 2.31 (t, *J* = 8.3 Hz, 1H), 2.26 (s, 3H), 2.01 (dt, *J* = 13.5, 7.8 Hz, 1H), 1.87 (dt, *J* = 13.6, 8.5 Hz, 1H), 1.21 (s, 6H), 1.21 (s, 6H), 1.20 – 1.18 (m, 18H), 1.17 (s, 6H), 0.82 (t, *J* = 8.1 Hz, 1H). ^{13}C NMR (126 MHz, $CDCl_3$) δ 140.2, 134.2, 128.9, 128.6, 83.0, 82.8, 28.7, 24.9, 24.9, 24.7, 24.6, 24.6, 21.0 (the resonance of the carbon with borons attached was not observed). HR-MS (ESI) m/z: calcd for $C_{28}H_{47}B_3NaO_6$ ([M+Na]$^+$): 535.3558; Found 535.3561.

2,2',2''-(3-(4-(*tert*-butyl)phenyl)propane-1,1,3-triyl)tris(4,4,5,5-tetramethyl-1,3,2-dioxaborolane) (8c)

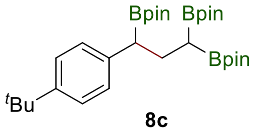

8c

The title compound was isolated (89.8 mg, 0.162 mmol, 54%, 99:1) as a colourless oil after chromatography on silica gel (100:1 to 20:1 hexane/EtOAc). **R_f** = 0.6 (hexane/EtOAc 5:1). ^{1}H NMR (500 MHz, $CDCl_3$) δ 7.20 (d, *J* = 8.3 Hz, 2H), 7.09 (d, *J* = 8.1 Hz, 2H), 2.32 (t, *J* = 8.2 Hz, 1H), 2.01 (dt, *J* = 13.4, 7.9 Hz, 1H), 1.88 (dt, *J* = 13.3, 8.4 Hz, 1H), 1.27 (s, 9H), 1.21 (s, 6H), 1.21 (s, 12H), 1.20 (s, 6H), 1.19 (s, 12H), 0.83 (t, *J* = 8.1 Hz, 1H). ^{13}C NMR (126 MHz, $CDCl_3$) δ 147.4, 140.1, 128.4, 125.0, 83.1, 82.9, 82.9, 34.2, 31.5, 28.8, 24.9, 24.9, 24.8, 24.7, 24.6, 24.6 (the resonance of the carbon with borons attached was not observed). HR-MS (ESI) m/z: calcd for $C_{31}H_{53}B_3NaO_6$ ([M+Na]$^+$): 577.4029; Found 577.4044.

2,2',2''-(3-(4-(trifluoromethoxy)phenyl)propane-1,1,3-triyl)tris(4,4,5,5-tetramethyl-1,3,2-dioxaborolane) (8d)

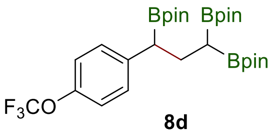

8d

The title compound was isolated (94.3 mg, 0.162 mmol, 54%, 95:5) as a colourless oil after chromatography on silica gel (100:1 to 20:1 hexane/EtOAc). **R_f** = 0.6 (hexane/EtOAc 5:1). ^{1}H NMR (400 MHz, $CDCl_3$) δ 7.21 – 7.14 (m, 2H), 7.06 – 6.99 (m, 2H), 2.37 (t, *J* = 8.1 Hz, 1H), 2.03 (dt, *J* = 13.7, 7.7 Hz, 1H), 1.88 (dt, *J* = 13.6, 8.5 Hz, 1H), 1.20 (s, 12H), 1.19 (s, 6H), 1.18 (s, 12H), 1.17 (s, 6H), 0.79 (t, *J* = 8.1 Hz, 1H). ^{13}C NMR (101 MHz, $CDCl_3$) δ 146.9 (q, *J* = 1.7 Hz), 142.2, 129.9, 120.7, 120.5 (q, *J* = 256.9 Hz), 83.3, 83.0, 83.0, 28.5, 24.8, 24.8, 24.7, 24.6, 24.6 (the resonance of the carbon with borons attached was not observed). HR-MS (ESI) m/z: calcd for $C_{28}H_{44}B_3NaF_3O_7$ ($[M+Na]^+$): 605.3224; Found 605.3231.

2,2',2''-(3-([1,1'-biphenyl]-4-yl)propane-1,1,3-triyl)tris(4,4,5,5-tetramethyl-1,3,2-dioxaborolane) (8e)

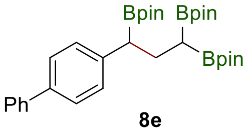

8e

The title compound was isolated (106.8 mg, 0.186 mmol, 62%, 94:6) as a colourless oil after chromatography on silica gel (100:1 to 20:1 hexane/EtOAc). **R_f** = 0.6 (hexane/EtOAc 5:1). ^{1}H NMR (500 MHz, $CDCl_3$) δ 7.61 – 7.57 (m, 2H), 7.49 – 7.46 (m, 2H), 7.45 – 7.41 (m, 2H), 7.32 (t, *J* = 7.5 Hz, 1H), 7.30 – 7.26 (m, 2H), 2.44 (t, *J* = 8.2 Hz, 1H), 2.11 (dt, *J* = 13.6, 7.7 Hz, 1H), 1.98 (dt, *J* = 13.7, 8.6 Hz, 1H), 1.26 – 1.25 (m, 12H), 1.25 (s, 6H), 1.24 (s, 6H), 1.23 (s, 12H), 0.90 (t, *J* = 8.0 Hz, 1H). ^{13}C NMR (126 MHz, $CDCl_3$) δ 142.8, 141.6, 138.0, 129.3, 128.7, 127.1, 127.1, 126.8, 83.3, 83.0, 83.0, 28.7, 25.0, 25.0, 24.9, 24.8, 24.8 (the resonance of the carbon with borons attached was not observed). HR-MS (ESI) m/z: calcd for $C_{33}H_{49}B_3O_6$ ($[M+H]^+$): 575.3897; Found 575.3911.

methyl 4-(1,3,3-tris(4,4,5,5-tetramethyl-1,3,2-dioxaborolan-2-yl)propyl)benzoate (8f)

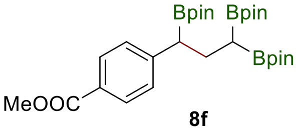

8f

1:1 Mixture of THF/Cyclohexane was used as the solvent. The title compound was isolated (75.1 mg, 0.135 mmol, 45%, 97:3) as a colourless oil after chromatography on silica gel (20:1 to 15:1 hexane/EtOAc). **R_f** = 0.4 (hexane/EtOAc 5:1). ^{1}H NMR (500 MHz, $CDCl_3$) δ 7.87 (d, *J* = 8.1 Hz, 2H), 7.23 (d, *J* = 8.3 Hz, 2H), 3.85 (s, 3H), 2.44 (t, *J* = 8.2 Hz, 1H), 2.06 (dt, *J* = 13.8, 7.9 Hz, 1H), 1.91 (dt, *J* = 13.6, 8.5 Hz, 1H), 1.20 (s, 12H), 1.19 (s, 6H), 1.18 (s, 6H), 1.16 (s, 6H), 1.15 (s, 6H), 0.78 (t, *J* = 8.1 Hz, 1H). ^{13}C NMR (126 MHz, $CDCl_3$) δ 167.4, 149.4, 129.6, 128.6, 127.0, 83.3, 83.0, 51.8, 28.0, 24.9, 24.8, 24.7, 24.6, 24.6, 24.6 (the resonance of the carbon with borons attached was not observed). HR-MS (ESI) m/z: calcd for $C_{29}H_{47}B_3NaO_8$ ($[M+Na]^+$): 579.3457; Found 579.3465.

2,2',2''-(3-(4-fluorophenyl)propane-1,1,3-triyl)tris(4,4,5,5-tetramethyl-1,3,2-dioxaborolane) (8g)

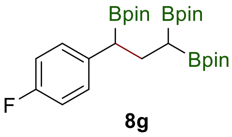

8g

The title compound was isolated (85.3 mg, 0.165 mmol, 55%, 95:5) as a white solid after chromatography on silica gel (100:1 to 20:1 hexane/EtOAc). **R_f** = 0.6 (hexane/EtOAc 5:1). ^{1}H NMR (400 MHz, $CDCl_3$) δ 7.15 – 7.08 (m, 2H), 6.91 – 6.84 (m, 2H), 2.33 (t, *J* = 8.3 Hz, 1H), 2.01 (dt, *J* = 13.7, 7.8 Hz, 1H), 1.86 (dt, *J* = 13.6, 8.5 Hz, 1H), 1.20 (s, 12H), 1.19 (s, 6H), 1.19 (s, 6H), 1.18 (s, 6H), 1.17 (s, 6H), 0.79 (t, *J* = 8.1 Hz, 1H). ^{13}C NMR (101 MHz, $CDCl_3$) δ 161.0 (d, *J* = 241.9 Hz), 139.1 (d, *J* = 3.1 Hz), 130.1 (d, *J* = 7.7 Hz), 114.9 (d, *J* =

20.9 Hz), 83.3, 83.0, 83.0, 28.7, 25.0, 25.0, 24.8, 24.7, 24.7 (the resonance of the carbon with borons attached was not observed). HR-MS (ESI) m/z: calcd for $C_{27}H_{44}B_3NaFO_6$ ($[M+Na]^+$): 539.3307; Found 539.3321.

Crystals suitable for XRD analysis were attained by slow evaporation of **8g** in Et_2O:

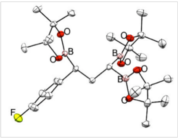

Supplementary Figure 2. X-ray structure of **8g**.

2,2',2''-(3-(*m*-tolyl)propane-1,1,3-triyl)tris(4,4,5,5-tetramethyl-1,3,2-dioxaborolane) (8h)

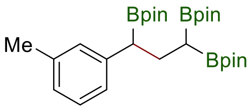

8h

The title compound was isolated (86.0 mg, 0.168 mmol, 56%, 97:3) as a colourless oil after chromatography on silica gel (100:1 to 20:1 hexane/EtOAc). $\mathbf{R_f}$ = 0.6 (hexane/EtOAc 5:1). ^{1}H NMR (500 MHz, $CDCl_3$) δ 7.10 – 7.05 (m, 1H), 7.00 – 6.96 (m, 2H), 6.89 (d, *J* = 7.5 Hz, 1H), 2.32 (t, *J* = 8.3 Hz, 1H), 2.27 (s, 3H), 2.03 (dt, *J* = 13.6, 7.9 Hz, 1H), 1.89 (dt, *J* = 13.5, 8.5 Hz, 1H), 1.22 (s, 6H), 1.21 (s, 6H), 1.20 (s, 6H), 1.20 (s, 12H), 1.18 (s, 6H), 0.84 (t, *J* = 8.1 Hz, 1H). ^{13}C NMR (126 MHz, $CDCl_3$) δ 143.3, 137.4, 129.8, 128.0, 125.8, 125.7, 83.1, 82.9, 28.6, 24.9, 24.9, 24.7, 24.7, 24.6, 21.4 (the resonance of the carbon with borons attached was not observed). HR-MS (ESI) m/z: calcd for $C_{28}H_{47}B_3NaO_6$ ($[M+Na]^+$): 535.3558; Found 535.3568.

2,2',2''-(3-(3,5-bis(trifluoromethyl)phenyl)propane-1,1,3-triyl)tris(4,4,5,5-tetramethyl-1,3,2-dioxaborolane) (8i)

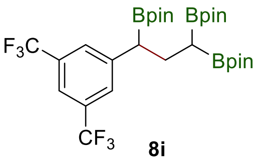

8i

1:1 Mixture of THF/Cyclohexane was used as the solvent. The title compound was isolated (74.2 mg, 0.117 mmol, 39%, 97:3) as a colourless oil after chromatography on silica gel (100:1 to 20:1 hexane/EtOAc). $\mathbf{R_f}$ = 0.6 (hexane/EtOAc 5:1). ^{1}H NMR (500 MHz, $CDCl_3$) δ 7.66 (d, *J* = 2.0 Hz, 1H), 7.61 (s, 1H), 2.54 (t, *J* = 8.3 Hz, 1H), 2.07 (dt, *J* = 13.8, 7.8 Hz, 1H), 1.92 (dt, *J* = 13.7, 8.6 Hz, 1H), 1.22 (s, 12H), 1.21 (s, 6H), 1.20 (s, 6H), 1.20 (s, 6H), 1.19 (s, 6H), 0.78 (t, *J* = 8.1 Hz, 1H). ^{13}C NMR (126 MHz, $CDCl_3$) δ 146.4, 131.3 (q, *J* = 32.7 Hz), 129.2, 123.8 (q, *J* = 272.6 Hz), 119.3 (q, *J* = 3.7 Hz), 83.9, 83.3, 83.3, 28.7, 25.0, 25.0, 24.9, 24.8, 24.7, 24.6 (the resonance of the carbon with borons attached was not observed). HR-MS (ESI) m/z: calcd for $C_{29}H_{43}B_3NaF_6O_6$ ($[M+Na]^+$): 657.3150; Found 657.3165.

2,2',2''-(3-(4-fluoro-3-phenoxyphenyl)propane-1,1,3-triyl)tris(4,4,5,5-tetramethyl-1,3,2-dioxaborolane) (8j)

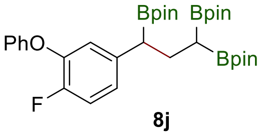

8j

The title compound was isolated (114.9 mg, 0.189 mmol, 63%, 95:5) as a colourless oil after chromatography on silica gel (100:1 to 20:1 hexane/EtOAc). $\mathbf{R_f}$ = 0.5 (hexane/EtOAc 5:1). ^{1}H NMR (400 MHz, $CDCl_3$) δ 7.31 – 7.23 (m, 2H), 7.07 – 6.98 (m, 2H), 6.98 – 6.88 (m, 4H), 2.31 (t, *J* = 8.3 Hz, 1H), 1.98 (dt, *J* = 13.7, 7.9 Hz, 1H), 1.84 (dt, *J* = 13.6, 8.6 Hz, 1H), 1.19 (s, 24H), 1.15 (s, 12H), 0.79 (t, *J* = 8.1 Hz, 1H). ^{13}C NMR (101 MHz, $CDCl_3$) δ 157.8, 152.4 (d, *J* = 245.3 Hz), 143.0 (d, *J* = 11.6 Hz), 140.4 (d, *J* =

3.6 Hz), 129.6, 125.1 (d, *J* = 6.5 Hz), 122.7, 122.2, 117.3, 116.5 (d, *J* = 18.1 Hz), 83.4, 83.0, 28.5, 25.0, 24.9, 24.8, 24.7, 24.6 (the resonance of the carbon with borons attached was not observed). HR-MS (ESI) m/z: calcd for $C_{33}H_{48}B_3NaFO_7$ ($[M+Na]^+$): 631.3572; Found 631.3573.

2,2',2''-(3-(benzo[*d*][1,3]dioxol-5-yl)propane-1,1,3-triyl)tris(4,4,5,5-tetramethyl-1,3,2-dioxaborolane) (8k)

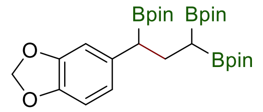

8k

The title compound was isolated (81.3 mg, 0.150 mmol, 50%, 97:3) as a colourless oil after chromatography on silica gel (100:1 to 20:1 hexane/EtOAc). $\mathbf{R_f}$ = 0.5 (hexane/EtOAc 5:1). ^{1}H NMR (500 MHz, $CDCl_3$) δ 6.70 (d, *J* = 1.6 Hz, 1H), 6.65 (d, *J* = 7.9 Hz, 1H), 6.61 (dd, *J* = 8.0, 1.6 Hz, 1H), 5.90 – 5.83 (m, 2H), 2.27 (t, *J* = 8.3 Hz, 1H), 1.99 (dt, *J* = 13.6, 7.9 Hz, 1H), 1.84 (dt, *J* = 13.6, 8.5 Hz, 1H), 1.21 (s, 6H), 1.21 (s, 6H), 1.20 (s, 12H), 1.19 (s, 6H), 1.18 (s, 6H), 0.80 (t, *J* = 8.0 Hz, 1H). ^{13}C NMR (101 MHz, $CDCl_3$) δ 147.3, 145.0, 137.2, 121.6, 109.2, 108.0, 100.5, 83.1, 82.9, 82.9, 28.7, 24.9, 24.9, 24.7, 24.6, 24.6 (the resonance of the carbon with borons attached was not observed). HR-MS (ESI) m/z: calcd for $C_{28}H_{45}B_3NaO_8$ ($[M+Na]^+$): 565.3300; Found 565.3315.

2,2',2''-(3-(naphthalen-2-yl)propane-1,1,3-triyl)tris(4,4,5,5-tetramethyl-1,3,2-dioxaborolane) (8l)

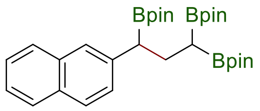

8l

1:1 Mixture of THF/Cyclohexane was used as the solvent. The title compound was isolated (69.1 mg, 0.126 mmol, 42%, 97:3) as a colourless oil after chromatography on silica gel (100:1 to 20:1 hexane/EtOAc). $\mathbf{R_f}$ = 0.6 (hexane/EtOAc 5:1). ^{1}H NMR (400 MHz, $CDCl_3$) δ 7.77 – 7.67 (m, 3H), 7.62 (s, 1H), 7.42 – 7.32 (m, 3H), 2.55 (t, *J* = 8.2 Hz, 1H), 2.15 (dt, *J* = 13.7, 7.8 Hz, 1H), 2.02 (dt, *J* = 13.6, 8.5 Hz, 1H), 1.23 (s, 12H), 1.20 (s, 6H), 1.19 (s, 6H), 1.18 (s, 6H), 1.17 (s, 6H), 0.86 (t, *J* = 8.1 Hz, 1H). ^{13}C NMR (101 MHz, $CDCl_3$) δ 141.0, 133.9, 131.8, 127.9, 127.5, 127.5, 127.4, 126.8, 125.4, 124.6, 83.2, 82.9, 82.9, 28.3, 24.9, 24.7, 24.7, 24.6, 24.6 (the resonance of the carbon with borons attached was not observed). HR-MS (ESI) m/z: calcd for $C_{31}H_{47}B_3NaO_6$ ($[M+Na]^+$): 571.3559; Found 571.3568.

2,2',2''-(3-(2-fluorophenyl)propane-1,1,3-triyl)tris(4,4,5,5-tetramethyl-1,3,2-dioxaborolane) (8m)

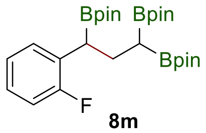

8m

The title compound was isolated (85.2 mg, 0.165 mmol, 55%, 99:1) as a colourless oil after chromatography on silica gel (100:1 to 20:1 hexane/EtOAc). $\mathbf{R_f}$ = 0.6 (hexane/EtOAc 5:1). ^{1}H NMR (400 MHz, $CDCl_3$) δ 7.20 (td, *J* = 7.6, 1.9 Hz, 1H), 7.08 – 7.01 (m, 1H), 6.98 (td, *J* = 7.4, 1.4 Hz, 1H), 6.92 (ddd, *J* = 9.5, 7.9, 1.3 Hz, 1H), 2.57 (t, *J* = 8.1 Hz, 1H), 2.08 (dt, *J* = 13.7, 7.9 Hz, 1H), 1.85 (dt, *J* = 13.6, 8.4 Hz, 1H), 1.22 (s, 6H), 1.21 (s, 6H), 1.21 (s, 6H), 1.19 (s, 6H), 1.19 (s, 12H), 0.83 (t, *J* = 8.0 Hz, 1H). ^{13}C NMR (101 MHz, $CDCl_3$) δ 161.2 (d, *J* = 243.6 Hz), 131.0 (d, *J* = 5.0 Hz), 130.6 (d, *J* = 16.2 Hz), 126.6 (d, *J* = 8.1 Hz), 123.9 (d, *J* = 3.5 Hz), 115.1 (d, *J* = 22.9 Hz), 83.3, 83.0, 83.0, 27.2, 25.0, 25.0, 24.8, 24.7, 24.7 (the resonance of the carbon with borons attached was not observed). HR-MS (ESI) m/z: calcd for $C_{27}H_{44}B_3NaFO_6$ ($[M+Na]^+$): 539.3307; Found 539.3323.

2,2',2''-(3-(2-methoxyphenyl)propane-1,1,3-triyl)tris(4,4,5,5-tetramethyl-1,3,2-dioxaborolane) (8n)

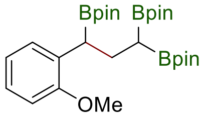

8n

The title compound was isolated (71.3 mg, 0.135 mmol, 45%, 94:6) as a colourless oil after chromatography on silica gel (20:1 to 15:1 hexane/EtOAc). **R$_f$** = 0.5 (hexane/EtOAc 5:1). ^{1}H NMR (500 MHz, $CDCl_3$) δ 7.13 (dd, 1H), 7.07 (td, *J* = 7.8, 1.7 Hz, 1H), 6.81 (td, *J* = 7.4, 0.9 Hz, 1H), 6.75 (d, *J* = 8.1 Hz, 1H), 3.75 (s, 3H), 2.40 (dd, *J* = 8.6, 7.2 Hz, 1H), 2.06 (dt, *J* = 13.7, 7.8 Hz, 1H), 1.82 (dt, *J* = 13.5, 8.3 Hz, 1H), 1.23 (s, 6H), 1.22 (s, 6H), 1.21 (s, 6H), 1.19 (s, 6H), 1.18 (s, 6H), 1.18 (s, 6H), 0.87 (t, *J* = 8.0 Hz, 1H). ^{13}C NMR (126 MHz, $CDCl_3$) δ 157.3, 132.4, 130.9, 126.3, 120.6, 110.1, 82.9, 82.9, 55.1, 27.0, 25.1, 25.0, 24.9, 24.8, 24.8, 24.7 6 (the resonance of the carbon with borons attached was not observed). HR-MS (ESI) m/z: calcd for $C_{28}H_{47}B_3NaO_7$ ([M+Na]$^+$): 551.3507; Found 551.3515.

2,2',2''-(3-(*o*-tolyl)propane-1,1,3-triyl)tris(4,4,5,5-tetramethyl-1,3,2-dioxaborolane) (8o)

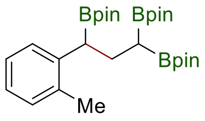

8o

The title compound was isolated (67.6 mg, 0.132 mmol, 44%, 97:3) as a colourless oil after chromatography on silica gel (100:1 to 20:1 hexane/EtOAc). **R$_f$** = 0.6 (hexane/EtOAc 5:1). ^{1}H NMR (500 MHz, $CDCl_3$) δ 7.23 – 7.18 (m, 1H), 7.09 – 7.02 (m, 2H), 7.01 – 6.94 (m, 1H), 2.60 (dd, *J* = 9.8, 6.6 Hz, 1H), 2.35 (s, 3H), 2.02 – 1.94 (m, 1H), 1.91 – 1.85 (m, 1H), 1.23 (s, 6H), 1.22 (s, 6H), 1.20 (s, 12H), 1.20 (s, 6H), 1.18 (s, 6H), 0.90 (t, *J* = 10.5 Hz, 1H). ^{13}C NMR (126 MHz, $CDCl_3$) δ 142.2, 136.4, 130.2, 128.3, 125.8, 124.9, 83.1, 83.0, 83.0, 28.5, 25.0, 25.0, 24.9, 24.8, 24.8, 24.7, 20.2 (the resonance of the carbon with borons attached was not observed). HR-MS (ESI) m/z: calcd for $C_{28}H_{47}B_3NaO_6$ ([M+Na]$^+$): 535.3558; Found 535.3570.

9-ethyl-3-(1,3,3-tris(4,4,5,5-tetramethyl-1,3,2-dioxaborolan-2-yl)propyl)-9*H*-carbazole (8p)

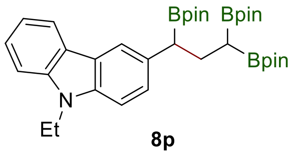

8p

The title compound was isolated (84.9 mg, 0.138 mmol, 46%, 99:1) as a colourless oil after chromatography on silica gel (20:1 to 15:1 hexane/EtOAc). **R$_f$** = 0.5 (hexane/EtOAc 5:1). ^{1}H NMR (400 MHz, $CDCl_3$) δ 8.03 (d, *J* = 7.7 Hz, 1H), 7.93 (d, *J* = 1.4 Hz, 1H), 7.41 (ddd, *J* = 8.1, 7.0, 1.2 Hz, 1H), 7.35 (d, *J* = 8.1 Hz, 1H), 7.31 (dd, *J* = 8.4, 1.7 Hz, 1H), 7.26 (d, *J* = 8.2 Hz, 1H), 7.19 – 7.14 (m, 1H), 4.32 (q, *J* = 7.2 Hz, 2H), 2.54 (t, *J* = 8.3 Hz, 1H), 2.15 (dt, *J* = 13.6, 7.8 Hz, 1H), 2.02 (dt, *J* = 13.6, 8.6 Hz, 1H), 1.41 (t, *J* = 7.2 Hz, 3H), 1.23 (s, 12H), 1.23 (s, 6H), 1.19 (s, 12H), 1.18 (s, 6H), 0.89 (t, *J* = 5.0 Hz, 1H). ^{13}C NMR (101 MHz, $CDCl_3$) δ 140.1, 138.3, 133.7, 127.0, 125.0, 123.1, 123.0, 120.3, 120.3, 118.2, 108.2, 108.1, 83.1, 82.9, 82.8, 37.5, 29.2, 24.9, 24.9, 24.8, 24.7, 24.6, 13.9 (the resonance of the carbon with borons attached was not observed). HR-MS (ESI) m/z: calcd for $C_{35}H_{52}B_3NaNO_6$ ([M+Na]$^+$): 638.3983; Found 638.3985.

4-(4-(1,3,3-tris(4,4,5,5-tetramethyl-1,3,2-dioxaborolan-2-yl)propyl)phenyl)morpholine (8q)

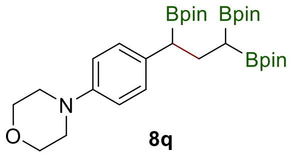

8q

The title compound was isolated (92.7 mg, 0.159 mmol, 53%, 98:2) as a colourless oil after chromatography on silica gel (20:1 to 5:1 hexane/EtOAc). **R$_f$** = 0.35 (hexane/EtOAc 3:1).^{1}H NMR (500 MHz, $CDCl_3$) δ 7.08 (d, *J* = 8.5 Hz, 2H), 6.78 (d, *J* = 8.5 Hz, 2H), 3.85 – 3.82 (m, 4H), 3.12 – 3.06 (m, 4H), 2.27 (t, *J* = 8.2 Hz, 1H), 1.99 (dt, *J* = 13.4, 7.9 Hz, 1H), 1.85 (dt, *J* = 13.5, 8.5 Hz, 1H), 1.21 (s, 6H), 1.20 (s, 6H), 1.19 (s, 6H), 1.18 (s, 12H), 1.17 (s, 6H), 0.81 (t, *J* = 7.9 Hz, 1H). ^{13}C NMR (126 MHz, $CDCl_3$) δ 148.7, 135.2, 129.4, 116.1, 83.0, 82.9, 82.8, 67.0, 50.0, 28.6, 24.9, 24.9, 24.7, 24.7, 24.6, 24.6

(the resonance of the carbon with borons attached was not observed). HR-MS (ESI) m/z: calcd for $C_{31}H_{52}B_3NaNO_7$ ($[M+Na]^+$): 606.3930; Found 606.3942.

2,2',2''-(3-(4-methoxyphenyl)propane-1,1,3-triyl)tris(4,4,5,5-tetramethyl-1,3,2-dioxaborolane) (8r)

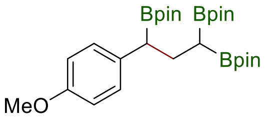

8r

The title compound was isolated (95.1 mg, 0.180 mmol, 60%, 94:6) as a colourless oil after chromatography on silica gel (20:1 to 15:1 hexane/EtOAc). **R$_f$** = 0.5 (hexane/EtOAc 5:1). ^{1}H NMR (500 MHz, $CDCl_3$) δ 7.11 – 7.05 (m, 2H), 6.77 – 6.71 (m, 2H), 3.73 (s, 3H), 2.28 (t, *J* = 8.3 Hz, 1H), 1.99 (dt, *J* = 13.6, 7.8 Hz, 1H), 1.85 (dt, *J* = 13.6, 8.5 Hz, 1H), 1.20 (s, 6H), 1.20 (s, 6H), 1.19 (s, 6H), 1.18 (s, 12H), 1.16 (s, 6H), 0.81 (t, *J* = 8.1 Hz, 1H). ^{13}C NMR (126 MHz, $CDCl_3$) δ 157.3, 135.5, 129.7, 113.7, 83.1, 83.0, 82.9, 55.2, 28.8, 25.0, 24.9, 24.8, 24.7 6 (the resonance of the carbon with borons attached was not observed). HR-MS (ESI) m/z: calcd for $C_{28}H_{47}B_3NaO_7$ ($[M+Na]^+$): 551.3507; Found 551.3526.

2,2',2''-(3-(3,4-dimethoxyphenyl)propane-1,1,3-triyl)tris(4,4,5,5-tetramethyl-1,3,2-dioxaborolane) (8s)

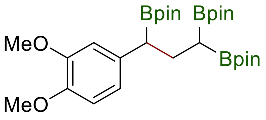

8s

The title compound was isolated (100.5 mg, 0.180 mmol, 60%, 93:7) as a colourless oil after chromatography on silica gel (20:1 to 15:1 hexane/EtOAc). **R$_f$** = 0.4 (hexane/EtOAc 4:1). ^{1}H NMR (500 MHz, $CDCl_3$) δ 6.75 – 6.69 (m, 3H), 3.82 (s, 3H), 3.80 (s, 3H), 2.25 (t, *J* = 8.1 Hz, 1H), 1.99 (dt, *J* = 12.8, 7.6 Hz, 1H), 1.86 (dt, *J* = 13.8, 8.6 Hz, 1H), 1.20 (s, 12H), 1.19 (s, 6H), 1.18 (s, 6H), 1.17 (s, 12H), 0.82 (t, *J* = 8.3 Hz, 1H). ^{13}C NMR (126 MHz, $CDCl_3$) δ 148.6, 146.7, 136.0, 120.8, 112.2, 111.3, 83.2, 83.0, 83.0, 55.9, 55.7, 28.9, 25.0, 24.9, 24.9, 24.7, 24.7, 24.6 (the resonance of the carbon with borons attached was not observed). HR-MS (ESI) m/z: calcd for $C_{29}H_{49}B_3NaO_8$ ($[M+Na]^+$): 581.3813; Found 581.3820.

***tert*-Butyl(2-methoxy-4-(1,3,3-tris(4,4,5,5-tetramethyl-1,3,2-dioxaborolan-2-yl)propyl)phenoxy)dimethylsilane (8t)**

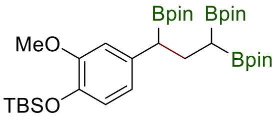

8t

The title compound was isolated (110.6 mg, 0.168 mmol, 56%, 99:1) as a colourless oil after chromatography on silica gel (20:1 to 15:1 hexane/EtOAc). **R$_f$** = 0.4 (hexane/EtOAc 4:1). ^{1}H NMR (400 MHz, $CDCl_3$) δ 6.70 – 6.65 (m, 2H), 6.63 – 6.59 (m, 1H), 3.75 (s, 3H), 2.27 (t, *J* = 8.3 Hz, 1H), 2.00 (dt, *J* = 13.5, 8.0 Hz, 1H), 1.88 (dt, *J* = 13.6, 8.6 Hz, 1H), 1.21 (s, 18H), 1.20 (s, 6H), 1.18 (s, 6H), 1.16 (s, 6H), 0.97 (s, 9H), 0.87 (t, *J* = 7.5 Hz, 1H), 0.11 (s, 6H). ^{13}C NMR (101 MHz, $CDCl_3$) δ 150.4, 142.4, 136.7, 120.9, 120.5, 112.8, 83.1, 82.9, 55.3, 28.6, 25.8, 24.9, 24.9, 24.7, 24.7, 24.6, 24.6, 18.4, -4.6 (the resonance of the carbon with borons attached was not observed). GC-MS (EI) m/z: calcd for $C_{30}H_{52}B_3O_8Si$ ($[M-C(CH_3)_3]^+$): 601.40; Found 601.40.

2,2',2''-(3-(3,4,5-trimethoxyphenyl)propane-1,1,3-triyl)tris(4,4,5,5-tetramethyl-1,3,2-dioxaborolane) (8u)

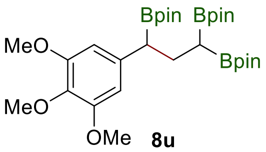

8u

The title compound was isolated (106.7 mg, 0.162 mmol, 54%, 99:1) as a colourless oil after chromatography on silica gel (20:1 to 5:1 hexane/EtOAc). **R$_f$** = 0.4 (hexane/EtOAc 3:1).^{1}H NMR (400 MHz, $CDCl_3$) δ 6.41 (d, *J* = 2.0 Hz, 2H), 3.81 (s, 6H), 3.78 (s, 3H), 2.24 (t, *J* = 8.2 Hz, 1H), 2.01 (dt, *J* = 13.6, 7.7 Hz,

1H), 1.88 (dt, *J* = 13.6, 8.5 Hz, 1H), 1.22 (s, 18H), 1.20 (s, 6H), 1.19 (s, 6H), 1.19 (s, 6H), 0.83 (t, *J* = 8.0 Hz, 1H). ^{13}C NMR (101 MHz, $CDCl_3$) δ 152.8, 139.0, 135.5, 105.8, 83.2, 82.9, 82.9, 60.8, 55.8, 28.9, 24.9, 24.7, 24.6, 24.6 (the resonance of the carbon with borons attached was not observed). HR-MS (ESI) m/z: calcd for $C_{30}H_{51}B_3NaO_9$ ([M+Na]$^+$): 611.3719; Found 611.3726.

General Procedure for the Catalytic 1,1,3-Triboronates Synthesis from 2-Aryl-allylmethyl Ethers

In an Ar-filled glovebox, a 20-mL screw-capped vial was charged with cobalt(III) acetylacetonate (5.3 mg, 15.0 μmol), xantphos (8.7 mg, 15.0 μmol), nbe (141.2 mg, 1.500 mmol), 2-Aryl-allylmethyl ether (0.300 mmol), cyclohexane (10 mL) and a magnetic stirring bar. The solution was stirred and pincolborane (261.2 μL, 1.800 mmol) was charged in the vial and sealed with a cap containing a PTFE septum. The vial was removed from the glovebox and stirred at 100 ˚c for 2 h, after which, the crude product was purified using silica gel flash column chromatography (column I.D. 13.4 mm) with gradient increase of ethyl acetate in hexanes as eluent. The conditions for chromatography and data for characterization of the products are given below.

2,2',2''-(2-(benzo[*d*][1,3]dioxol-5-yl)propane-1,1,3-triyl)tris(4,4,5,5-tetramethyl-1,3,2-dioxaborolane) (8v)

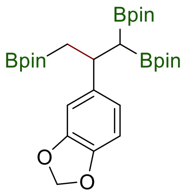

8v

The title compound was isolated (76.4 mg, 0.141 mmol, 47%) as a colourless oil after chromatography on silica gel (20:1 to 15:1 hexane/EtOAc). **R_f** = 0.4 (hexane/EtOAc 5:1). ^{1}H NMR (400 MHz, $CDCl_3$) δ 6.81 (d, *J* = 1.7 Hz, 1H), 6.75 (dd, *J* = 8.0, 1.7 Hz, 1H), 6.65 – 6.61 (m, 1H), 5.83 (s, 2H), 3.26 (td, *J* = 11.5, 3.9 Hz, 1H), 1.30 – 1.26 (m, 2H), 1.24 (s, 6H), 1.23 (s, 6H), 1.11 (dd, *J* = 15.0, 11.3 Hz, 1H), 1.04 (s, 12H), 0.98 (s, 6H), 0.96 (s, 6H). ^{13}C NMR (101 MHz, $CDCl_3$) δ 146.7, 145.0, 143.2, 120.7, 108.7, 107.4, 100.3, 83.0, 82.7, 82.6, 38.8, 24.9, 24.8, 24.6, 24.5, 24.5, 24.3 (the resonance of the carbon with borons attached was not observed). HR-MS (ESI) m/z: calcd for $C_{28}H_{45}B_3NaO_8$ ([M+Na]$^+$): 565.3300; Found 565.3312.

2,2',2''-(2-(4-(trifluoromethyl)phenyl)propane-1,1,3-triyl)tris(4,4,5,5-tetramethyl-1,3,2-dioxaborolane) (8w)

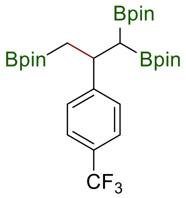

8w

The title compound was isolated (71.4 mg, 0.126 mmol, 42%) as a colourless oil after chromatography on silica gel (100:0 to 30:1 hexane/EtOAc). **R_f** = 0.5 (hexane/EtOAc 5:1). ^{1}H NMR (400 MHz, $CDCl_3$) δ 7.45 – 7.37 (m, 4H), 3.36 (td, *J* = 11.6, 3.9 Hz, 1H), 1.36 – 1.30 (m, 2H), 1.24 (s, 6H), 1.23 (s, 6H), 1.16 – 1.11 (m, 1H), 0.98 (s, 12H), 0.92 (s, 6H), 0.89 (s, 6H). ^{13}C NMR (101 MHz, $CDCl_3$) δ 153.2, 128.3, 127.7 (q, *J* = 32.2 Hz), 124.5 (q, *J* = 272.2 Hz), 124.5 (q, *J* = 3.8 Hz), 83.2, 82.9, 82.8, 38.8, 24.9, 24.7, 24.5, 24.5, 24.4, 24.2 (the resonance of the carbon with borons attached was not observed). HR-MS (ESI) m/z: calcd for $C_{28}H_{44}B_3NaF_3O_6$ ([M+Na]$^+$): 589.3275; Found 589.3288.

2,2',2''-(2-(*p*-tolyl)propane-1,1,3-triyl)tris(4,4,5,5-tetramethyl-1,3,2-dioxaborolane) (8x)

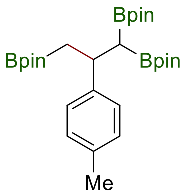

8x

The title compound was isolated (70.7 mg, 0.138 mmol, 46%) as a colourless oil after chromatography on silica gel (100:0 to 20:1 hexane/EtOAc). **R_f** = 0.5 (hexane/EtOAc 5:1). ^{1}H NMR (400 MHz, $CDCl_3$) δ 7.14 (d, *J* = 8.0 Hz, 2H), 6.95 (d, *J* = 7.9 Hz, 2H), 3.27 (td, *J* = 11.5, 3.9 Hz, 1H), 2.22 (s, 3H), 1.34 – 1.25 (m, 2H), 1.24 (s, 6H), 1.22 (s, 6H), 1.14 (dd, *J* = 15.1, 11.3 Hz, 1H), 1.00 (s, 6H), 0.99 (s, 6H), 0.93 (s, 6H), 0.91 (s, 6H). ^{13}C NMR (101 MHz, $CDCl_3$) δ 145.7, 134.5, 128.1, 127.7, 82.9, 82.6, 82.6, 38.5, 24.9, 24.7, 24.6, 24.5, 24.4, 24.3, 21.0 (the resonance of the carbon with borons attached was not observed). HR-MS (ESI) m/z: calcd for $C_{28}H_{47}B_3NaO_6$ ([M+Na]$^+$): 535.3558; Found 535.3574.

2,2',2''-(2-(3-methoxyphenyl)propane-1,1,3-triyl)tris(4,4,5,5-tetramethyl-1,3,2-dioxaborolane) (8y)

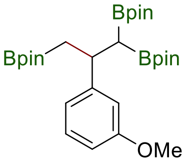

8y

The title compound was isolated (87.1 mg, 0.165 mmol, 55%) as a colourless oil after chromatography on silica gel (20:1 to 15:1 hexane/EtOAc). $\mathbf{R_f}$ = 0.5 (hexane/EtOAc 5:1). ^{1}H NMR (400 MHz, $CDCl_3$) δ 7.05 (t, J = 8.0 Hz, 1H), 6.88 – 6.83 (m, 2H), 6.61 – 6.56 (m, 1H), 3.74 (s, 3H), 3.28 (td, J = 11.4, 4.0 Hz, 1H), 1.36 – 1.26 (m, 2H), 1.23 (s, 6H), 1.21 (s, 6H), 1.14 (dd, J = 15.1, 11.2 Hz, 1H), 1.00 (s, 6H), 0.99 (s, 6H), 0.93 (s, 6H), 0.91 (s, 6H). ^{13}C NMR (101 MHz, $CDCl_3$) δ 158.9, 150.5, 128.6, 120.2, 113.2, 111.7, 83.0, 82.7, 82.6, 55.1, 39.1, 24.9, 24.7, 24.6, 24.5, 24.4, 24.3. (the resonance of the carbon with borons attached was not observed). HR-MS (ESI) m/z: calcd for $C_{28}H_{47}B_3NaO_7$ ($[M+Na]^+$): 551.3507; Found 551.3511.

Applications

Triborylation of a large substrate

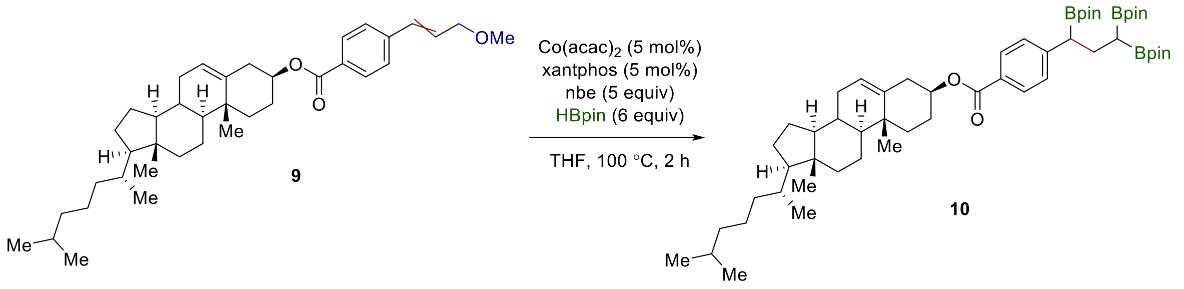

Supplementary Equation 4. Triborylation of compound **9**.

In an Ar-filled glovebox, a 20-mL screw-capped vial was charged with cobalt(II) acetylacetonate (3.9 mg, 15.0 μmol), xantphos (8.7 mg, 15.0 μmol), nbe (141.2 mg, 1.500 mmol), cinnamyl methyl ether (**9**, 0.300 mmol), THF (10 mL) and a magnetic stirring bar. The solution was stirred and pincolborane (261.2 μL, 1.800 mmol) was charged in the vial and sealed with a cap containing a PTFE septum. The vial was removed from the glovebox and stirred at 100 ˚c for 2 h, after which, the crude product was purified using silica gel flash column chromatography (column I.D. 13.4 mm) with gradient increase of ethyl acetate in hexanes as eluent. The conditions for chromatography and data for characterization of the products are given below.

Cholesteryl 4-(1,3,3-tris(4,4,5,5-tetramethyl-1,3,2-dioxaborolan-2-yl)propyl)benzoate (10)

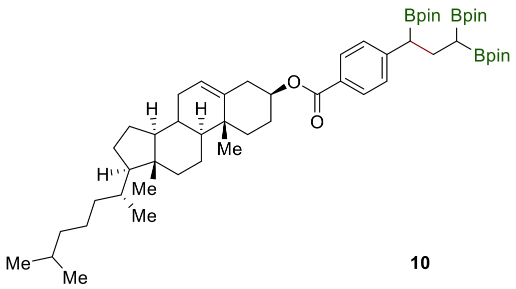

10

The title compound was isolated (125.7 mg, 0.138 mmol, 46%, 99:1) as a colourless oil after chromatography on silica gel (20:1 to 5:1 hexane/EtOAc). **R$_f$** = 0.4 (hexane/EtOAc 5:1).^{1}H NMR (500 MHz, $CDCl_3$) δ 7.88 (d, *J* = 8.3 Hz, 2H), 7.24 (d, *J* = 8.4 Hz, 2H), 5.40 (dd, *J* = 4.8, 1.8 Hz, 1H), 4.91 – 4.73 (m, 1H), 2.48 – 2.41 (m, 3H), 2.07 (dt, *J* = 13.7, 8.0 Hz, 1H), 2.03 – 1.96 (m, 3H), 1.94 – 1.88 (m, 2H), 1.86 – 1.80 (m, 1H), 1.75 – 1.67 (m, 3H), 1.61 – 1.44 (m, 8H), 1.37 – 1.31 (m, 3H), 1.21 (s, 12H), 1.20 (s, 6H), 1.19 (s, 6H), 1.18 (s, 6H), 1.16 (s, 6H), 1.14 – 1.09 (m, 4H), 1.06 (s, 3H), 1.03 – 0.97 (m, 3H), 0.92 (d, *J* = 6.5 Hz, 3H), 0.86 (d, *J* = 6.6 Hz, 3H), 0.86 (d, *J* = 6.6 Hz, 3H), 0.78 (t, *J* = 8.1 Hz, 1H), 0.68 (s, 3H). (Due to the complexity of the aliphatic part, we cannot guarantee the correct assignment of the peaks in the range of 1.0 to 2.2.) ^{13}C NMR (126 MHz, $CDCl_3$) δ 166.4, 149.3, 140.0, 129.7, 128.7, 127.8, 122.7, 83.5, 83.1, 83.1, 74.3, 56.9, 56.3, 50.2, 42.5, 39.9, 39.7, 38.4, 37.2, 36.8, 36.3, 35.9, 32.1, 32.0, 28.4, 28.2, 28.2, 28.1, 25.0, 25.0, 24.8, 24.8, 24.7, 24.4, 24.0, 23.0, 22.7, 21.2, 19.5, 18.9, 12.0. (the resonance of the carbon with borons attached was not observed). HR-MS (APCI) m/z: calcd for $C_{55}H_{90}B_3O_8$ ($[M+H]^+$): 911.6934; Found 911.6925.

Alkylation-Protodeborylation of 8a

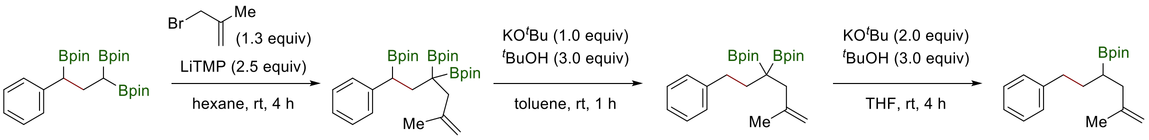

8a **11**, 52% **12**, 42% **13**, 76%

Supplementary Equation 5. Alkylation-protodeborylation of compound **8a**.

2,2',2''-(5-methyl-1-phenylhex-5-ene-1,3,3-triyl)tris(4,4,5,5-tetramethyl-1,3,2-dioxaborolane) (11)

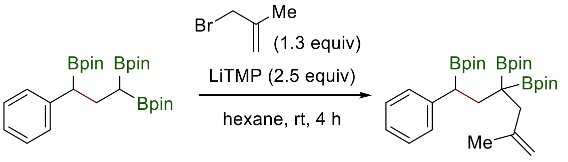

8a **11**, 52%

Supplementary Equation 6. Alkylation of compound **8a**.

To a solution of **8a** (99.6 mg, 0.200 mmol) in hexane (2 mL) was added LiTMP (73.5 mg, 0.500 mmol) portion-wise at 0 ˚c. 3-bromo-2-methylpropene (26.2 µL, 0.260 mmol) was added dropwise at the same temperature. The reaction was stirred at room temperature for 4h, after which, the solvent was removed under reduce pressure. **11** was isolated (57.4 mg, 0.104 mmol, 52%) was purified and isolated as a colourless oil using flash chromatography on silica gel (20:1 to 15:1 hexane/EtOAc). $\mathbf{R_f}$ = 0.55 (hexane/EtOAc 5:1). ^{1}H NMR (400 MHz, $CDCl_3$) δ 7.22 (dd, *J* = 8.3, 1.3 Hz, 2H), 7.15 (t, *J* = 7.6 Hz, 2H), 7.03 (t, *J* = 7.2 Hz, 1H), 4.69 (s, 1H), 4.65 (s, 1H), 2.41 – 2.17 (m, 5H), 1.68 (s, 3H), 1.21 (s, 6H), 1.21 (s, 6H), 1.11 (s, 6H), 1.10 (s, 12H), 1.09 (s, 6H). ^{13}C NMR (126 MHz, $CDCl_3$) δ 145.3, 144.6, 128.8, 127.9, 124.6, 110.1, 83.0, 83.0, 82.9, 36.9, 31.0, 24.9, 24.8, 24.8, 24.6, 24.4, 24.3 (the resonance of the carbon with borons attached was not observed). GC-MS (ESI) m/z: calcd for $C_{31}H_{51}B_3O_6$ ($[M-CH_3]^+$): 537.40; Found 537.40.

2,2'-(5-methyl-1-phenylhex-5-ene-3,3-diyl)bis(4,4,5,5-tetramethyl-1,3,2-dioxaborolane) (12)

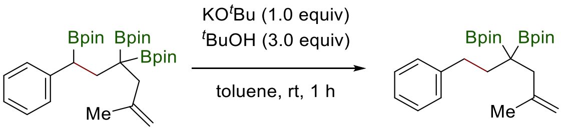

11 **12**, 42%

Supplementary Equation 7. Protodeborylation of compound **11**.

To a solution of **11** (55.2 mg, 0.100 mmol) in toluene (1 mL) was added KO^{t}Bu (11.2 mg, 0.100 mmol) portion-wise at 0 ˚c. ^{t}BuOH (29.6 µL, 0.300 mmol) was added at the same temperature. The reaction was stirred at room temperature for 1h, after which, the solvent was removed under reduce pressure. **12** was isolated (19.5 mg, 0.042 mmol, 42%) was purified and isolated as a colourless oil using flash chromatography on silica gel (100:0 to 20:1 hexane/EtOAc). $\mathbf{R_f}$ = 0.6 (hexane/EtOAc 10:1). ^{1}H NMR (400 MHz, $CDCl_3$) δ 7.22 (t, *J* = 7.3 Hz, 2H), 7.17 – 7.09 (m, 3H), 4.82 (s, 1H), 4.79 (s, 1H), 2.56 – 2.48 (m, 4H), 1.90 – 1.83 (m, 2H), 1.72 (s, 3H), 1.25 (s, 24H). ^{13}C NMR (101 MHz, $CDCl_3$) δ 145.2, 144.0, 128.7, 128.2, 125.5, 112.0, 83.4, 37.4, 34.0, 32.3, 25.1, 24.9, 23.4 (the resonance of the carbon with

bisboron attached was not observed). HR-MS (ESI) m/z: calcd for $C_{25}H_{40}B_2NaO_4$ ($[M+Na]^+$): 449.3013; Found 449.3016.

4,4,5,5-tetramethyl-2-(5-methyl-1-phenylhex-5-en-3-yl)-1,3,2-dioxaborolane (13)

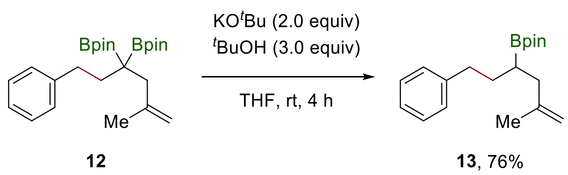

Supplementary Equation 8. Protodeborylation of compound **12**.

To a solution of **12** (42.6 mg, 0.100 mmol) in THF (1 mL) was added KO^{t}Bu (22.4 mg, 0.200 mmol) portion-wise at 0 ˚c. ^{t}BuOH (29.6 µL, 0.300 mmol) was added at the same temperature. The reaction was stirred at room temperature for 4h, after which, the solvent was removed under reduce pressure. **13** was isolated (22.8 mg, 0.076 mmol, 76%) was purified and isolated as a colourless oil using flash chromatography on silica gel (100:0 to 100:1 hexane/EtOAc). $\mathbf{R_f}$ = 0.7 (hexane/EtOAc 10:1). ^{1}H NMR (400 MHz, CDCl$_3$) δ 7.28 – 7.24 (m, 2H), 7.20 – 7.14 (m, 3H), 4.70 (d, *J* = 1.1 Hz, 1H), 4.70 (d, *J* = 1.1 Hz, 1H), 2.71 – 2.53 (m, 2H), 2.20 (dd, *J* = 14.3, 8.6 Hz, 1H), 2.10 (dd, *J* = 14.3, 7.3 Hz, 1H), 1.73 – 1.65 (m, 5H), 1.26 (s, 12H), 1.24 – 1.22 (m, 1H). ^{13}C NMR (101 MHz, CDCl$_3$) δ 145.4, 143.0, 128.4, 128.2, 125.6, 110.6, 83.1, 39.3, 35.6, 33.1, 24.9, 24.8, 22.4 (the resonance of the carbon with boron attached was not observed). HR-MS (ESI) m/z: calcd for $C_{19}H_{28}BO_2$ ($[M-H]^+$): 299.2182; Found 299.2185.

Construction of three different carbon-carbon bonds

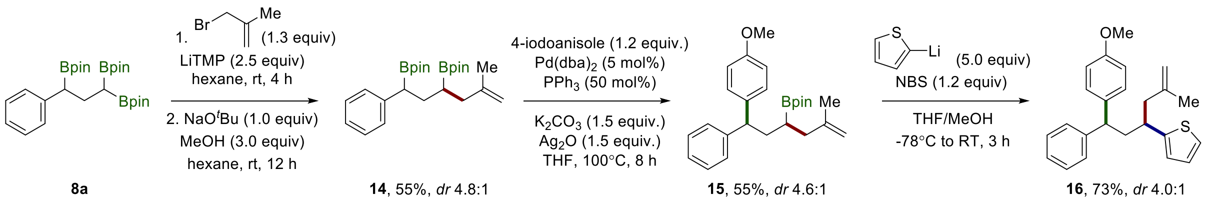

Supplementary Equation 9. Construction of three different carbon-carbon bonds from **8a**.

2,2'-(5-methyl-1-phenylhex-5-ene-1,3-diyl)bis(4,4,5,5-tetramethyl-1,3,2-dioxaborolane) (14)

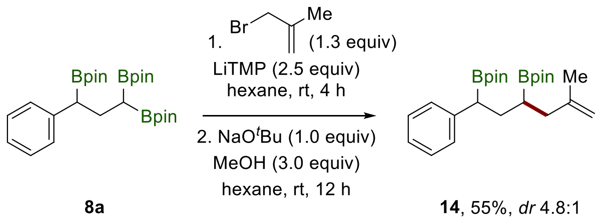

Supplementary Equation 10. Alkylation-protodeborylation of compound **8a**.

To a solution of **8a** (99.6 mg, 0.200 mmol) in hexane (2 mL) was added LiTMP (73.5 mg, 0.500 mmol) portion-wise at 0 ˚c. 3-bromo-2-methylpropene (26.2 µL, 0.260 mmol) was added dropwise at the same temperature. The reaction was stirred at room temperature for 4h, after which, the solvent was removed under reduce pressure. The crude product **11** was purified and isolated as a colourless oil using silica gel flash column chromatography with hexane/EA as eluent and used for the next step. To

a solution of **11** in hexane (1 mL) was added NaO^{t}Bu (19.2 mg, 0.200 mmol) portion-wise at 0 ˚c. MeOH (8.0 µL, 0.600 mmol) was added at the same temperature. The reaction was stirred at room temperature for 12h, after which, the solvent was removed under reduce pressure. **14** was purified and isolated (46.9 mg, 0.055 mmol, 55%, *dr* 4.8:1) as a colourless oil using flash chromatography on silica gel (100:0 to 20:1 hexane/EtOAc). **R$_f$** = 0.6 (hexane/EtOAc 10:1). ^{1}H NMR (400 MHz, $CDCl_3$) δ 7.25 – 7.17 (m, 4H), 7.12 – 7.07 (m, 1H), 4.68 (s, 1H), 4.67 (s, 1H), 2.39 (dd, *J* = 9.9, 6.4 Hz, 1H), 2.17 – 1.98 (m, 2H), 1.88 (ddd, *J* = 13.0, 10.0, 6.3 Hz, 1H), 1.73 – 1.67 (m, 4H), 1.21 (s, 12H), 1.20 (s, 6H), 1.17 (s, 6H), 0.91 – 0.82 (m, 1H). ^{13}C NMR (101 MHz, $CDCl_3$) δ 145.6, 143.7, 128.4, 128.2, 125.0, 110.5, 83.2, 82.9, 39.6, 34.1, 24.9, 24.8, 24.6, 22.3 (the resonance of the carbon with borons attached was not observed). HR-MS (ESI) m/z: calcd for $C_{25}H_{40}B_2NaO_4$ ([M+Na]$^+$): 449.3013; Found 449.3014. Diastereoselectivity is confirmed by ^{1}H NMR.

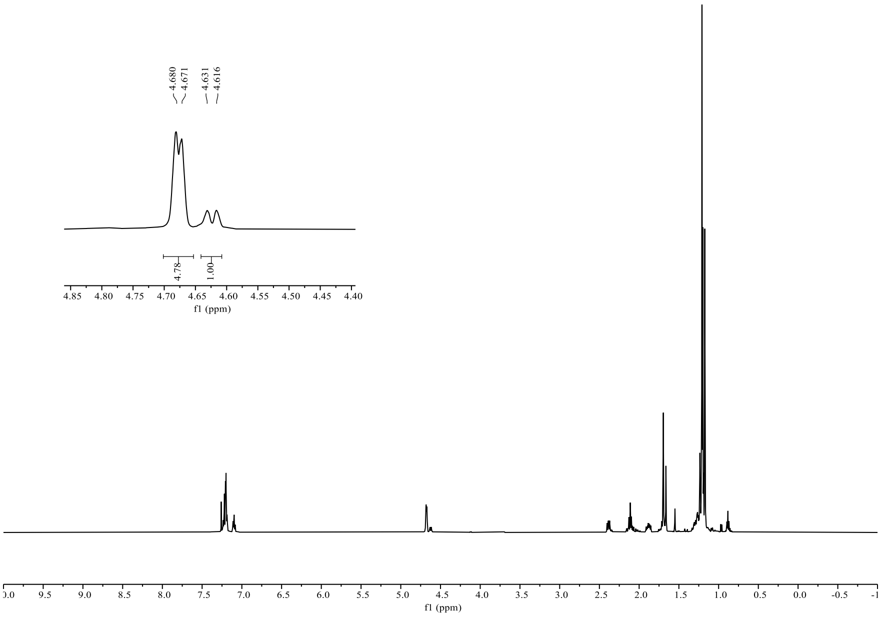

Supplementary Figure 3. ^{1}H NMR spectrum of compound **14**.

The configuration is derived through oxidation of **14** to 1,3-diol **14-ox** and checked with literature.[9]

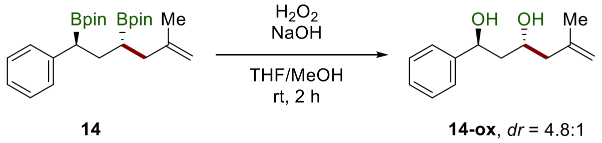

Supplementary Equation 11. Oxidation of compound **14**.

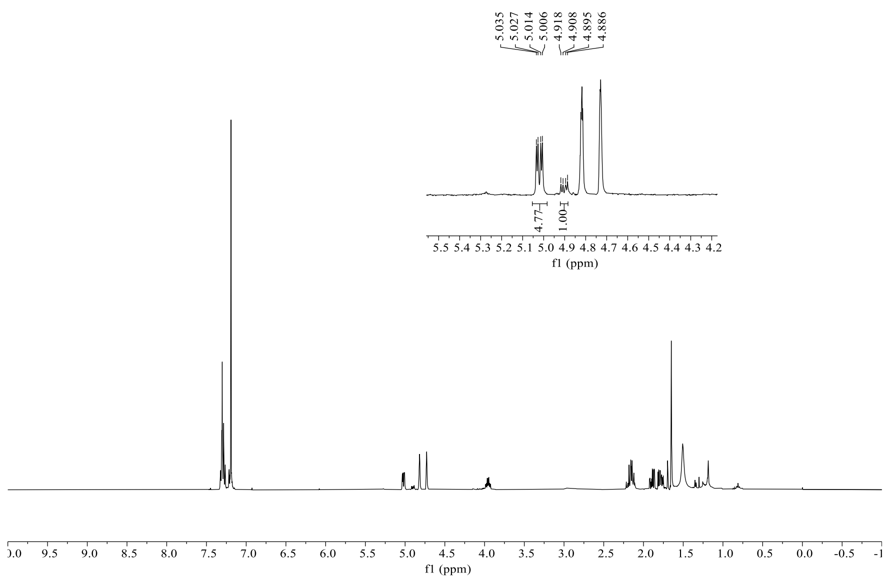

Supplementary Figure 4. ^{1}H NMR spectrum of compound **14-ox**.

2-(1-(4-methoxyphenyl)-5-methyl-1-phenylhex-5-en-3-yl)-4,4,5,5-tetramethyl-1,3,2-dioxaborolane (15)

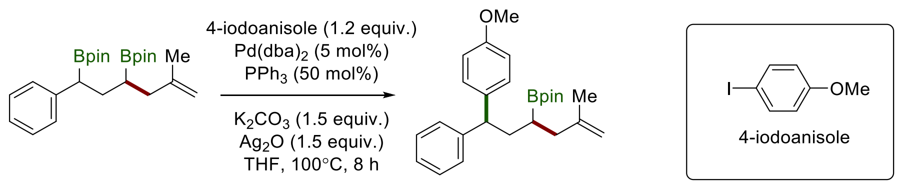

14 **15**, 55%, *dr* 4.6:1

Supplementary Equation 12. Suzuki-Miyaura coupling of compound **14**.

In an Ar-filled glovebox, a 4-mL screw-capped vial was charged with **14** (42.6 mg, 0.100 mmol), $Pd(dba)_2$ (2.9 mg, 5.00 μmol), PPh_3 (13.1 mg, 50.0 μmol), K_2CO_3 (20.7 mg, 0.150 mmol), Ag_2O (34.8 mg, 0.150 mmol), 4-iodoanisole (28.1 mg, 0.120 mmol), THF (1 mL) and a magnetic stirring bar. The solution was sealed with a cap containing a PTFE septum. The vial was removed from the glovebox and stirred at 100 ˚c for 8 h, after which, the crude product was purified using silica gel flash column chromatography. **15** was isolated (22.3 mg, 0.055 mmol, 55%, *dr* 4.6:1) was purified and isolated as a colourless oil using flash chromatography on silica gel (100:0 to 50:1 hexane/EtOAc). $\mathbf{R_f}$ = 0.7 (hexane/EtOAc 10:1). ^{1}H NMR (500 MHz, $CDCl_3$) δ 7.26 – 7.19 (m, 4H), 7.17 – 7.11 (m, 3H), 6.82 (d, *J* = 8.7 Hz, 2H), 4.69 (s, 1H), 4.65 (s, 1H), 3.95 (dd, *J* = 8.8, 6.9 Hz, 1H), 3.77 (s, 3H), 2.16 (dd, *J* = 14.1, 7.7 Hz, 1H), 2.09 – 2.02 (m, 3H), 1.57 (s, 3H), 1.24 (s, 12H), 1.15 – 1.09 (m, 1H). ^{13}C NMR (126 MHz, $CDCl_3$) δ 158.0, 146.3, 145.4, 136.9, 129.2, 128.4, 127.8, 125.9, 113.9, 111.0, 83.1, 55.3, 49.8, 39.7, 37.2, 25.1, 25.0, 22.3. (the resonance of the carbon with bisboron attached was not observed). GC-MS (EI) m/z: calcd for $C_{26}H_{35}BO_3$ ($[M]^+$): 426.27; Found 426.27. Diastereoselectivity is confirmed by ^{1}H NMR.

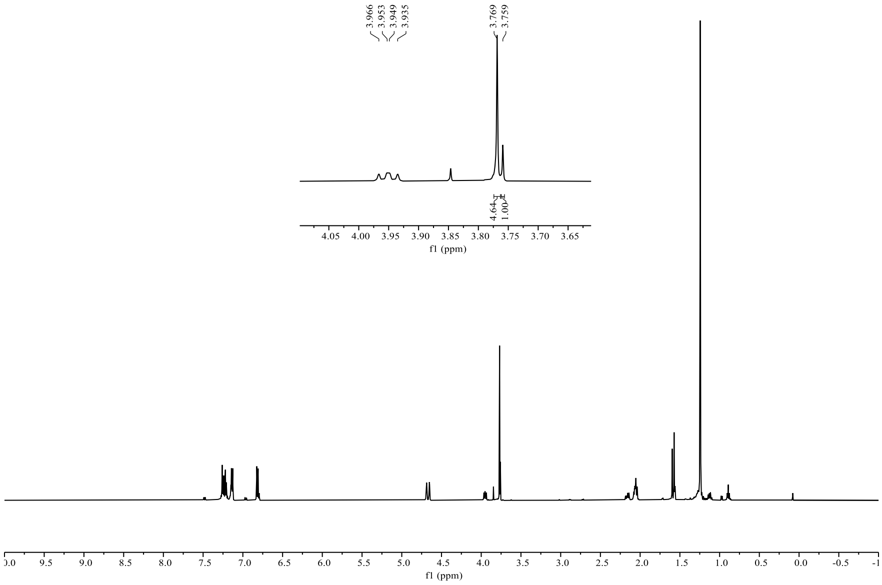

Supplementary Figure 5. ^{1}H NMR spectrum of compound **15**.

2-(1-(4-methoxyphenyl)-5-methyl-1-phenylhex-5-en-3-yl)thiophene (16) [10]

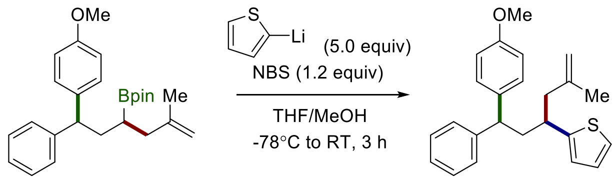

Supplementary Equation 13. Thienylation of compound **15**.

To a solution of **15** (40.6 mg, 0.100 mmol) in THF (1 mL) was added 2-thienyllithium (0.5 mL, 1 M in THF/Hexene, 0.500 mmol) dropwise at -78 ˚c and stirred for 2 h before the solution was warmed to room temperature. N-Bromosuccinimide (NBS, 20.9 mg, 0.120 mmol, 0.1 M in MeOH) was added dropwise. The reaction was stirred at room temperature for another 1 h, after which, a saturated solution of $Na_2S_2O_3$ (2 mL) was added and the mixture was diluted with Et_2O (10 mL). The organic layer was separated and the aqueous layer was extracted with Et_2O. The combined organic layers were washed with brine and dried over Na_2SO_4. The solvent was removed under reduce pressure. **16** was isolated (26.4 mg, 0.073 mmol, 73%, *dr* 4.0:1) was purified and isolated as a yellowish oil using flash chromatography on silica gel (100:0 to 100:1 hexane/EtOAc). **R$_f$** = 0.8 (hexane/EtOAc 10:1). ^{1}H NMR (400 MHz, $CDCl_3$) δ 7.30 (t, *J* = 7.4 Hz, 1H), 7.23 – 7.16 (m, 3H), 7.14 – 7.10 (m, 4H), 6.93 (dd, *J* = 5.1, 3.4 Hz, 1H), 6.85 (d, *J* = 8.7 Hz, 2H), 6.70 (dd, *J* = 3.4, 1.3 Hz, 1H), 4.72 (s, 1H), 4.62 (s, 1H), 3.80 (s, 3H), 3.78 – 3.75 (m, 1H), 2.98 – 2.88 (m, 1H), 2.50 (ddd, *J* = 13.8, 11.5, 3.7 Hz, 1H), 2.41 – 2.29 (m, 2H), 2.10 (ddd, *J* = 13.8, 10.8, 4.2 Hz, 1H), 1.54 (s, 3H). ^{13}C NMR (101 MHz, $CDCl_3$) δ 158.2, 149.5, 146.0, 143.5, 135.7, 129.4, 128.5, 127.6, 126.5, 126.0, 124.3, 123.1, 114.0, 112.8, 55.4, 47.7, 47.6, 43.2, 37.0, 22.2. GC-MS (EI) m/z: calcd for $C_{24}H_{27}OS$ ([M+H]$^+$): 363.1777; Found 363.1775. Diastereoselectivity is confirmed by ^{1}H NMR.

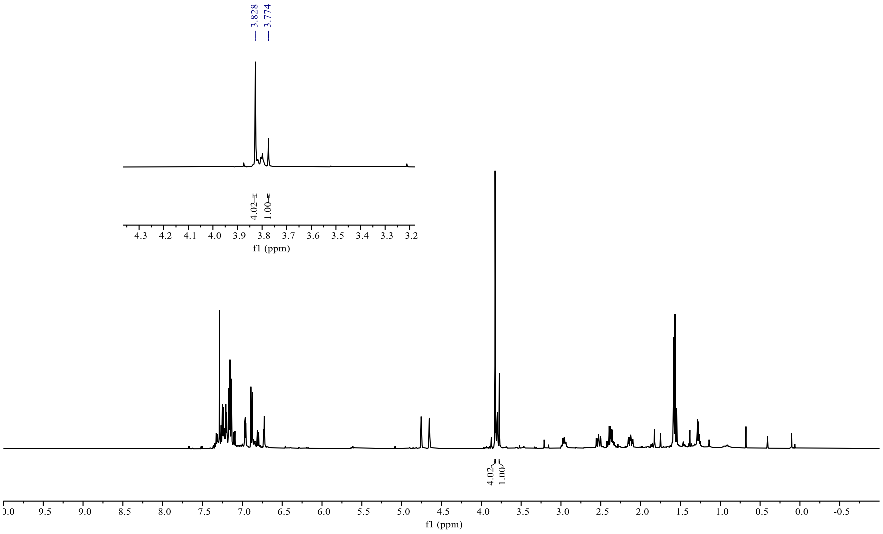

Supplementary Figure 6. ^{1}H NMR spectrum of compound **16**.

Model for the protodeborylation

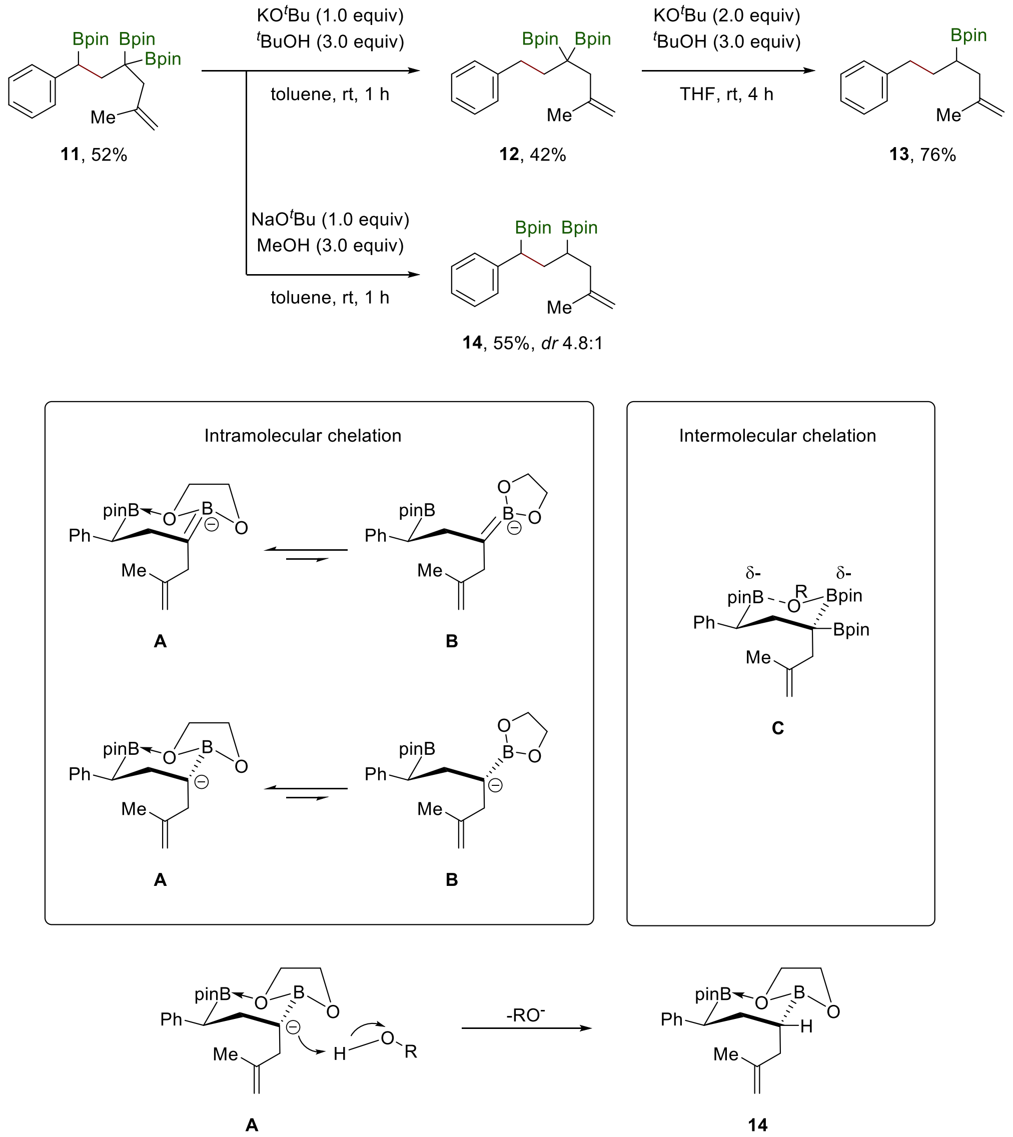

Supplementary Figure 7. Model for the protodeborylation.

In using sodium *tert*-butoxide accompanied by MeOH as the protic source, **11** underwent proto-deborylation at the *gem*-bisboron containing carbon center to form 1,3-bisboron compound **14** in an *anti*-diastereoselective fashion. This diastereoselectivity can be explained by the proposed carbanion model in the presences of Bronsted base. The chelated model **A**, with the phenyl-substituent on the equatorial position *anti* to the substituent on the carbanion minimizes steric strain and allows approach from small electrophile to form *anti*-diastereoselective **14**.

Procedures for Deuterium-labeling Experiments

2a-D[12] and DBpin[13] was synthesized based on known literature methods.

Deuterium-labeling experiment (1)

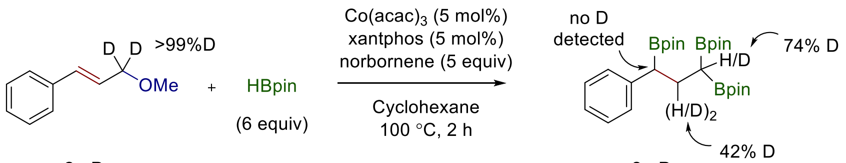

2a-D **8a-D**

Supplementary Equation 14. Reaction of **2a-D** with HBpin.

In an Ar-filled glovebox, a 20-mL screw-capped vial was charged with cobalt(III) acetylacetonate (5.3 mg, 15.0 μmol), xantphos (8.7 mg, 15.0 μmol), nbe (141.2 mg, 1.500 mmol), **2a-D** (0.300 mmol), cyclohexane (10 mL) and a magnetic stirring bar. The solution was stirred and pincolborane (261.2 μL, 1.800 mmol) was charged in the vial and sealed with a cap containing a PTFE septum. The vial was removed from the glovebox and stirred at 100 ˚c for 2 h, after which, the crude product was purified using silica gel flash column chromatography (column I.D. 13.4 mm) with hexane/EtOAc. Next, chloroform-d_1 (99.8 atom % D, 2 equivalent) was added and dissolved in chloroform (0.6 mL) for ^{2}H NMR analysis. The sample was then dried for ^{1}H NMR and quantitative ^{13}C NMR analysis.

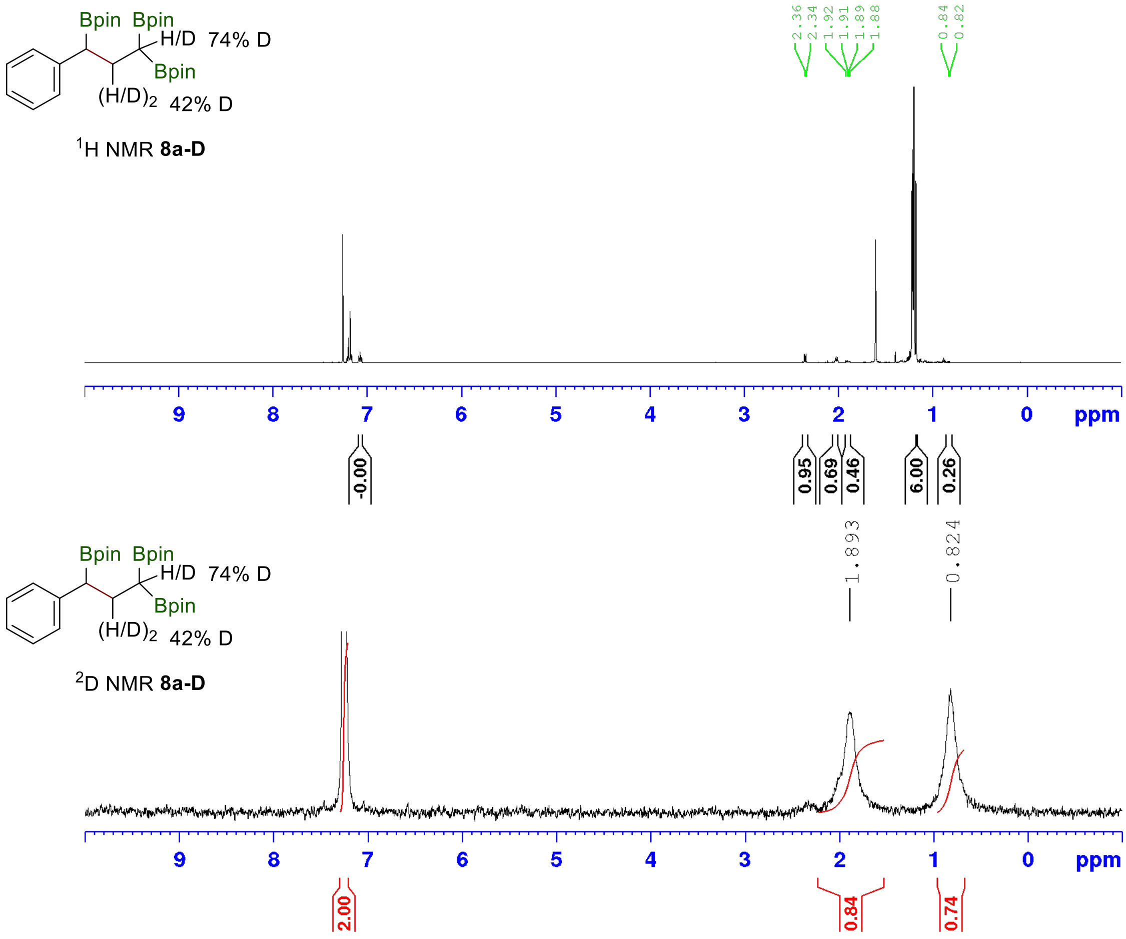

Supplementary Figure 8. ^{1}H and ^{2}D NMR of the product from the reaction of **2a-D** with HBpin.

Deuterium-labeling experiment (2)

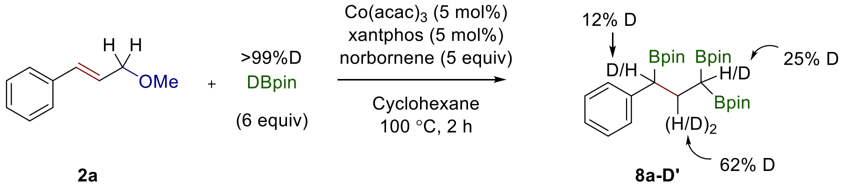

Supplementary Equation 15. Reaction of **2a** with DBpin.

In an Ar-filled glovebox, a 20-mL screw-capped vial was charged with cobalt(III) acetylacetonate (5.3 mg, 15.0 μmol), xantphos (8.7 mg, 15.0 μmol), nbe (141.2 mg, 1.500 mmol), **2a** (0.300 mmol), cyclohexane (10 mL) and a magnetic stirring bar. The solution was stirred and DBpin (1.800 mmol) was charged in the vial and sealed with a cap containing a PTFE septum. The vial was removed from the glovebox and stirred at 100 ˚c for 2 h, after which, the crude product was purified using silica gel flash column chromatography (column I.D. 13.4 mm) with hexane/EtOAc (100:1 to 20:1). Next, chloroform-*d*$_1$ (99.8 atom % D, 1 equivalent) was added and dissolved in chloroform (0.6 mL) for ^{2}H NMR analysis. The sample was then dried for ^{1}H NMR and quantitative ^{13}C NMR analysis.

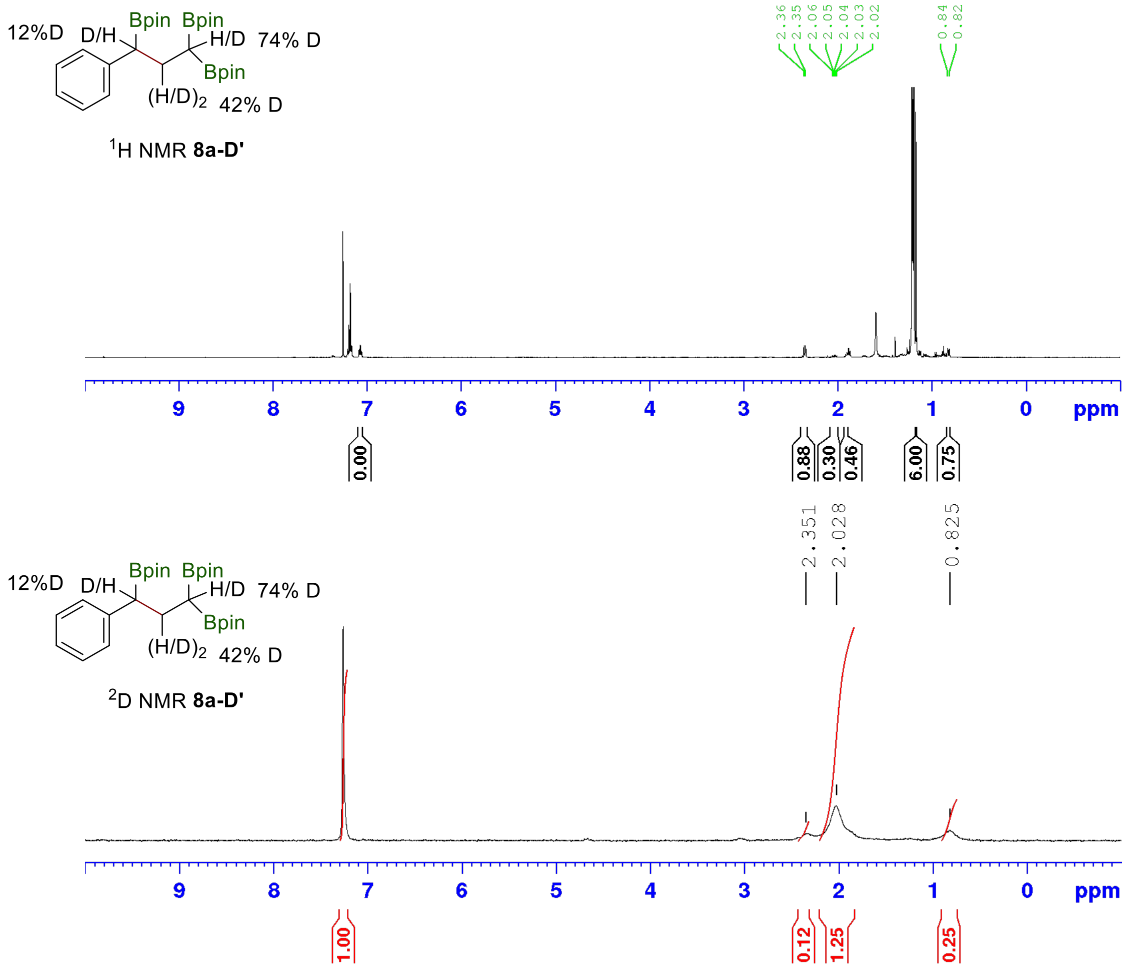

Supplementary Figure 9. ^{1}H and ^{2}D NMR of the product from the reaction of **2a** with DBpin.

Procedures for Stepwise Reactions

4,4,5,5-tetramethyl-2-(1-phenylallyl)-1,3,2-dioxaborolane (17, a mixture with PhBpin)

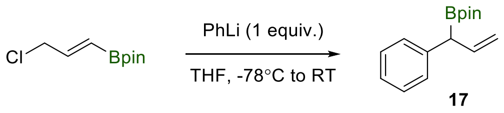

Supplementary Equation 16. Preparation of compound **17**.

The title compound was synthesized according to the known procedures.[14]

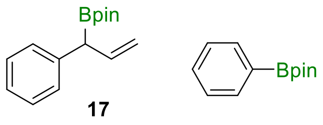

The title compound was isolated as a colorless oil after chromatography on silica gel (100:1 hexane/EtOAc). ^{1}H NMR (500 MHz, $CDCl_3$) δ 7.77 (dd, *J* = 8.0, 1.4 Hz, 1.3H, PhBpin), 7.43 – 7.38 (m, 0.7H, PhBpin), 7.32 (t, *J* = 7.2 Hz, 1.4H, PhBpin), 7.23 – 7.17 (m, 4H), 7.11 (t, *J* = 7.0 Hz, 1H), 6.07 (ddd, *J* = 16.9, 10.3, 8.1 Hz, 1H), 5.02 – 4.98 (m, 1H), 4.98 – 4.94 (m, 1H), 3.20 (d, *J* = 8.1 Hz, 1H), 1.30 (s, 9H, PhBpin), 1.18 (s, 6H), 1.18 (s, 6H). ^{13}C NMR (101 MHz, $CDCl_3$) δ 141.3, 138.9, 134.9 (PhBpin), 131.4 (PhBpin), 128.6, 128.6, 127.8 (PhBpin), 125.7, 114.6, 83.9 (PhBpin), 83.7, 25.0 (PhBpin), 24.7. (the resonance of the carbon with boron attached was not observed). GC-MS (EI) m/z: calcd for $C_{15}H_{21}BO_2$ ($[M]^+$): 244.16; Found 244.16.

gem-Diborylation reaction of 17 with HBpin

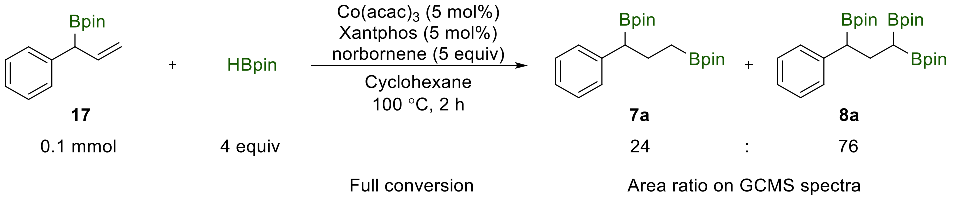

Supplementary Equation 17. Reaction of **17** with HBpin.

In an Ar-filled glovebox, a 10-mL screw-capped vial was charged with cobalt(III) acetylacetonate (1.8 mg, 5.0 μmol), xantphos (2.9 mg, 5.0 μmol), nbe (47.1 mg, 0.500 mmol), **17** (0.100 mmol), cyclohexane (3 mL) and a magnetic stirring bar. The solution was stirred and HBpin (1.200 mmol) was charged in the vial and sealed with a cap containing a PTFE septum. The vial was removed from the glovebox and stirred at 100 ˚c for 2 h, after which, the crude product was determined by GCMS with internal standard to give a ratio of 24:76 (**7a**:**8a**).

(*E*)-4,4,5,5-tetramethyl-2-(3-phenylbut-1-en-1-yl)-1,3,2-dioxaborolane (19)

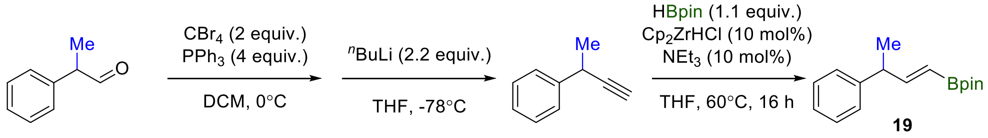

Supplementary Equation 18. Preparation of compound **19**.

The title compound was synthesized according to the known procedures.[15, 16]

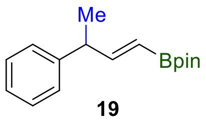

19

The title compound was isolated (35%, total yield of two steps) as a colourless oil after chromatography on silica gel (100:1 hexane/EtOAc). ^{1}H NMR (500 MHz, $CDCl_3$) δ 7.33 – 7.27 (m, 2H), 7.20 (d, *J* = 7.3 Hz, 3H), 6.78 (dd, *J* = 18.0, 6.3 Hz, 1H), 5.43 (dd, *J* = 18.0, 1.6 Hz, 1H), 3.53 (p, *J* = 7.1, 6.4 Hz, 1H), 1.39 (d, *J* = 7.1 Hz, 3H), 1.25 (s, 12H). ^{13}C NMR (126 MHz, $CDCl_3$) δ 158.0, 144.8, 128.6, 128.2, 127.6, 126.4, 83.2, 45.2, 24.9, 24.9, 20.4. (the resonance of the carbon with boron attached was not observed). GC-MS (EI) m/z: calcd for $C_{16}H_{22}BO_2$ ($[M]^+$): 257.17; Found 257.17.

Reactions of 19 with HBpin

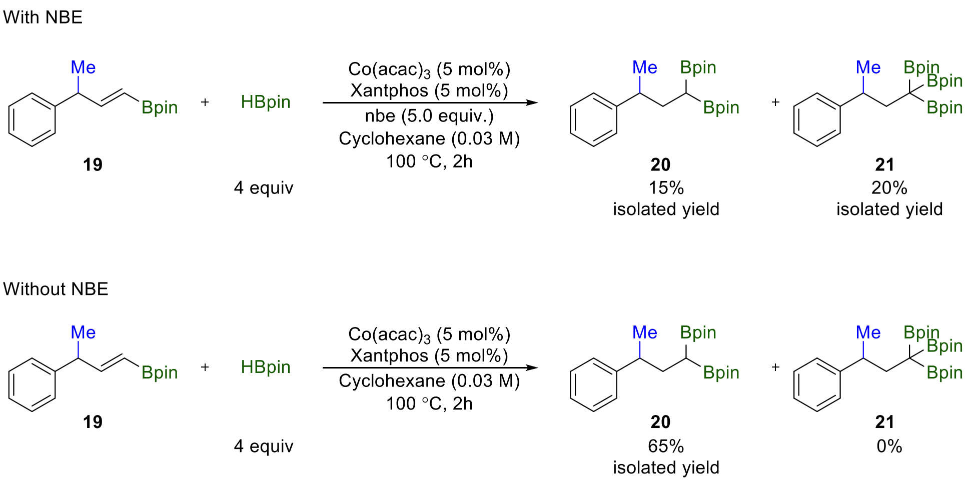

Supplementary Equation 19. Reaction of **19** with HBpin.

2,2'-(3-phenylbutane-1,1-diyl)bis(4,4,5,5-tetramethyl-1,3,2-dioxaborolane) (20) [17]

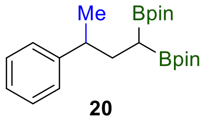

20

The title compound was isolated (17.6 mg, 15%) as a colourless oil after chromatography on silica gel (100:1 to 30:1 hexane/EtOAc). ^{1}H NMR (500 MHz, $CDCl_3$) δ 7.26 (d, *J* = 7.5 Hz, 2H), 7.20 – 7.14 (m, 3H), 2.71 – 2.63 (m, 1H), 1.84 (t, *J* = 7.8 Hz, 2H), 1.29 (d, *J* = 8.0 Hz, 3H), 1.27 – 1.26 (m, 12H), 1.22 (s, 12H), 0.74 (t, *J* = 7.8 Hz, 1H). ^{13}C NMR (126 MHz, $CDCl_3$) δ 147.8, 128.3, 127.3, 125.8, 83.1, 83.0, 42.0, 34.6, 25.0, 25.0, 24.7, 24.7, 21.8. (the resonance of the carbon with boron attached was not observed). GC-MS (EI) m/z: calcd for $C_{22}H_{35}B_2O_4$ ($[M]^+$): 385.27; Found 385.27.

2,2',2''-(3-phenylbutane-1,1,1-triyl)tris(4,4,5,5-tetramethyl-1,3,2-dioxaborolane) (21)

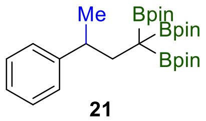

21

The title compound was isolated (30.6 mg, 20%) as a white solid after chromatography on silica gel (100:1 to 20:1 hexane/EtOAc). ^{1}H NMR (500 MHz, $CDCl_3$) δ 7.26 – 7.19 (m, 4H), 7.12 (tt, *J* = 7.2, 1.8 Hz, 1H), 3.06 – 2.97 (m, 1H), 2.13 (dd, *J* = 13.8, 5.3 Hz, 1H), 1.96 (dd, *J* = 13.8, 9.8 Hz, 1H), 1.20 (s, 18H), 1.99 – 1.84 (m, 21H). ^{13}C NMR (126 MHz, $CDCl_3$) δ 149.8, 128.1, 127.6, 125.4, 82.9, 40.6, 36.9, 24.9, 24.8, 21.6. (the resonance of the carbon with boron attached was not observed). GC-MS (EI) m/z: calcd for $C_{28}H_{46}B_3O_6$ ($[M]^+$): 511.36; Found 511.36.

11B NMR of crude products

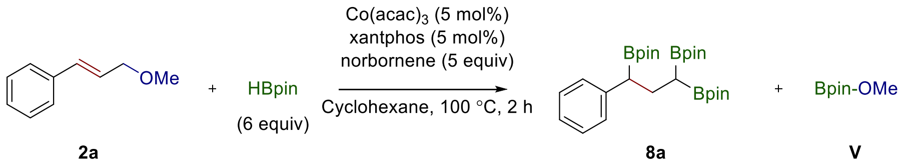

Supplementary Equation 20. Reaction of **2a** with HBpin.

In an Ar-filled glovebox, a 20-mL screw-capped vial was charged with cobalt(III) acetylacetonate (5.3 mg, 15.0 μmol), xantphos (8.7 mg, 15.0 μmol), nbe (141.2 mg, 1.500 mmol), **2a** (0.300 mmol), cyclohexane (10 mL) and a magnetic stirring bar. The solution was stirred and HBpin (1.800 mmol) was charged in the vial and sealed with a cap containing a PTFE septum. The vial was removed from the glovebox and stirred at 100 ˚c for 2 h, after which, the crude product was dissolved in chloroform-d_1 (0.6 mL) for ^{11}B NMR analysis. The chemical shift of Bpin-OMe (**V**, 22.3 ppm) is matched with the known literature. [18]

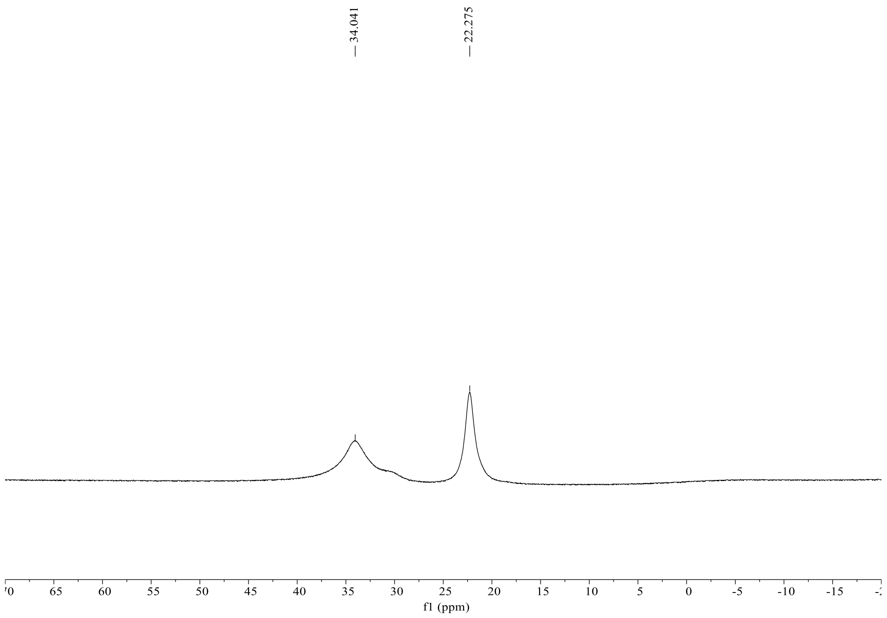

Supplementary Figure 10. ^{11}B NMR of the product from the reaction of **2a** with HBpin.

Reactions of 6 with HBpin

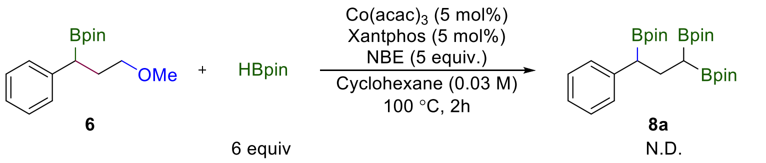

Supplementary Equation 21. Reaction of **6** with HBpin.

Compound **6** was treated under standard conditions, which resulted in no conversion of **6**.

Details of Single-Crystal X-ray Diffraction Analysis of 8g

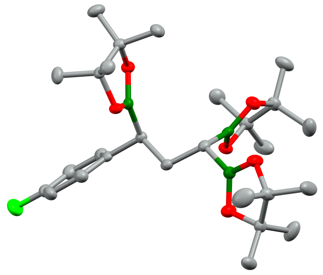

X-ray structure of **8g**

Supplementary Figure 11. X-ray structure of **8g**.

A specimen of $C_{27}H_{44}B_3FO_6$, approximate dimensions 0.524 mm x 0.559 mm x 0.637 mm, was used for the X-ray crystallographic analysis. The X-ray intensity data were measured (λ = 0.71073 Å).

The total exposure time was 3.05 hours. The frames were integrated with the Bruker SAINT software package using a narrow-frame algorithm. The integration of the data using a monoclinic unit cell yielded a total of 51449 reflections to a maximum θ angle of 28.35° (0.75 Å resolution), of which 7443 were independent (average redundancy 6.912, completeness = 99.8%, R_{int} = 3.95%, R_{sig} = 2.83%) and 6387 (85.81%) were greater than 2σ(F^2). The final cell constants of a = 11.8659 Å, b = 9.2712 Å, c = 27.7298 Å, α = 90.000°, β = 101.114°, γ = 90.000°, volume = 2993.4 Å^3, are based upon the refinement of the XYZ-centroids of 74 reflections above 20 σ(I) with 7.595° < 2θ < 36.67°. Data were corrected for absorption effects using the Multi-Scan method (SADABS). The ratio of minimum to maximum apparent transmission was 0.918.

The structure was solved and refined using the Bruker SHELXTL Software Package, with Z = 4 for the formula unit, $C_{27}H_{44}B_3FO_6$. The final anisotropic full-matrix least-squares refinement on F^2 with 405 variables converged at R1 = 6.28%, for the observed data and wR2 = 15.94% for all data. The goodness-of-fit was 1.093. The largest peak in the final difference electron density synthesis was 0.523 e$^-$/Å^3 and the largest hole was -0.483 e$^-$/Å^3 with an RMS deviation of 0.053 e$^-$/Å^3. On the basis of the final model, the calculated density was 1.149 g/cm^3 and F(000), 1112 e$^-$.

NMR Spectra

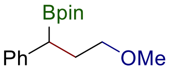

6

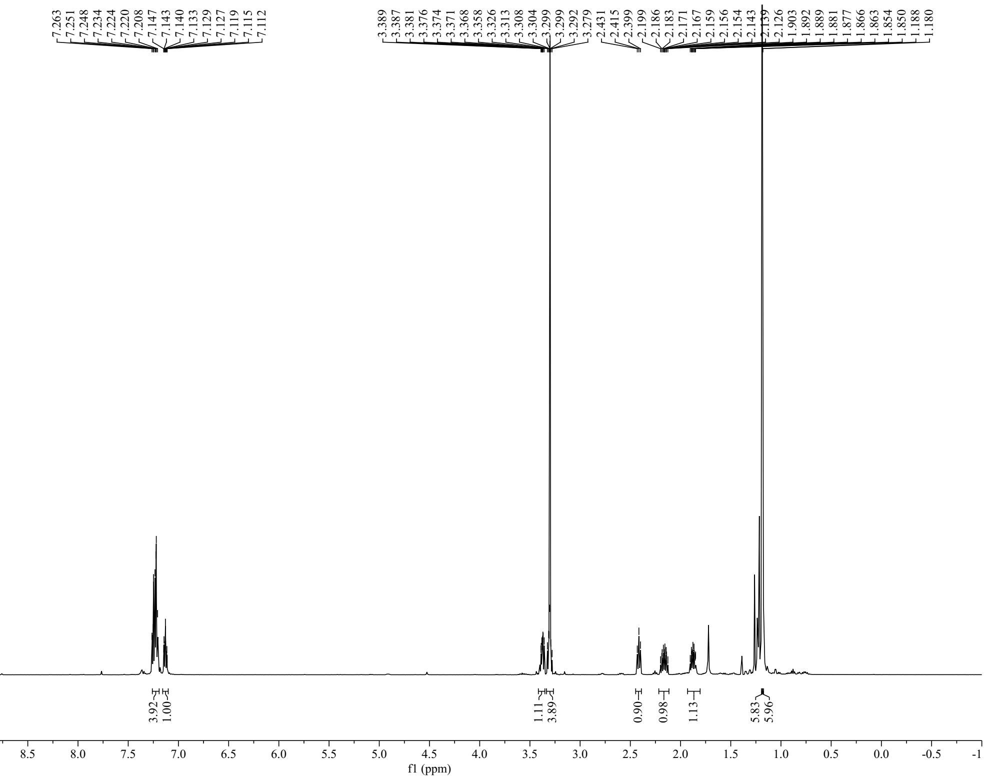

Supplementary Figure 12. ^{1}H NMR spectrum of compound **6**.

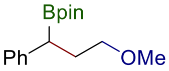

6

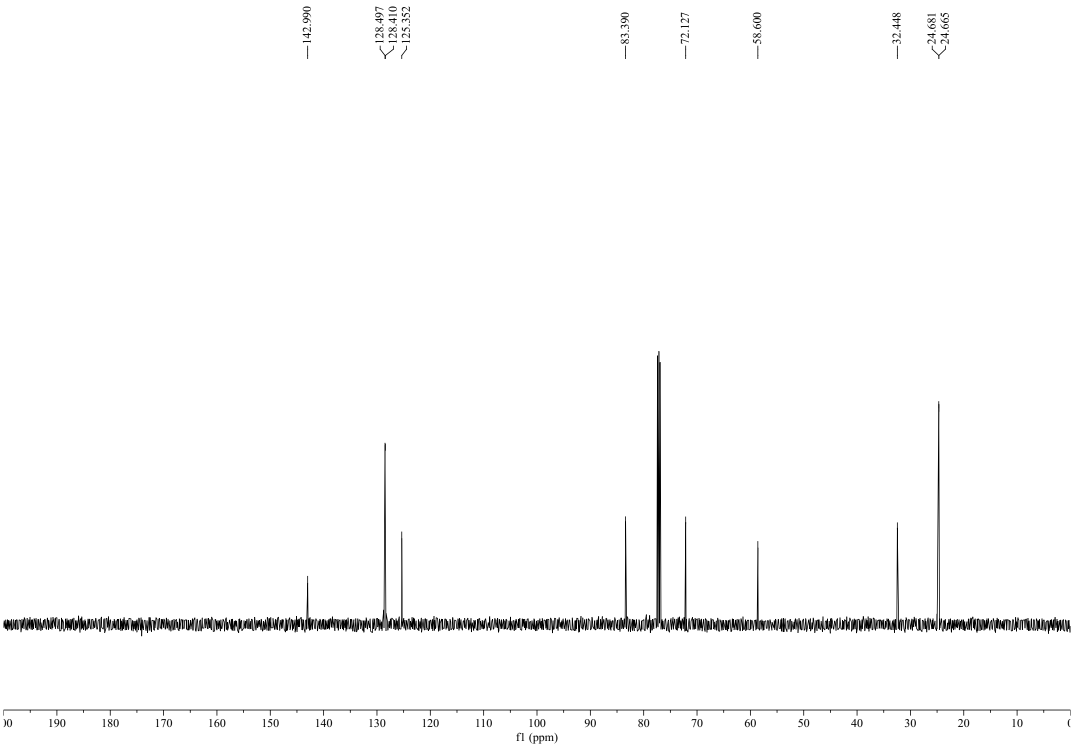

Supplementary Figure 13. $^{13}C\{^{1}H\}$ NMR spectrum of compound **6**.

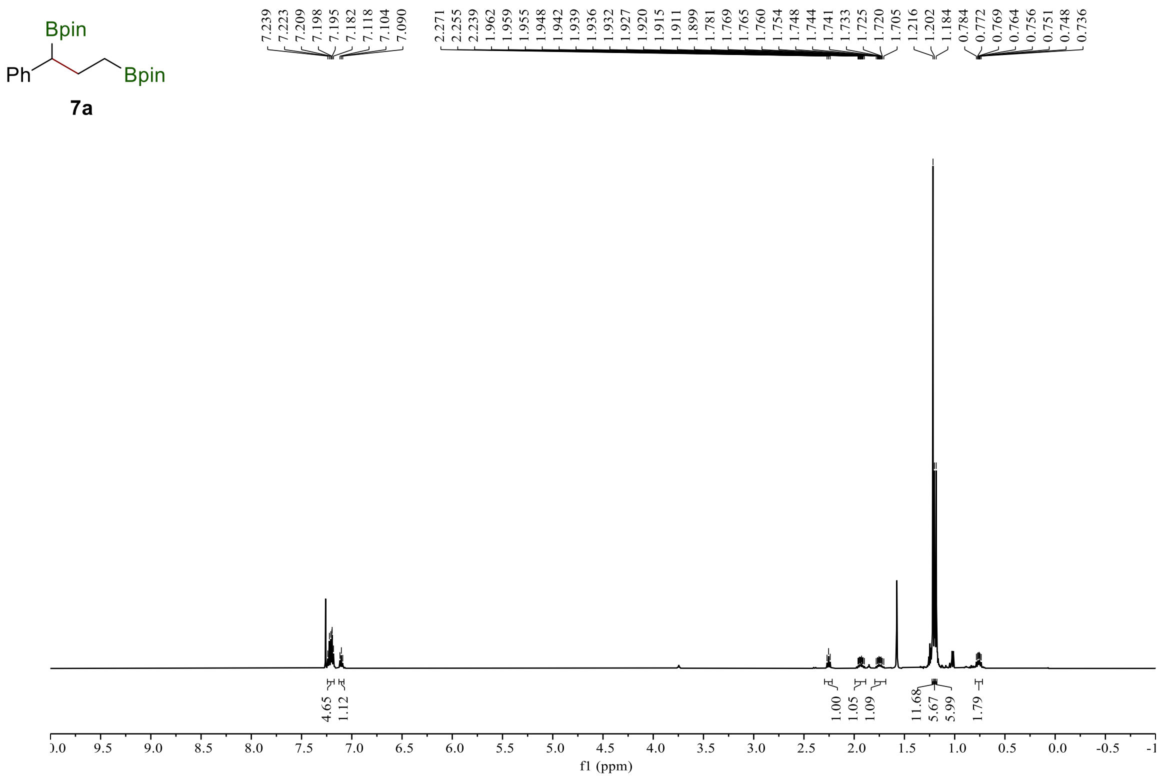

Supplementary Figure 14. ^{1}H NMR spectrum of compound **7a**.

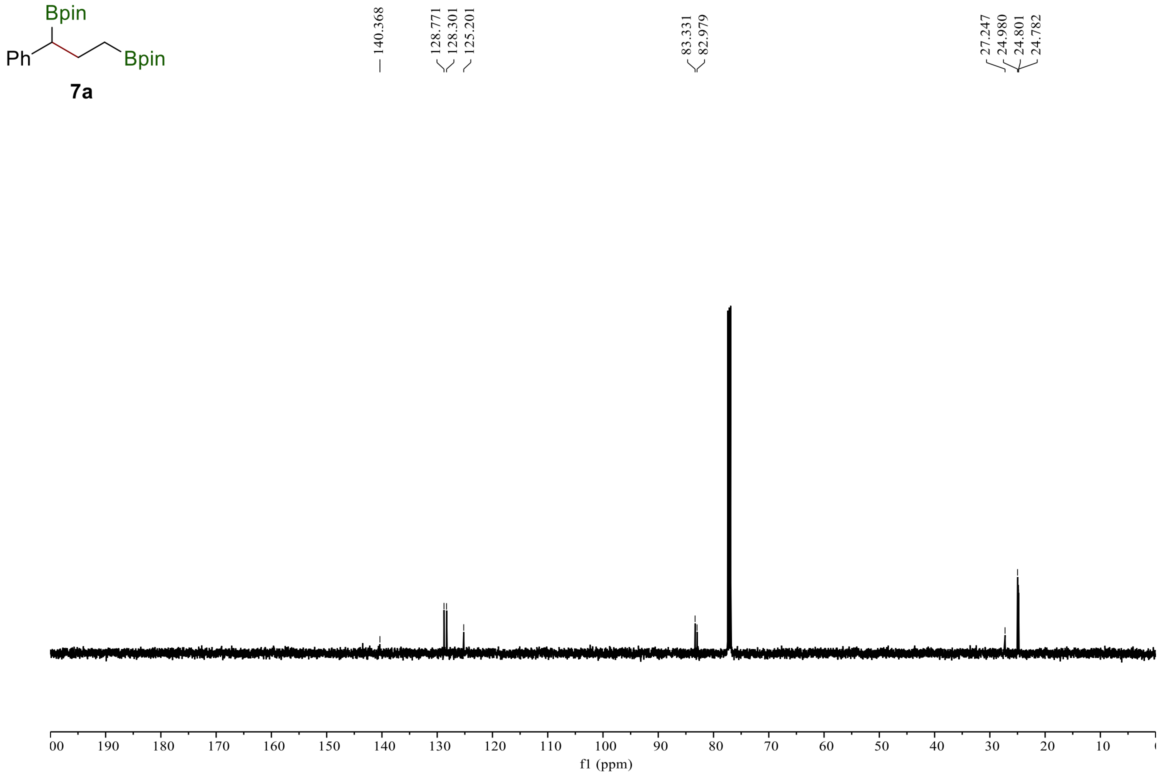

Supplementary Figure 15. $^{13}C\{^{1}H\}$ NMR spectrum of compound **7a**.

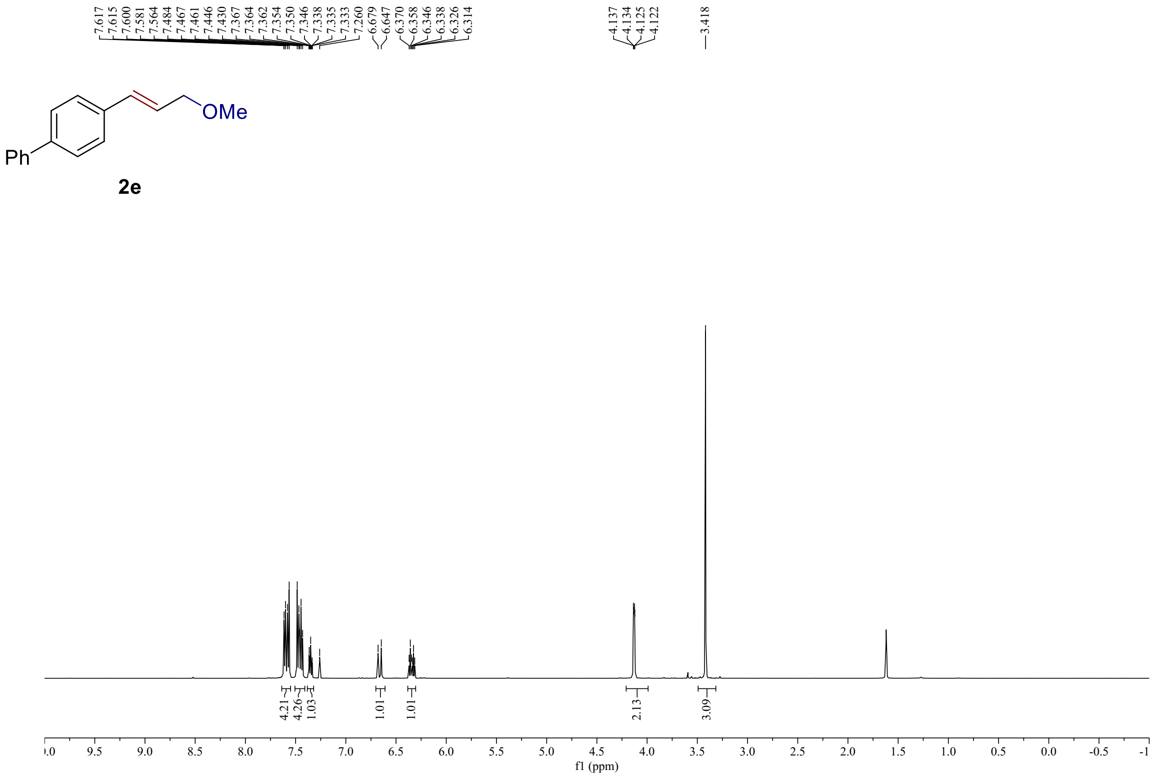

Supplementary Figure 16. ^{1}H NMR spectrum of compound **2e**.

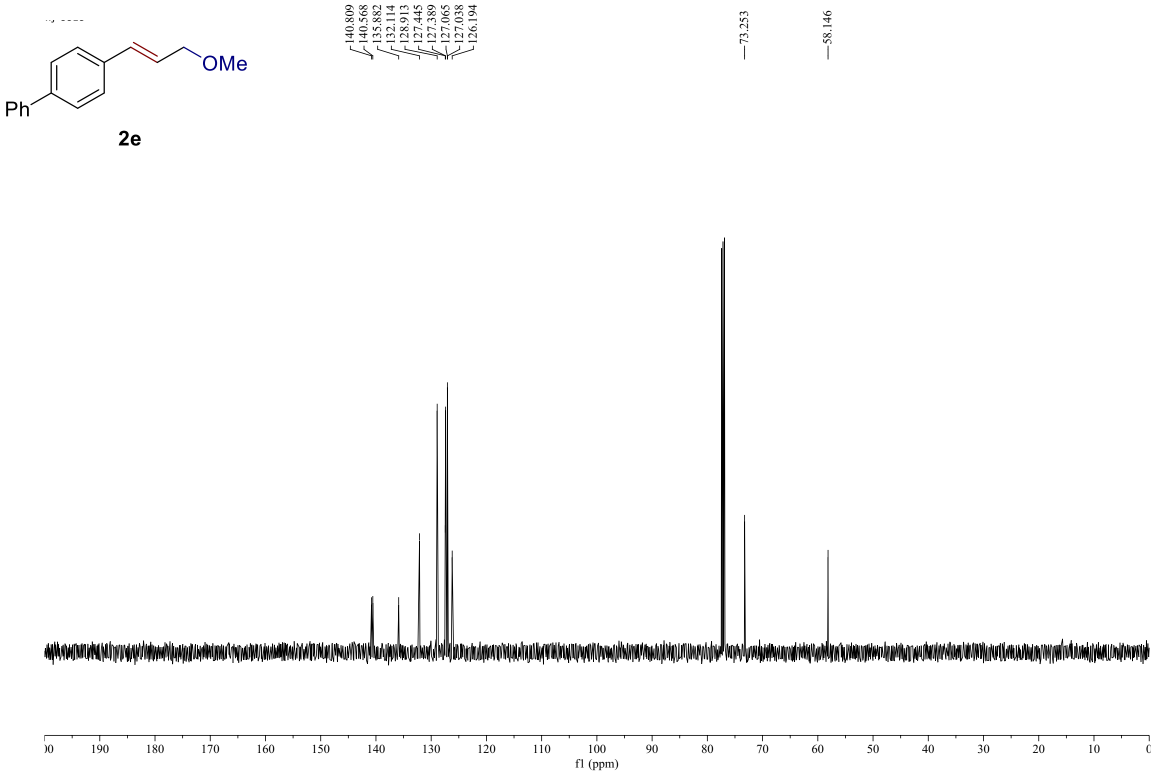

Supplementary Figure 17. $^{13}C\{^{1}H\}$ NMR spectrum of compound **2e**.

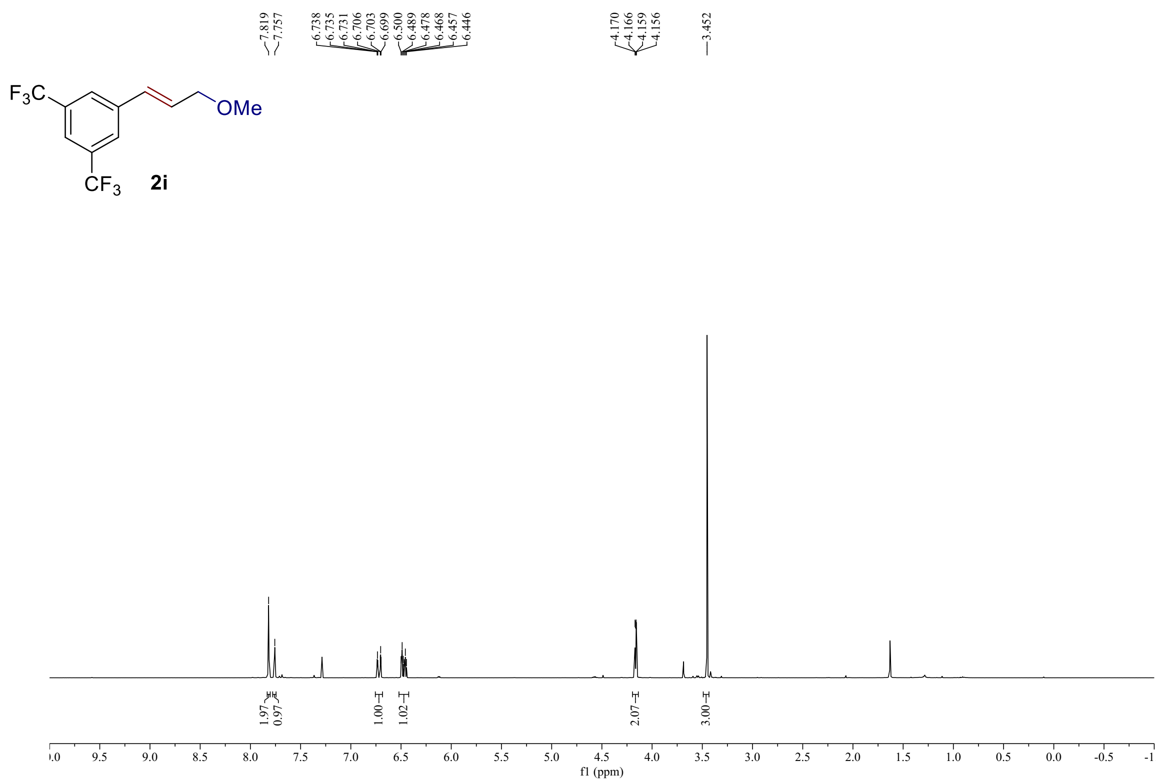

Supplementary Figure 18. ^{1}H NMR spectrum of compound **2i**.

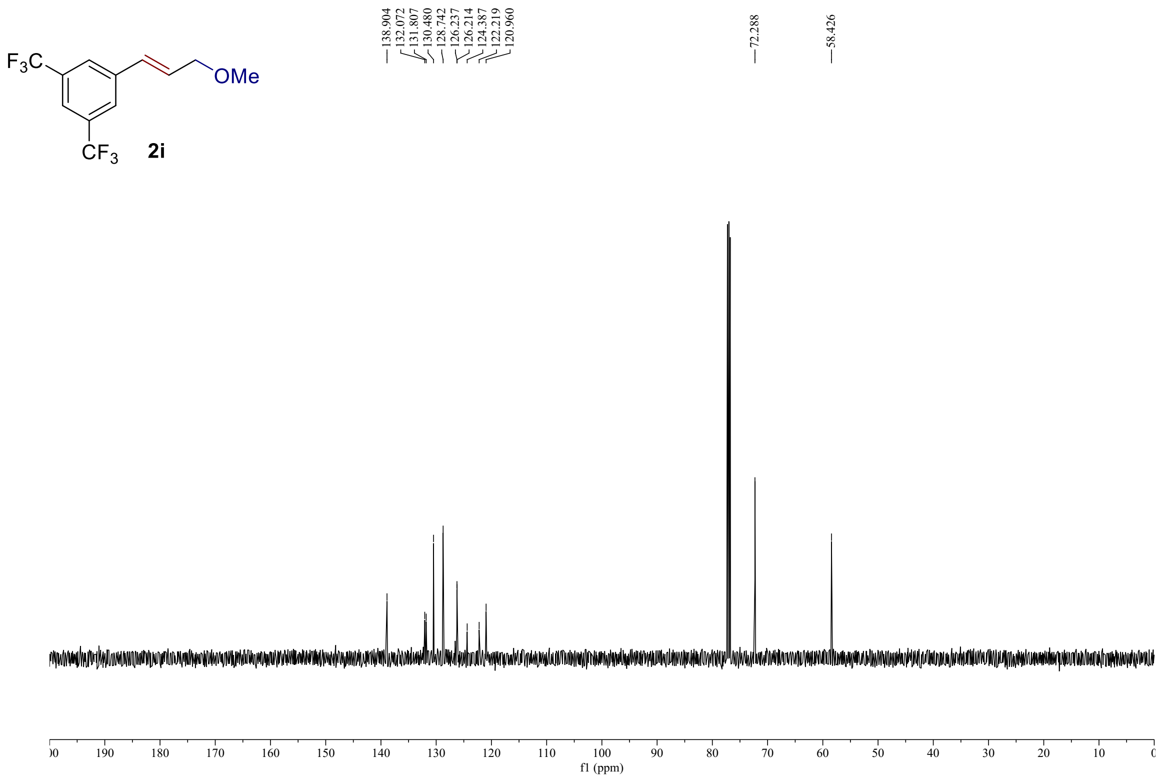

Supplementary Figure 19. $^{13}C\{^{1}H\}$ NMR spectrum of compound **2i**

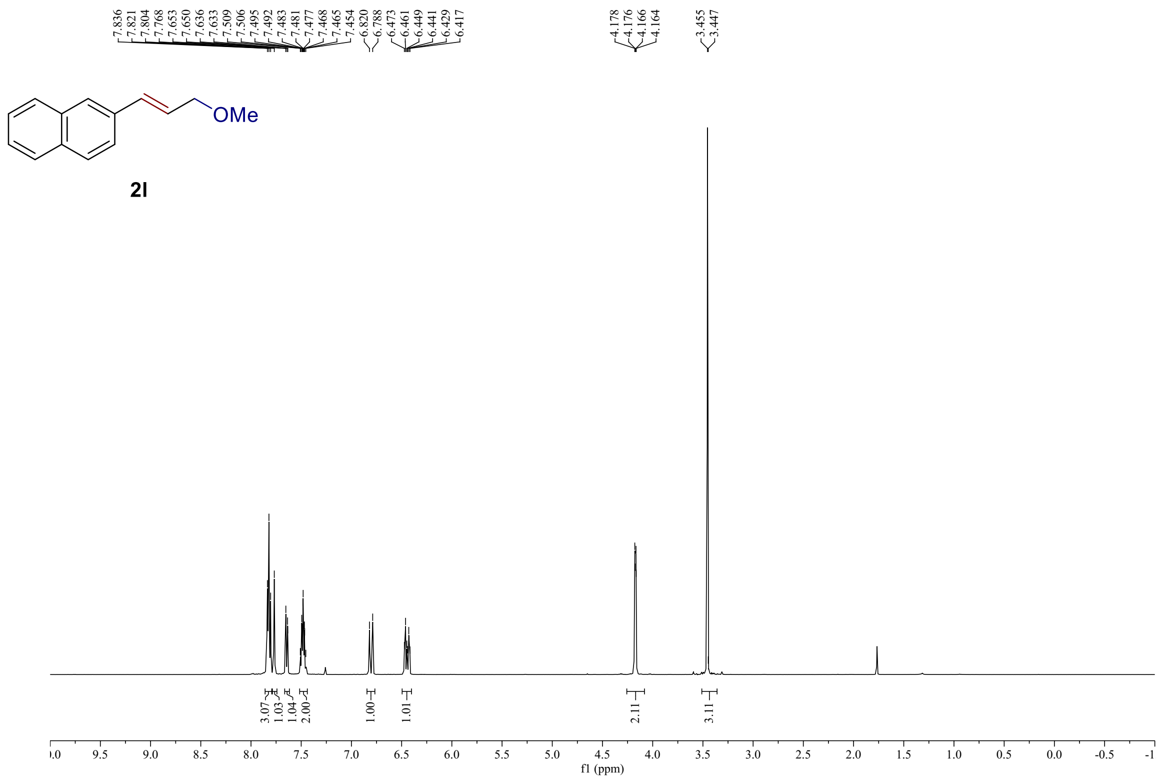

Supplementary Figure 20. ^{1}H NMR spectrum of compound **2l**.

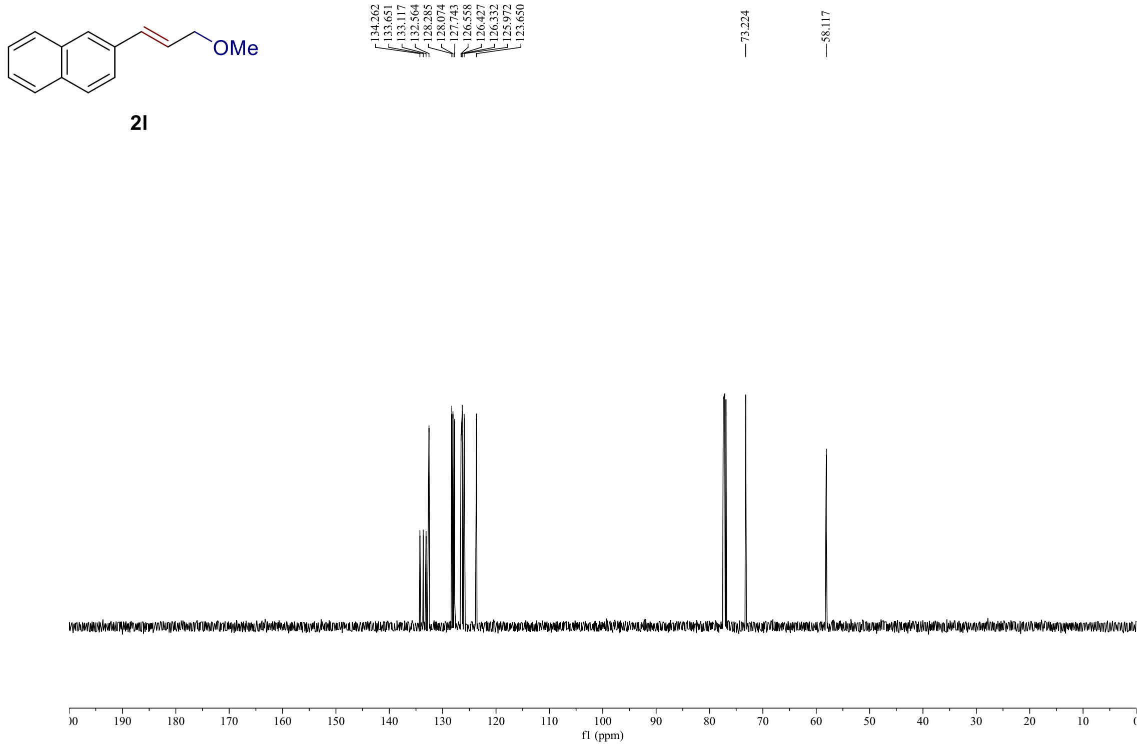

Supplementary Figure 21. $^{13}C\{^{1}H\}$ NMR spectrum of compound **2l**.

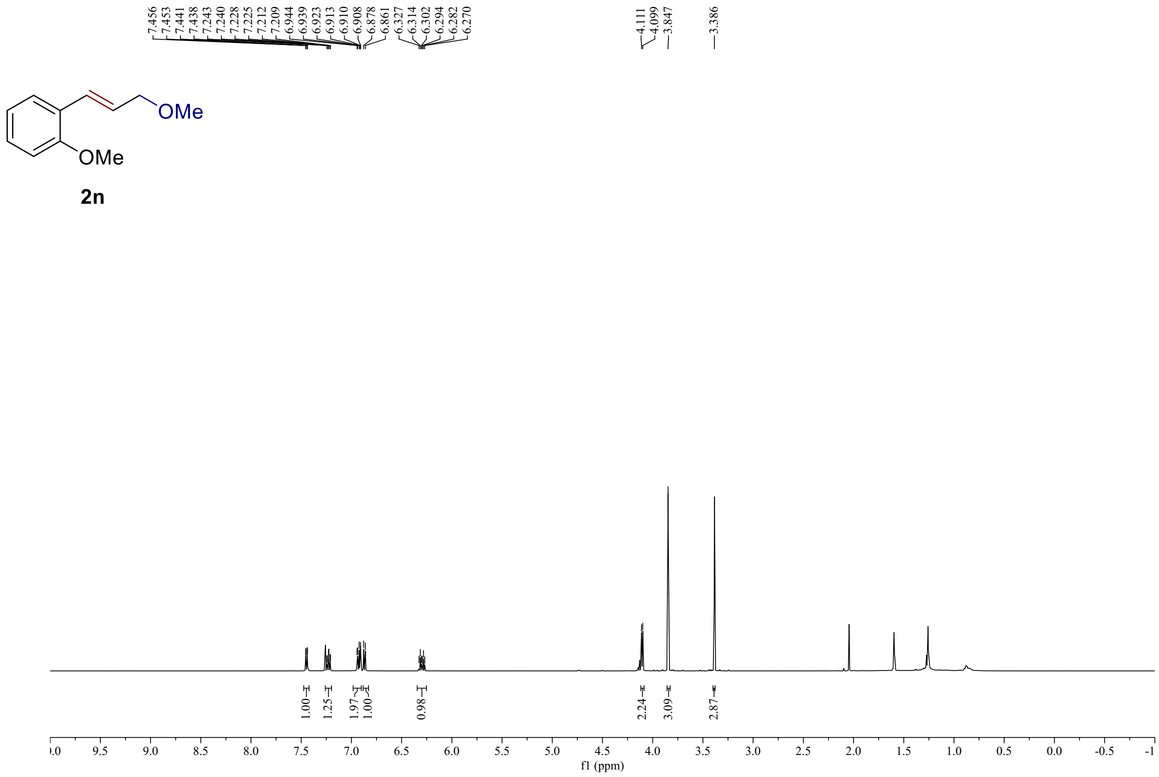

Supplementary Figure 22. ^{1}H NMR spectrum of compound **2n**.

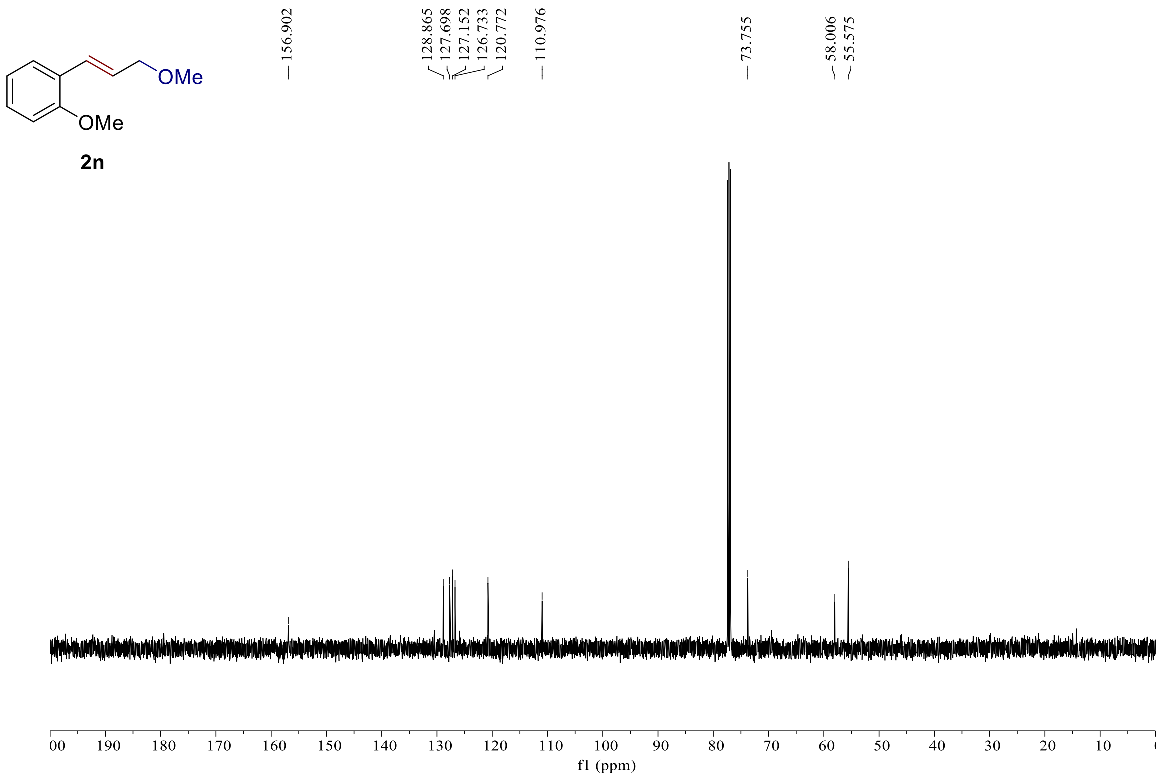

Supplementary Figure 23. $^{13}C\{^{1}H\}$ NMR spectrum of compound **2n**.

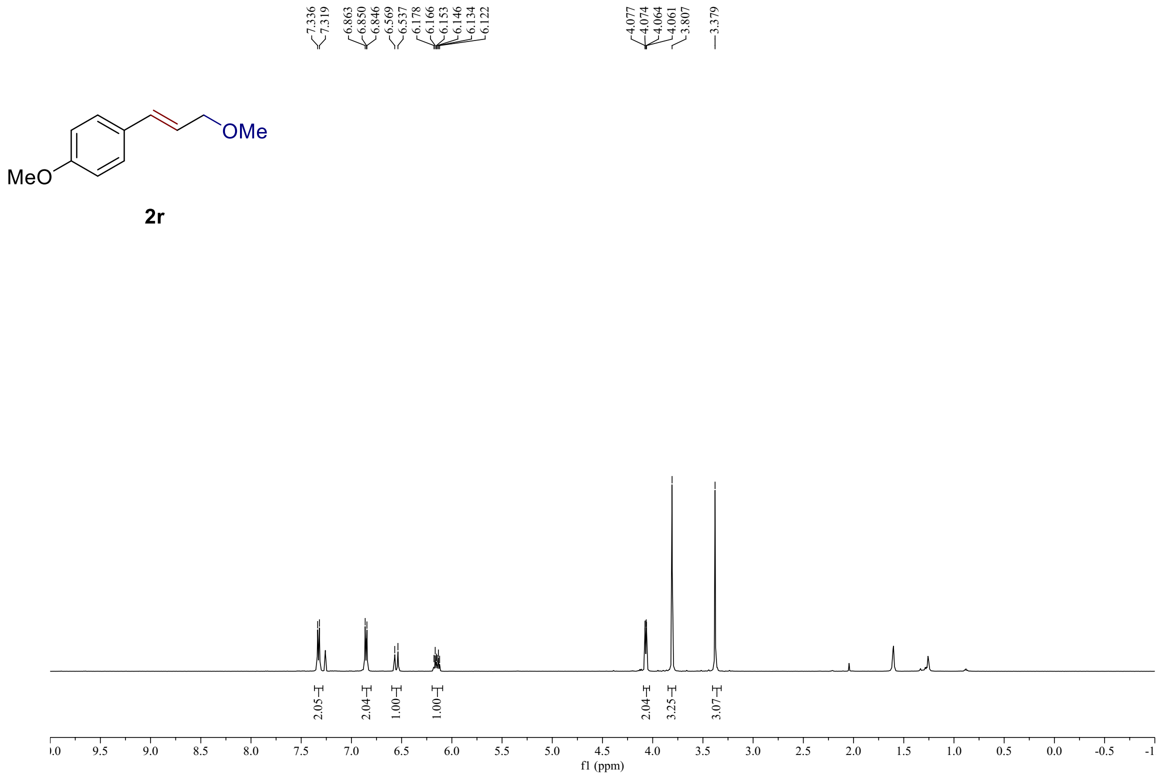

Supplementary Figure 24. ^{1}H NMR spectrum of compound **2r**.

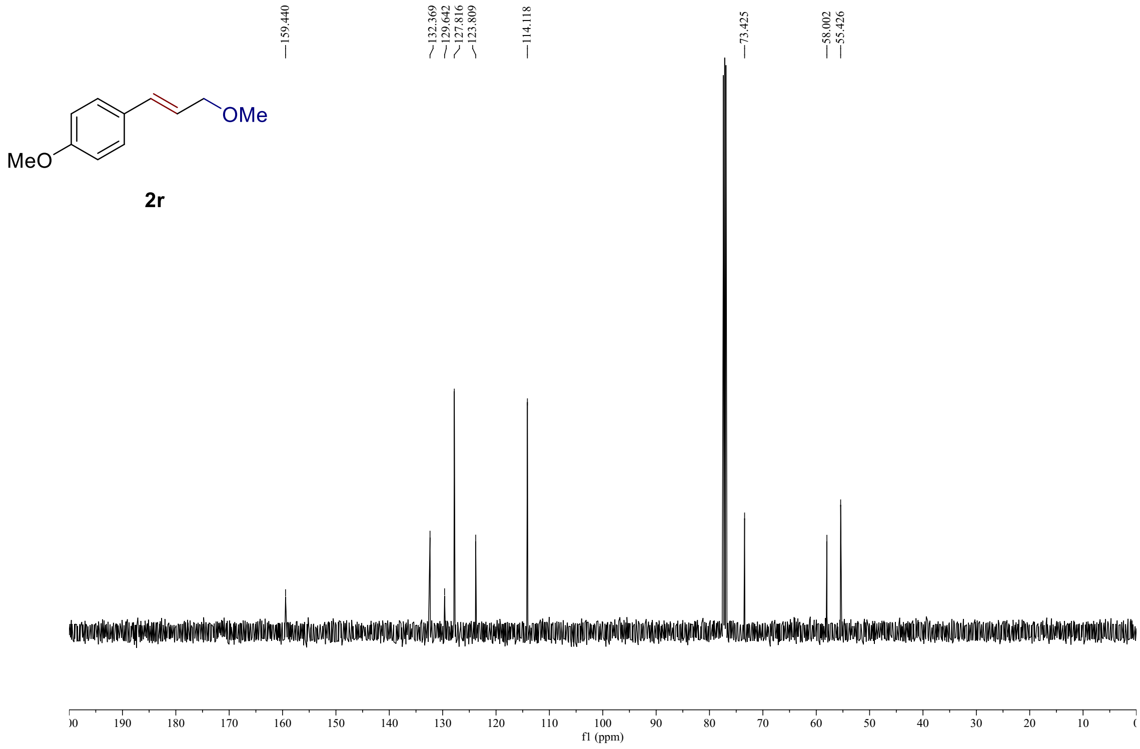

Supplementary Figure 25. $^{13}C\{^{1}H\}$ NMR spectrum of compound **2r**.

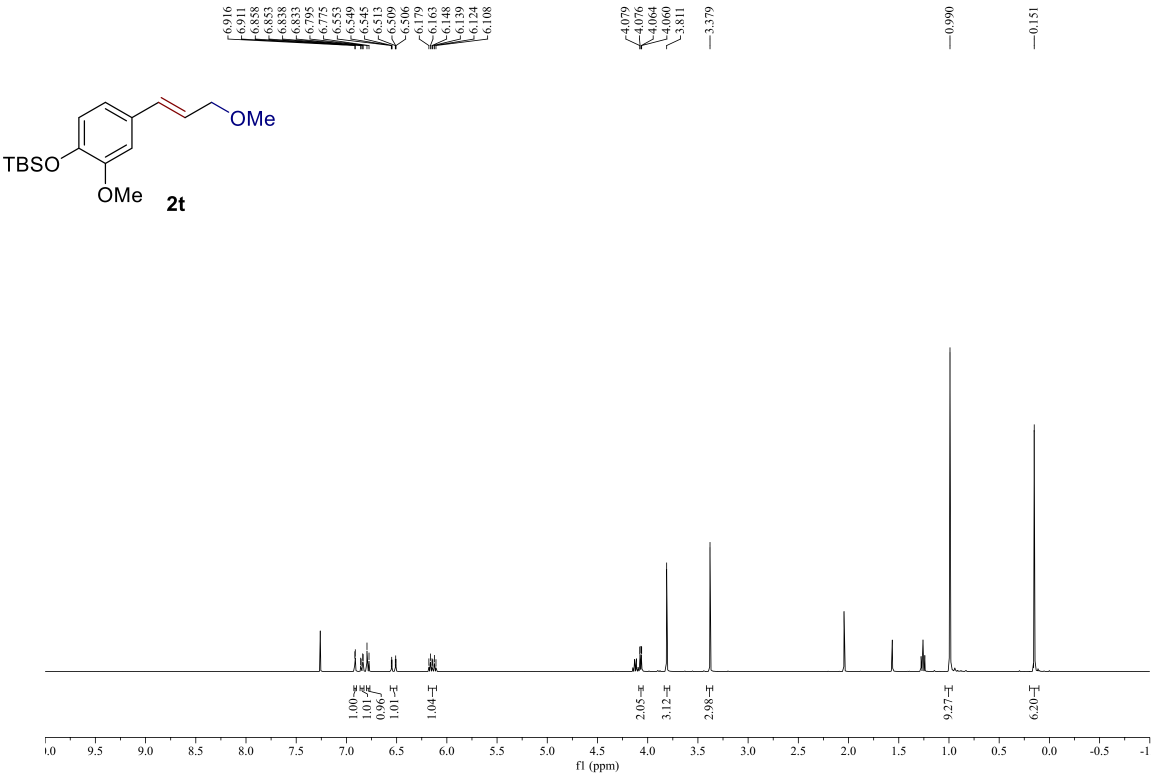

Supplementary Figure 26. ^{1}H NMR spectrum of compound **2t**.

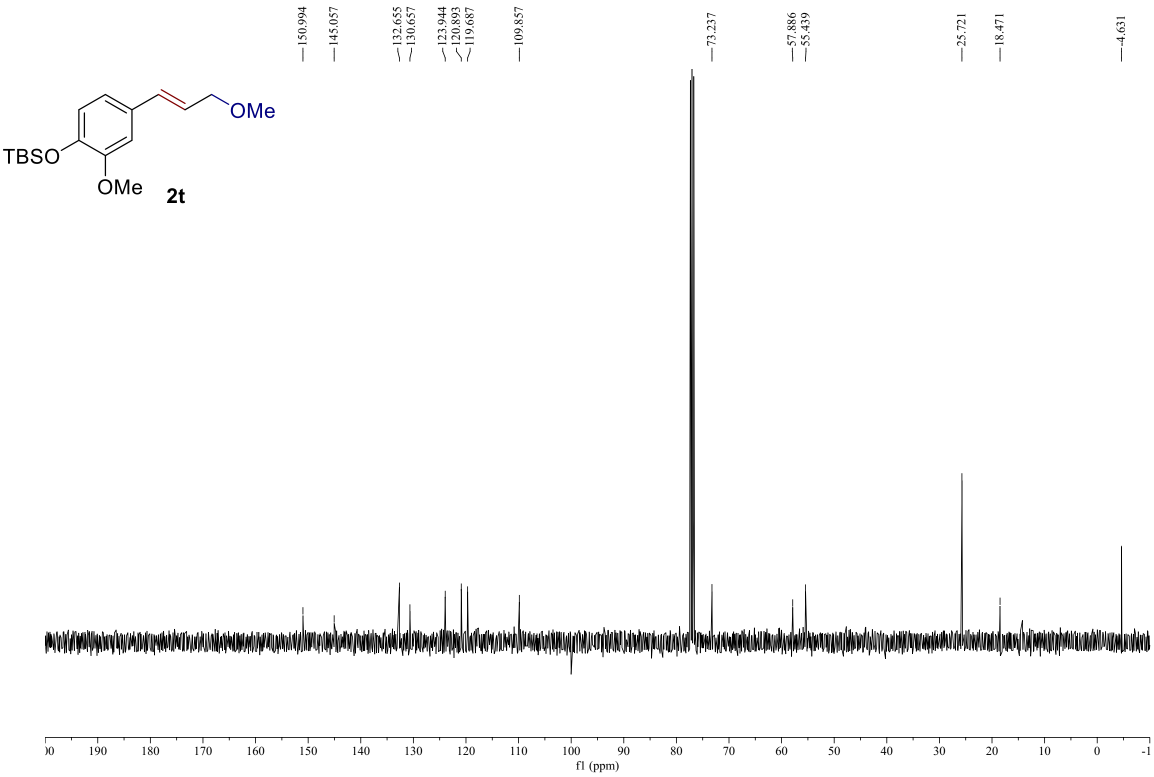

Supplementary Figure 27. $^{13}C\{^{1}H\}$ NMR spectrum of compound **2t**.

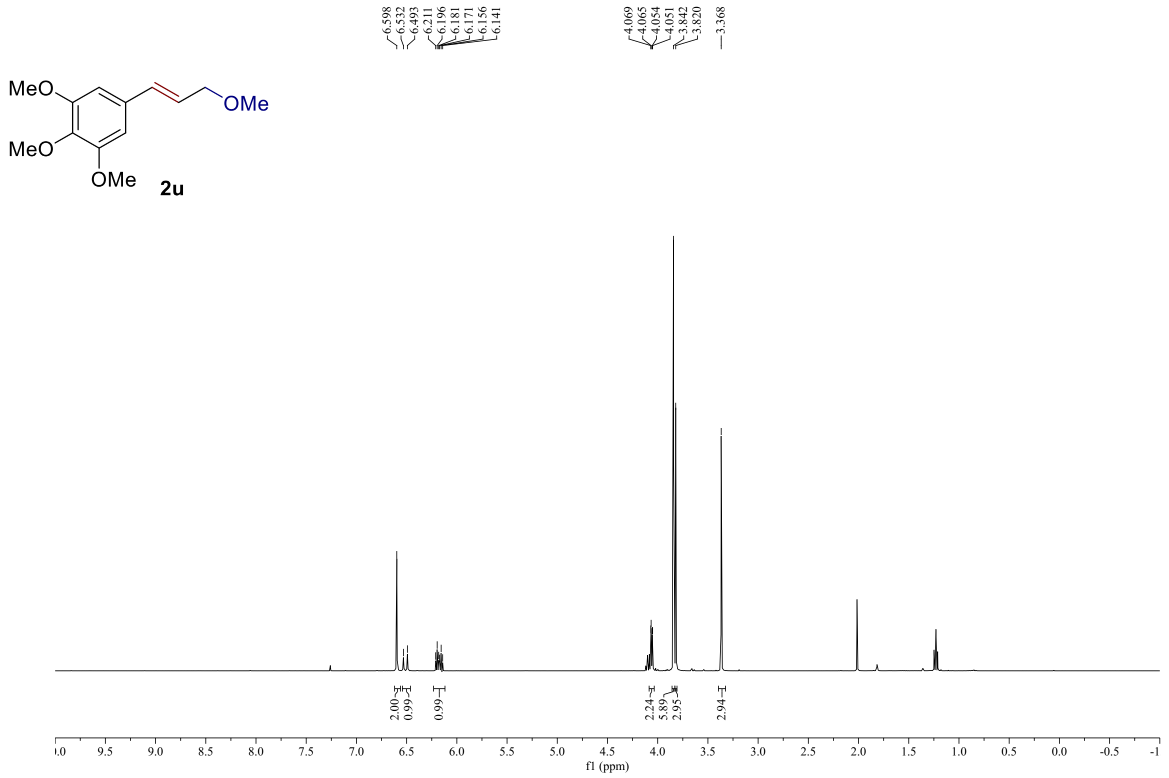

Supplementary Figure 28. ^{1}H NMR spectrum of compound **2u**.

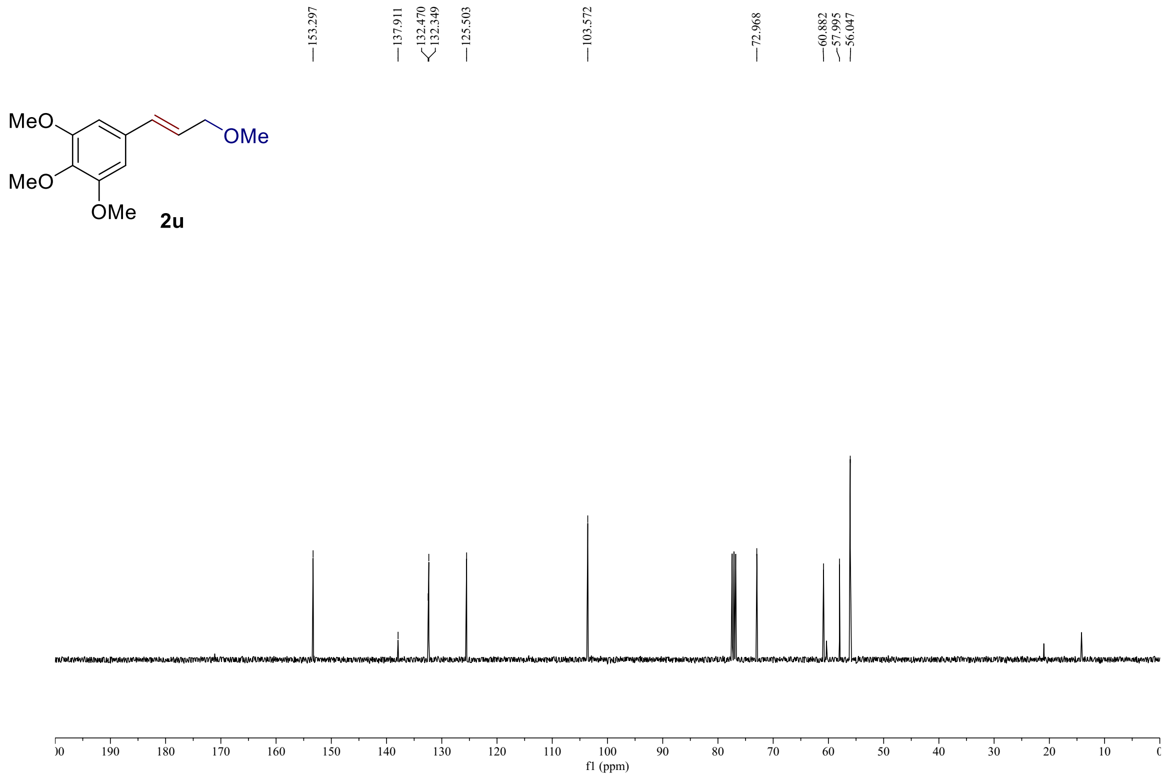

Supplementary Figure 29. $^{13}C\{^{1}H\}$ NMR spectrum of compound **2u**.

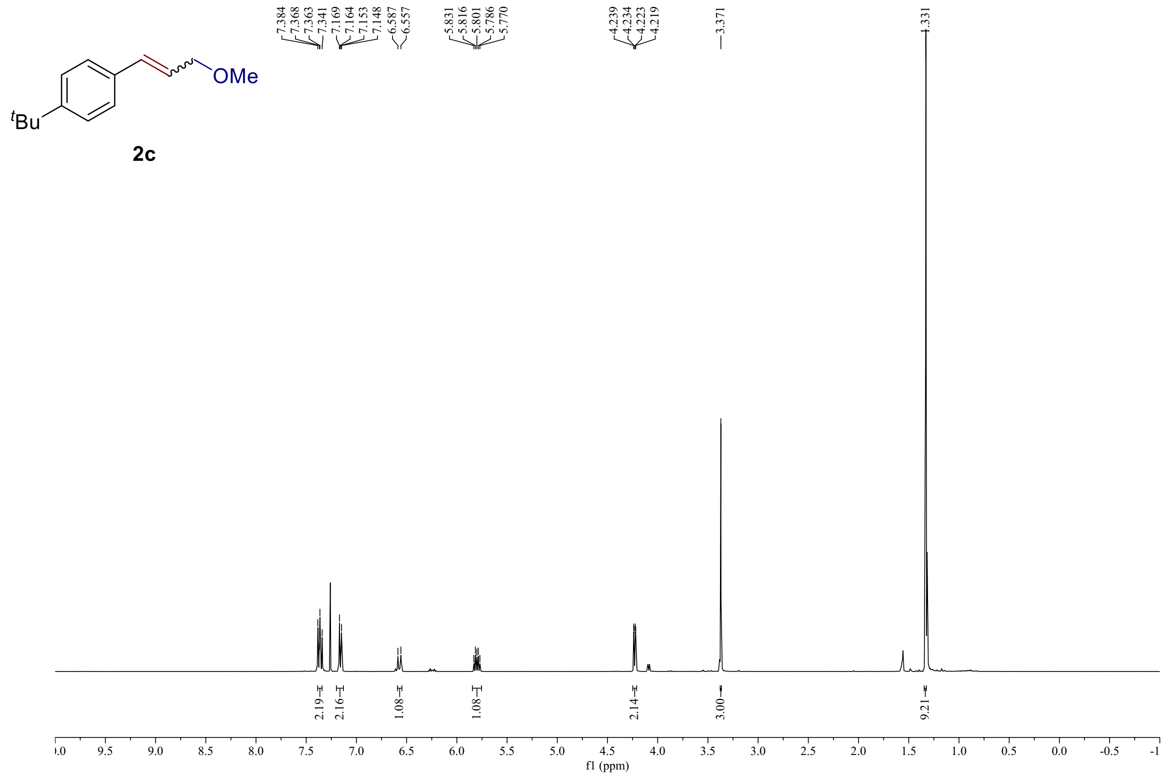

Supplementary Figure 30. ^{1}H NMR spectrum of compound **2c**.

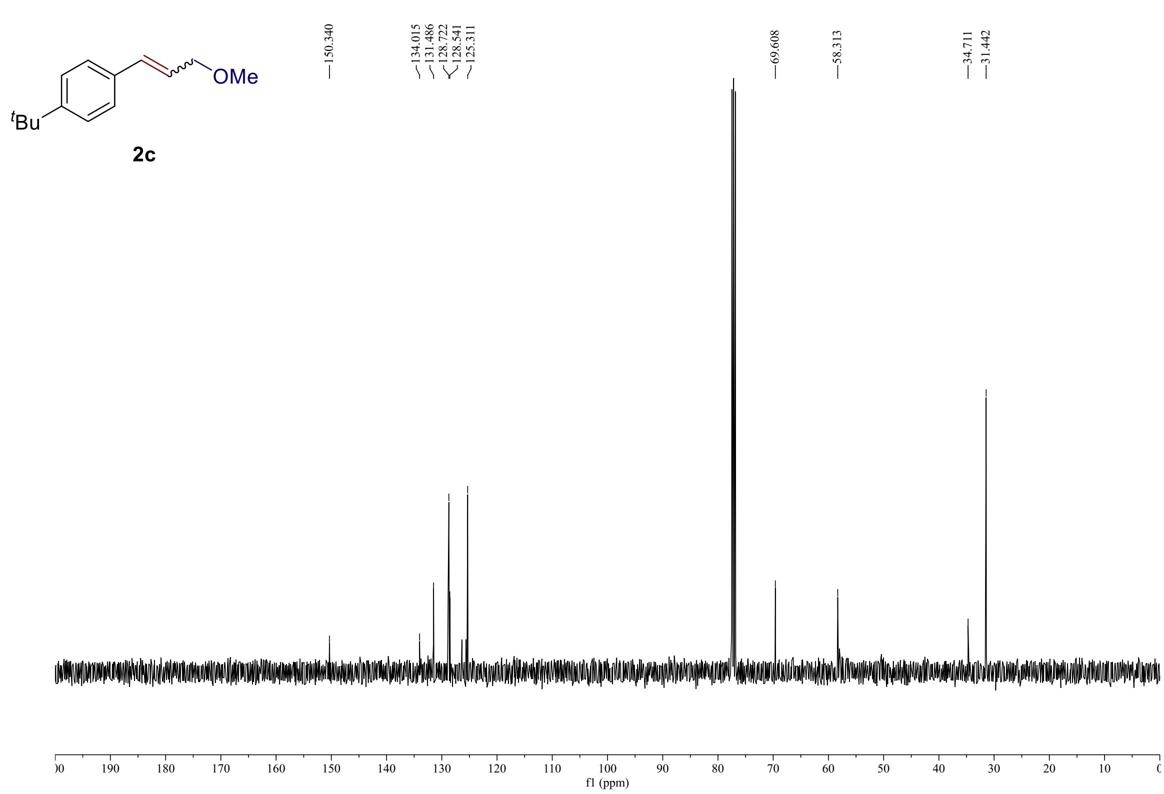

Supplementary Figure 31. ^{13}C{^{1}H} NMR spectrum of compound **2c**.

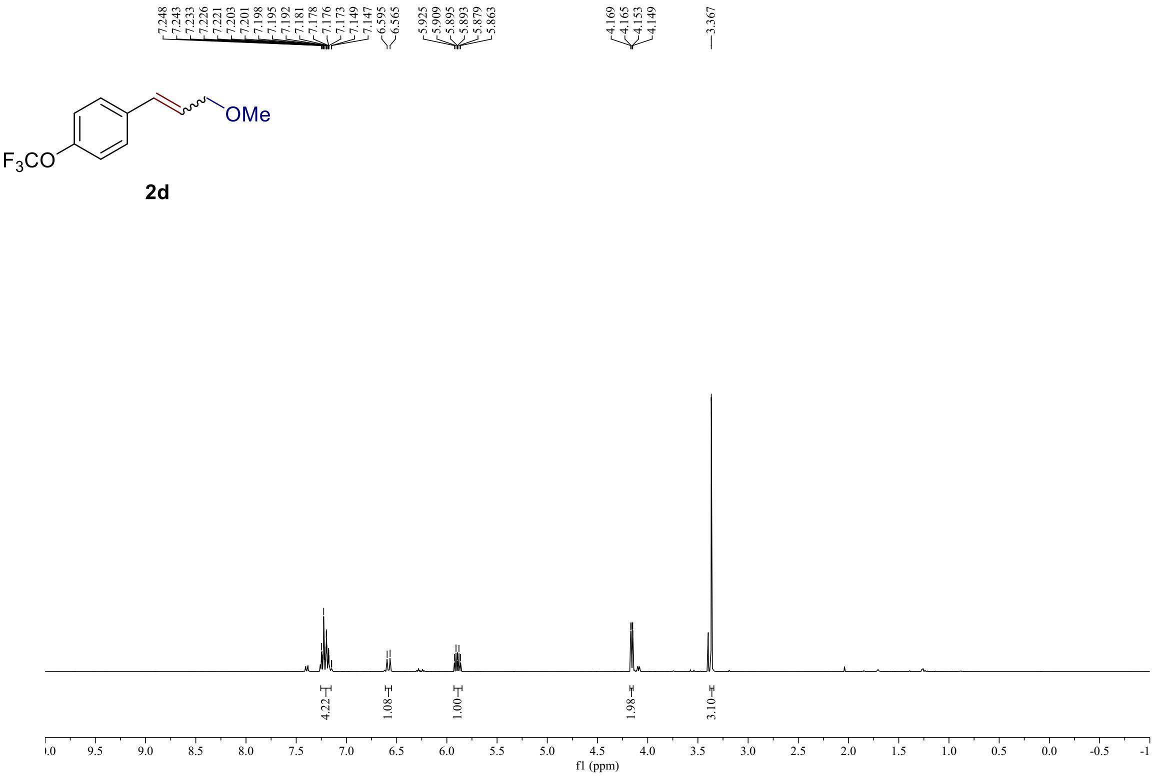

Supplementary Figure 32. ^{1}H NMR spectrum of compound **2d**.

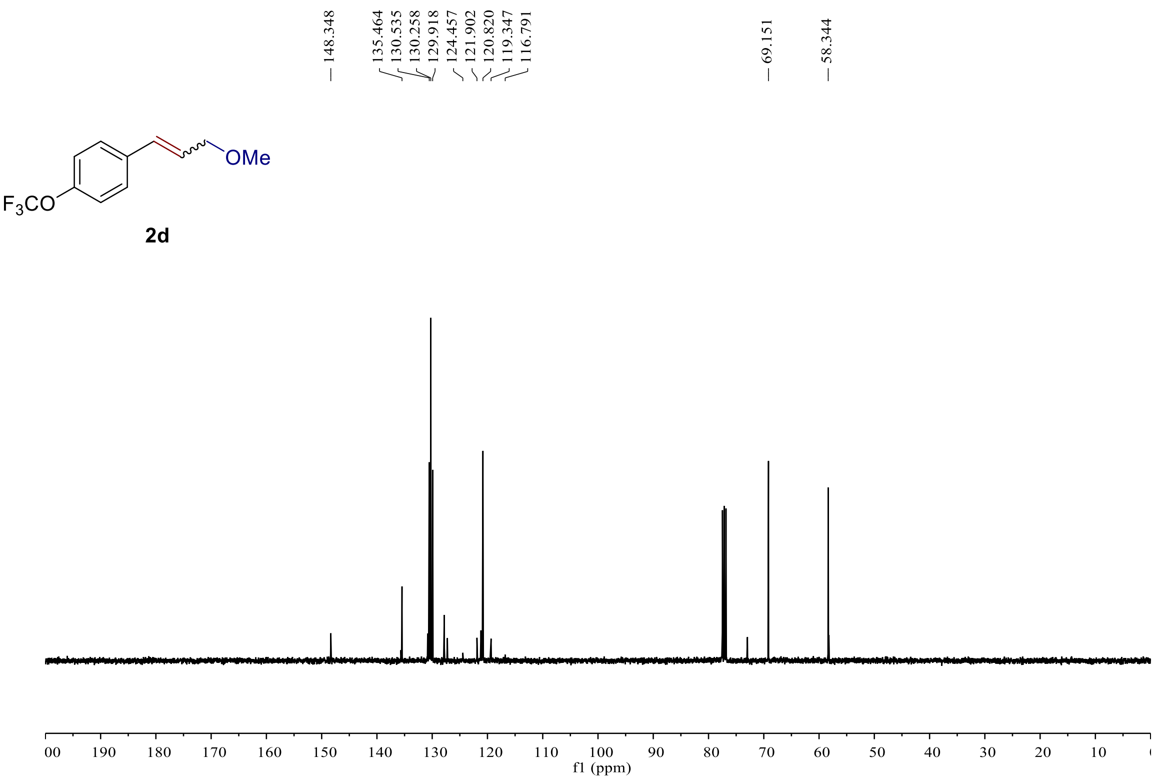

Supplementary Figure 33. ^{13}C{^{1}H} NMR spectrum of compound **2d**.

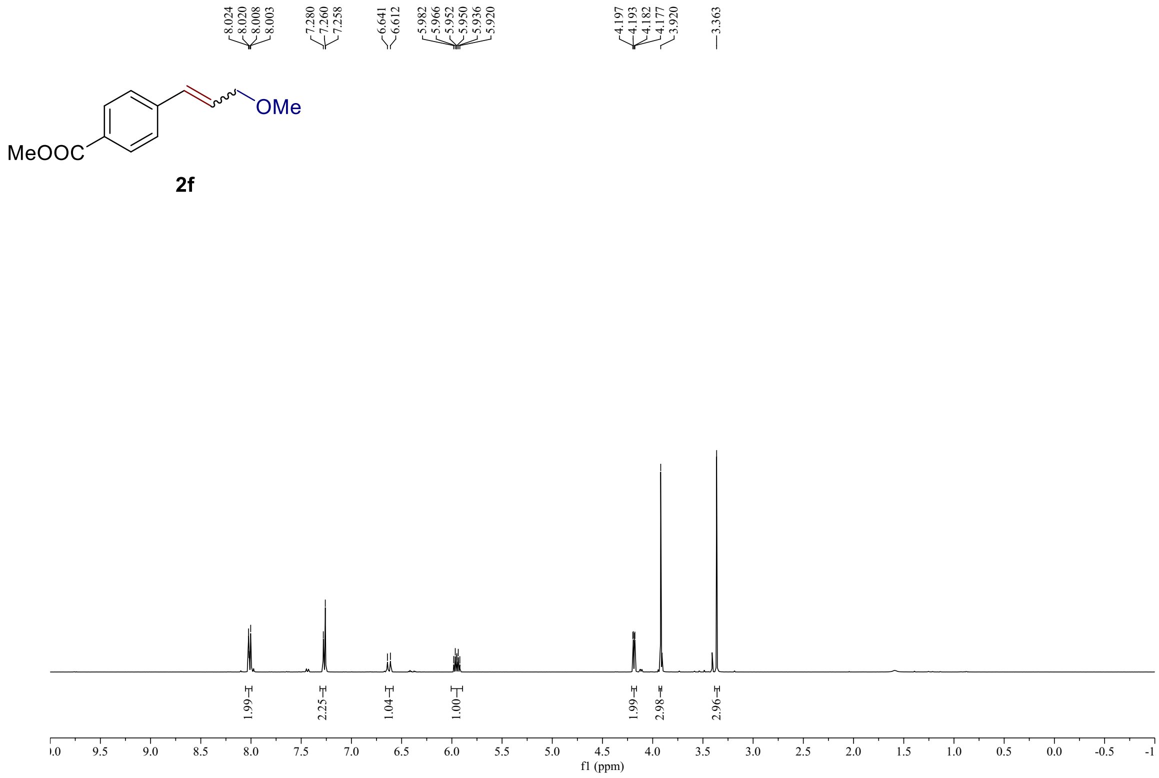

Supplementary Figure 34. ^{1}H NMR spectrum of compound **2f**.

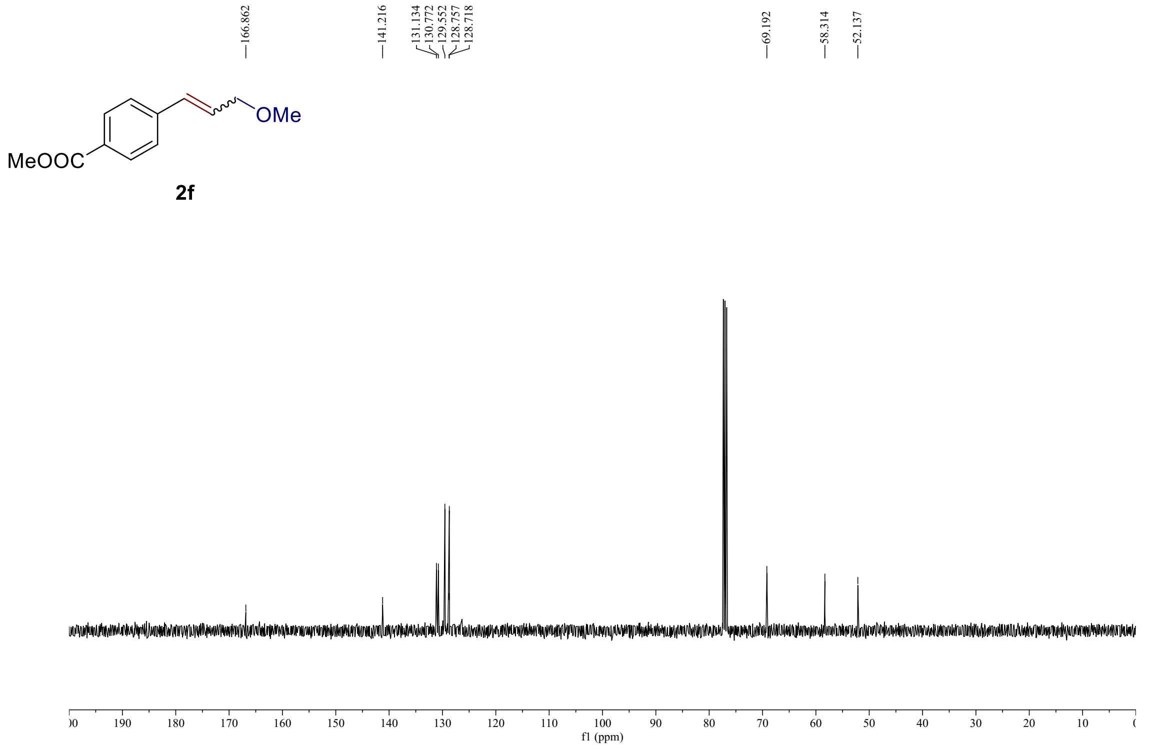

Supplementary Figure 35. $^{13}C\{^{1}H\}$ NMR spectrum of compound **2f**.

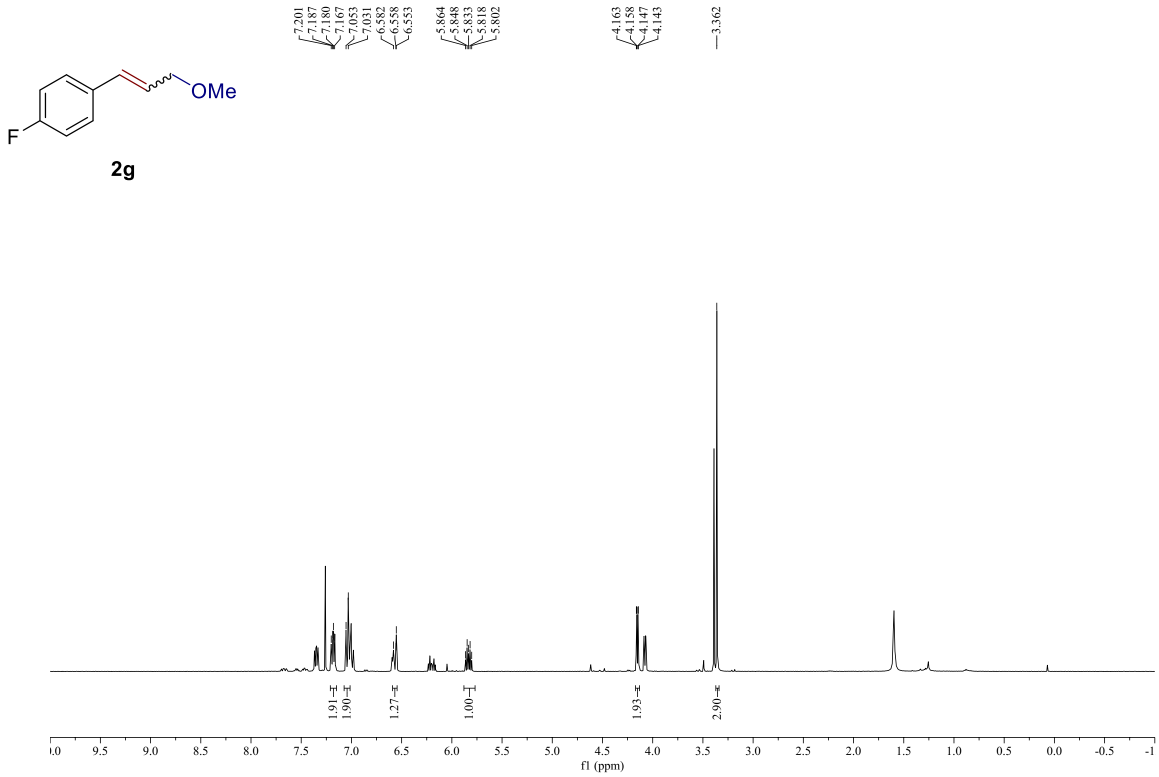

Supplementary Figure 36. ^{1}H NMR spectrum of compound **2g**.

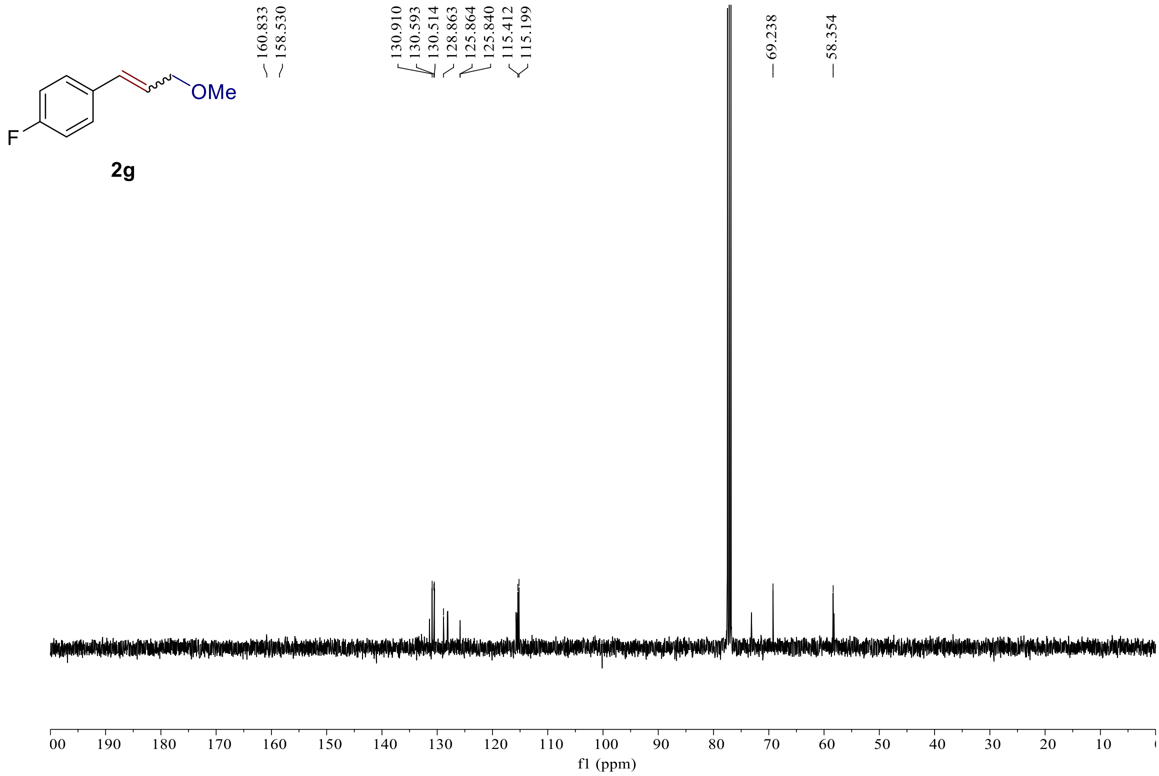

Supplementary Figure 37. ^{13}C{^{1}H} NMR spectrum of compound **2g**.

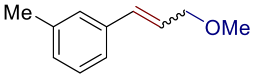

2h

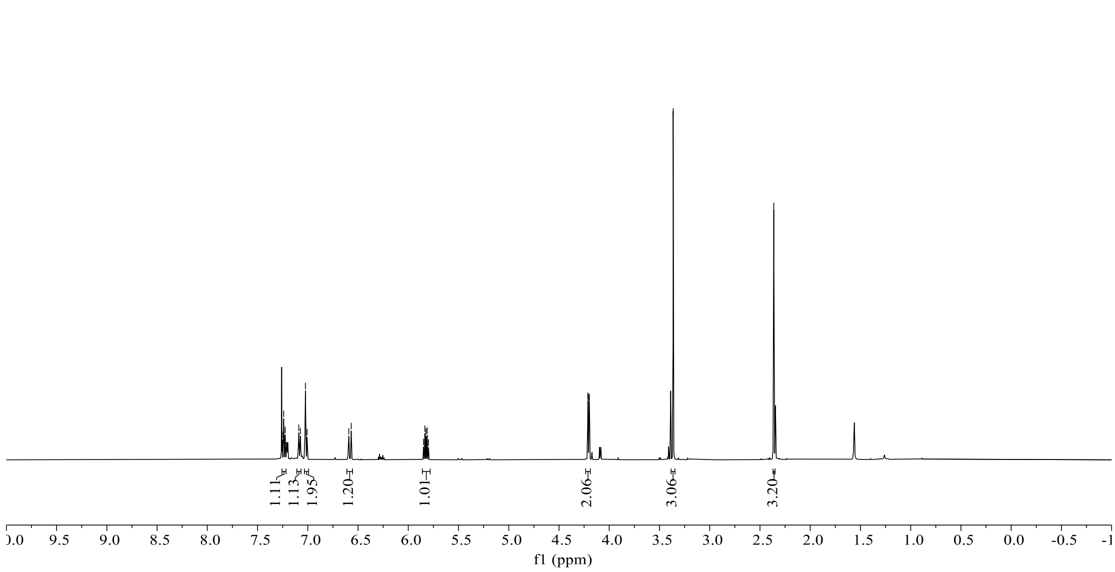

Supplementary Figure 38. ^{1}H NMR spectrum of compound **2h**.

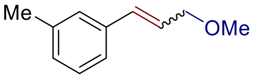

2h

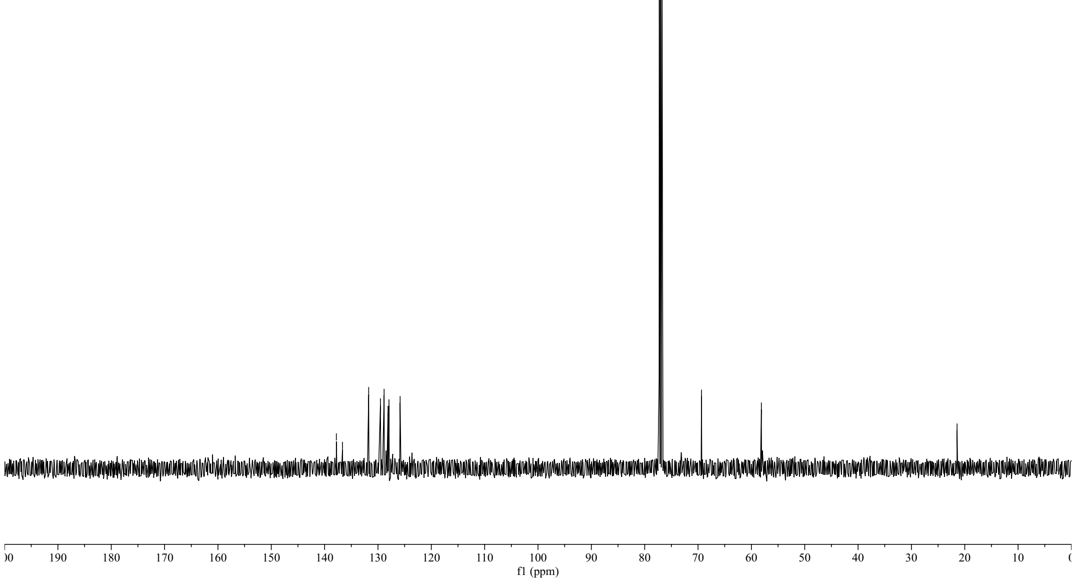

Supplementary Figure 39. ^{13}C{^{1}H} NMR spectrum of compound **2h**.

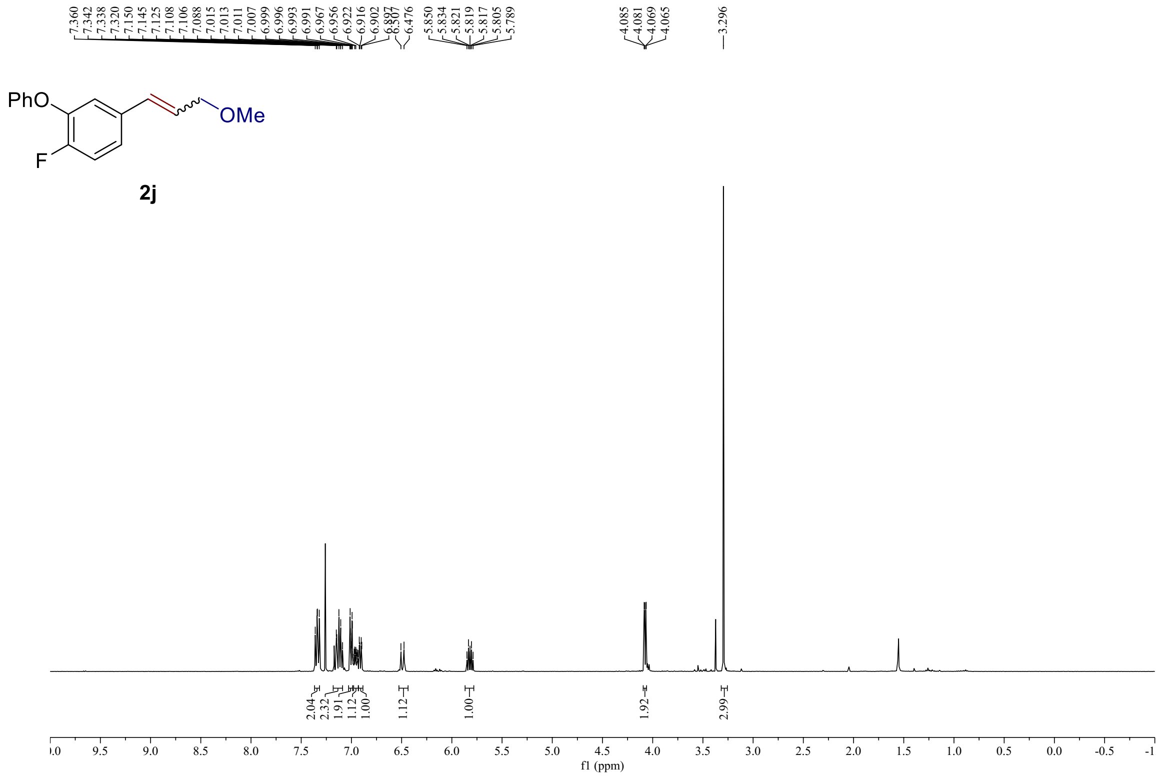

Supplementary Figure 40. ^{1}H NMR spectrum of compound **2f**.

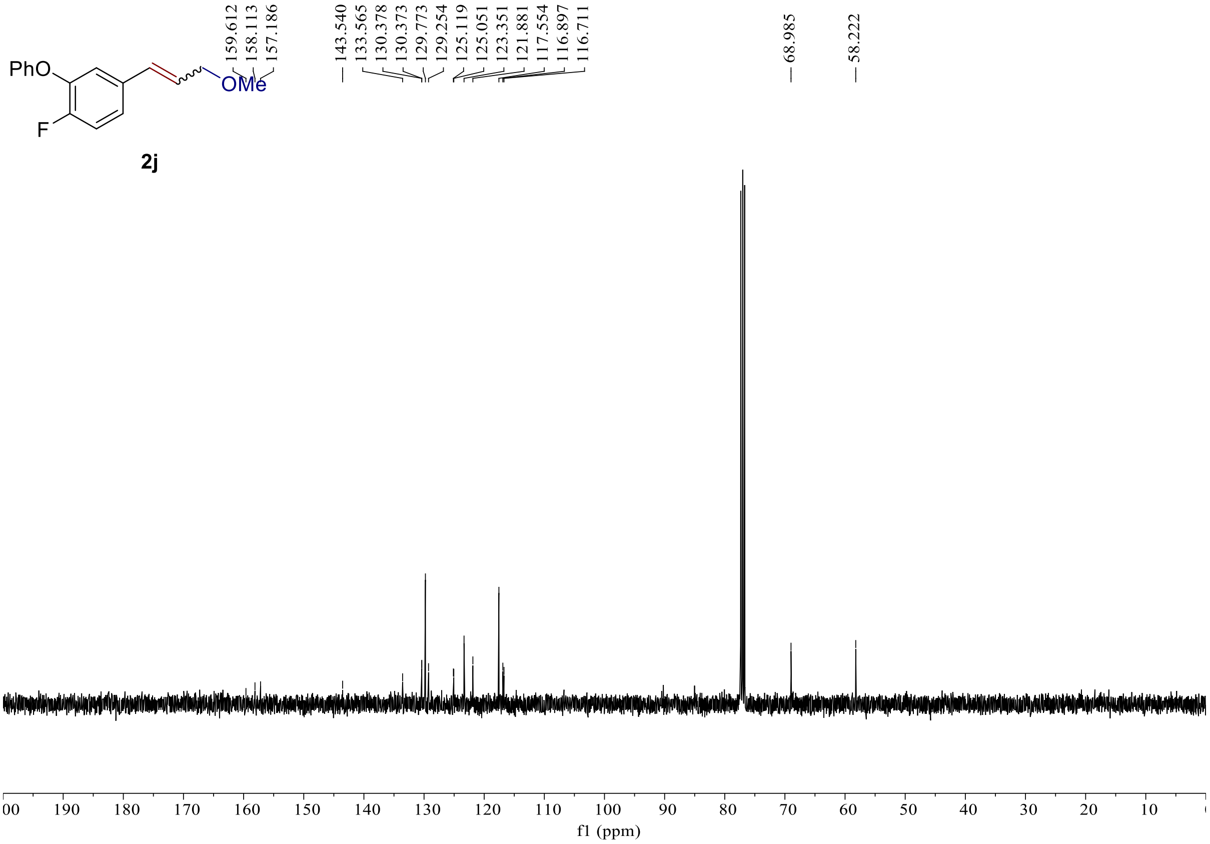

Supplementary Figure 41. $^{13}C\{^{1}H\}$ NMR spectrum of compound **2f**.

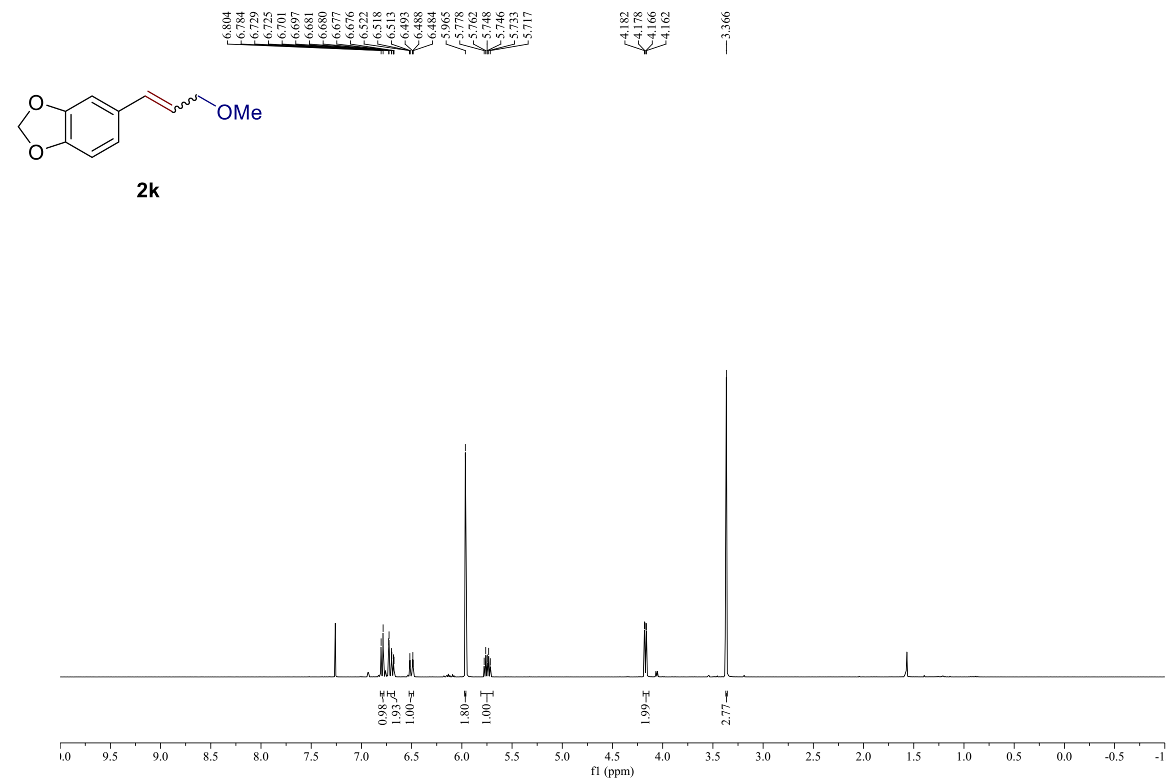

Supplementary Figure 42. ^{1}H NMR spectrum of compound **2k**.

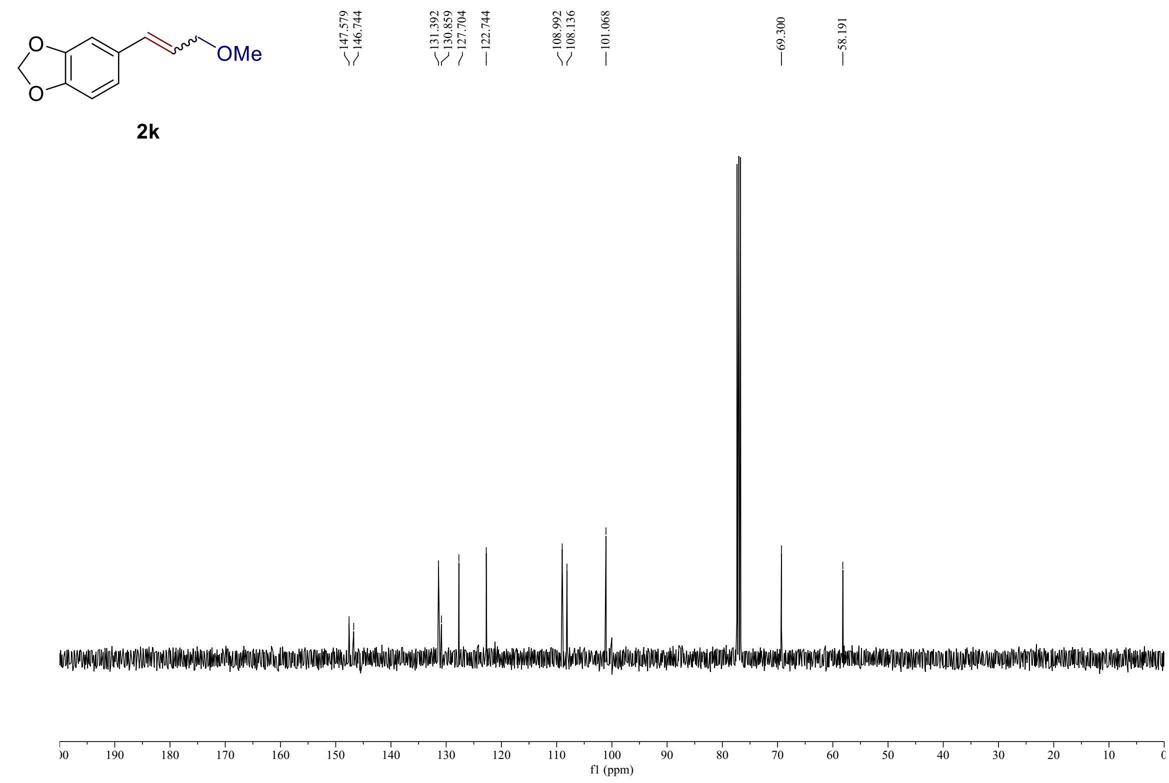

Supplementary Figure 43. $^{13}C\{^1H\}$ NMR spectrum of compound **2k**.

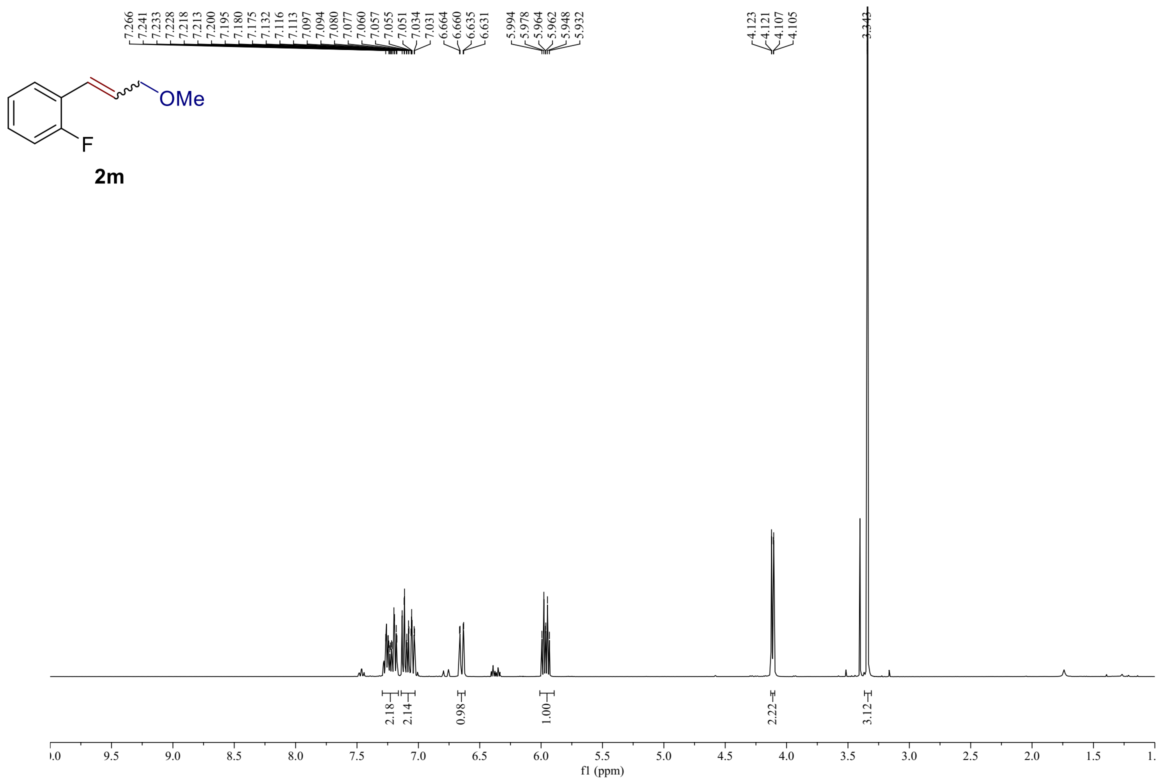

Supplementary Figure 44. ^{1}H NMR spectrum of compound **2m**.

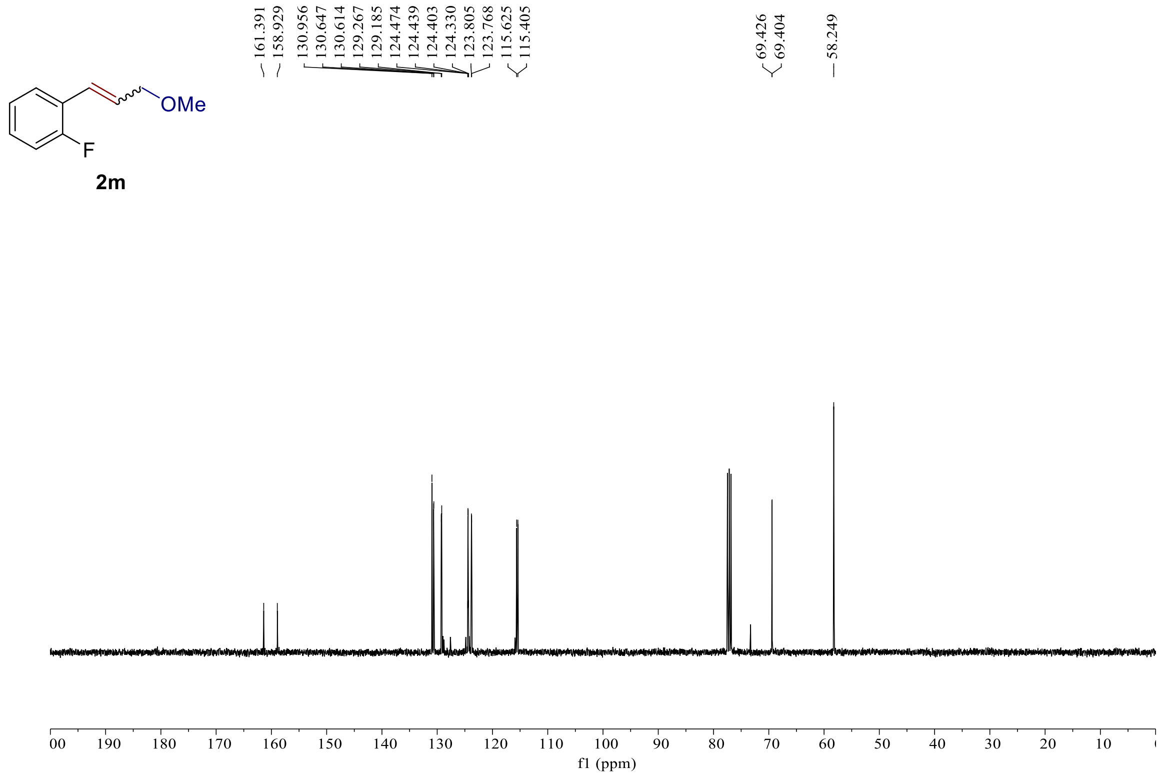

Supplementary Figure 45. ^{13}C{^{1}H} NMR spectrum of compound **2m**.

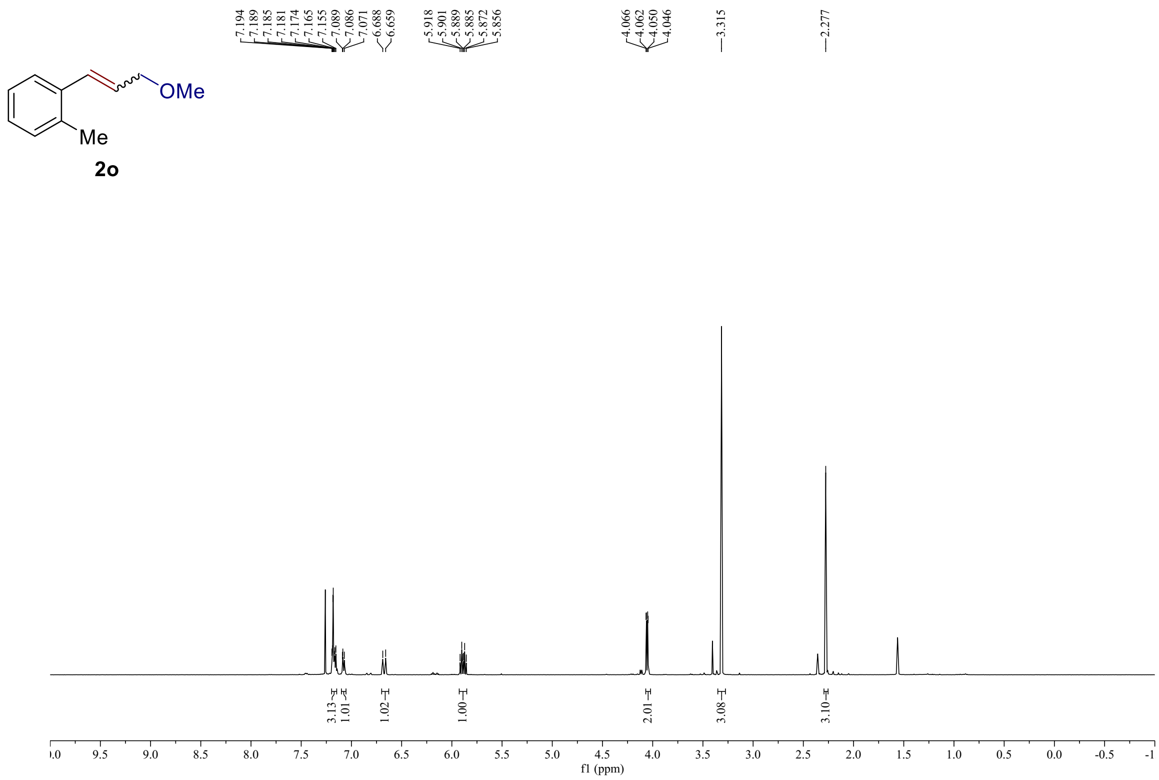

Supplementary Figure 46. ^{1}H NMR spectrum of compound **2o**.

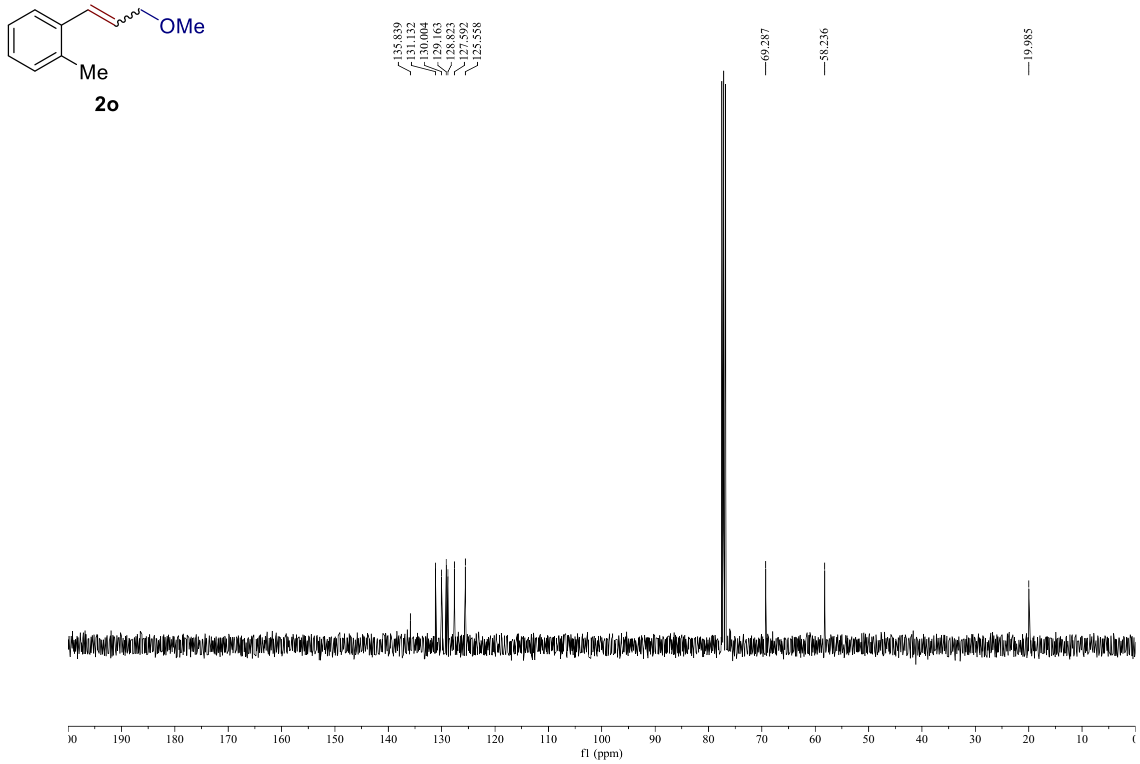

Supplementary Figure 47. $^{13}C\{^{1}H\}$ NMR spectrum of compound **2o**.

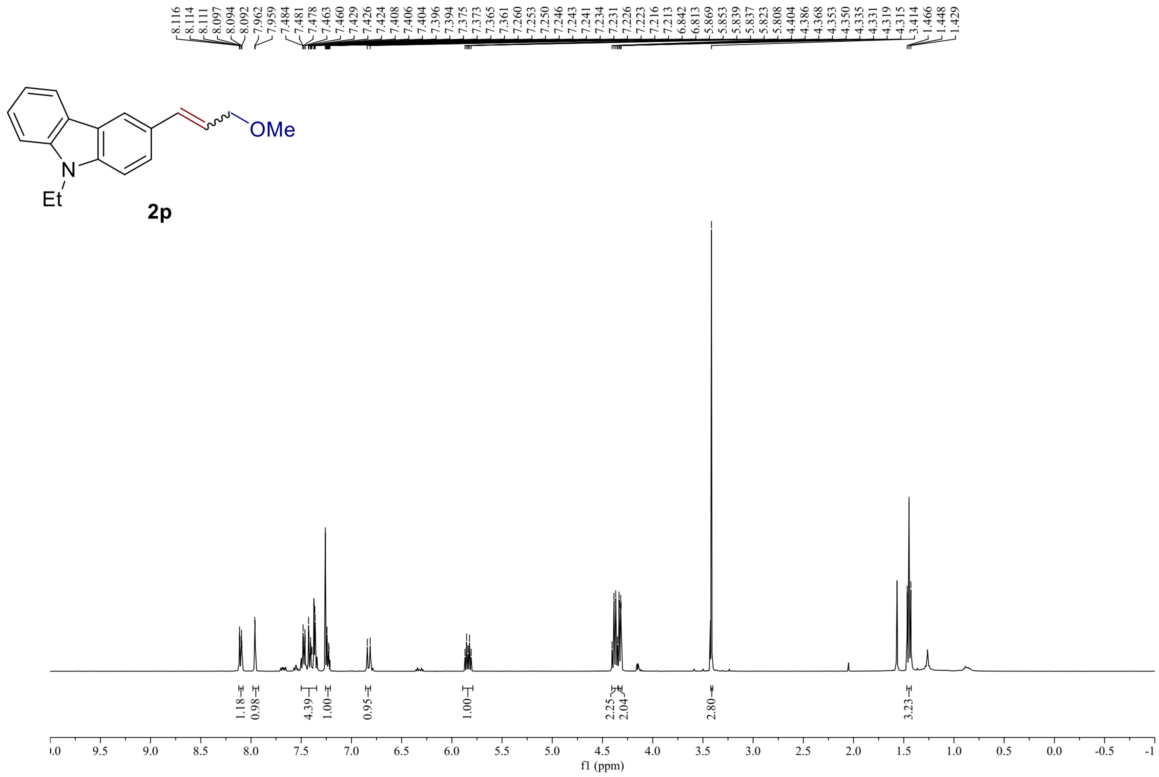

Supplementary Figure 48. ^{1}H NMR spectrum of compound **2p**.

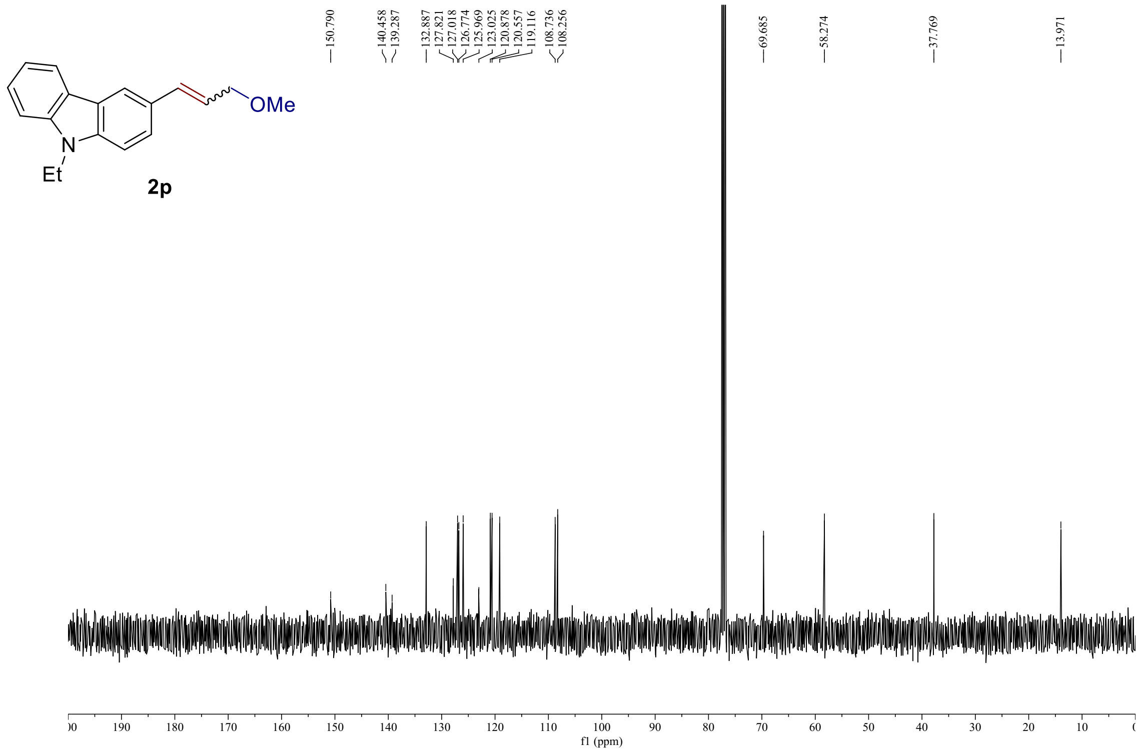

Supplementary Figure 49. ^{13}C{^{1}H} NMR spectrum of compound **2p**.

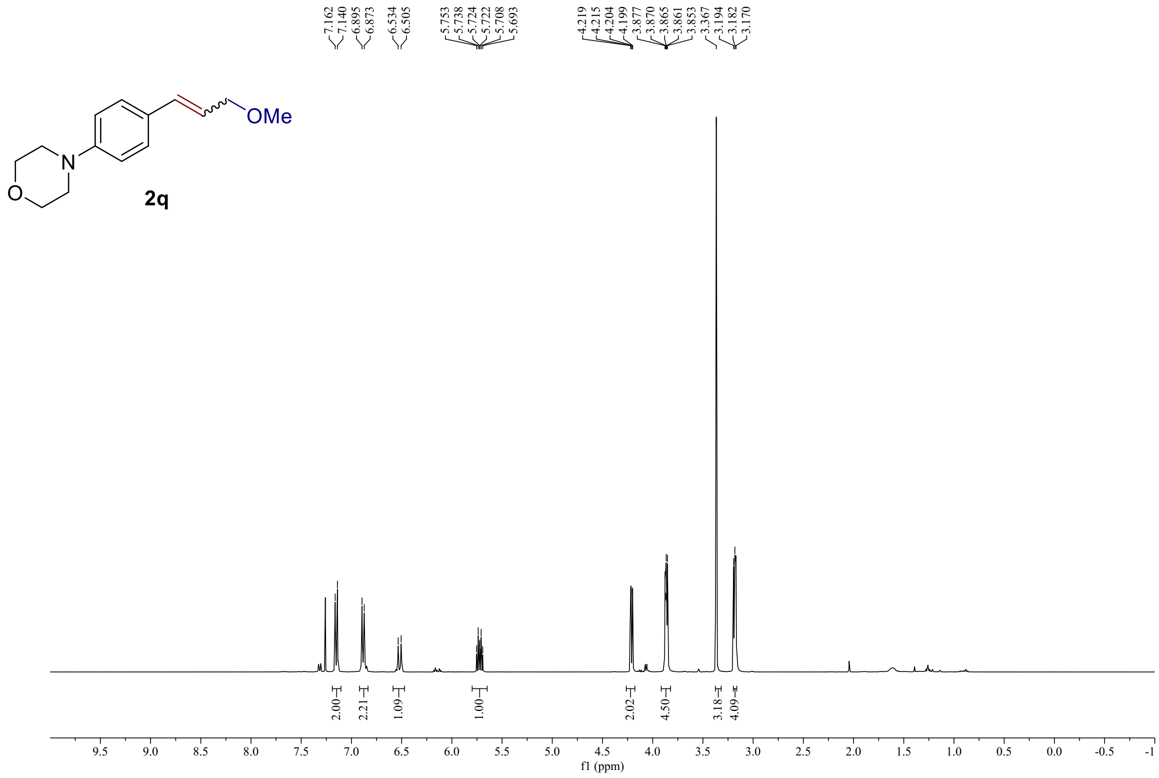

Supplementary Figure 50. ^{1}H NMR spectrum of compound **2q**.

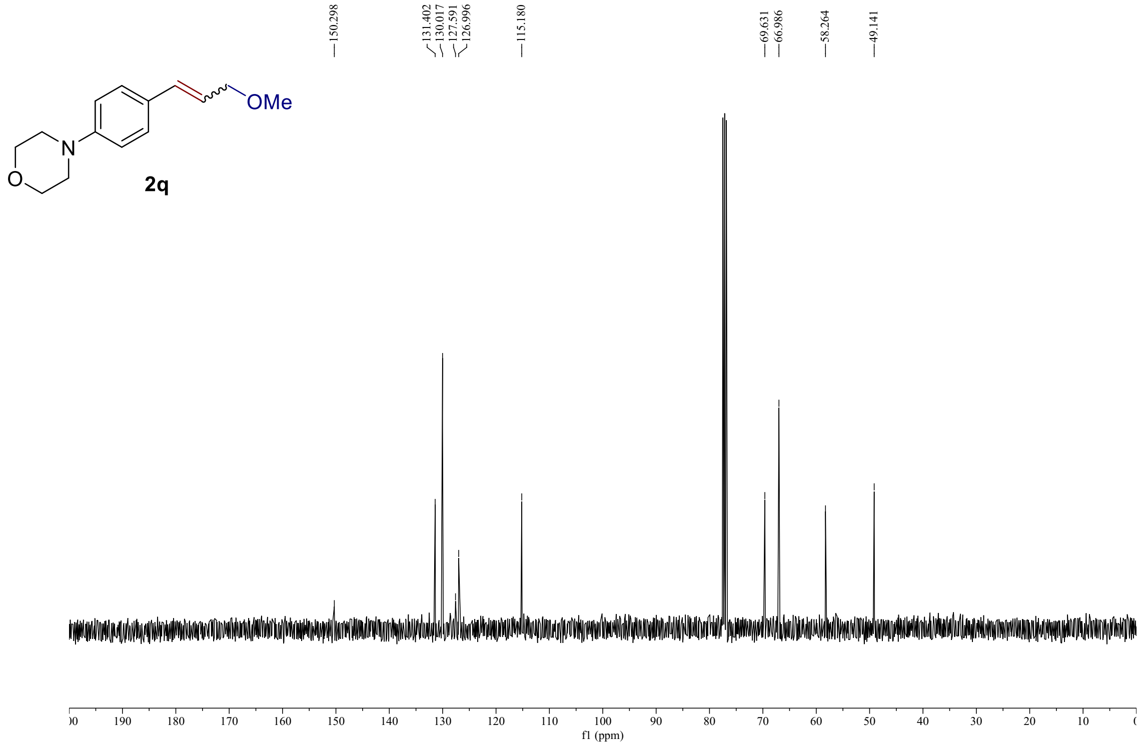

Supplementary Figure 51. ^{13}C{^{1}H} NMR spectrum of compound **2q**.

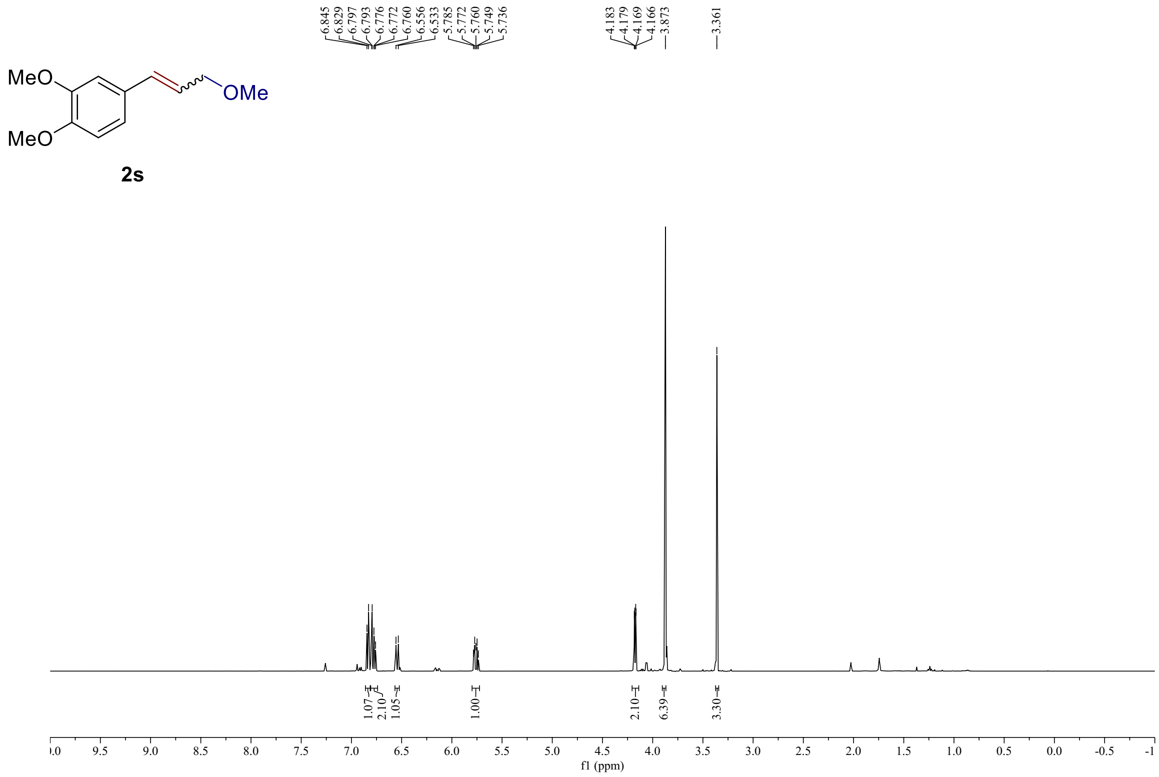

Supplementary Figure 52. ^{1}H NMR spectrum of compound **2s**.

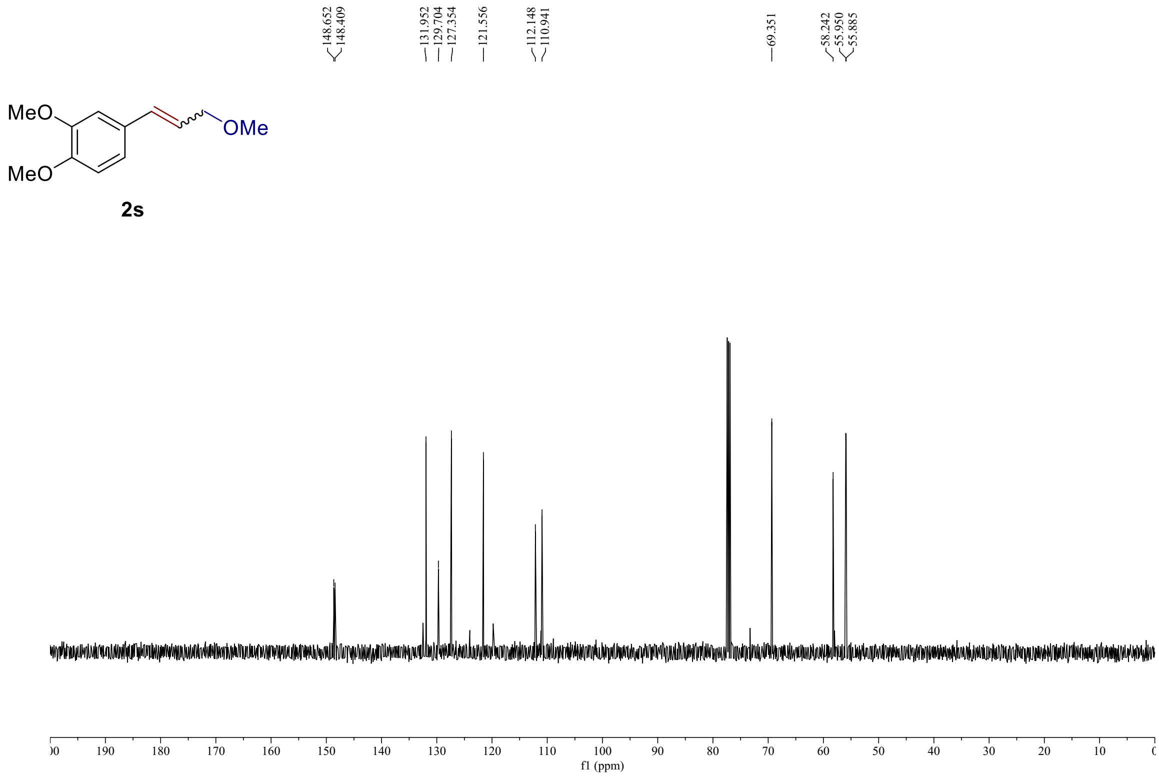

Supplementary Figure 53. $^{13}C\{^{1}H\}$ NMR spectrum of compound **2s**.

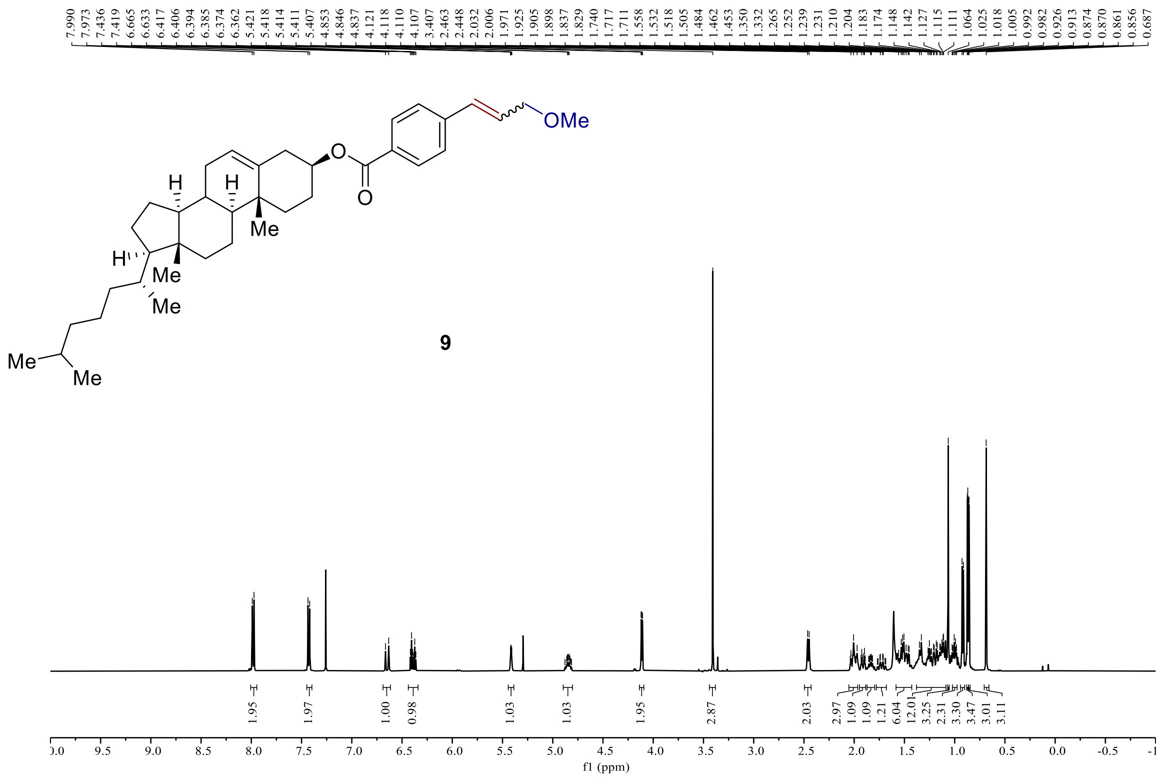

Supplementary Figure 54. ^{1}H NMR spectrum of compound **9**.

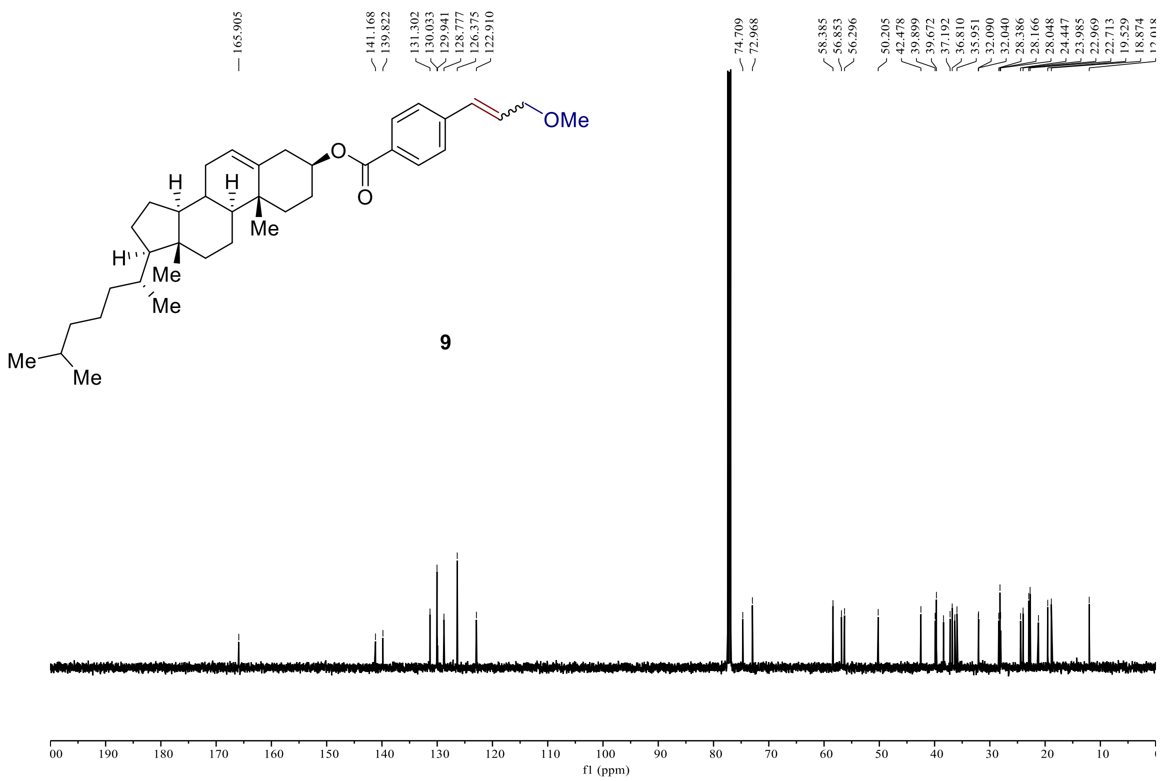

Supplementary Figure 55. ^{13}C{^{1}H} NMR spectrum of compound **9**.

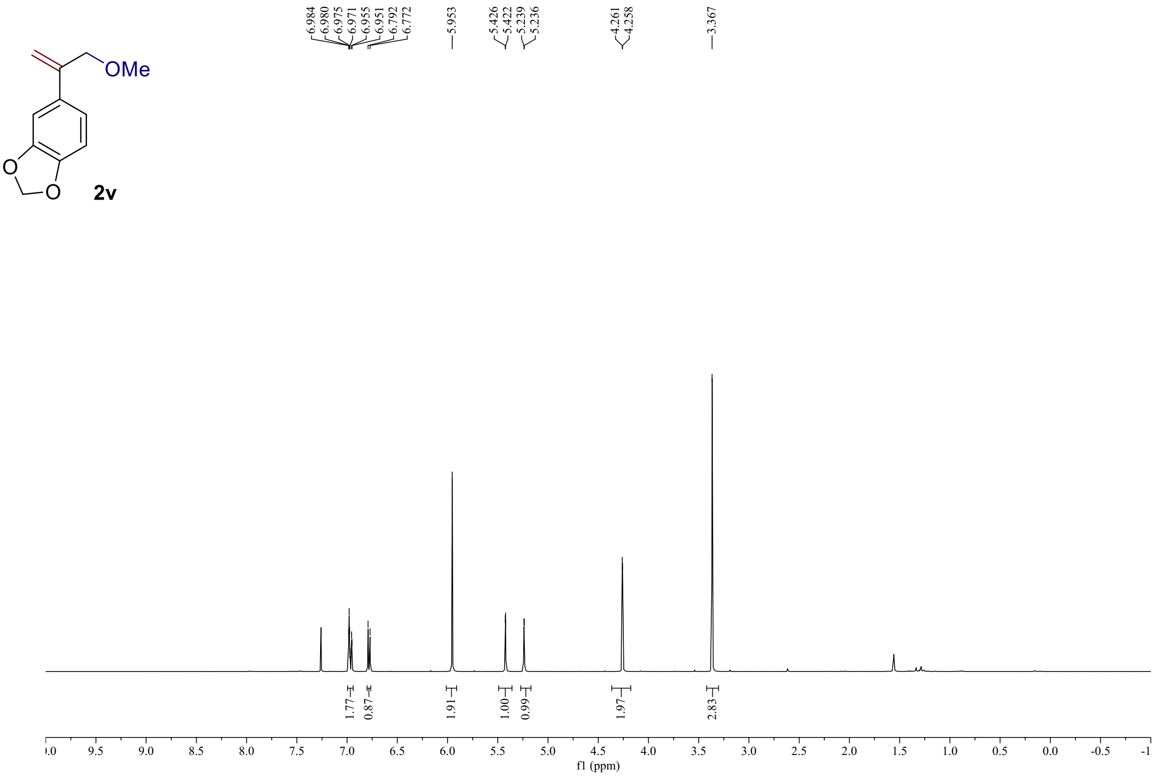

Supplementary Figure 56. ^{1}H NMR spectrum of compound **2v**.

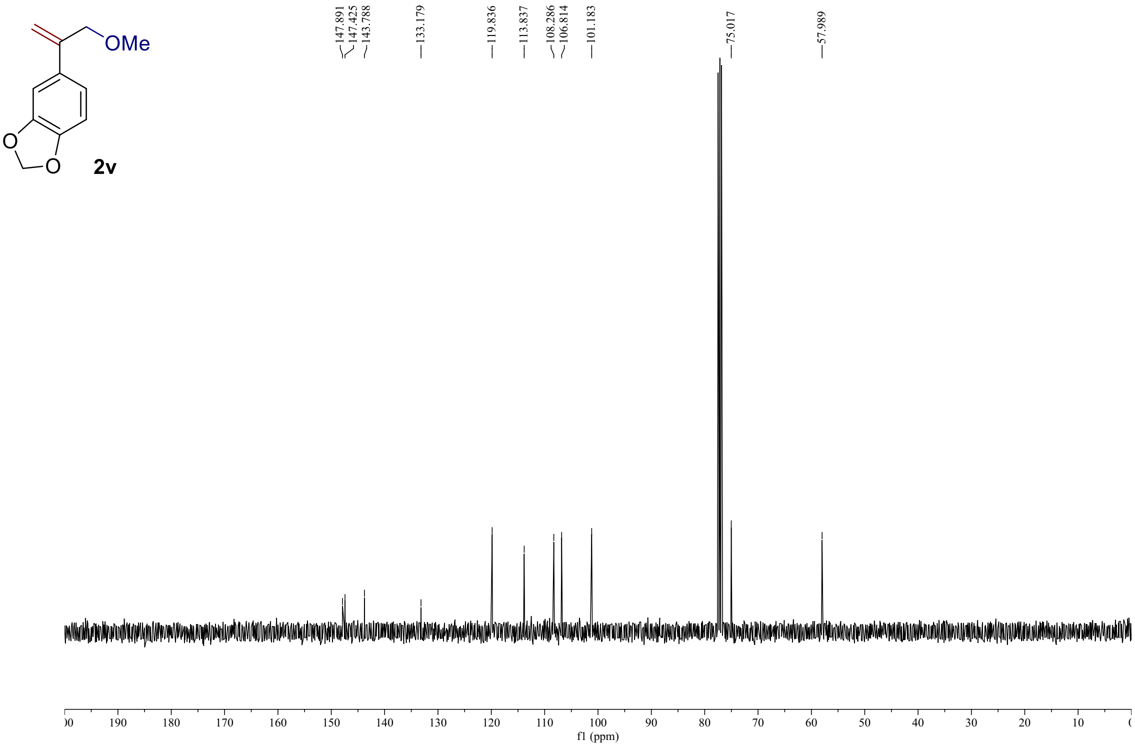

Supplementary Figure 57. $^{13}C\{^{1}H\}$ NMR spectrum of compound **2v**.

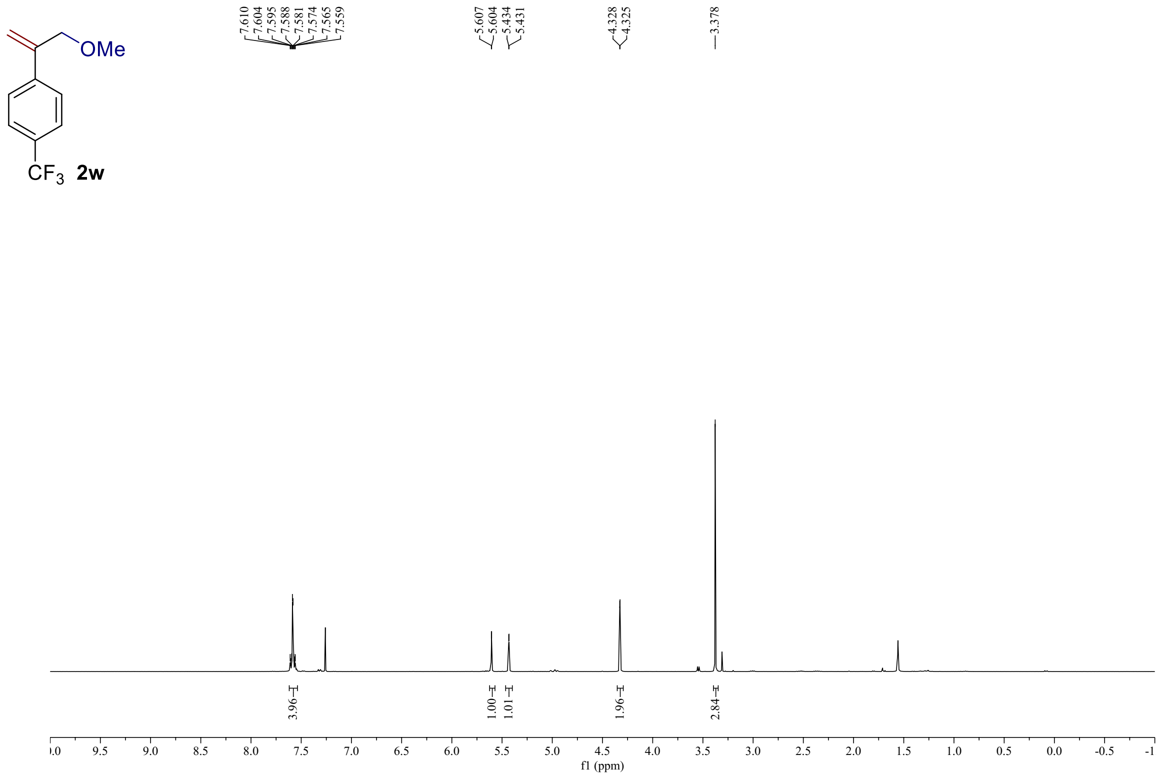

Supplementary Figure 58. ^{1}H NMR spectrum of compound **2w**.

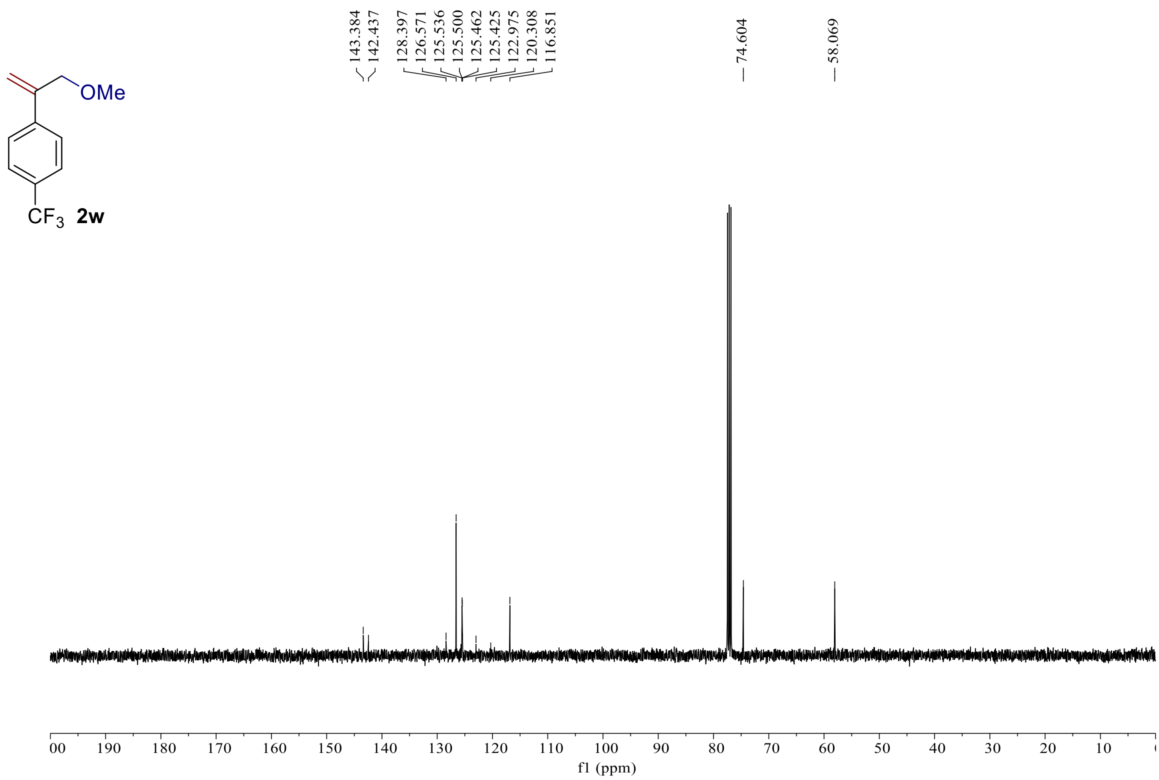

Supplementary Figure 59. ^{13}C{^{1}H} NMR spectrum of compound **2w**.

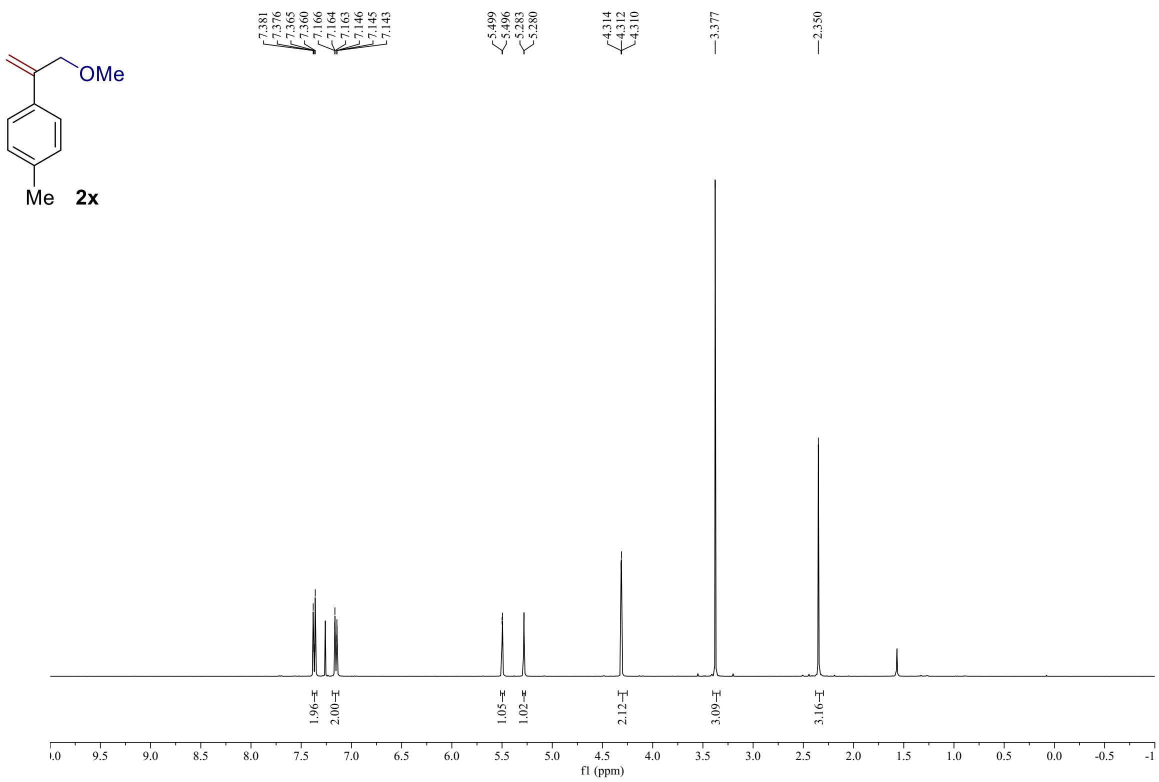

Supplementary Figure 60. ^{1}H NMR spectrum of compound **2x**.

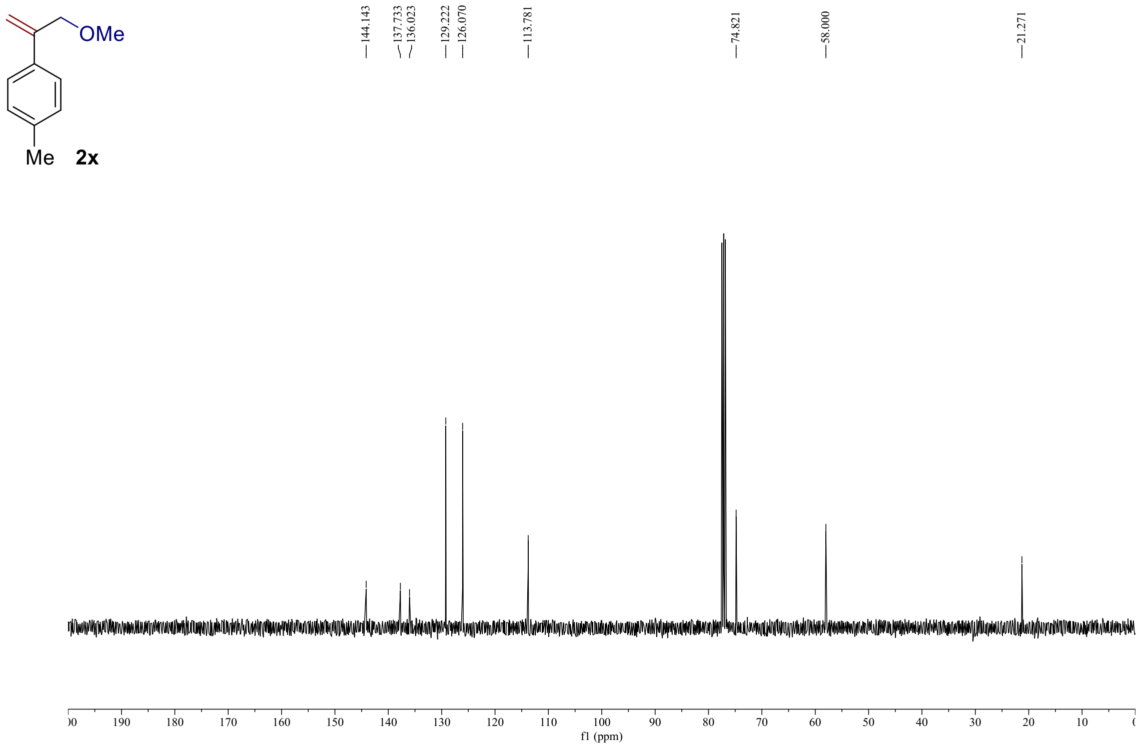

Supplementary Figure 61. $^{13}C\{^{1}H\}$ NMR spectrum of compound **2x**.

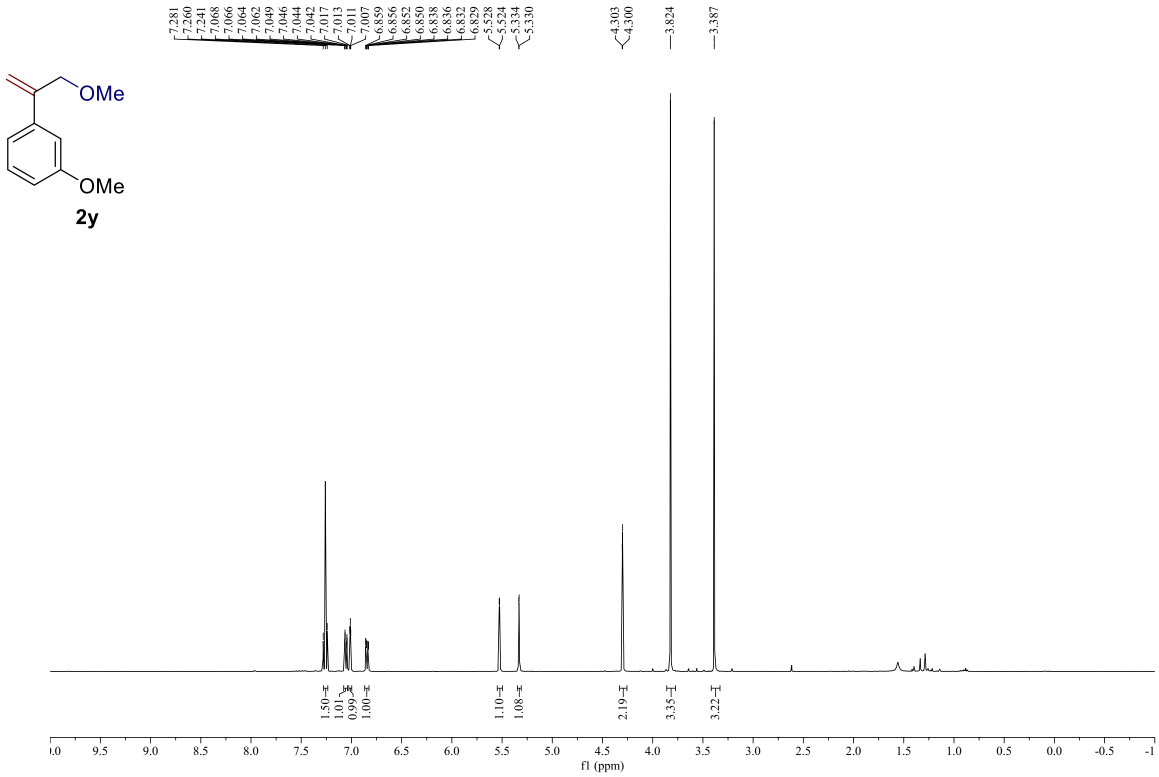

Supplementary Figure 62. ^{1}H NMR spectrum of compound **2y**.

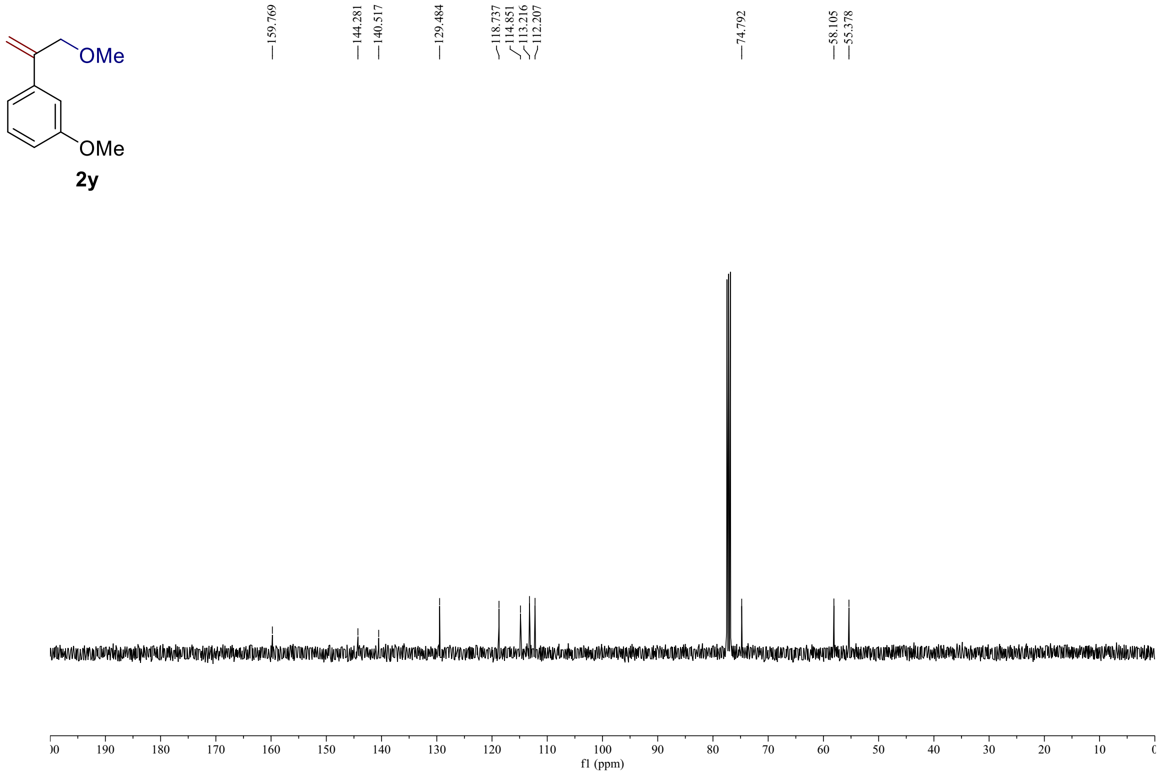

Supplementary Figure 63. $^{13}C\{^{1}H\}$ NMR spectrum of compound **2y**.

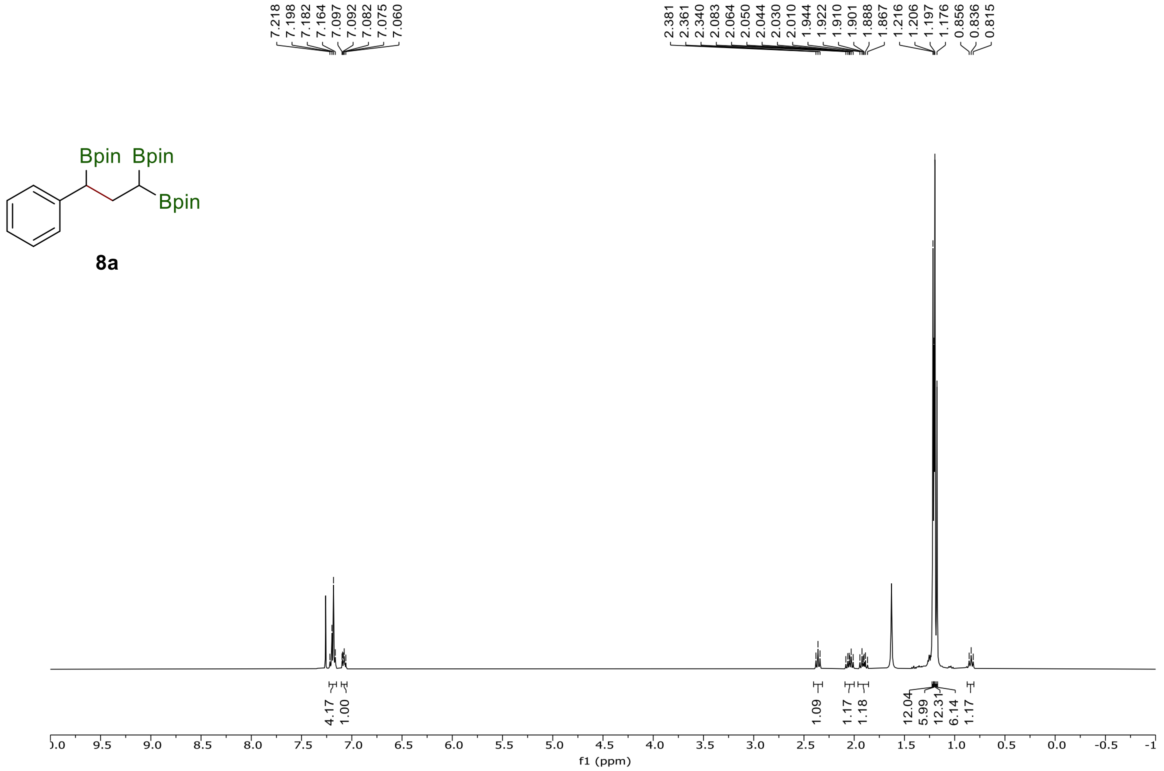

Supplementary Figure 64. ^{1}H NMR spectrum of compound **8a**.

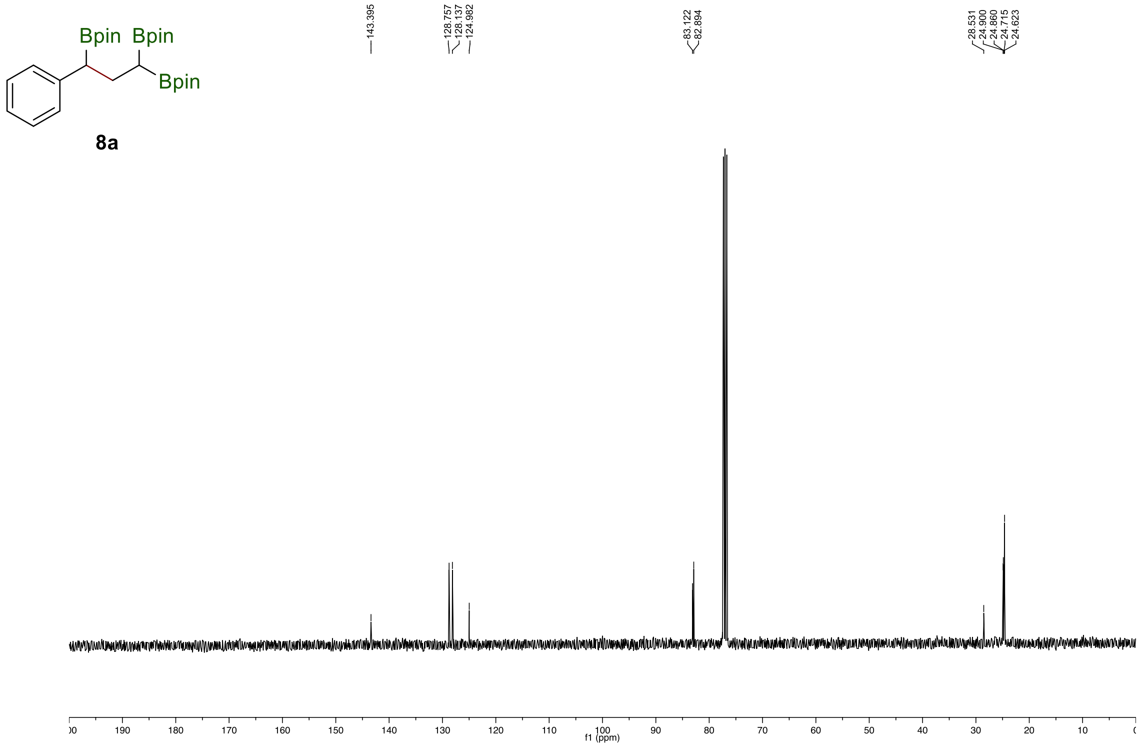

Supplementary Figure 65. $^{13}C\{^{1}H\}$ NMR spectrum of compound **8a**.

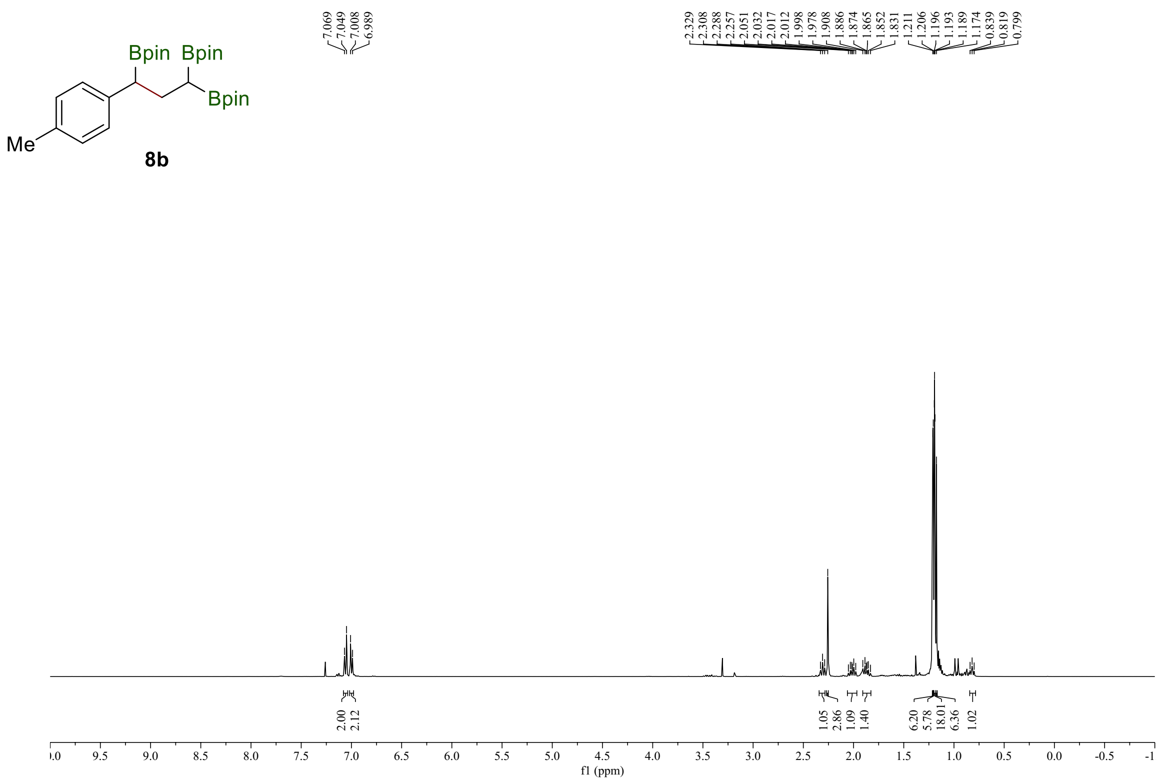

Supplementary Figure 66. ^{1}H NMR spectrum of compound **8b**.

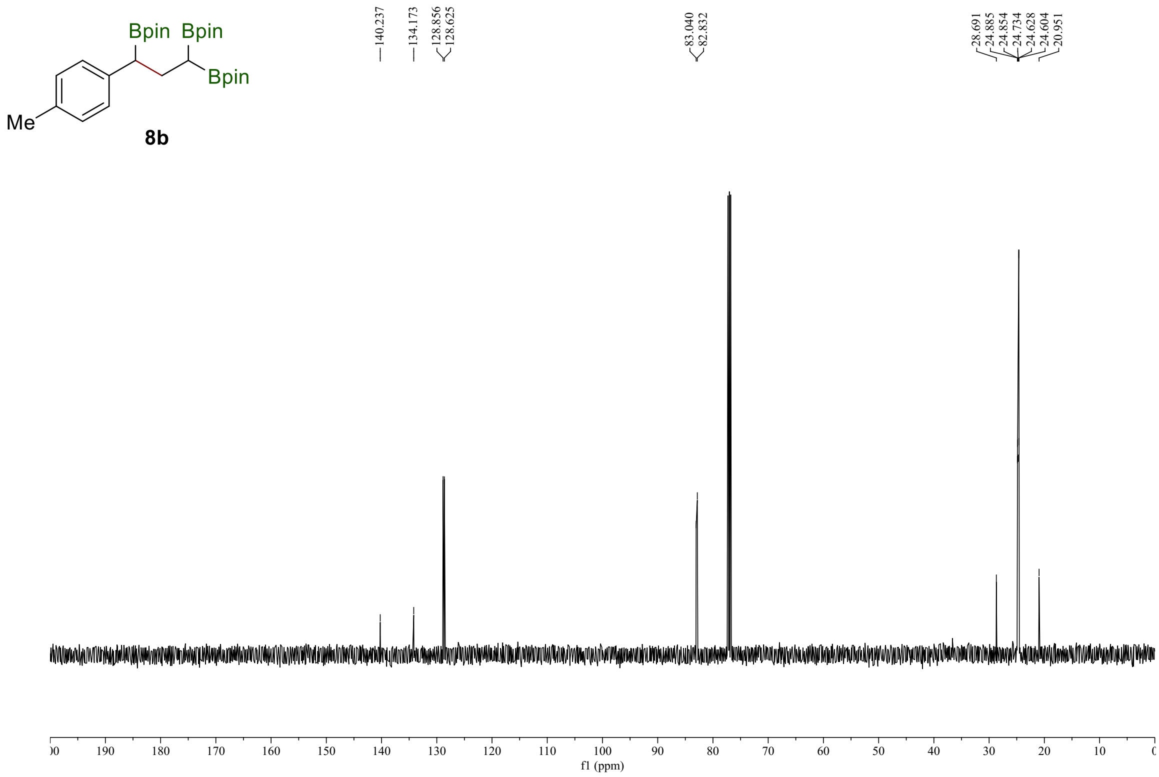

Supplementary Figure 67. ^{13}C{^{1}H} NMR spectrum of compound **8b**.

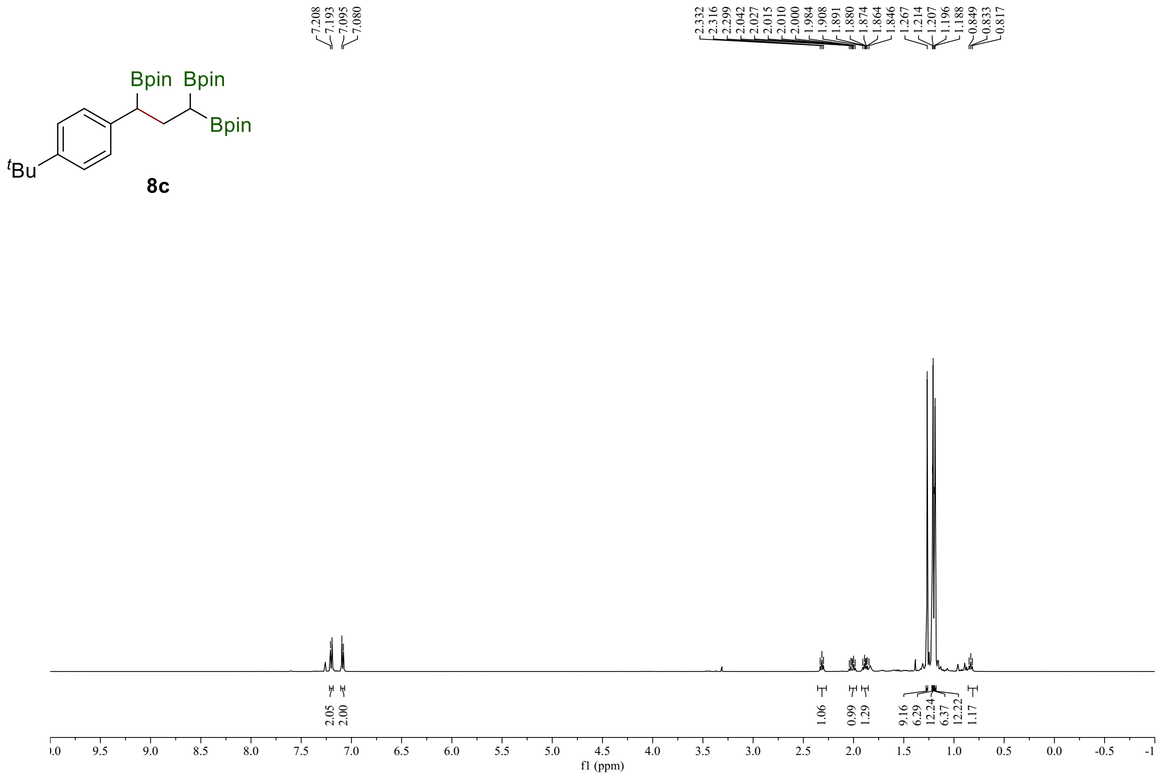

Supplementary Figure 68. ^{1}H NMR spectrum of compound **8c**.

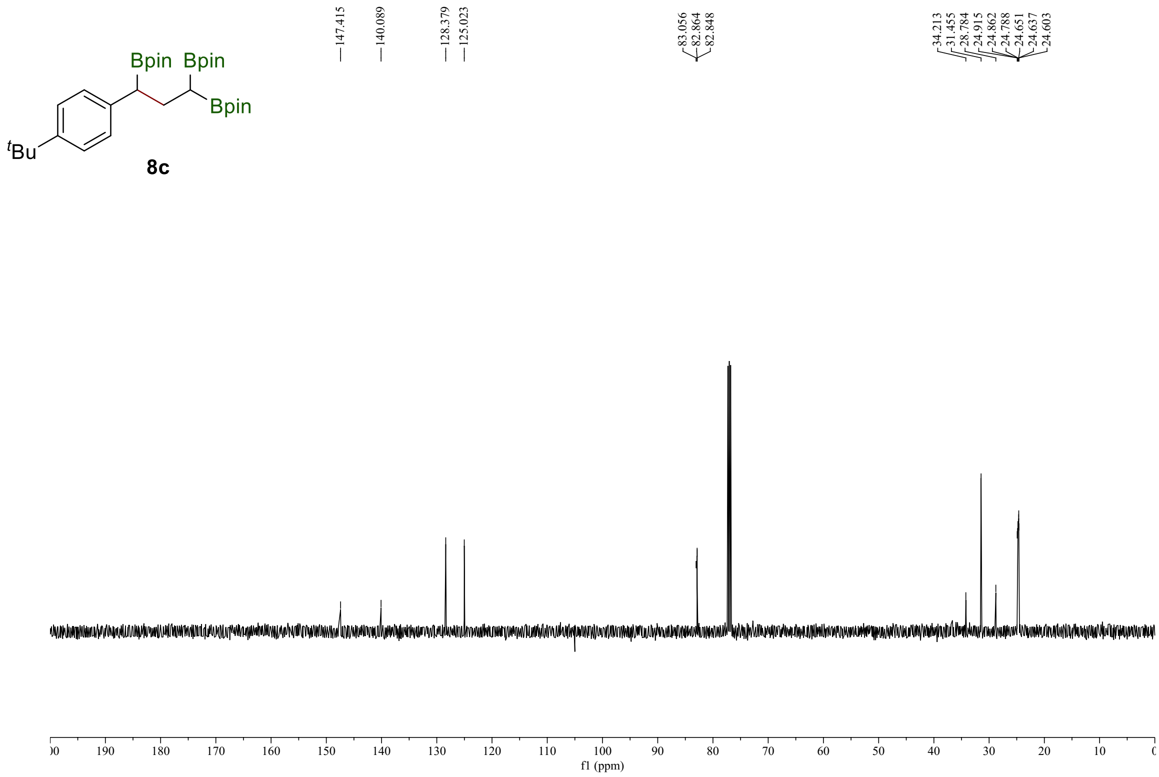

Supplementary Figure 69. ^{13}C{^{1}H} NMR spectrum of compound **8c**.

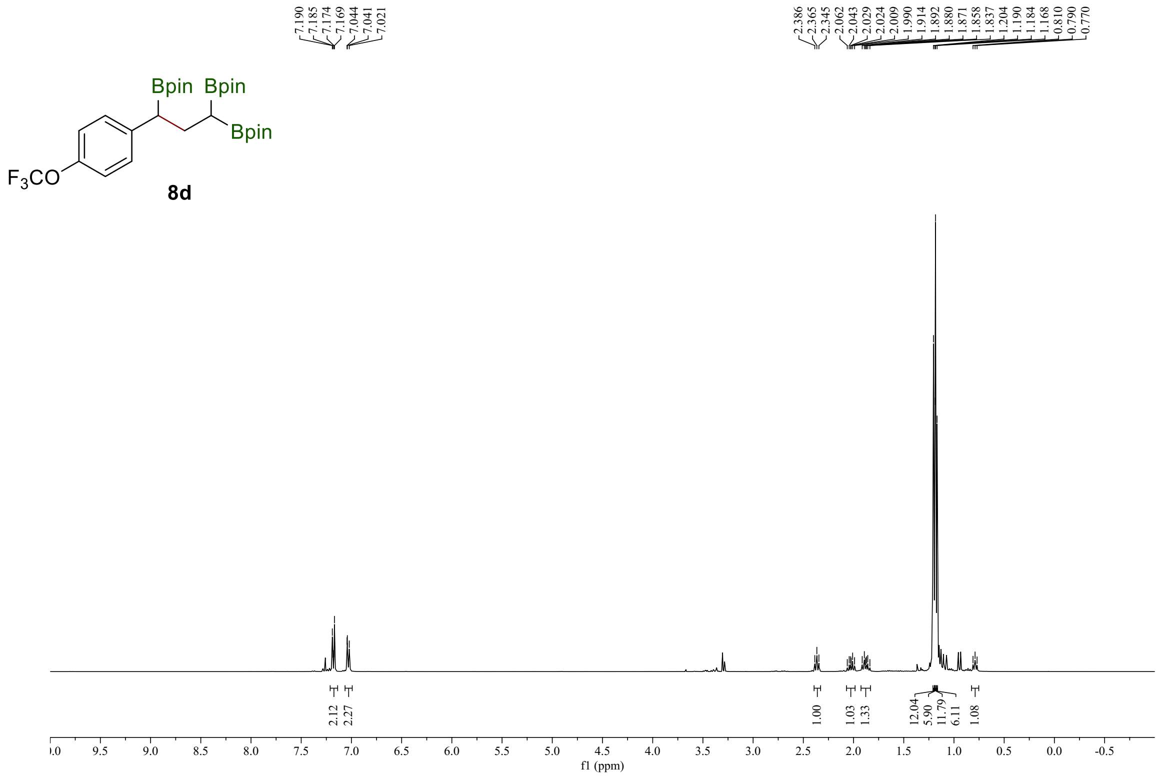

Supplementary Figure 70 ^{1}H NMR spectrum of compound **8d**.

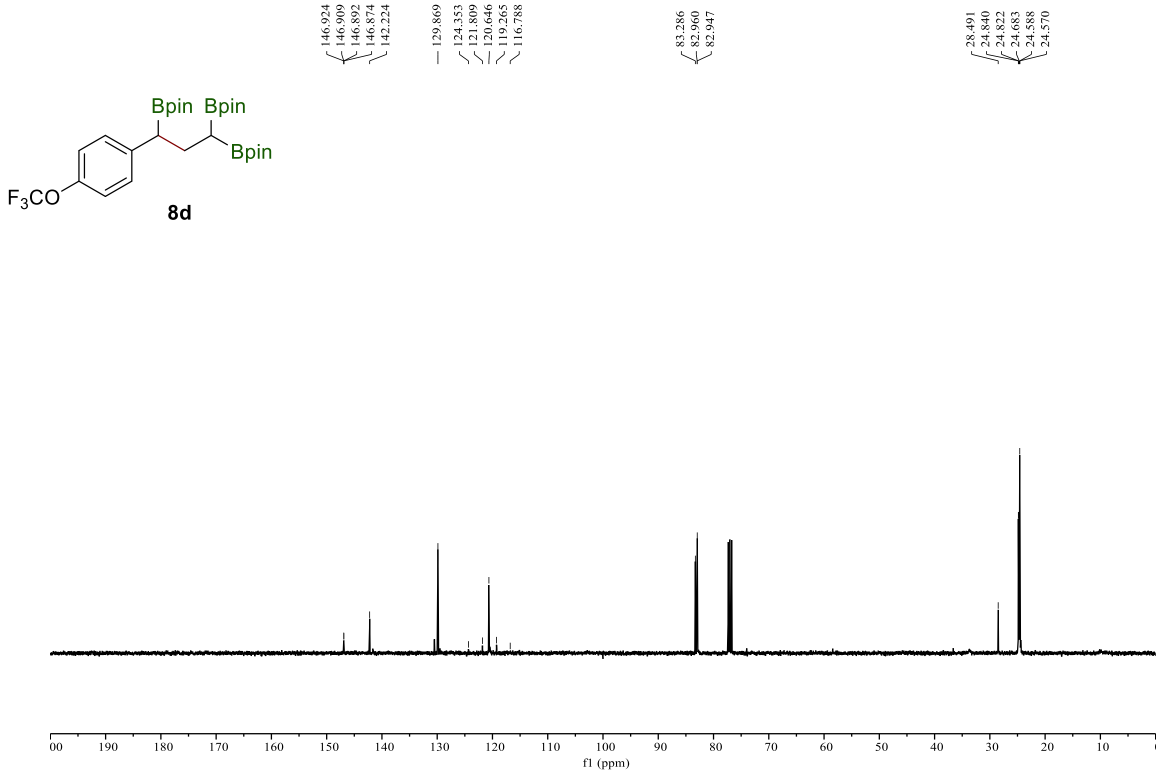

Supplementary Figure 71. ^{13}C{^{1}H} NMR spectrum of compound **8d**.

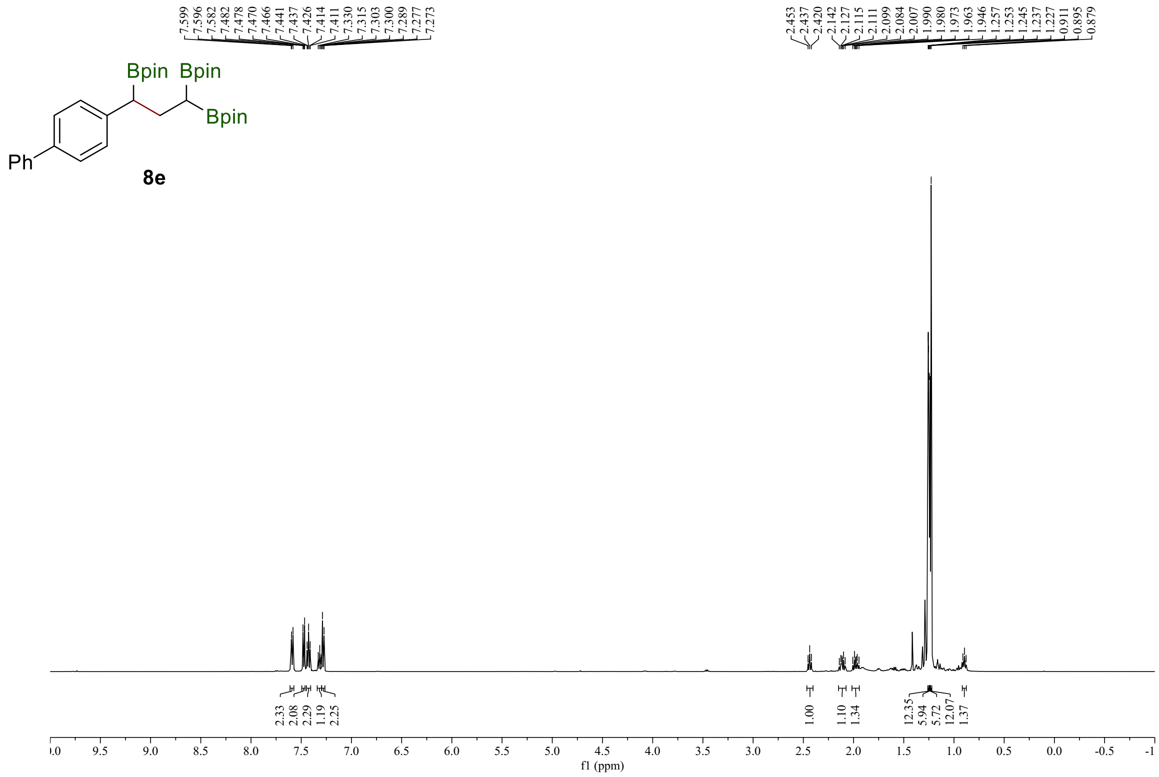

Supplementary Figure 72. ^{1}H NMR spectrum of compound **8e**.

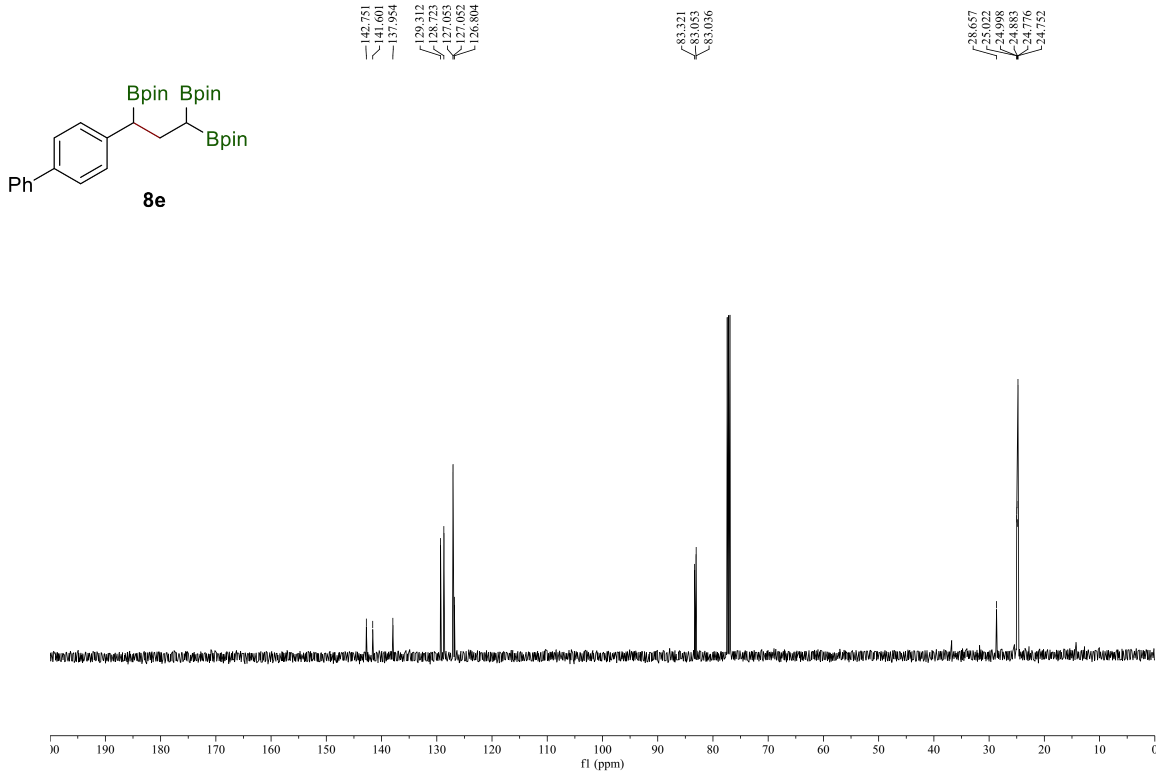

Supplementary Figure 73. ^{13}C{^{1}H} NMR spectrum of compound **8e**.

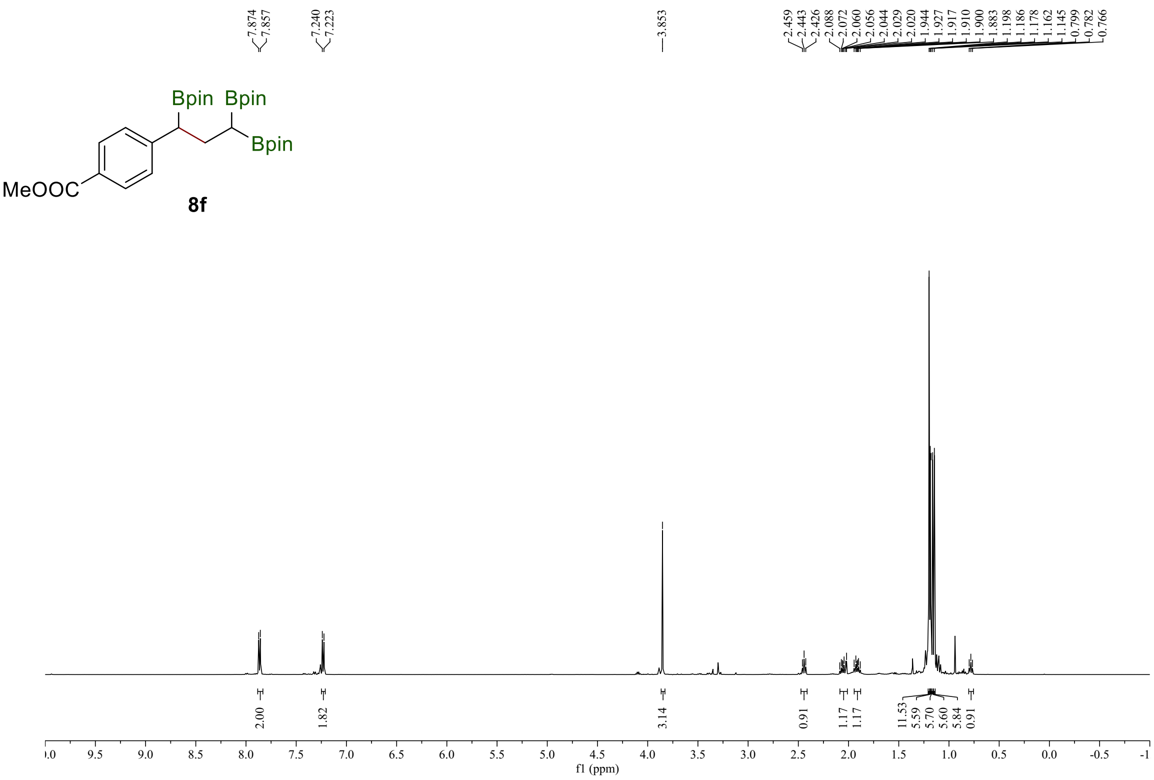

Supplementary Figure 74. ^{1}H NMR spectrum of compound **8f**.

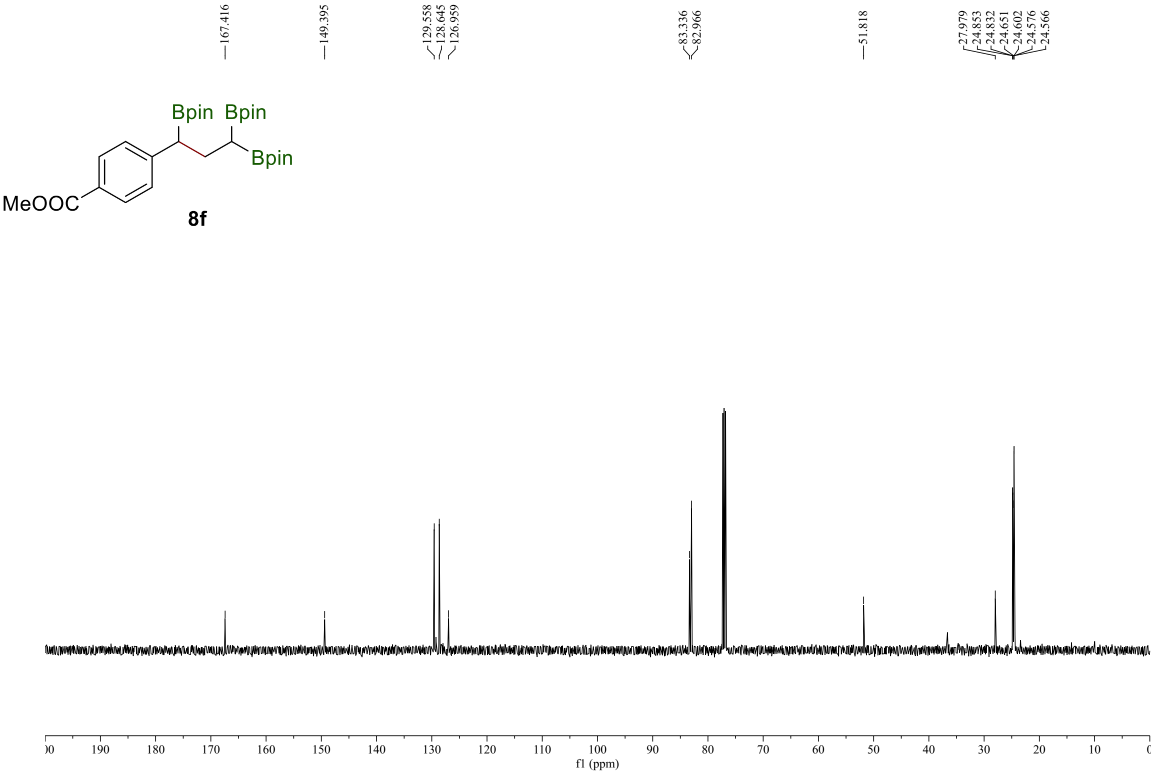

Supplementary Figure 75. $^{13}C\{^{1}H\}$ NMR spectrum of compound **8f**.

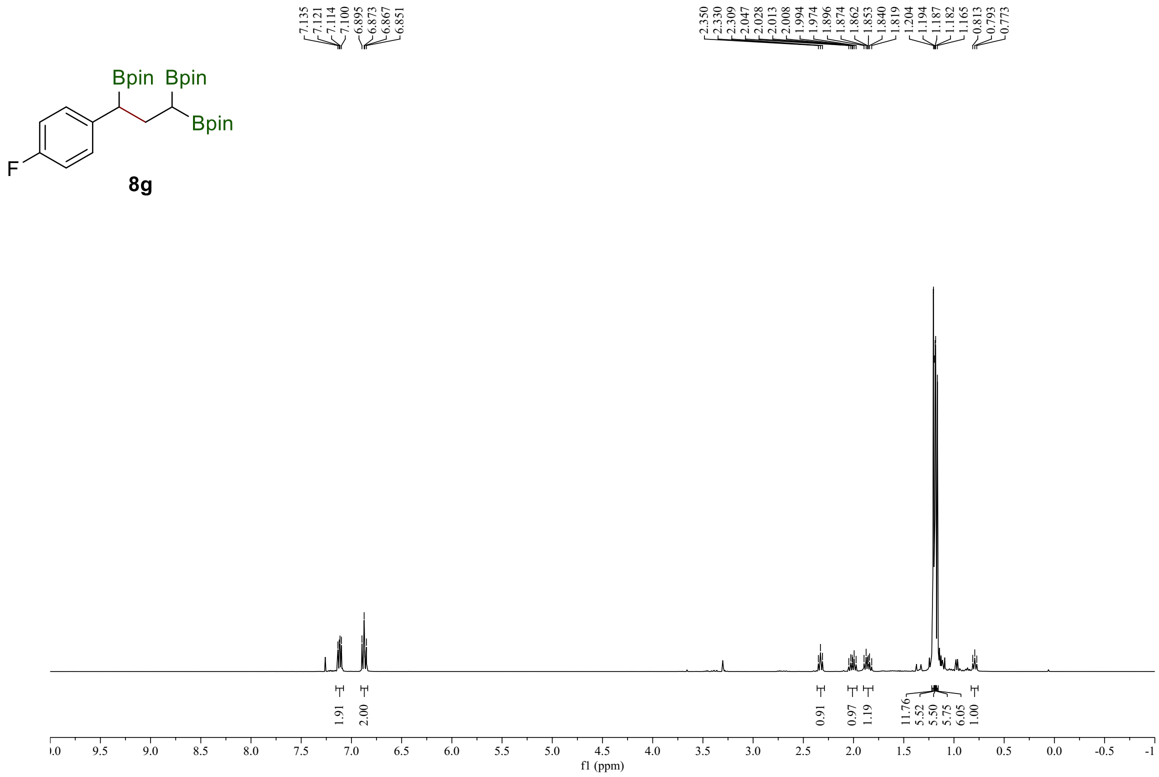

Supplementary Figure 76. ^{1}H NMR spectrum of compound **8g**.

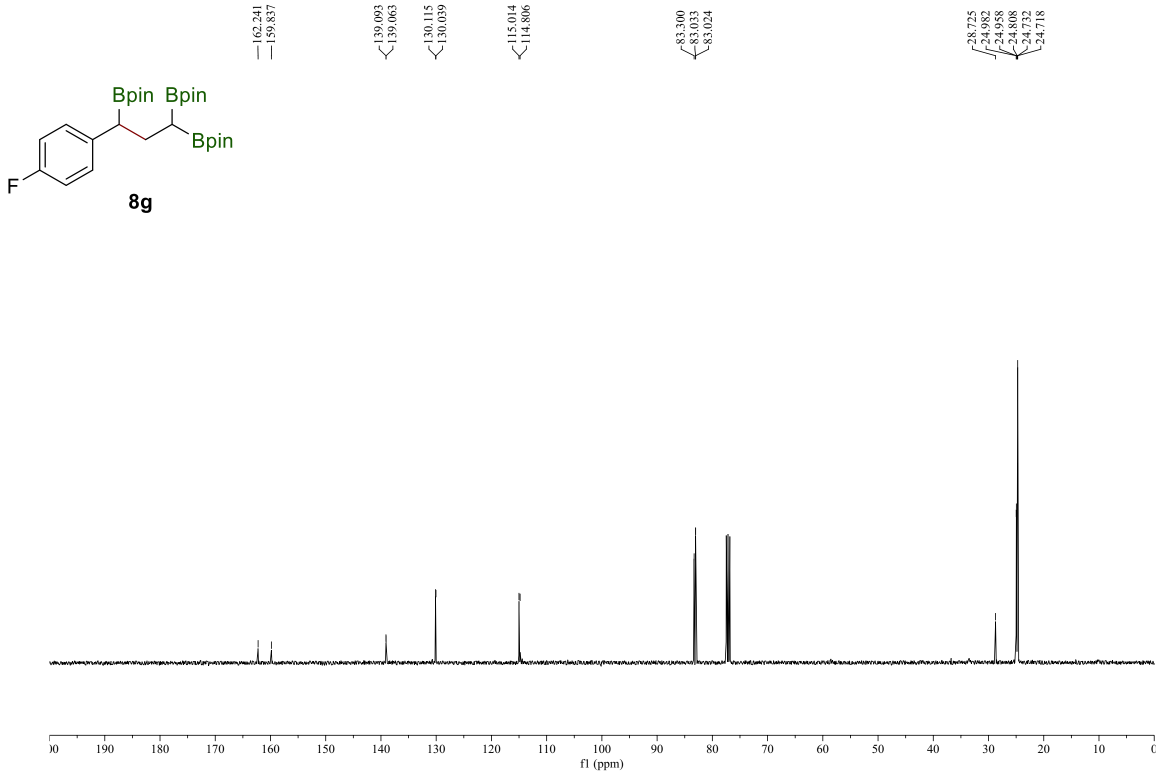

Supplementary Figure 77. $^{13}C\{^{1}H\}$ NMR spectrum of compound **8g**.

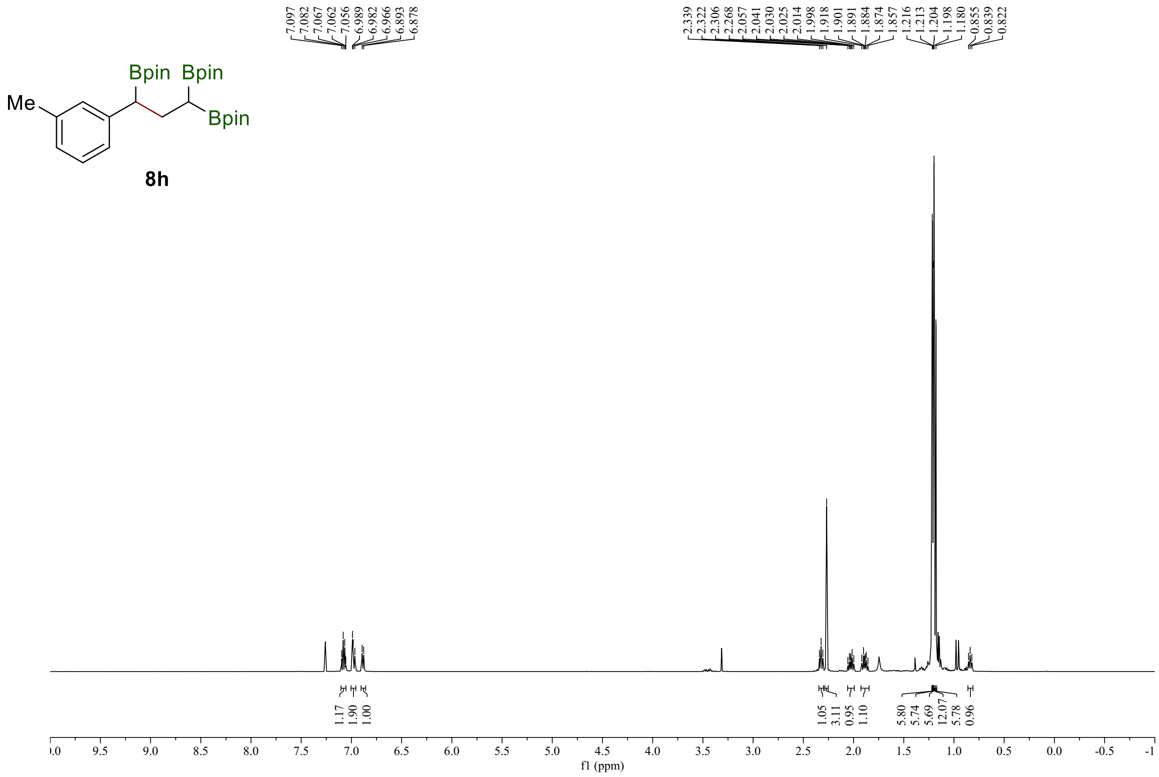

Supplementary Figure 78. ^{1}H NMR spectrum of compound **8h**.

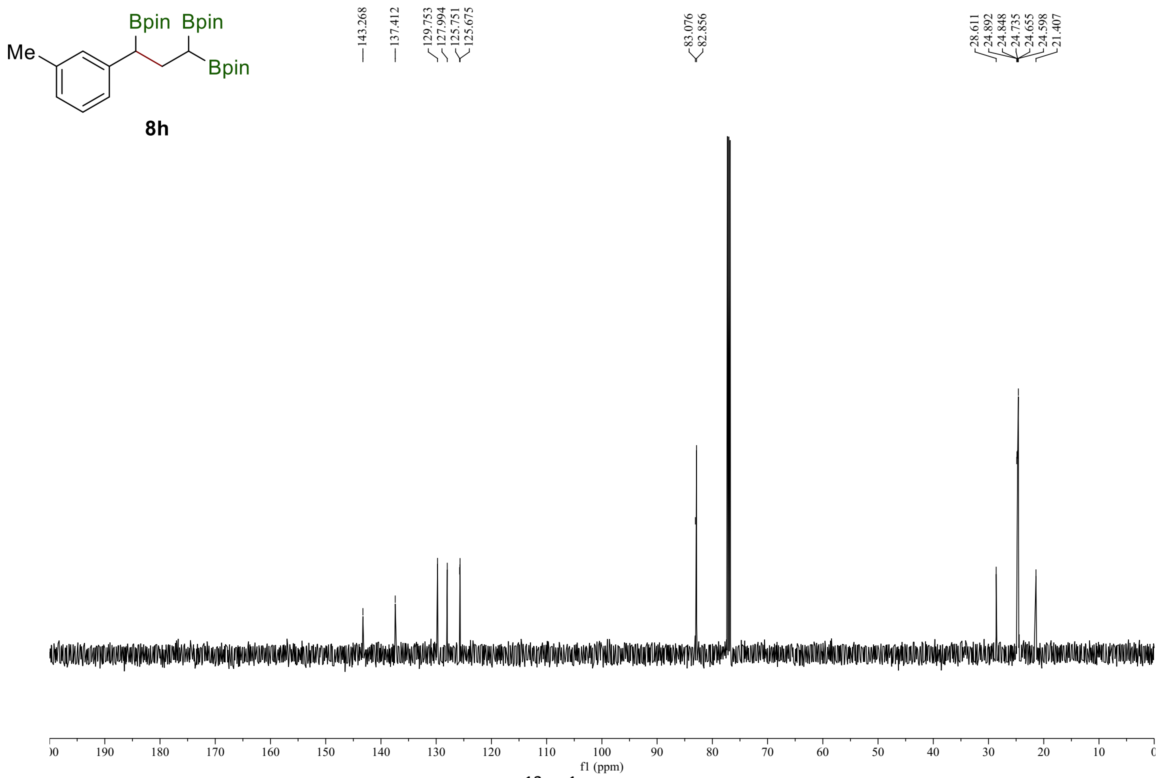

Supplementary Figure 79. $^{13}C\{^{1}H\}$ NMR spectrum of compound **8h**.

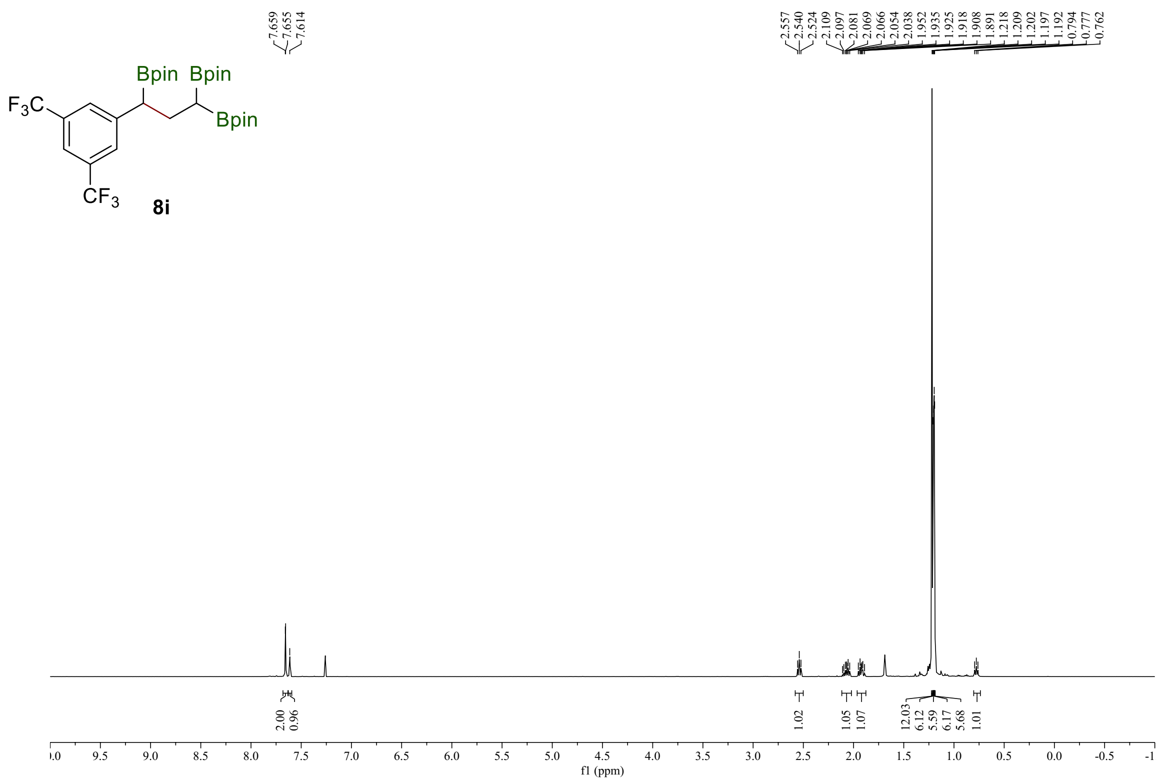

Supplementary Figure 80. ^{1}H NMR spectrum of compound **8i**.

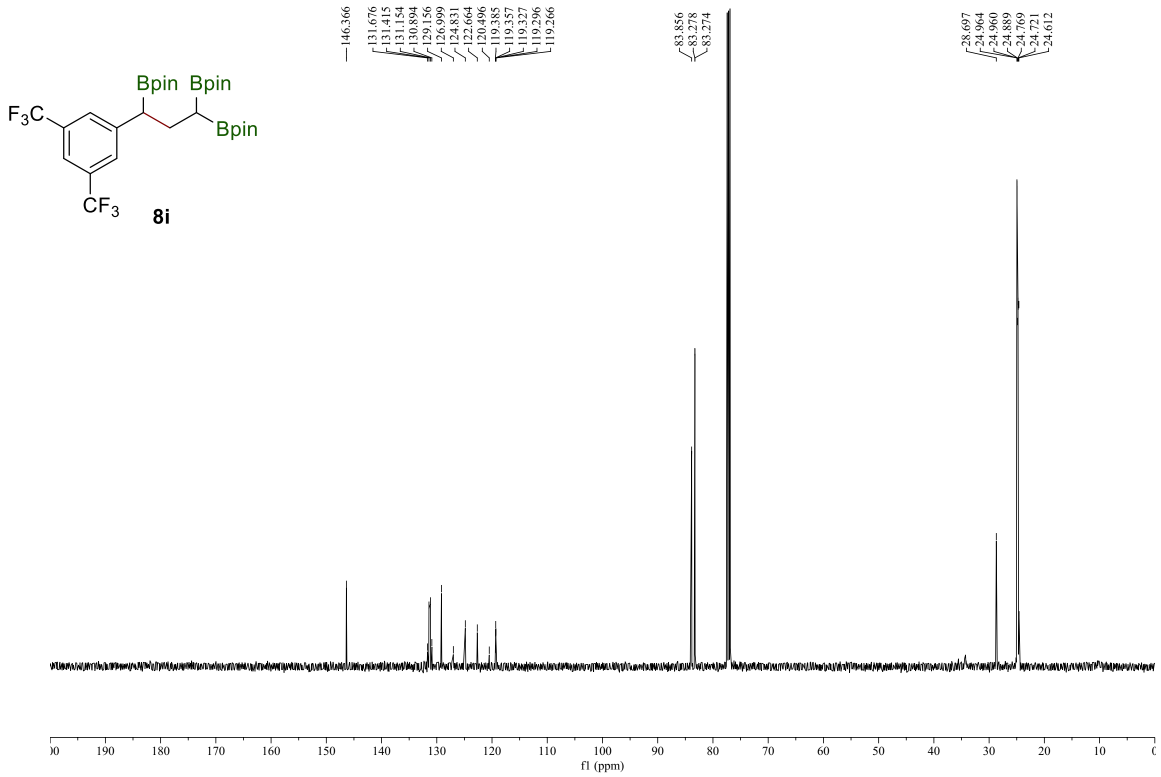

Supplementary Figure 81. ^{13}C{^{1}H} NMR spectrum of compound **8i**.

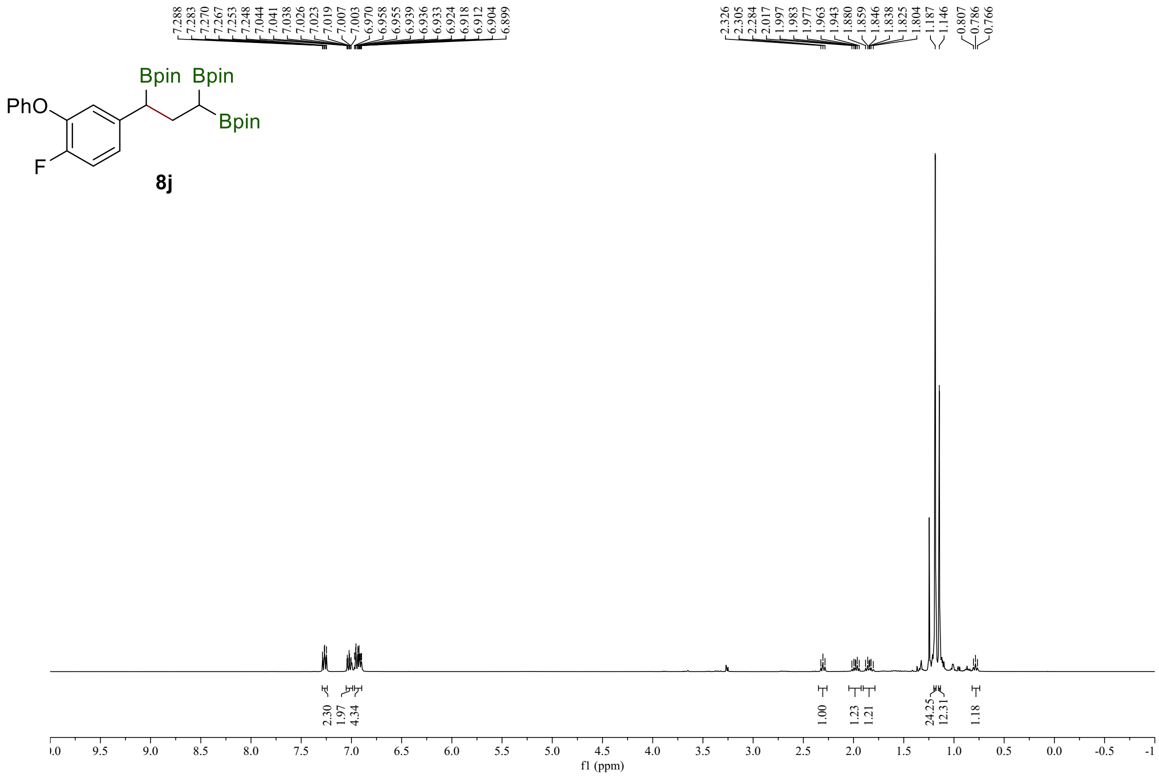

Supplementary Figure 82. ^{1}H NMR spectrum of compound **8j**.

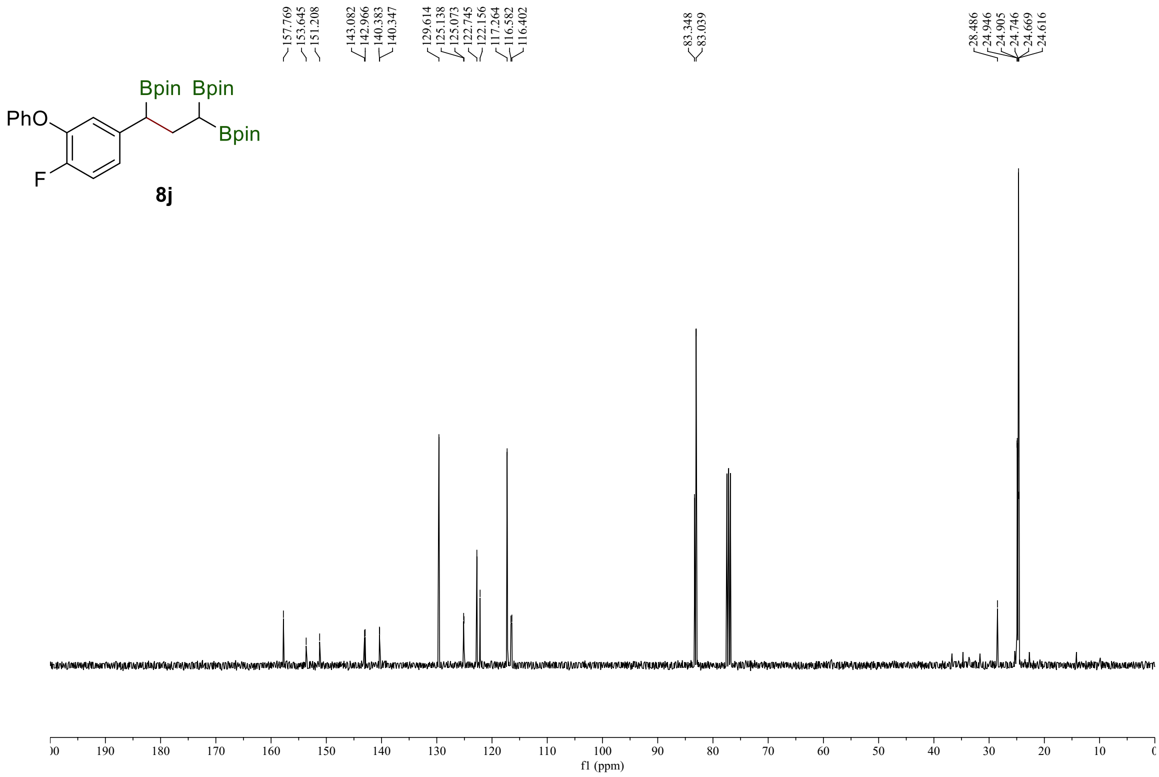

Supplementary Figure 83. ^{13}C{^{1}H} NMR spectrum of compound **8j**.

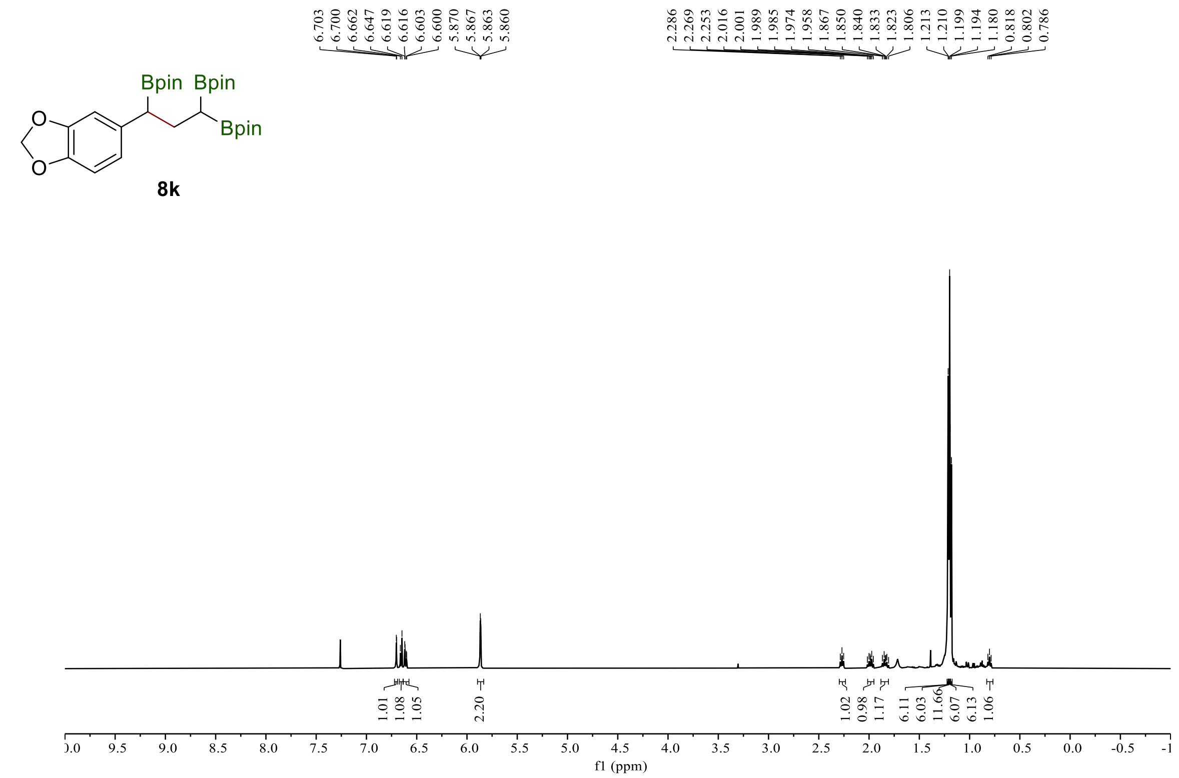

Supplementary Figure 84. ^{1}H NMR spectrum of compound **8k**.

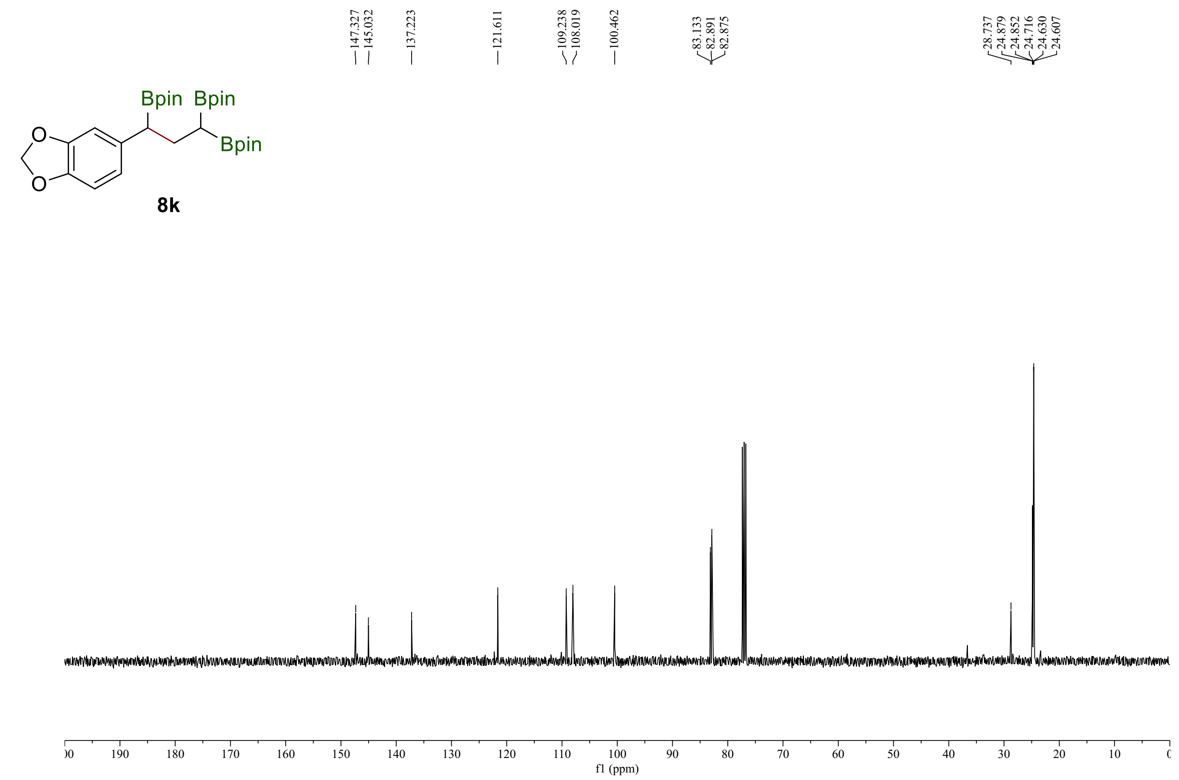

Supplementary Figure 85. ^{13}C{^{1}H} NMR spectrum of compound **8k**.

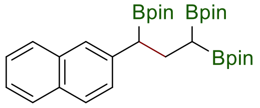

8l

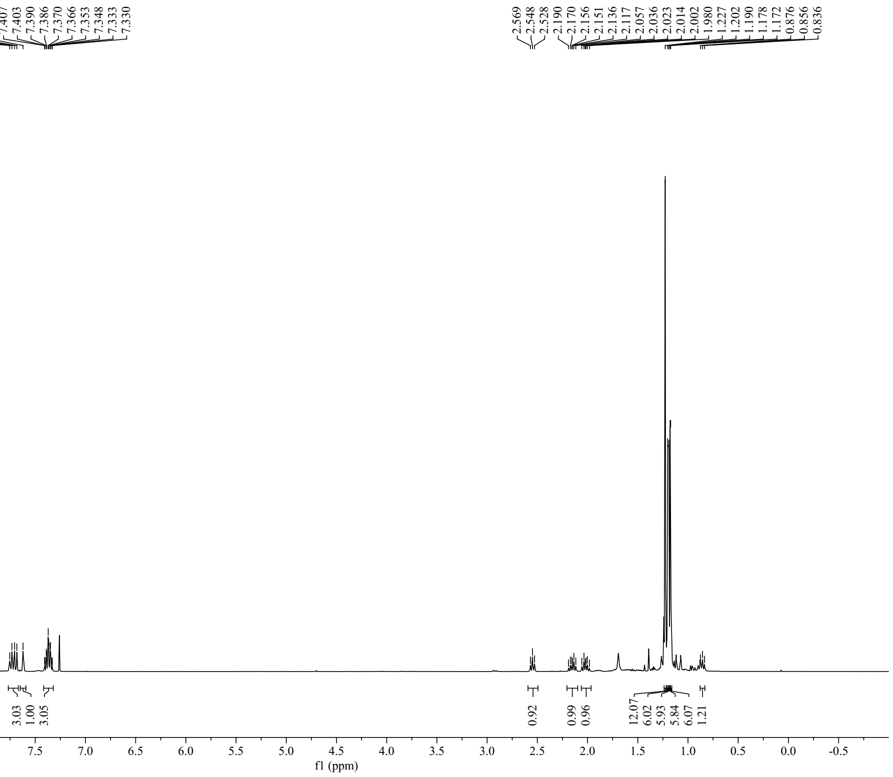

Supplementary Figure 86. ^{1}H NMR spectrum of compound **8l**.

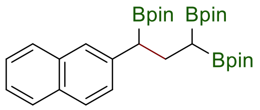

8l

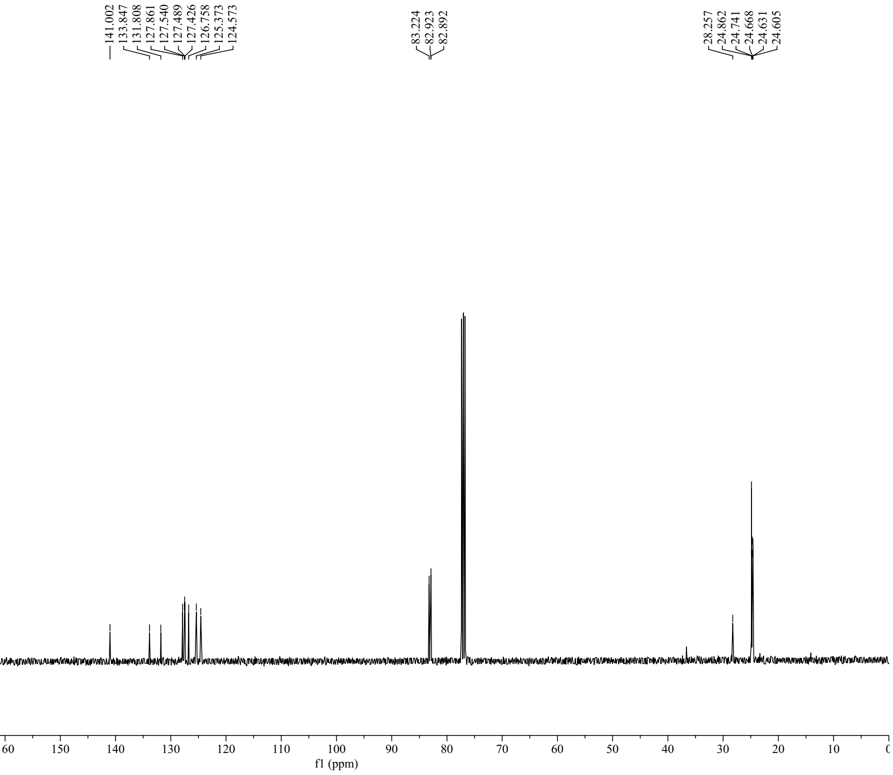

Supplementary Figure 87. $^{13}C\{^{1}H\}$ NMR spectrum of compound **8l**.

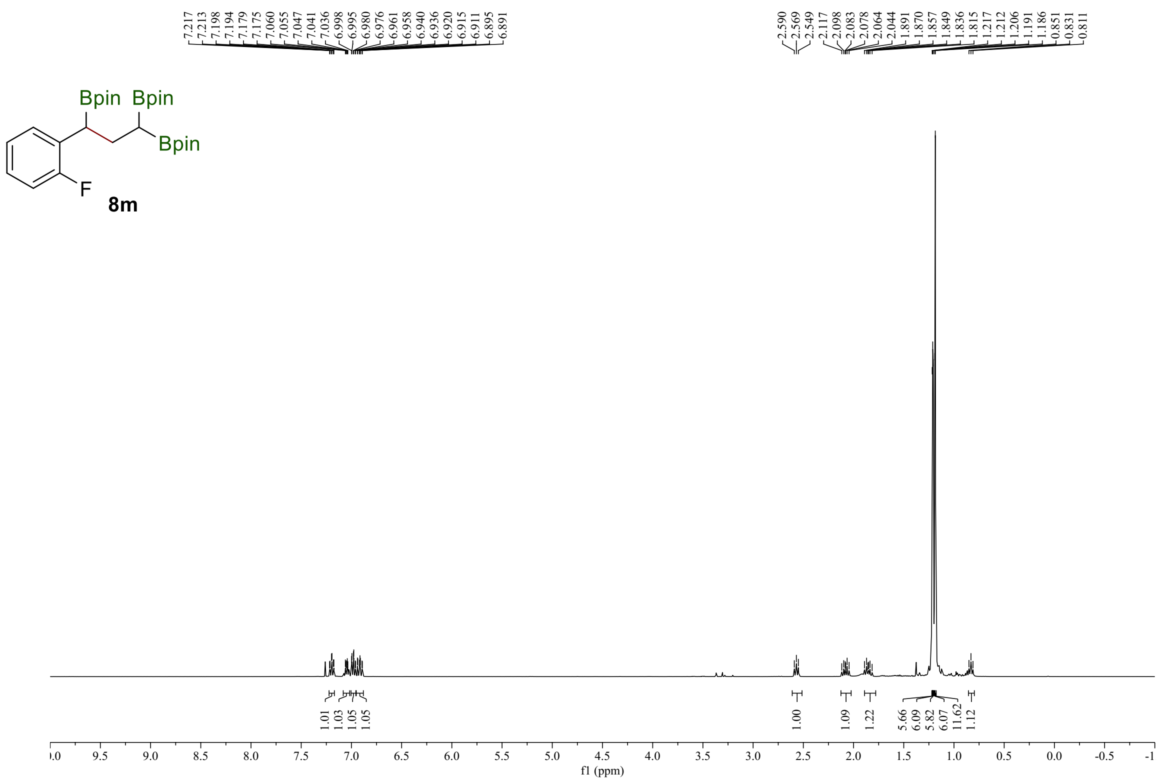

Supplementary Figure 88. ^{1}H NMR spectrum of compound **8m**.

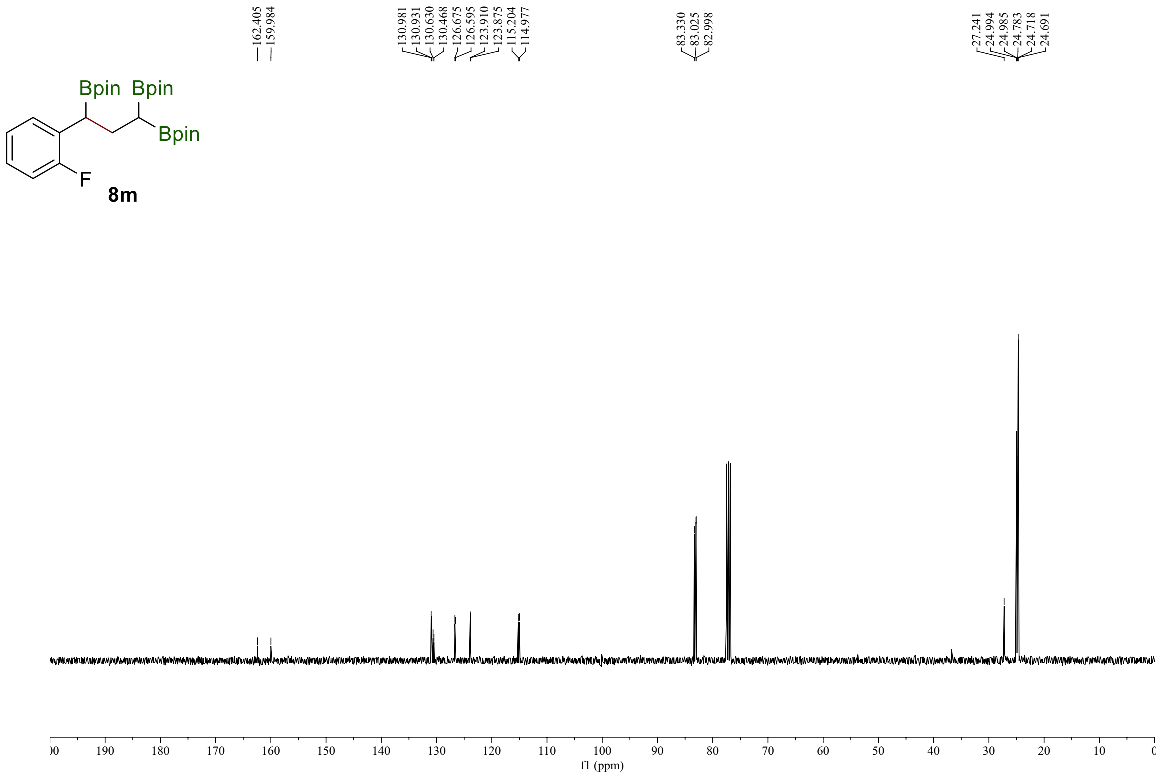

Supplementary Figure 89. ^{13}C{^{1}H} NMR spectrum of compound **8m**.

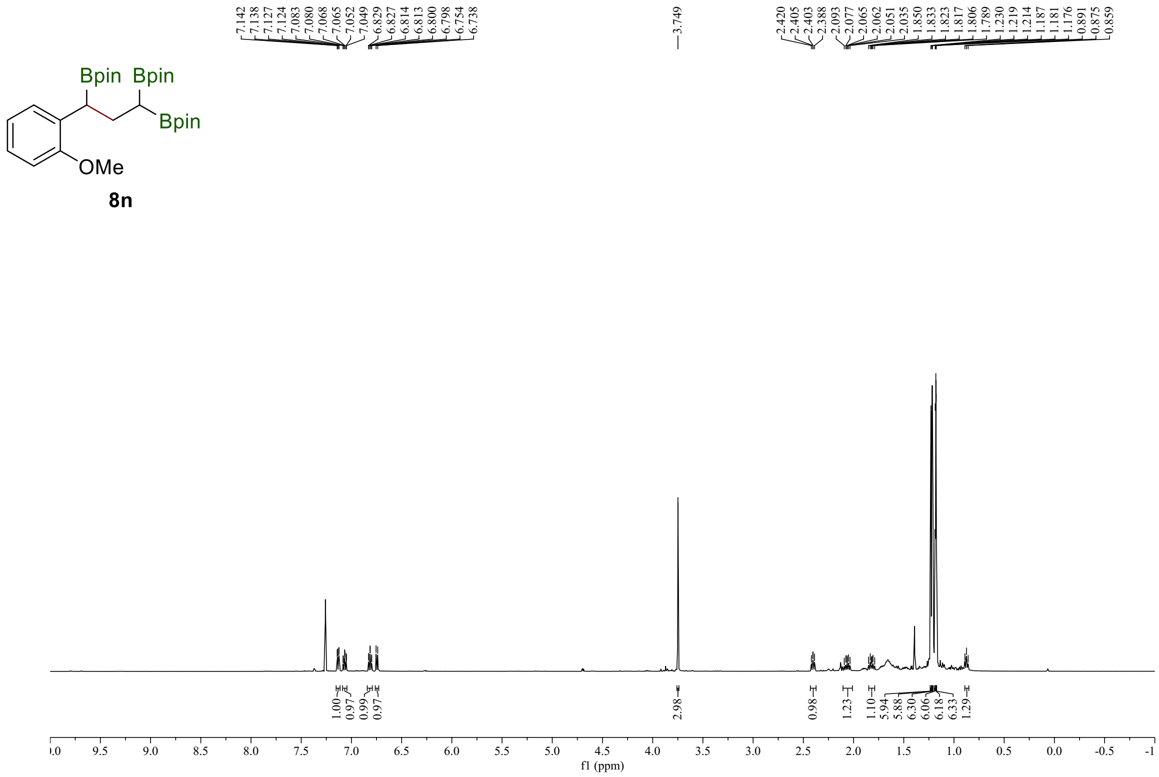

Supplementary Figure 90. ^{1}H NMR spectrum of compound **8n**.

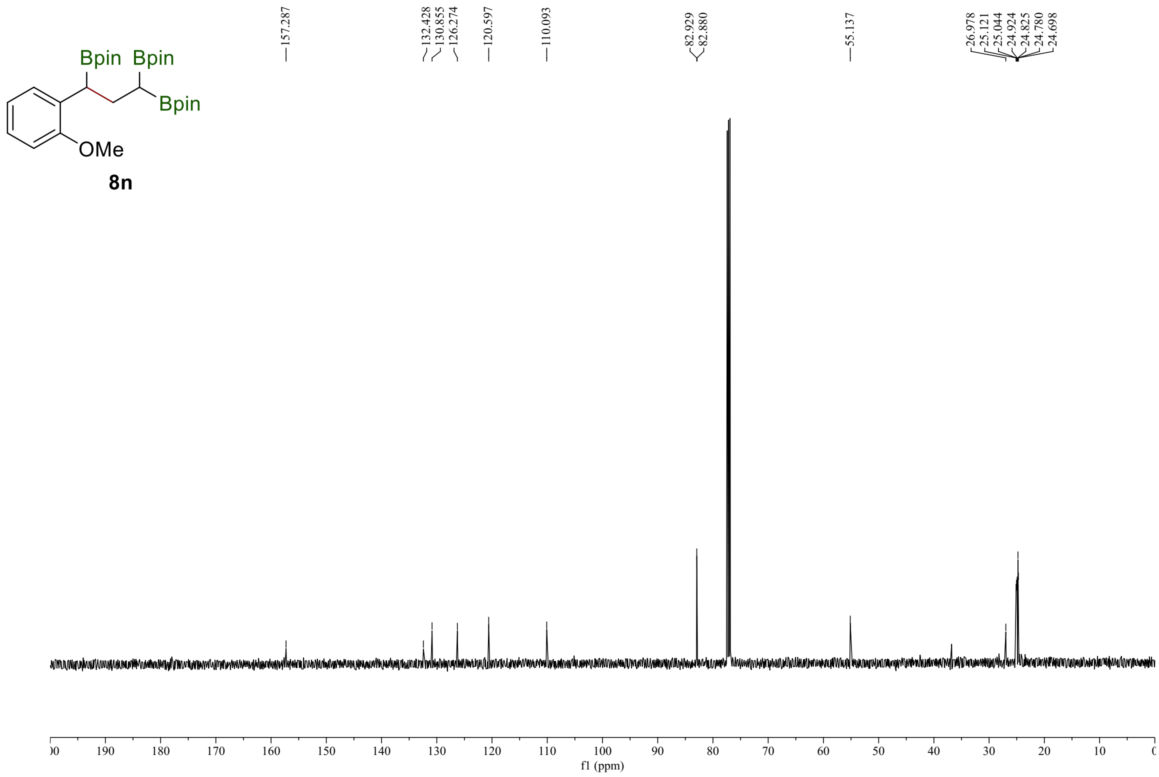

Supplementary Figure 91. $^{13}C\{^{1}H\}$ NMR spectrum of compound **8n**.

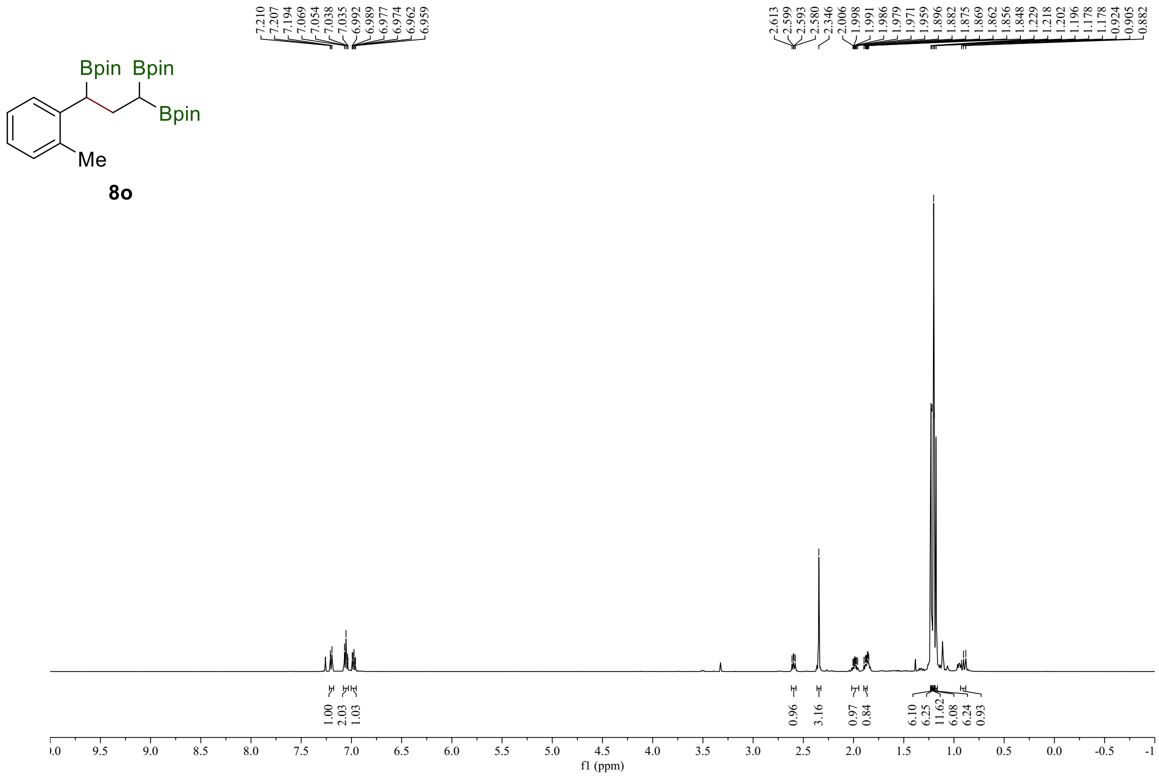

Supplementary Figure 92. ^{1}H NMR spectrum of compound **8o**.

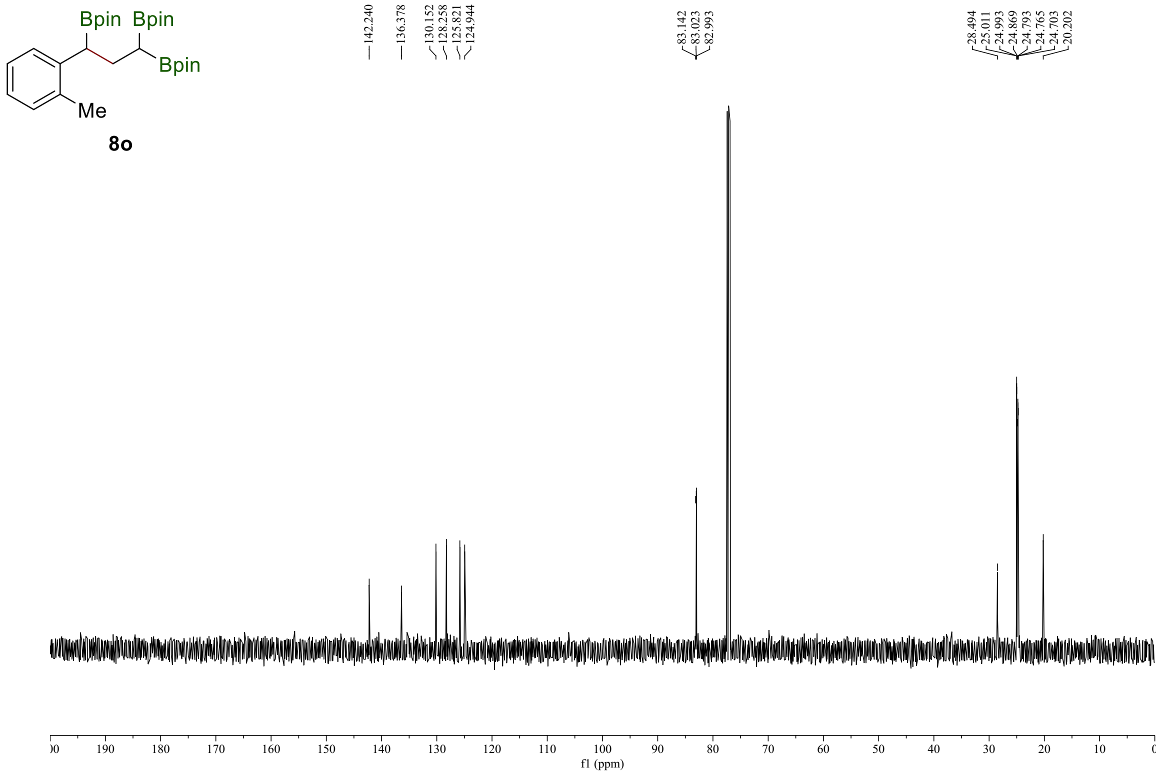

Supplementary Figure 93. ^{13}C{^{1}H} NMR spectrum of compound **8o**.

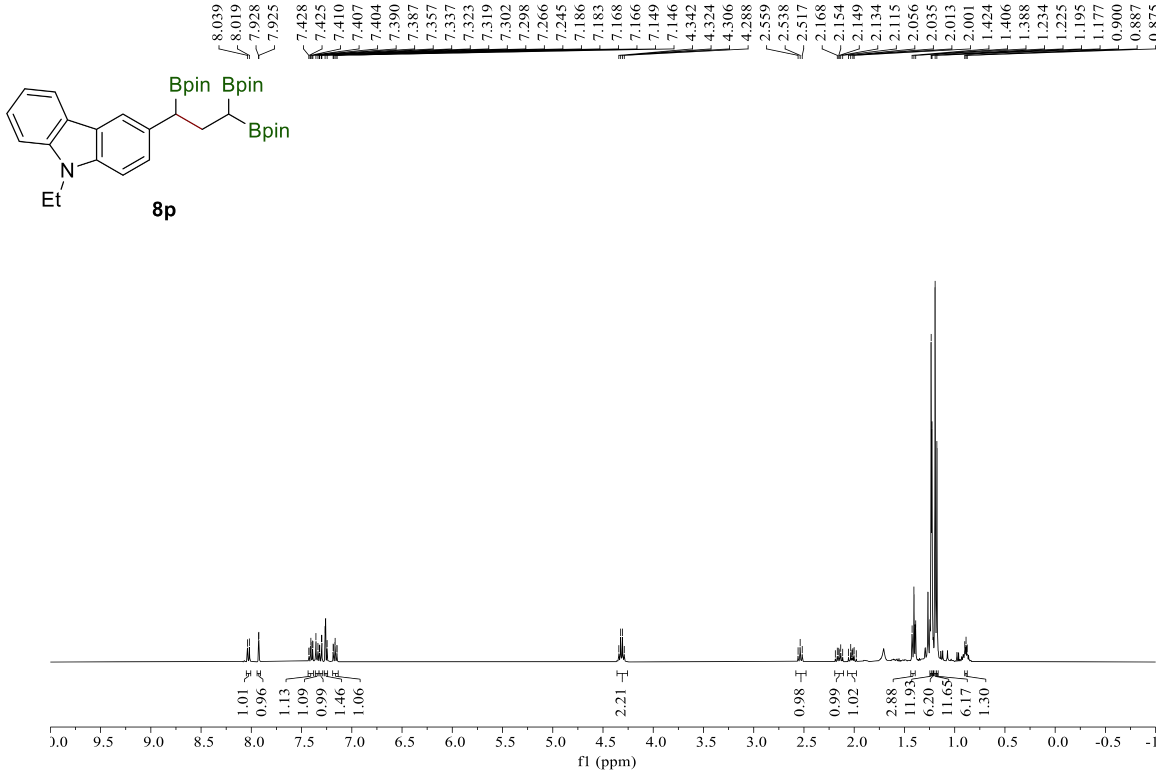

Supplementary Figure 94. ^{1}H NMR spectrum of compound **8p**.

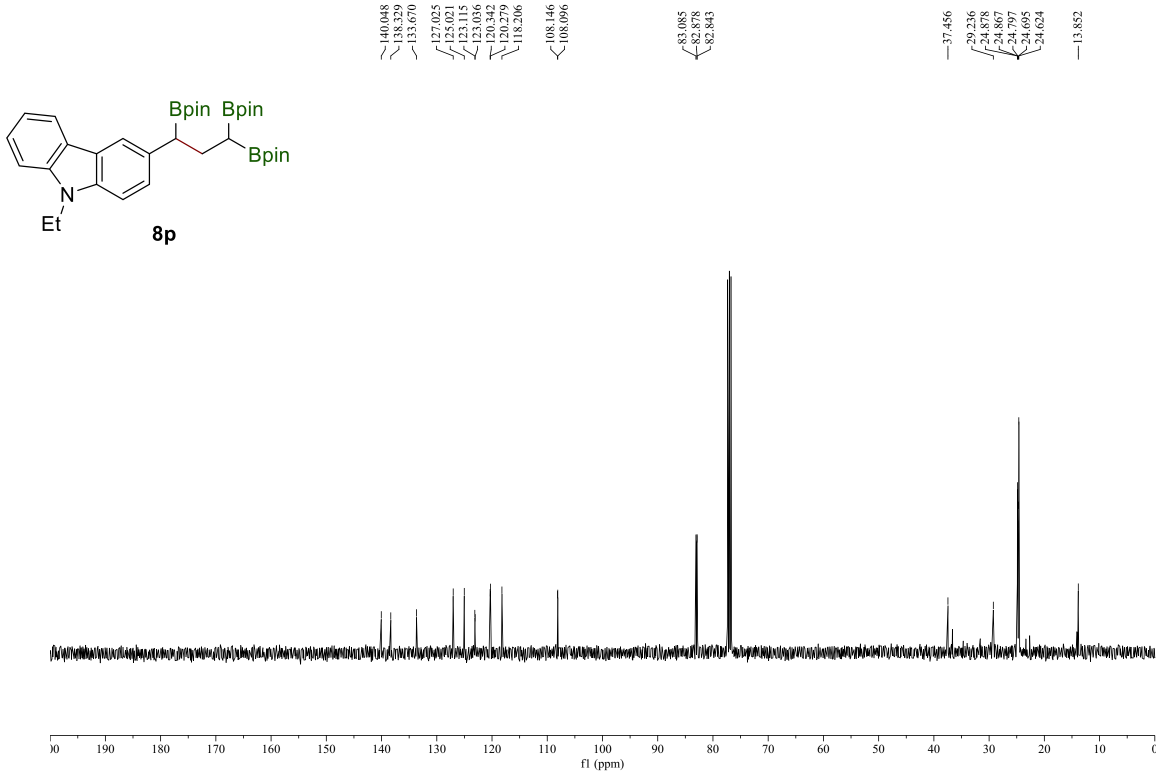

Supplementary Figure 95. $^{13}C\{^{1}H\}$ NMR spectrum of compound **8p**.

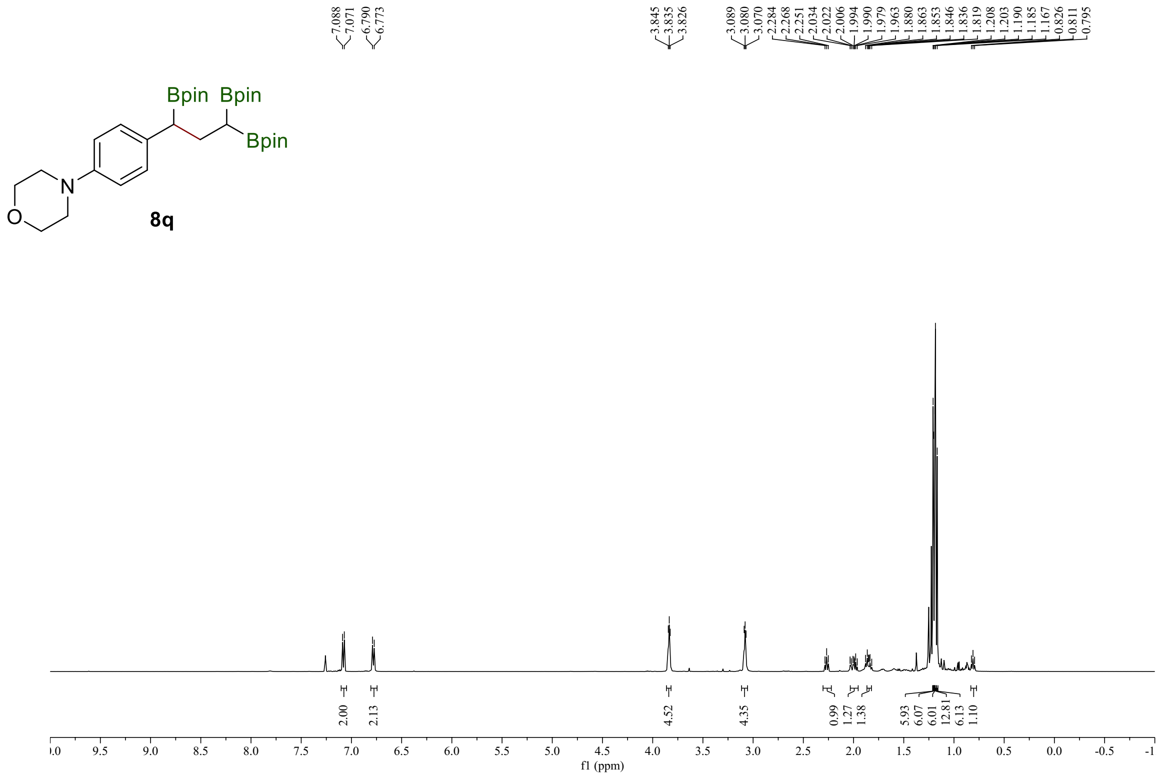

Supplementary Figure 96. ^{1}H NMR spectrum of compound **8q**.

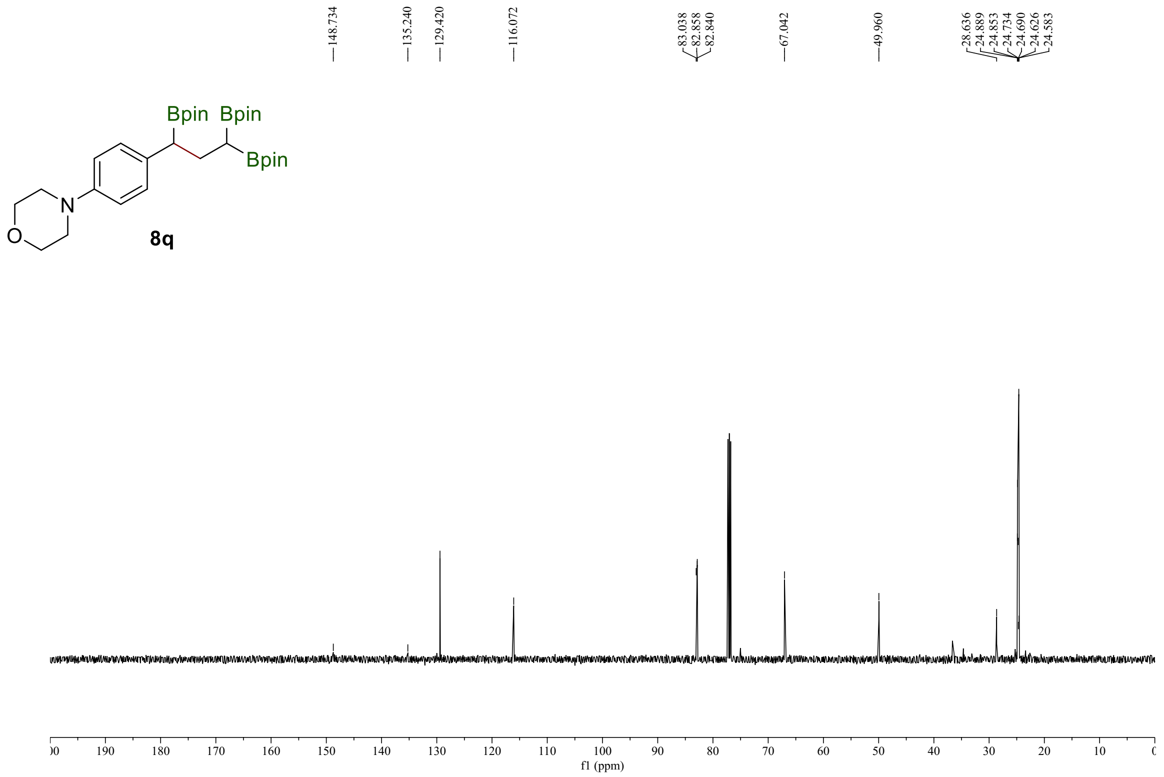

Supplementary Figure 97. ^{13}C{^{1}H} NMR spectrum of compound **8q**.

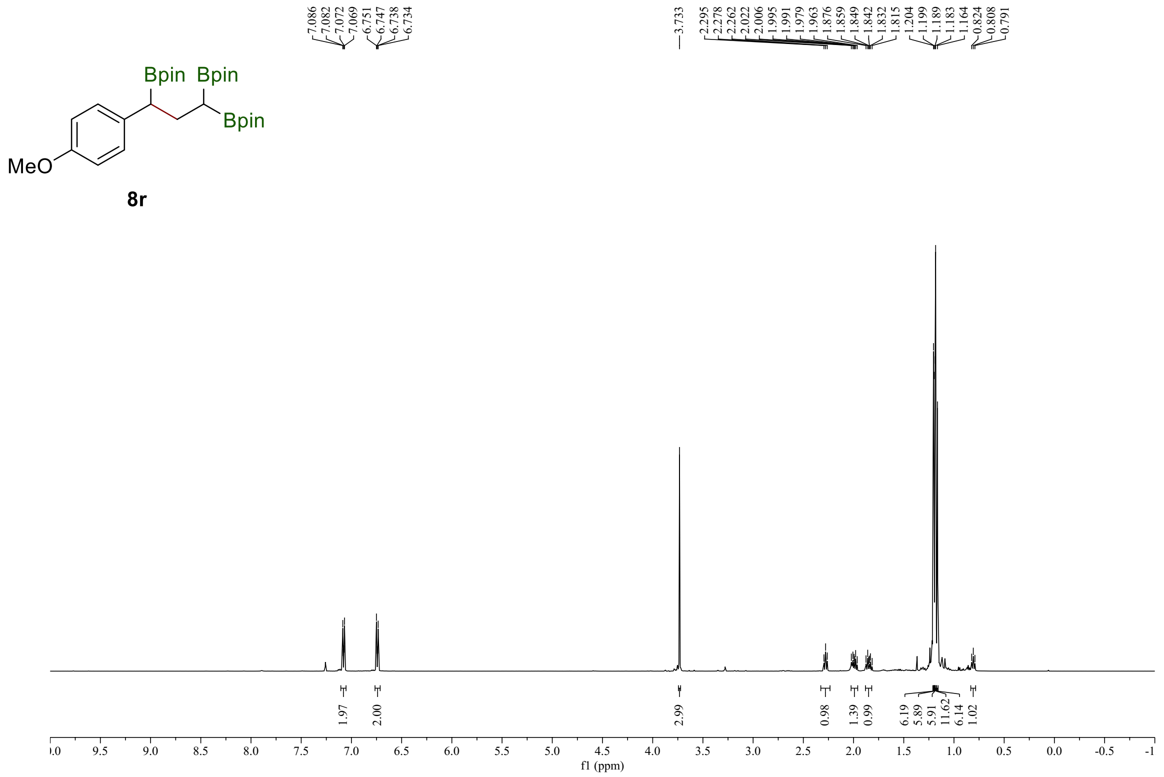

Supplementary Figure 98. ^{1}H NMR spectrum of compound **8r**.

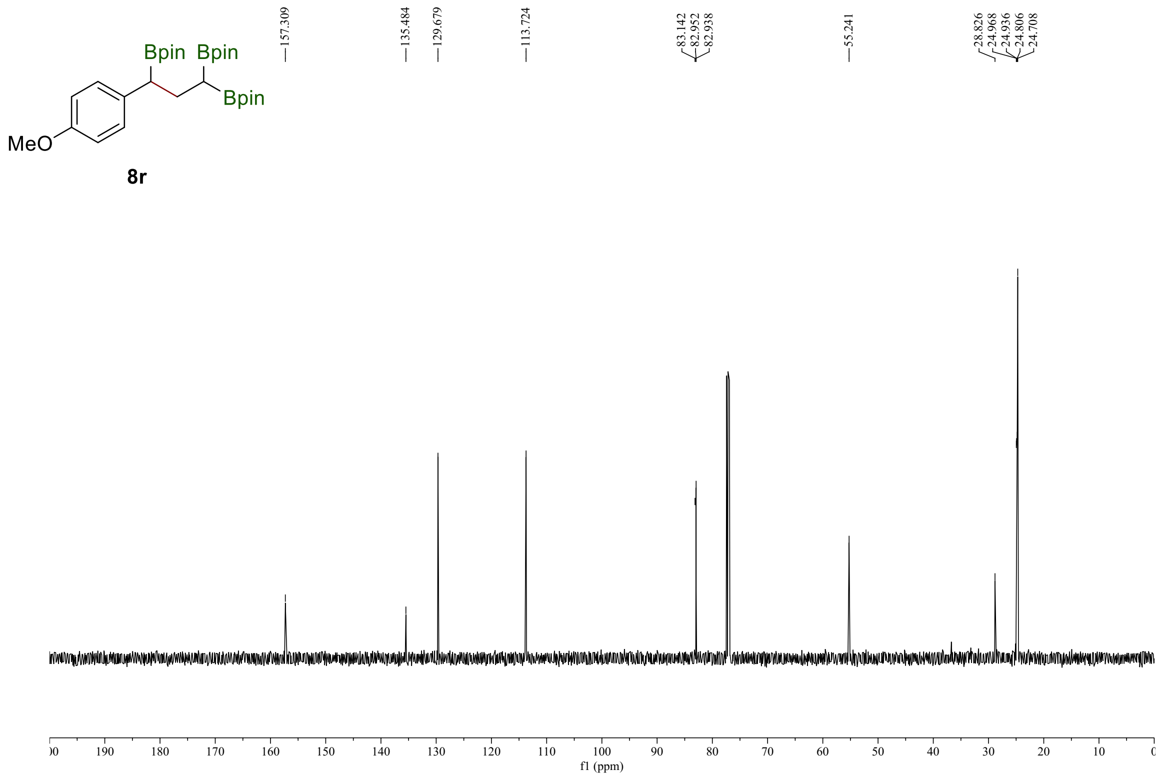

Supplementary Figure 99. $^{13}C\{^1H\}$ NMR spectrum of compound **8r**.

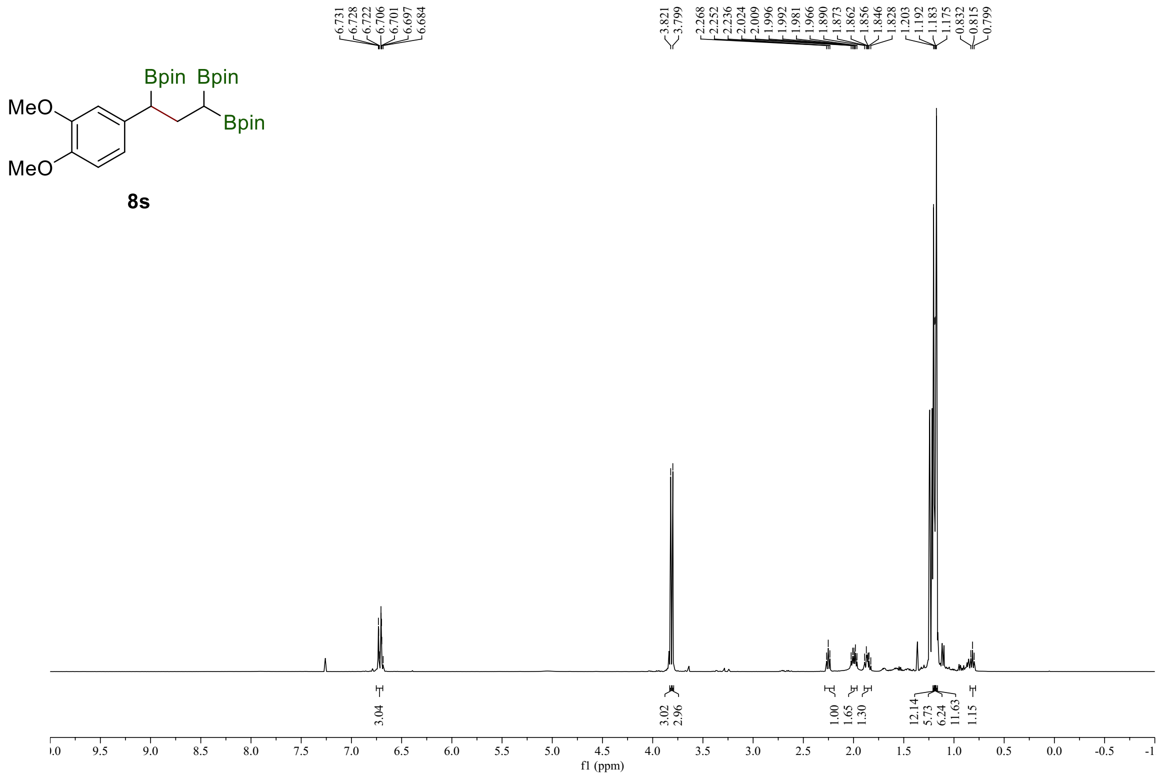

Supplementary Figure 100. ^{1}H NMR spectrum of compound **8s**.

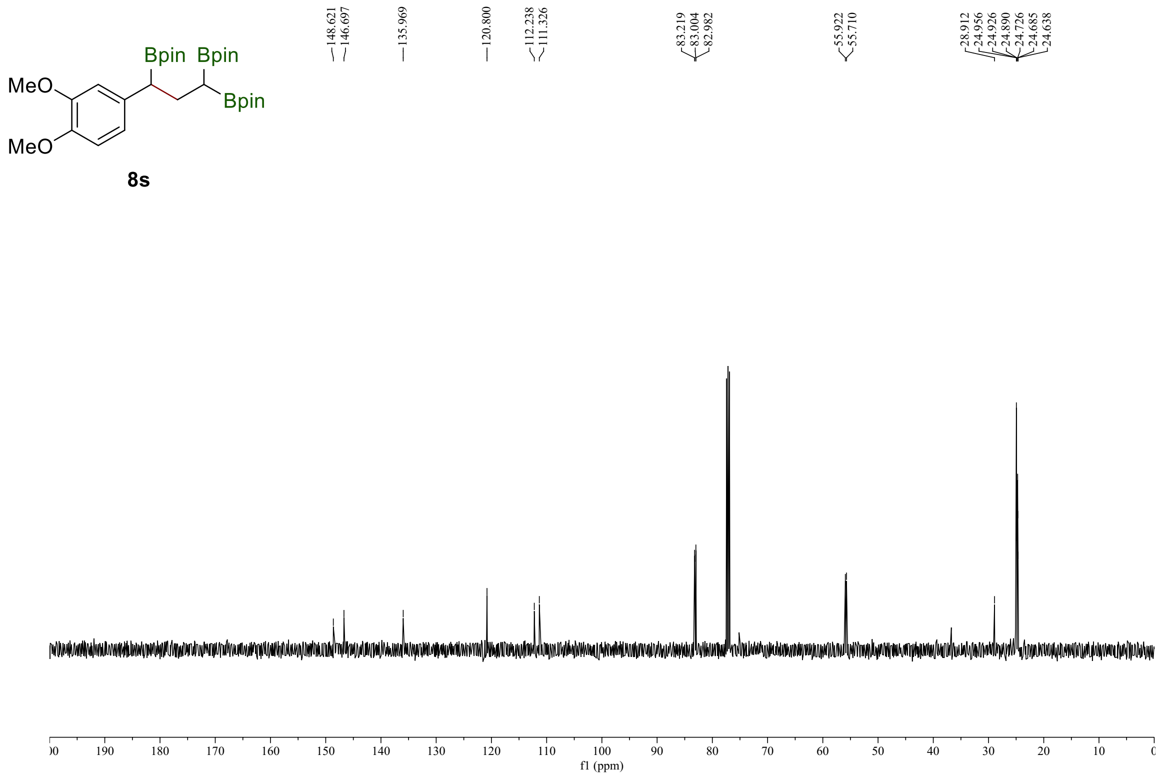

Supplementary Figure 101. $^{13}C\{^{1}H\}$ NMR spectrum of compound **8s**.

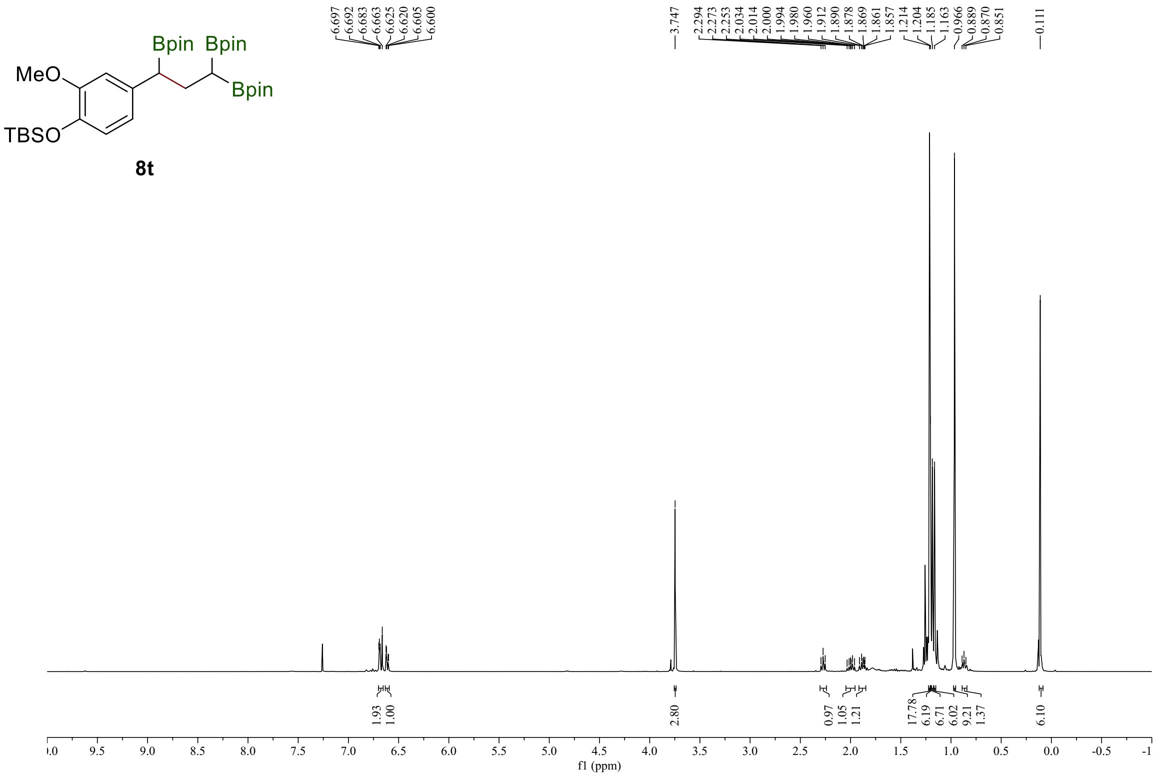

Supplementary Figure 102. ^{1}H NMR spectrum of compound **8t**.

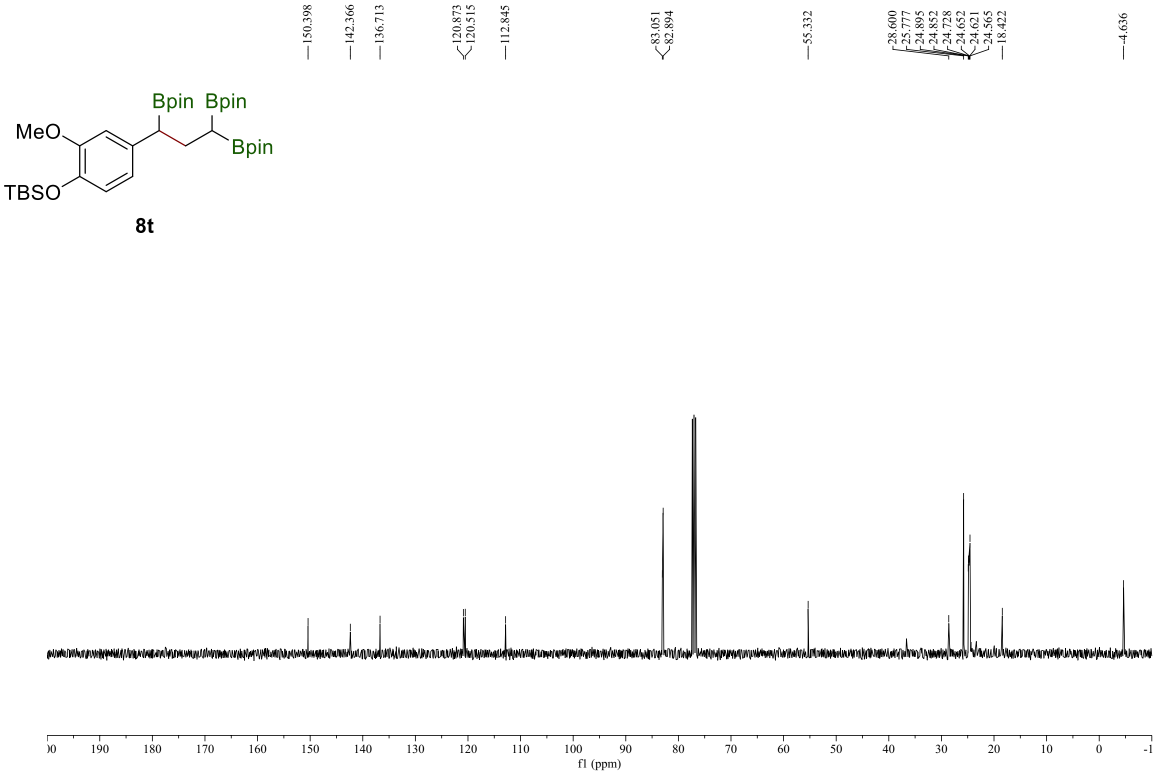

Supplementary Figure 103. $^{13}C\{^{1}H\}$ NMR spectrum of compound **8t**.

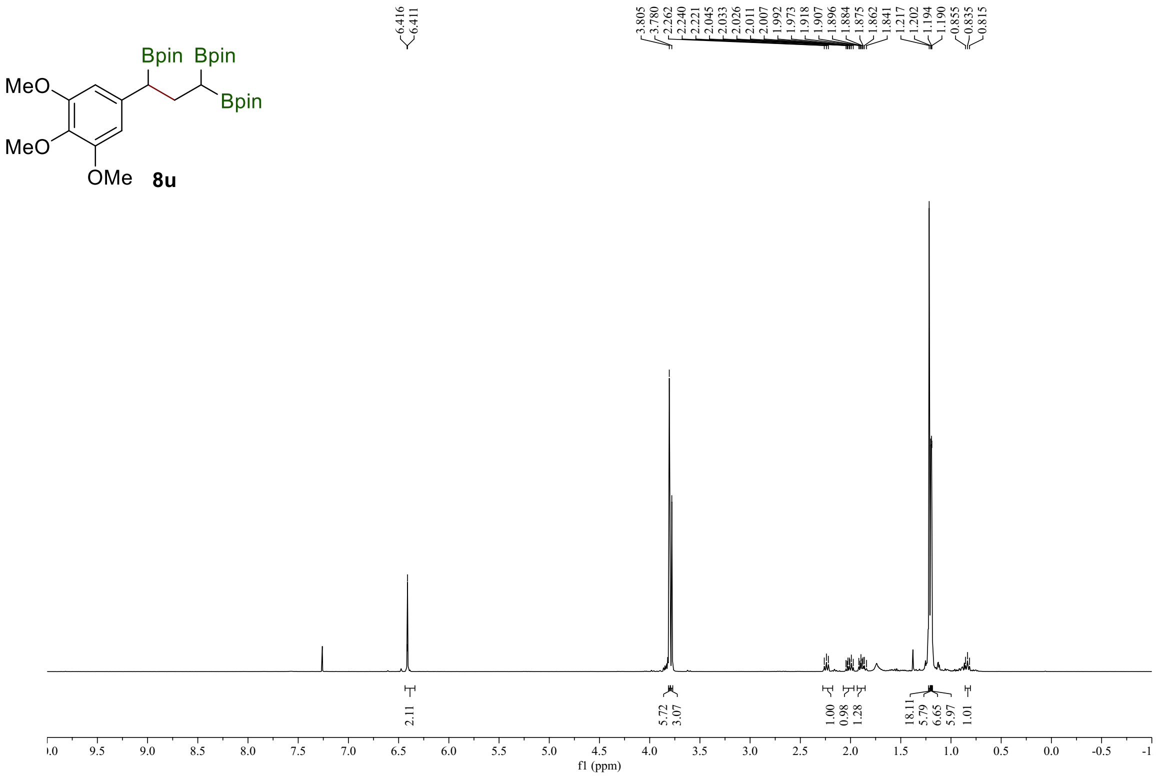

Supplementary Figure 104. ^{1}H NMR spectrum of compound **8u**.

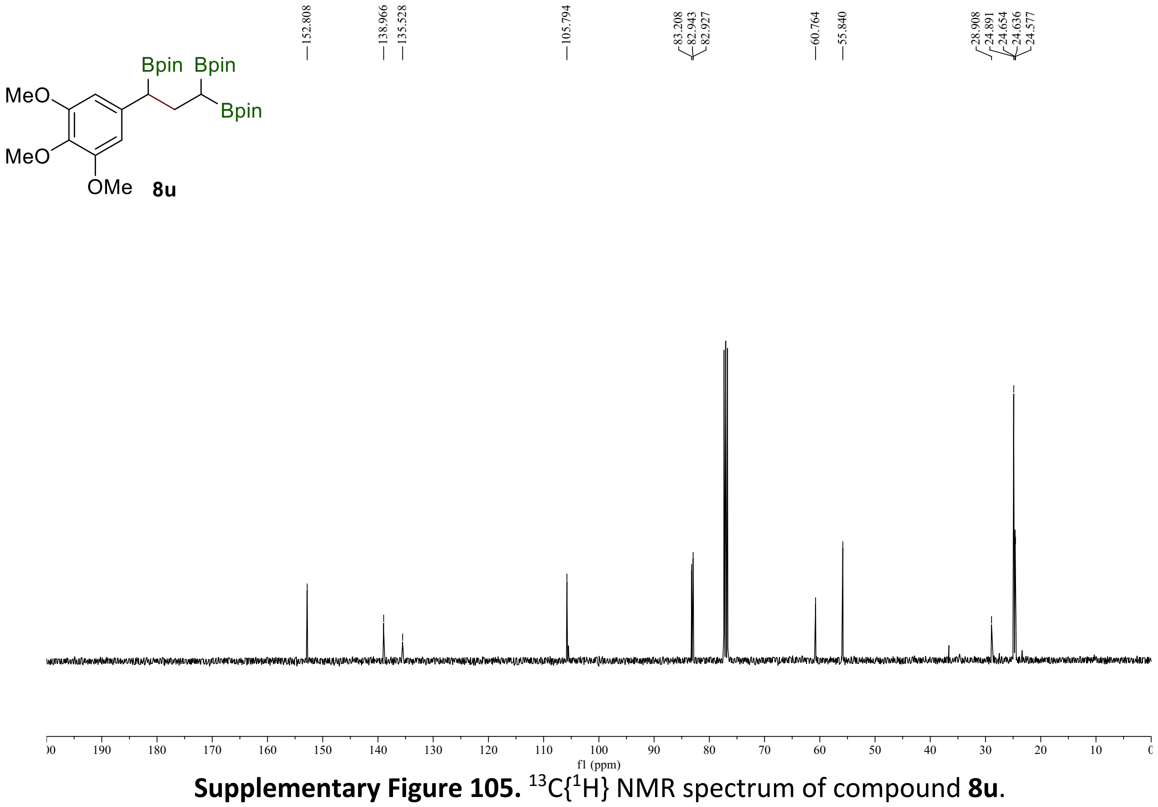

Supplementary Figure 105. $^{13}C\{^{1}H\}$ NMR spectrum of compound **8u**.

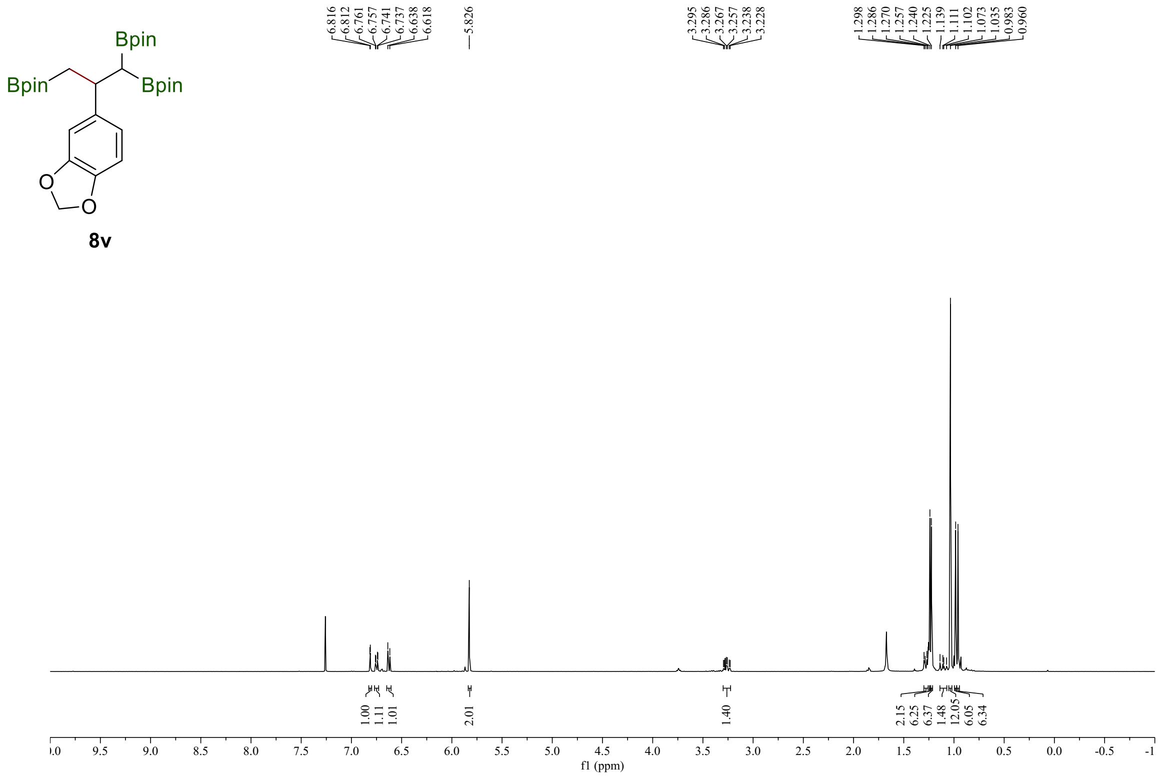

Supplementary Figure 106. ^{1}H NMR spectrum of compound **8v**.

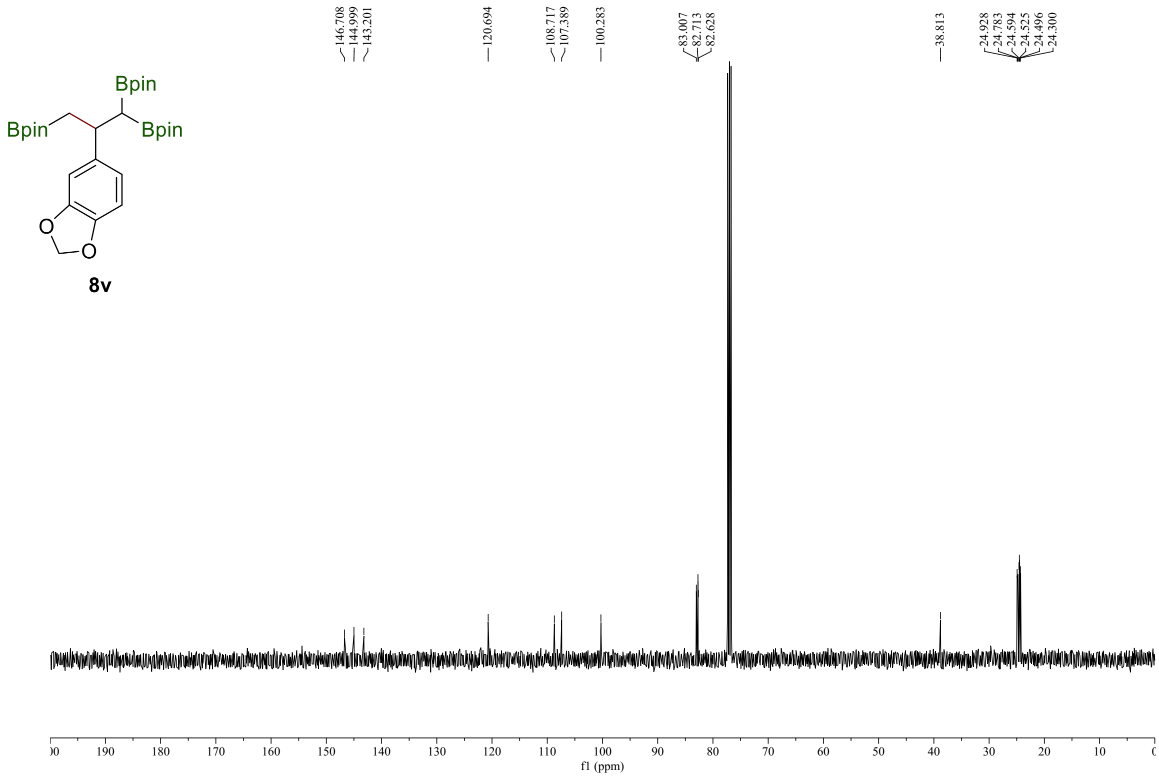

Supplementary Figure 107. ^{13}C{^{1}H} NMR spectrum of compound **8v**.

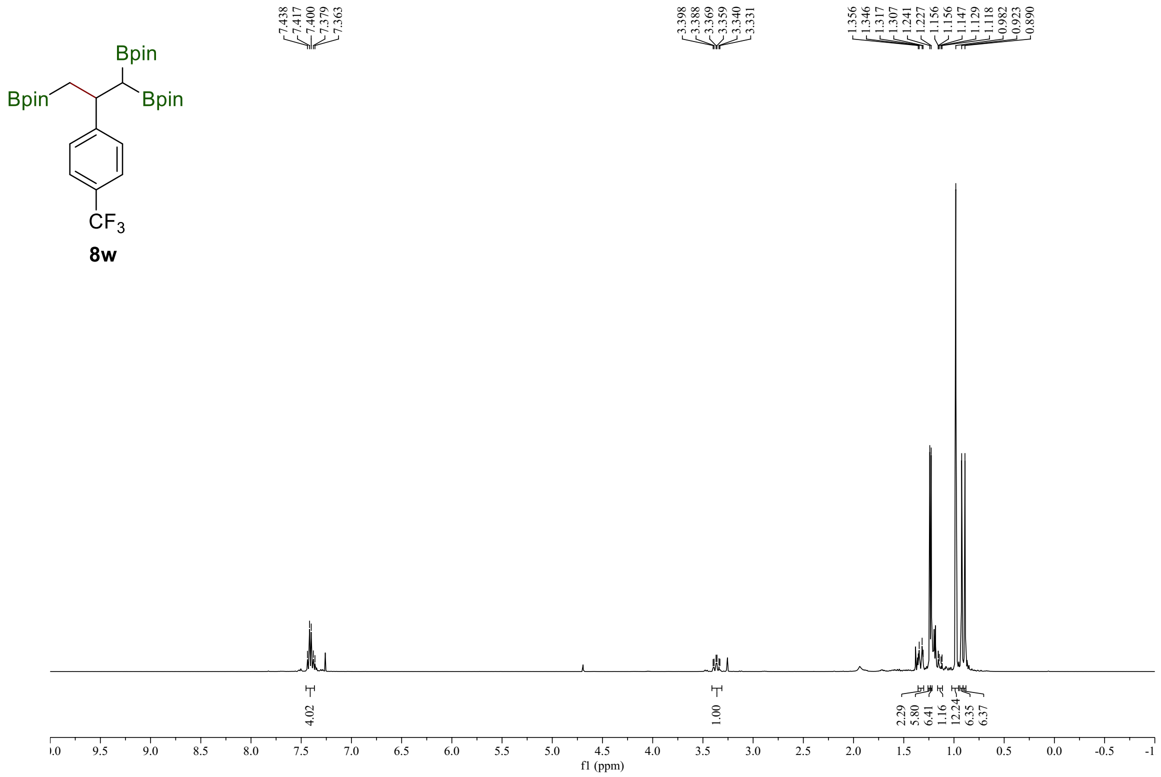

Supplementary Figure 108. ^{1}H NMR spectrum of compound **8w**.

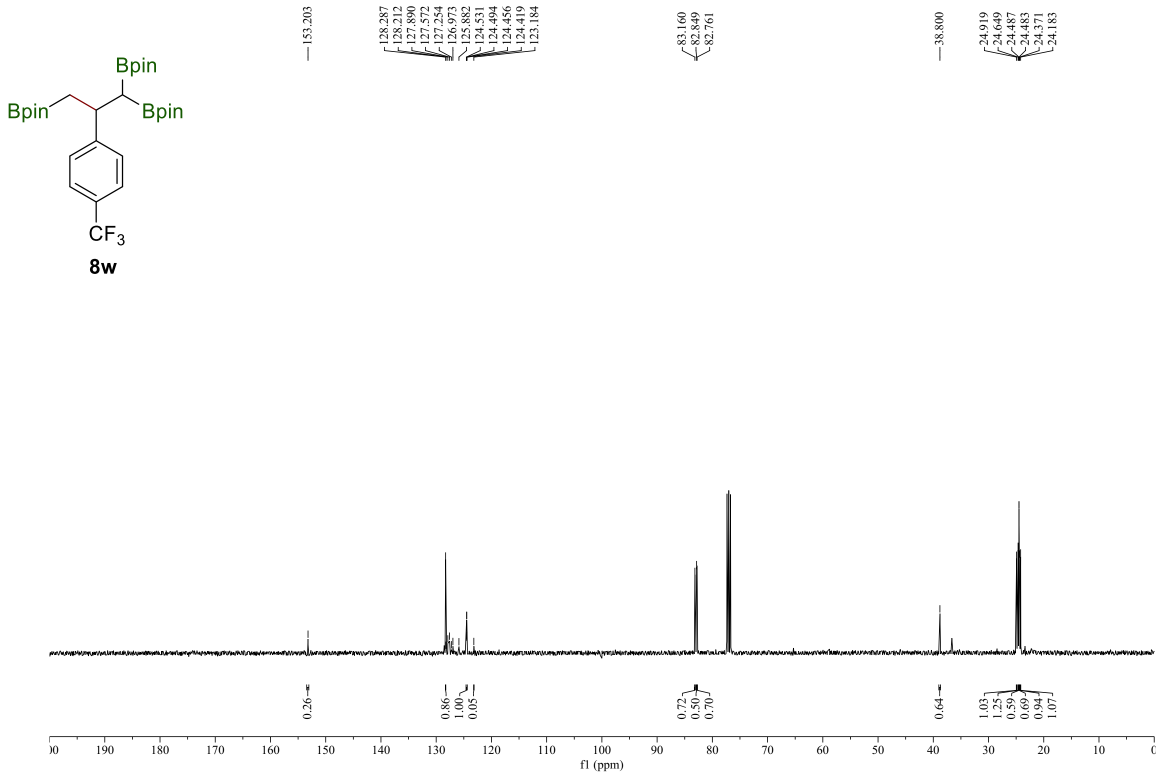

Supplementary Figure 109. ^{13}C{^{1}H} NMR spectrum of compound **8w**.

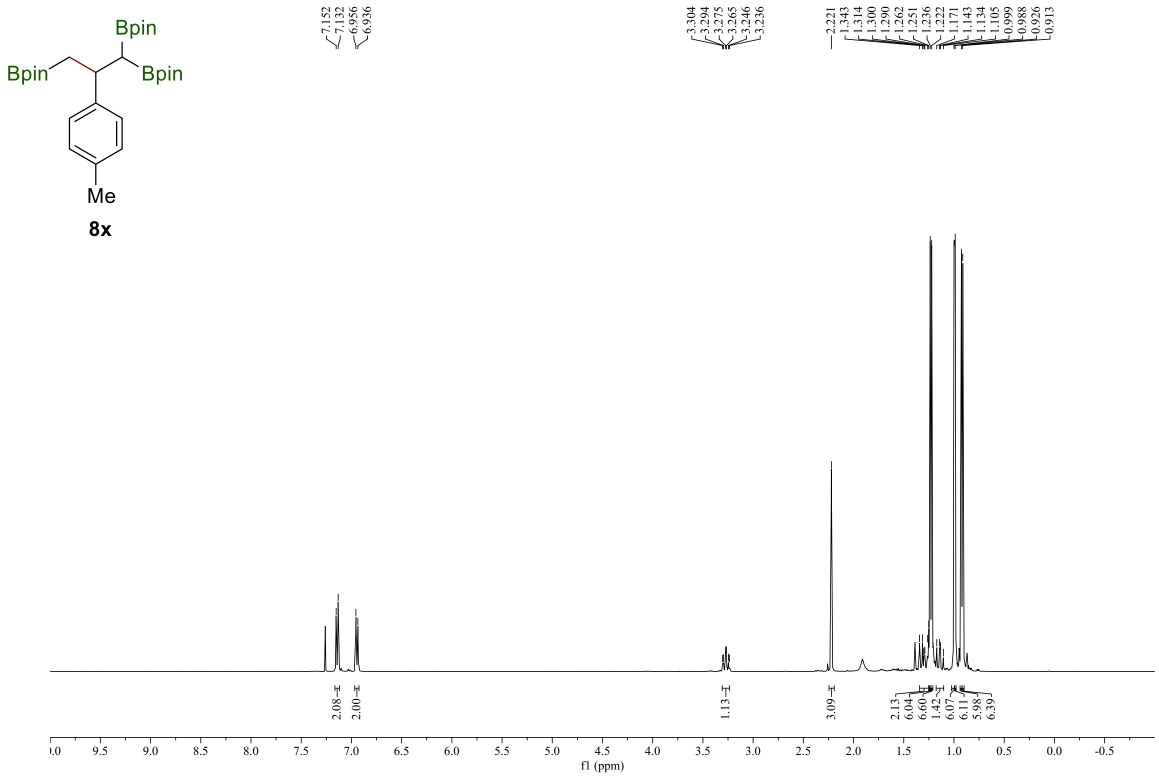

Supplementary Figure 110. ^{1}H NMR spectrum of compound **8x**.

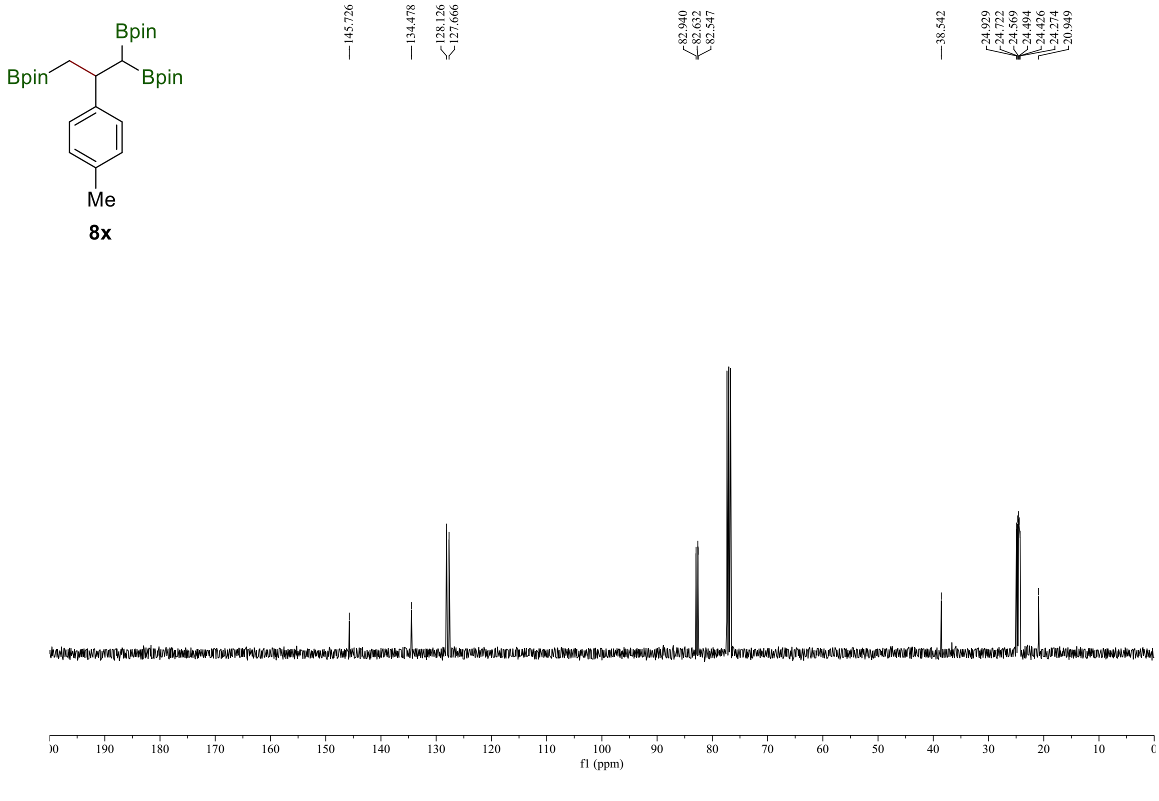

Supplementary Figure 111. $^{13}C\{^{1}H\}$ NMR spectrum of compound **8x**.

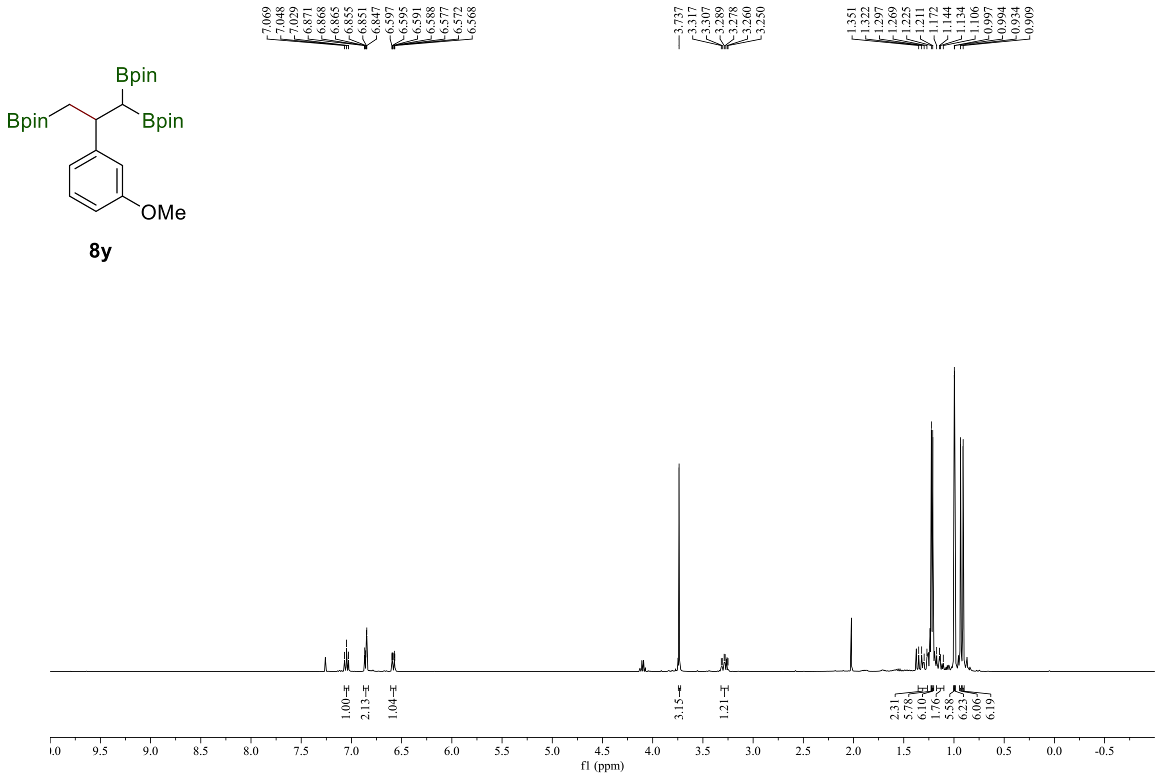

Supplementary Figure 112. ^{1}H NMR spectrum of compound **8y**.

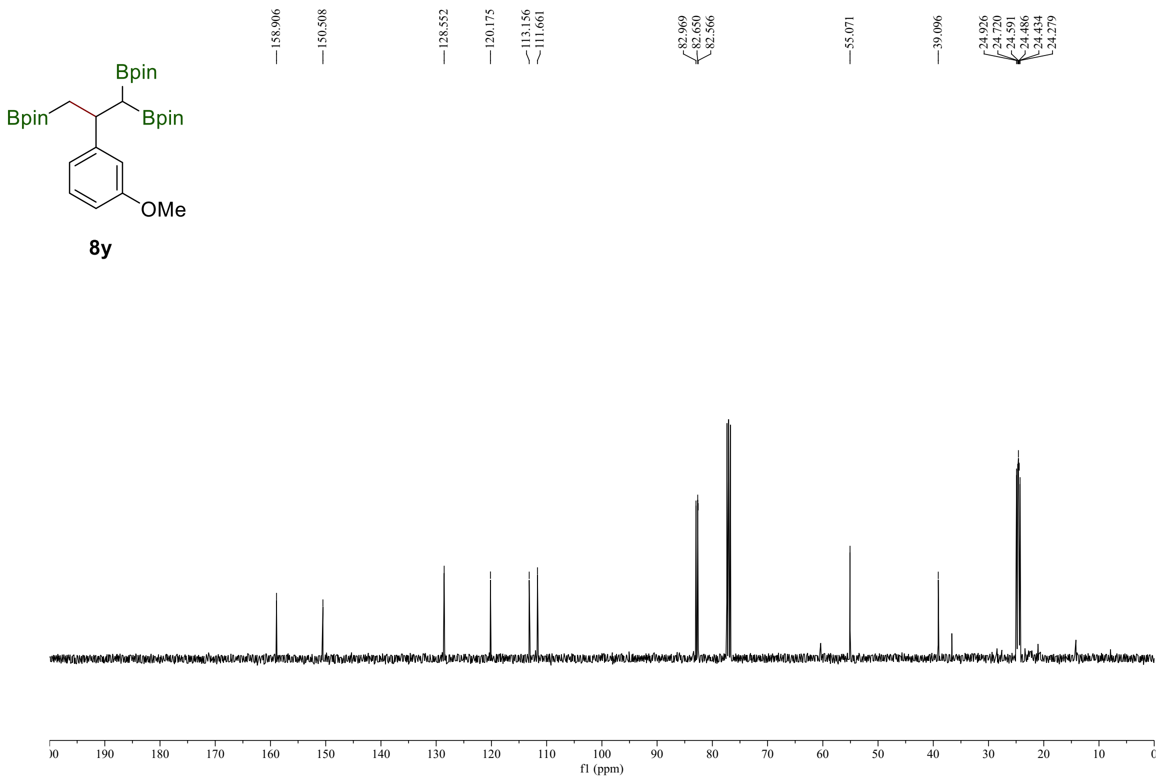

Supplementary Figure 113. $^{13}C\{^{1}H\}$ NMR spectrum of compound **8y**.

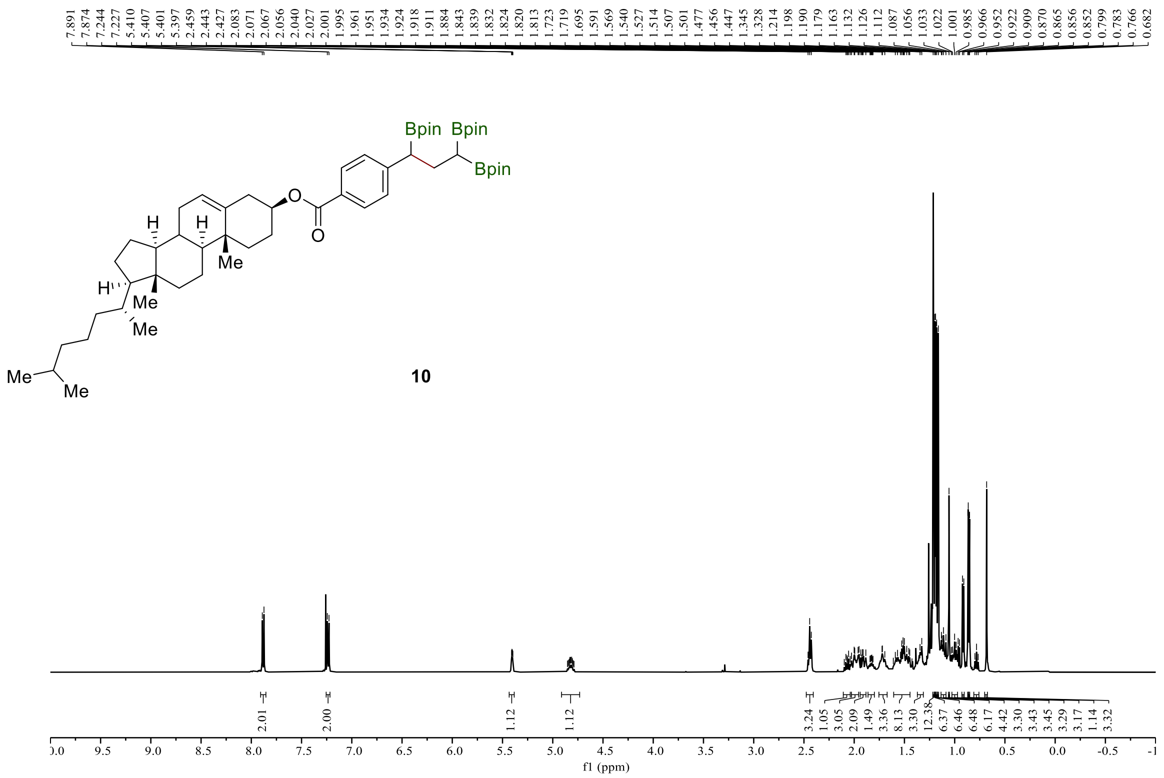

Supplementary Figure 114. ^{1}H NMR spectrum of compound **10**.

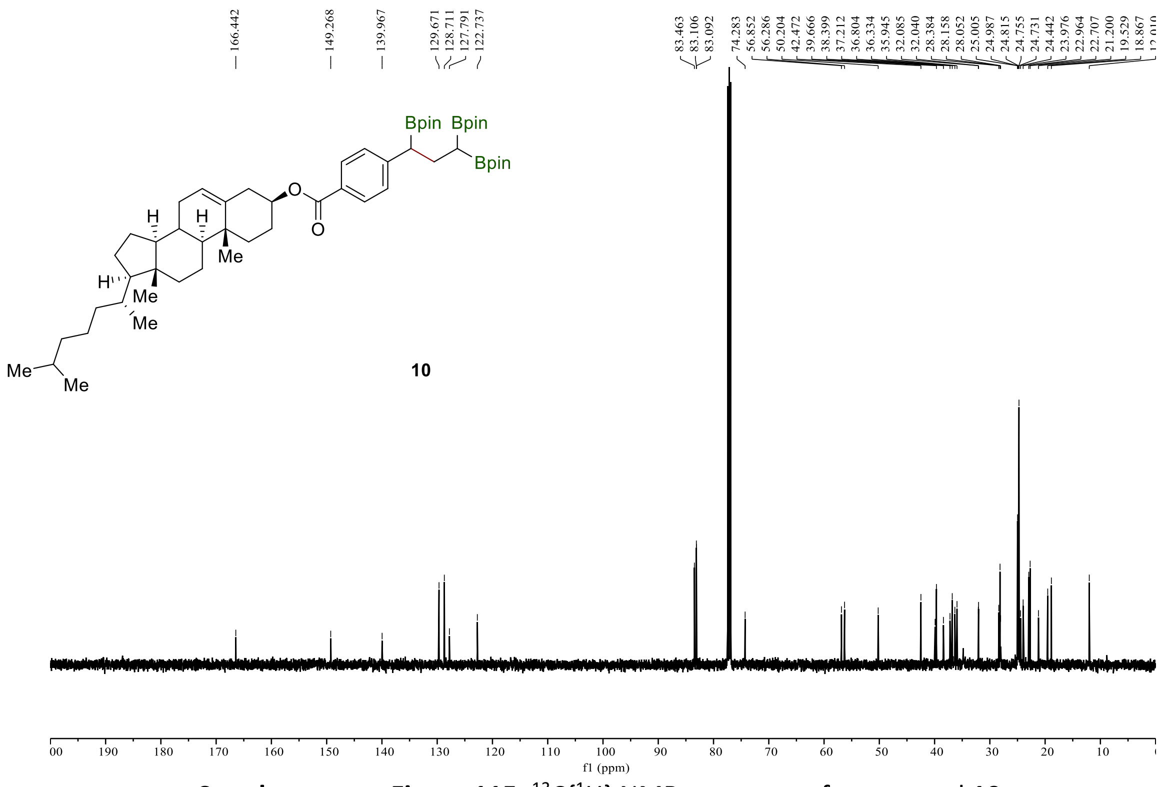

Supplementary Figure 115. ^{13}C{^{1}H} NMR spectrum of compound **10**.

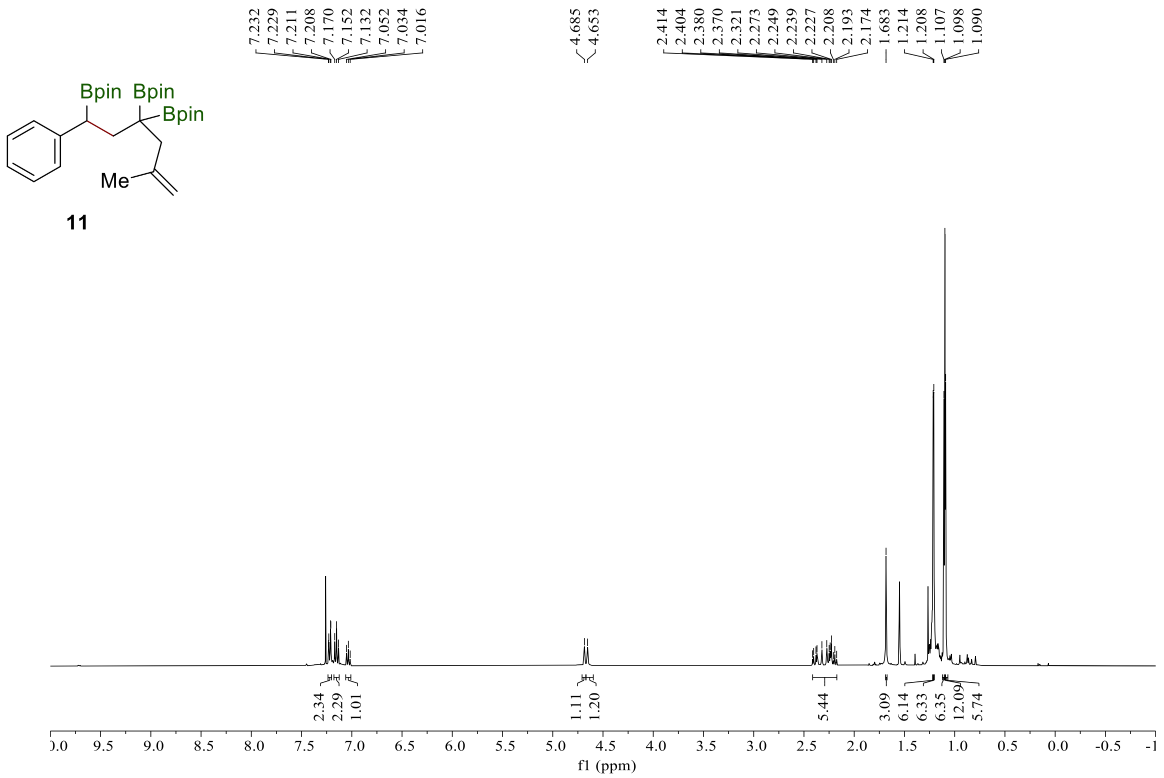

Supplementary Figure 116. ^{1}H NMR spectrum of compound **11**.

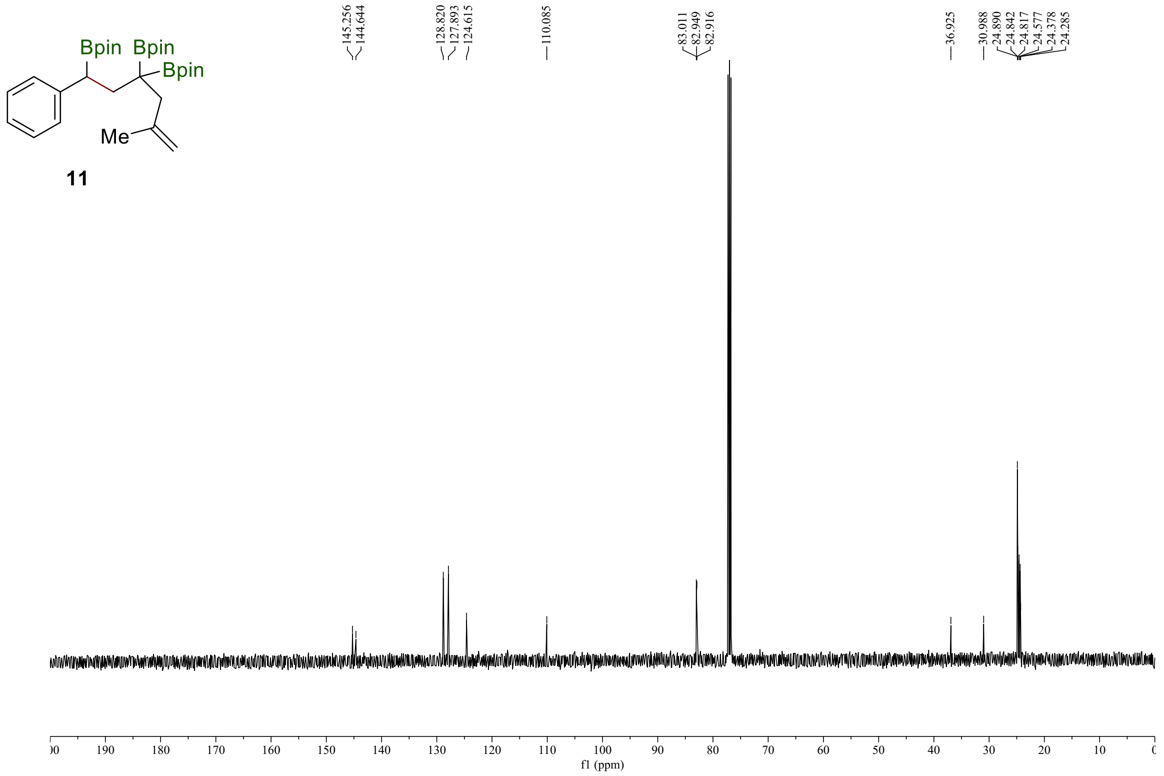

Supplementary Figure 117. ^{13}C{^{1}H} NMR spectrum of compound **11**.

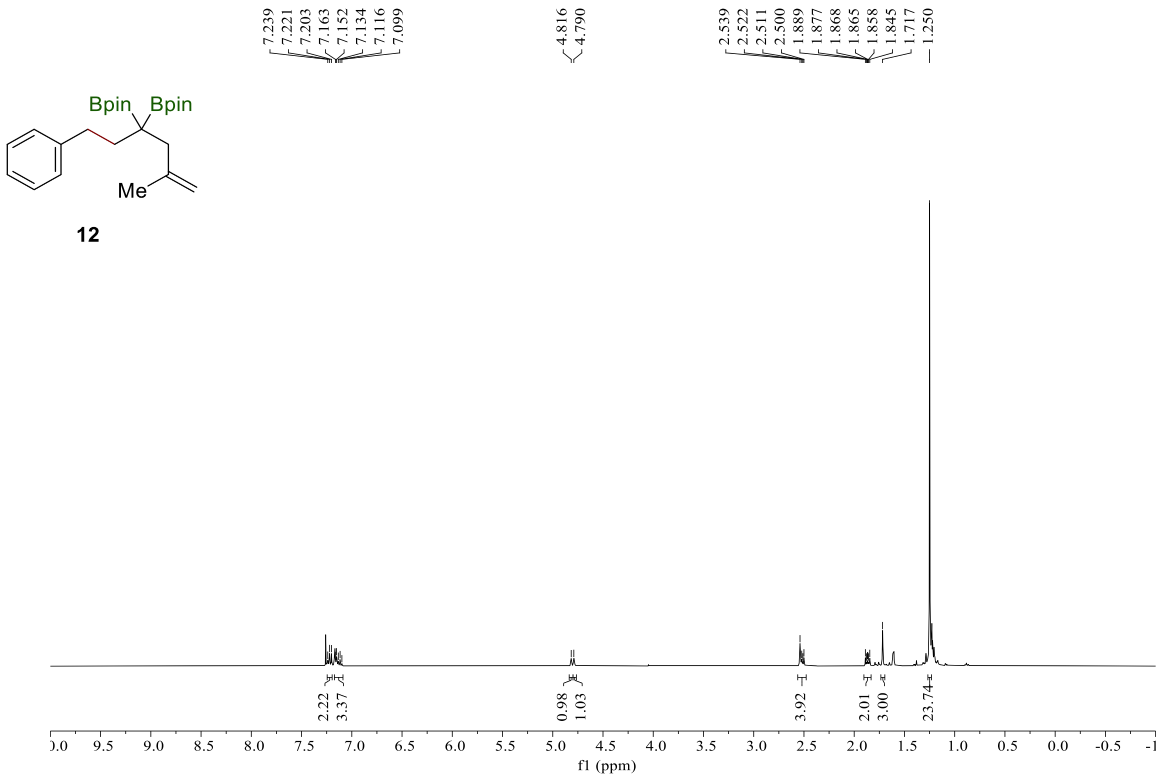

Supplementary Figure 118. ^{1}H NMR spectrum of compound **12**.

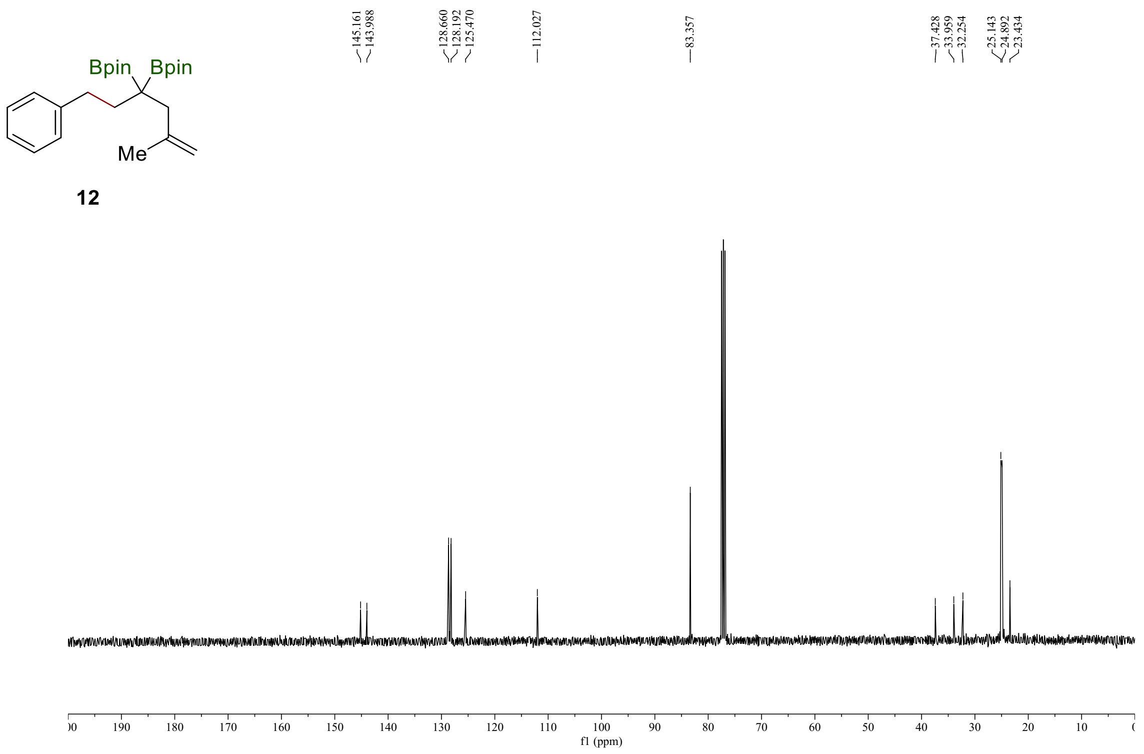

Supplementary Figure 119. $^{13}C\{^{1}H\}$ NMR spectrum of compound **12**.

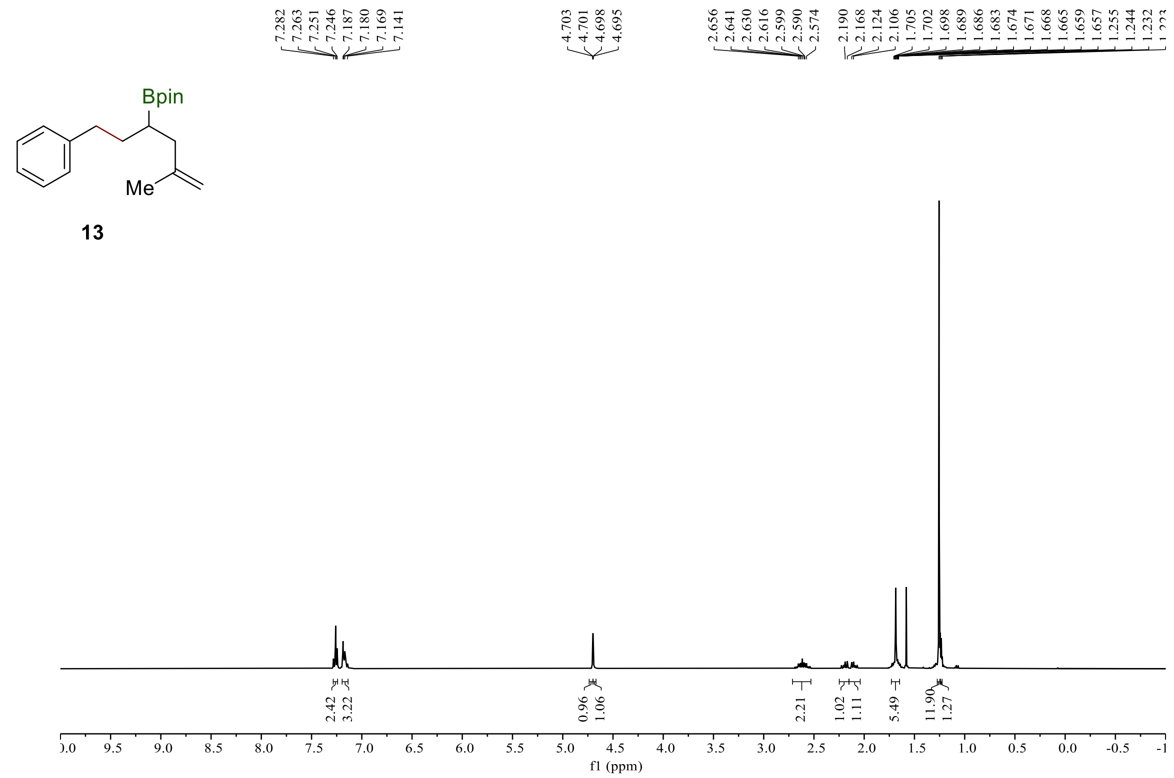

Supplementary Figure 120. ^{1}H NMR spectrum of compound **13**.

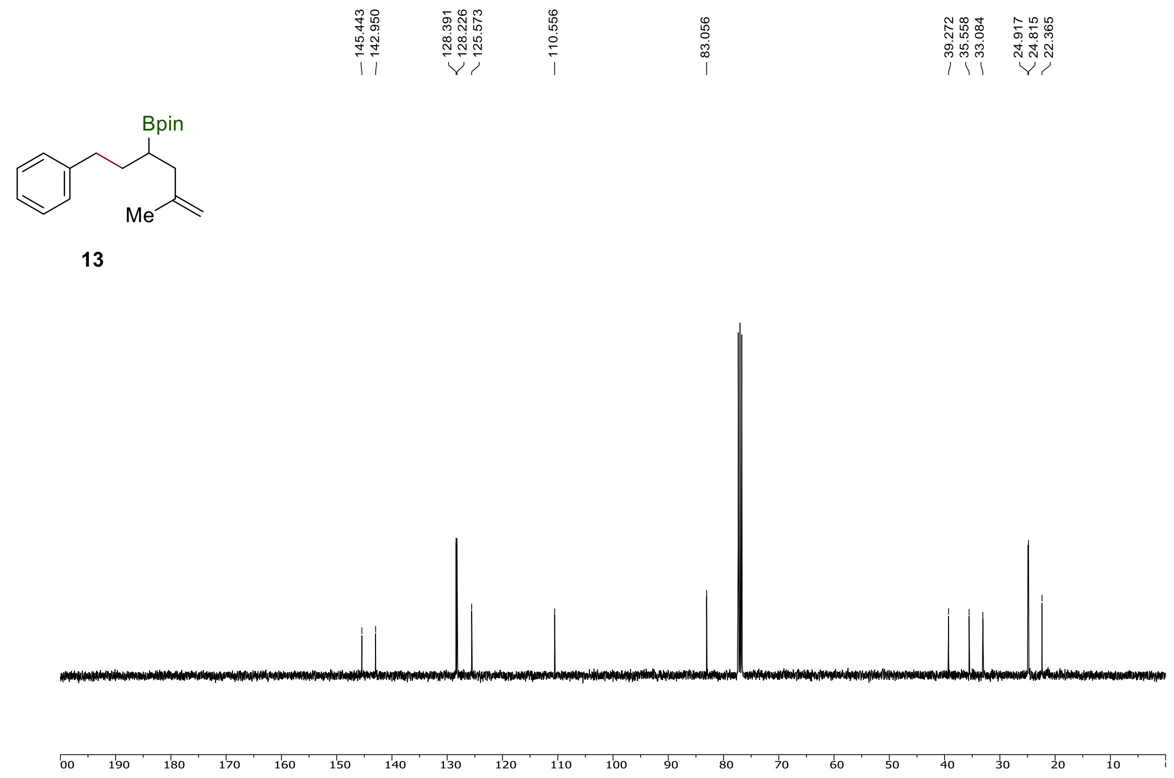

Supplementary Figure 121. $^{13}C\{^{1}H\}$ NMR spectrum of compound **13**.

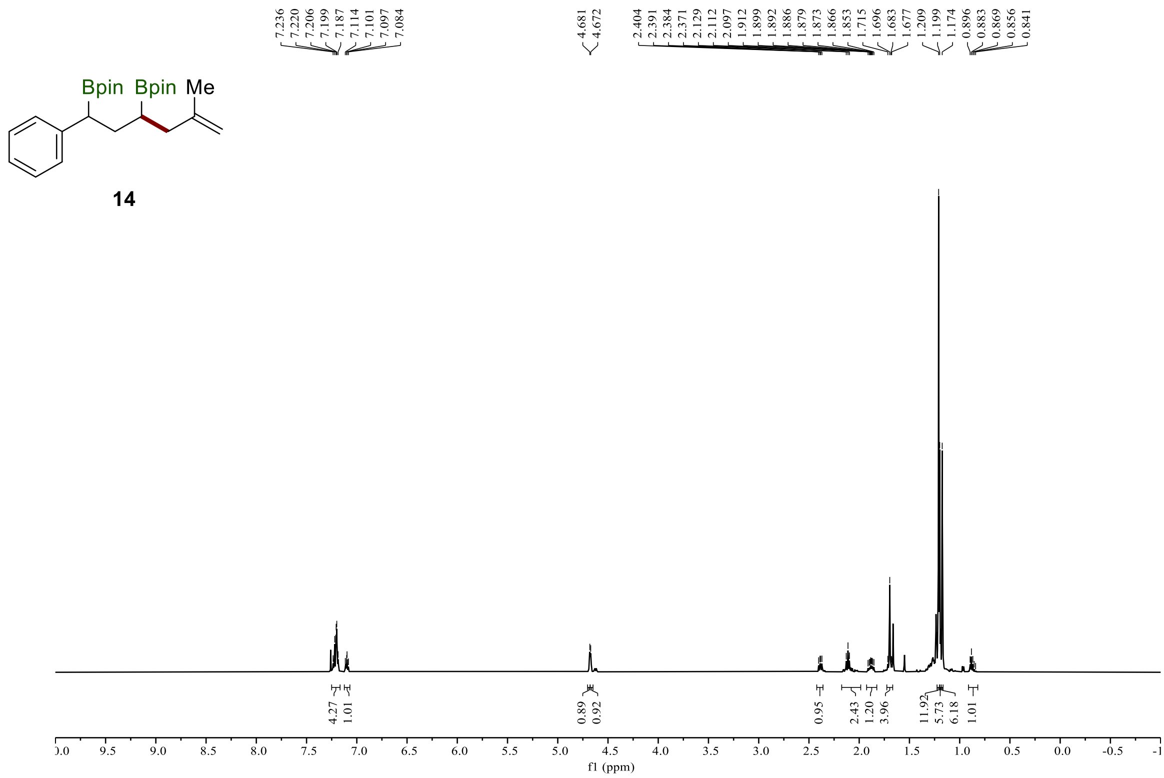

Supplementary Figure 122. ^{1}H NMR spectrum of compound **14**.

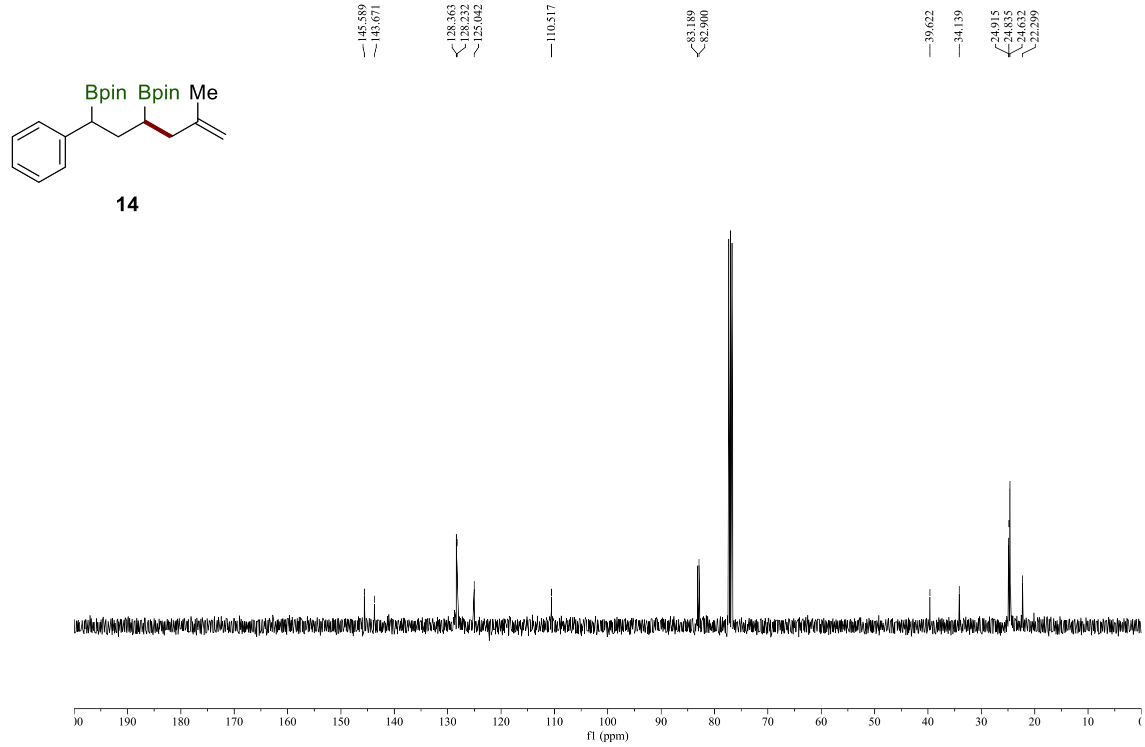

Supplementary Figure 123. ^{13}C{^{1}H} NMR spectrum of compound **14**.

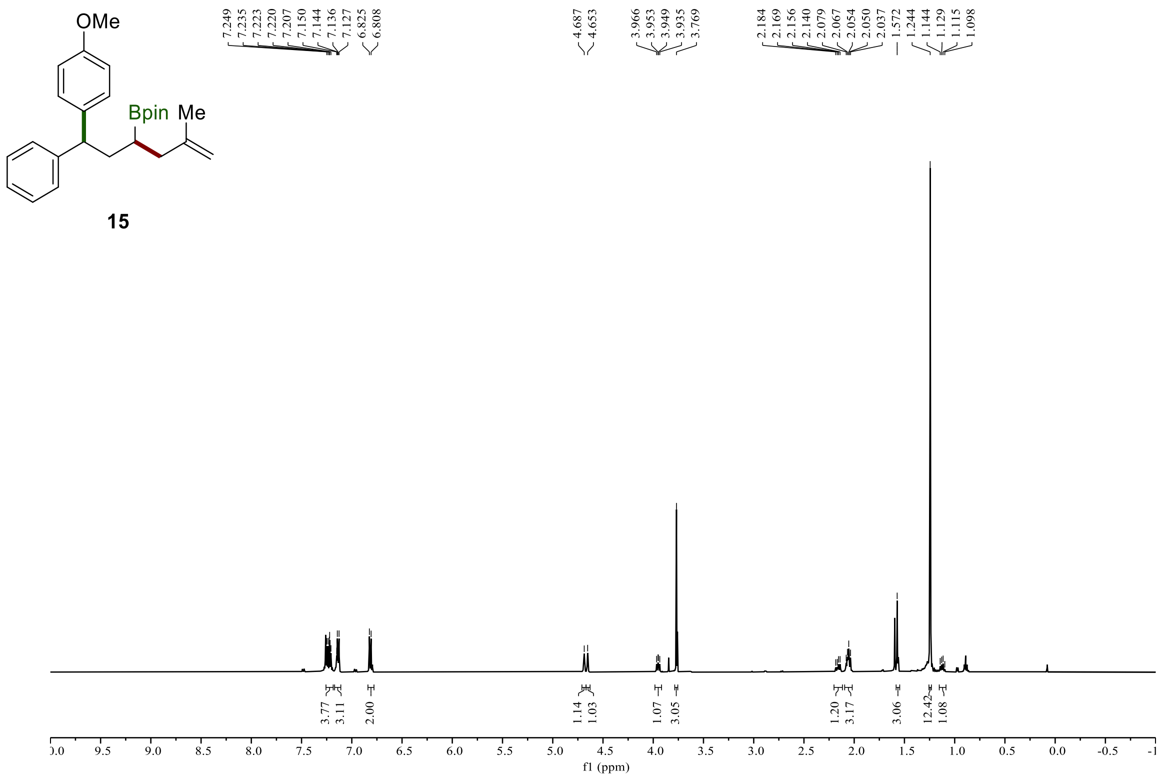

Supplementary Figure 124. ^{1}H NMR spectrum of compound **15**.

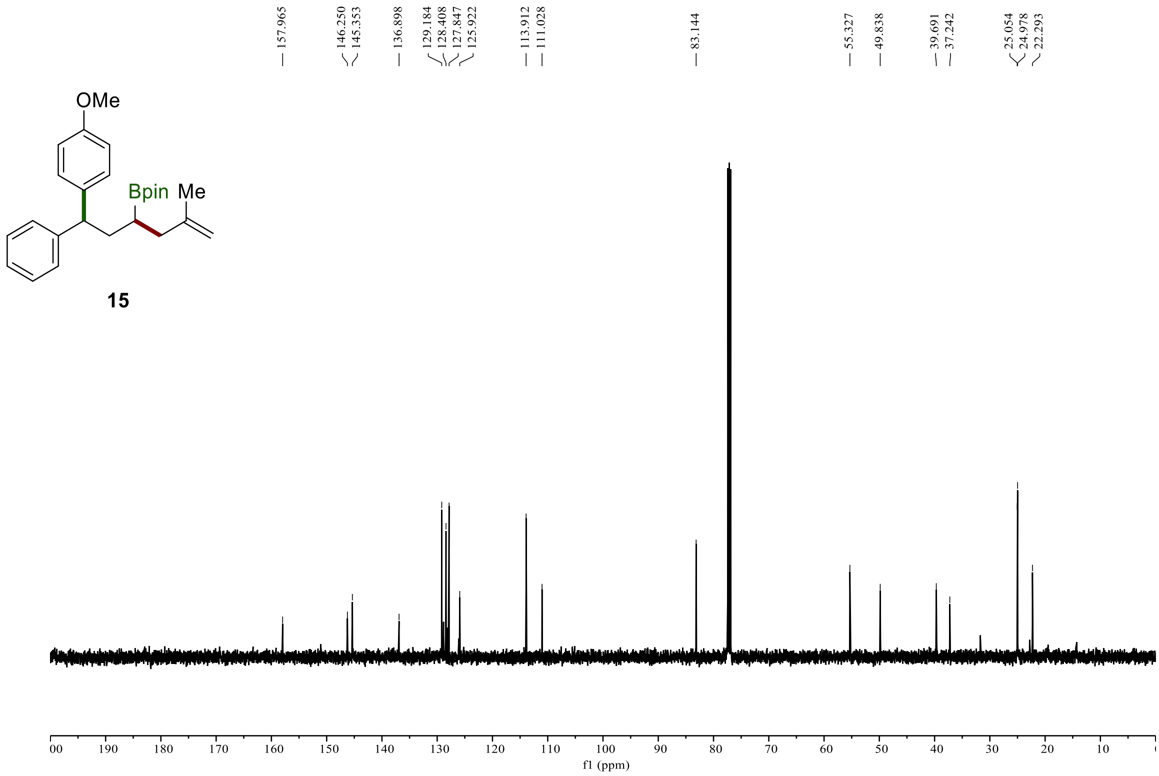

Supplementary Figure 125. $^{13}C\{^{1}H\}$ NMR spectrum of compound **15**.

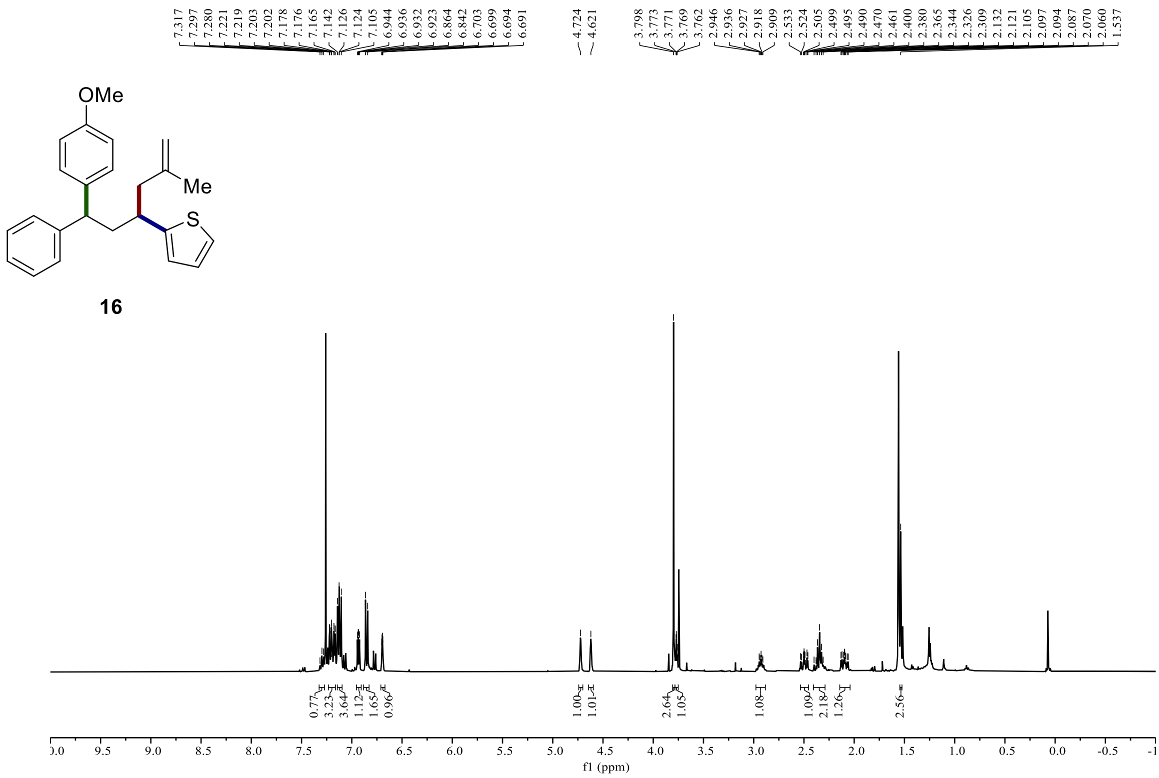

Supplementary Figure 126. ^{1}H NMR spectrum of compound **16**.

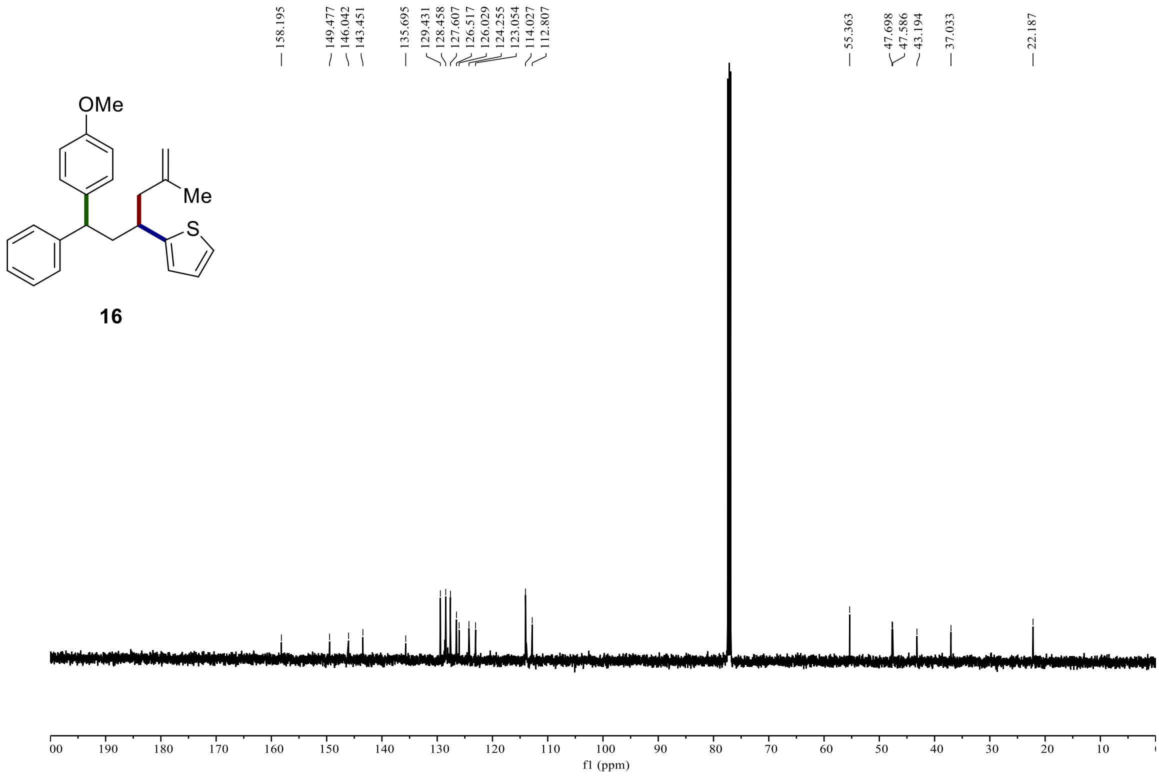

Supplementary Figure 127. ^{13}C{^{1}H} NMR spectrum of compound **16**.

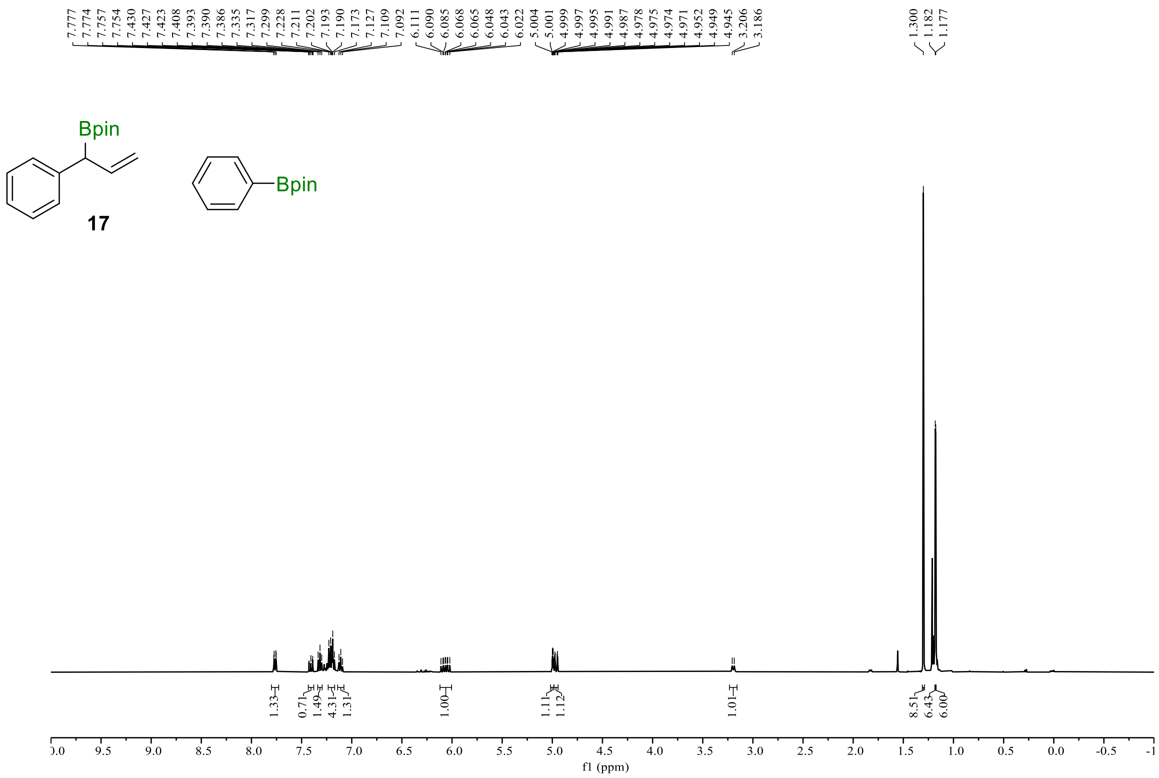

Supplementary Figure 128. ^{1}H NMR spectrum of compound **17**.

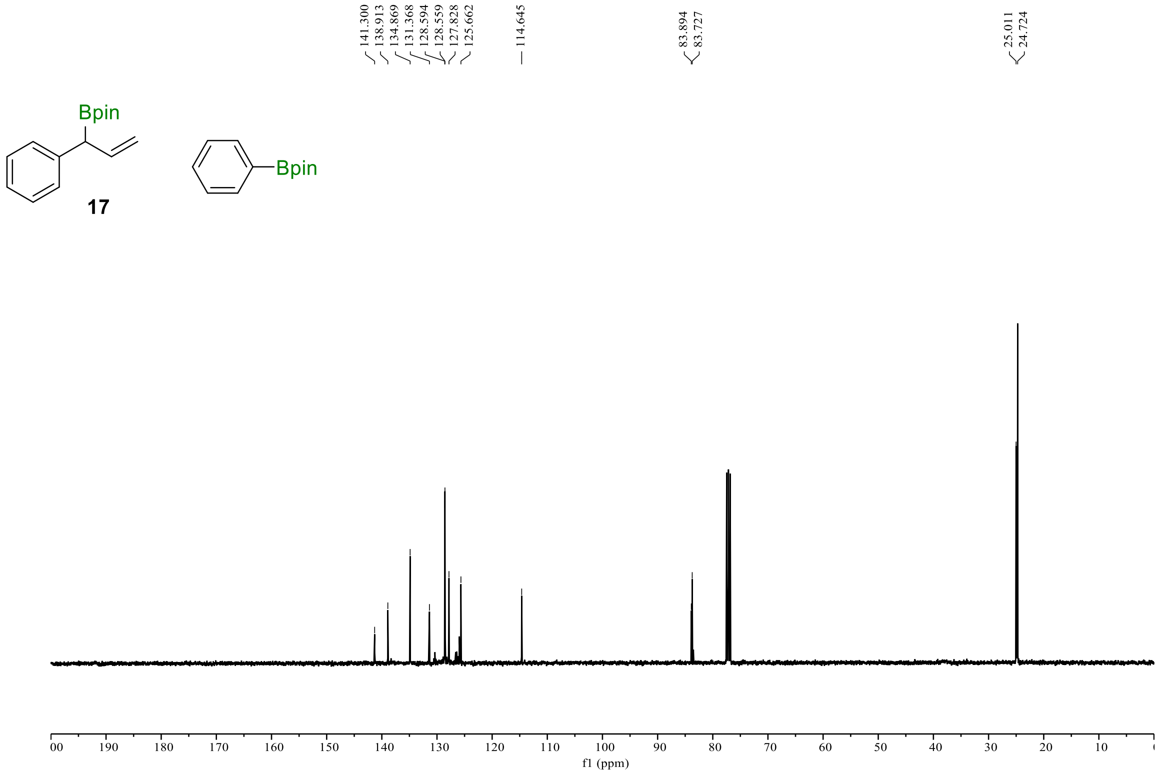

Supplementary Figure 129. $^{13}C\{^1H\}$ NMR spectrum of compound **17**.

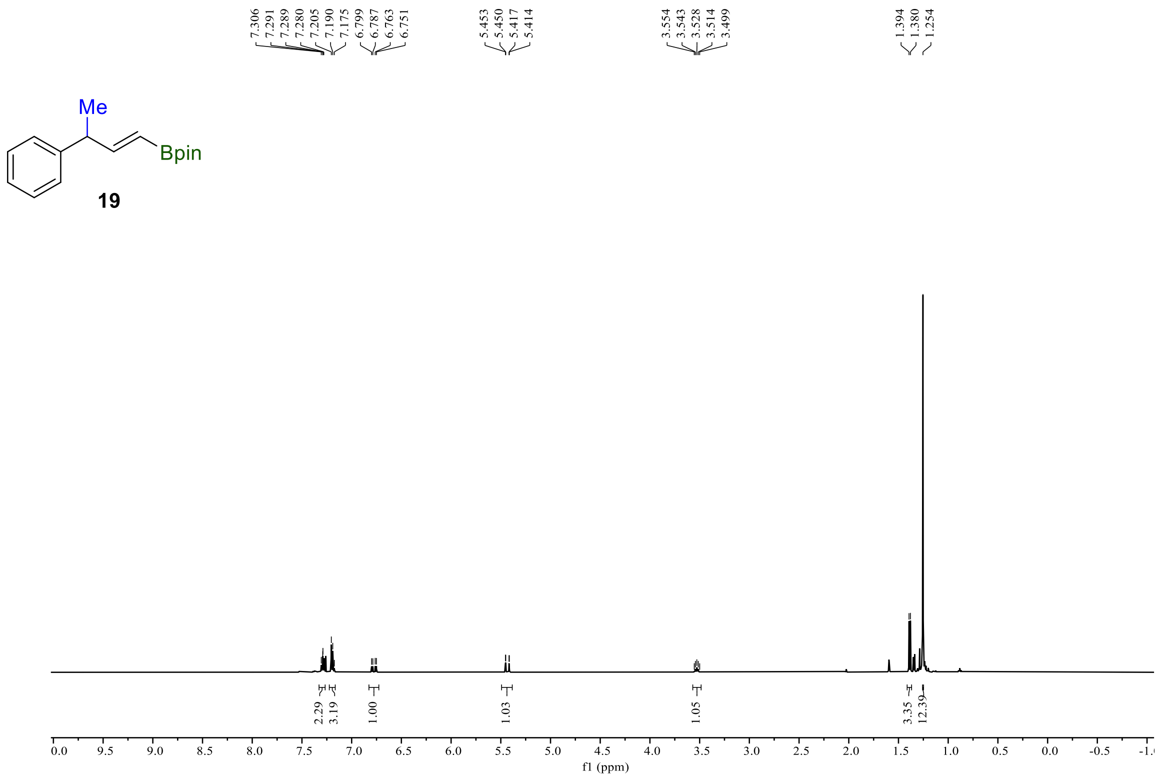

Supplementary Figure 130. ^{1}H NMR spectrum of compound **19**.

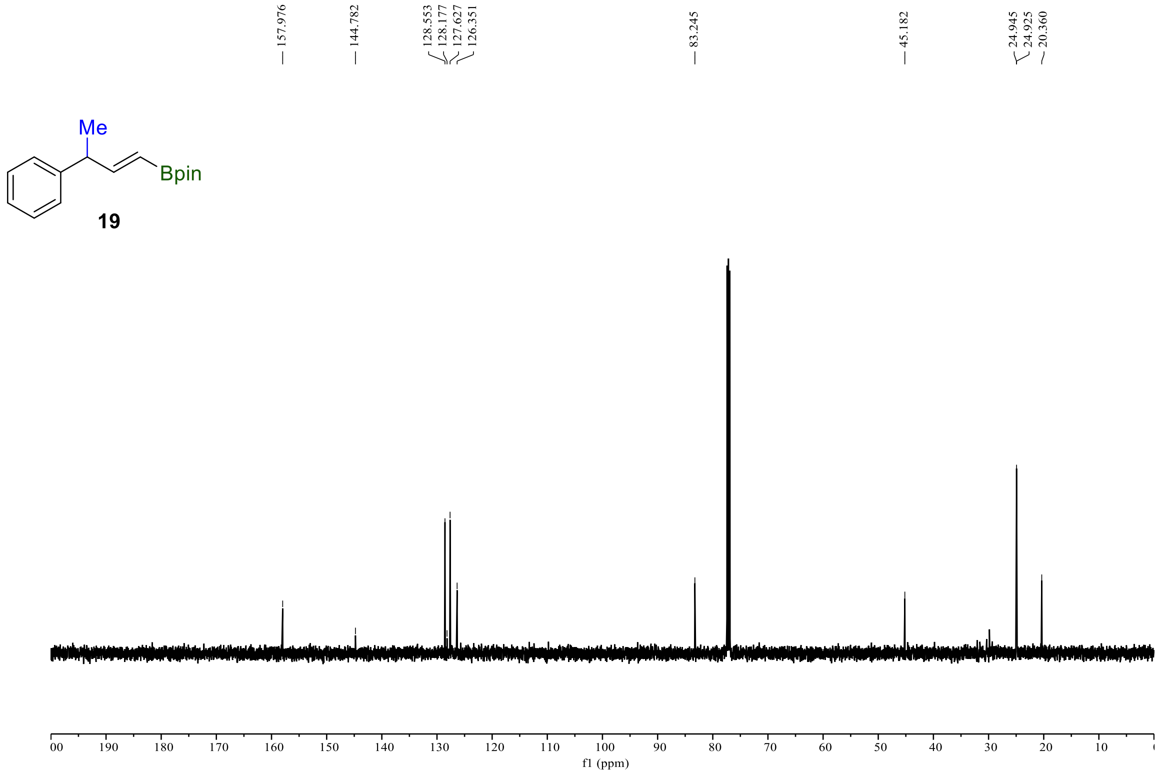

Supplementary Figure 131. ^{13}C{^{1}H} NMR spectrum of compound **19**.

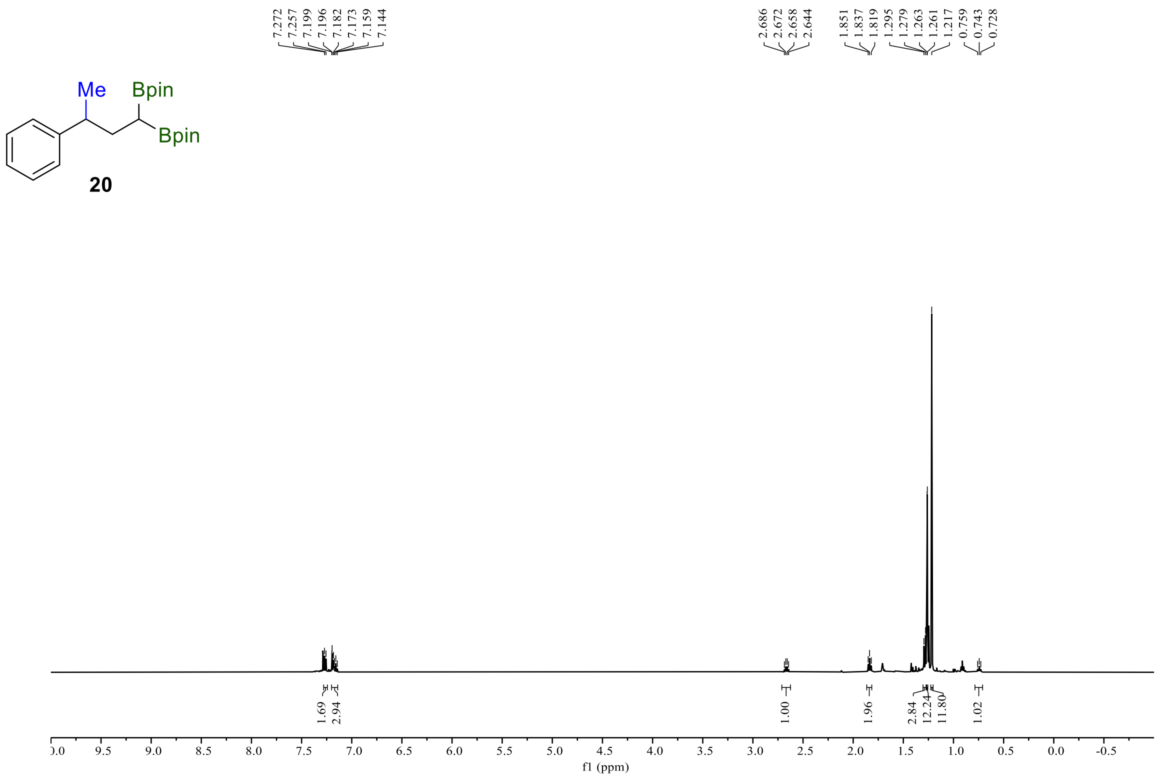

Supplementary Figure 132. ^{1}H NMR spectrum of compound **20**.

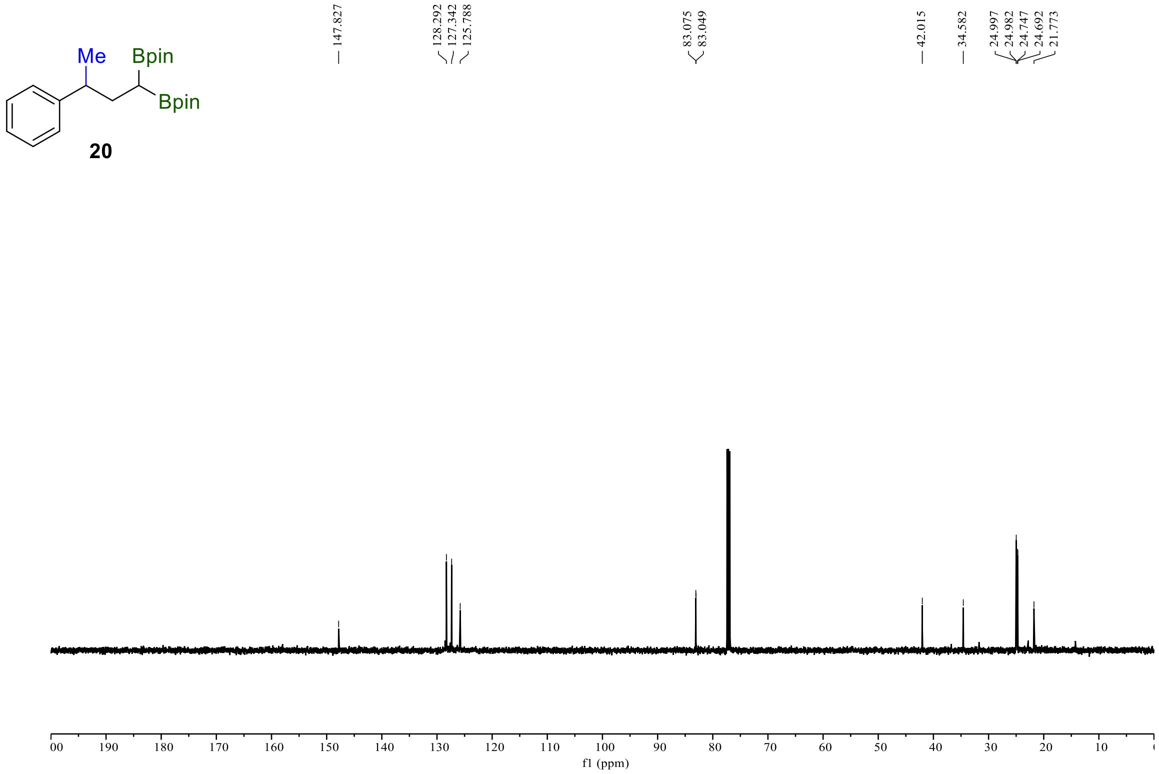

Supplementary Figure 133. $^{13}C\{^{1}H\}$ NMR spectrum of compound **20**.

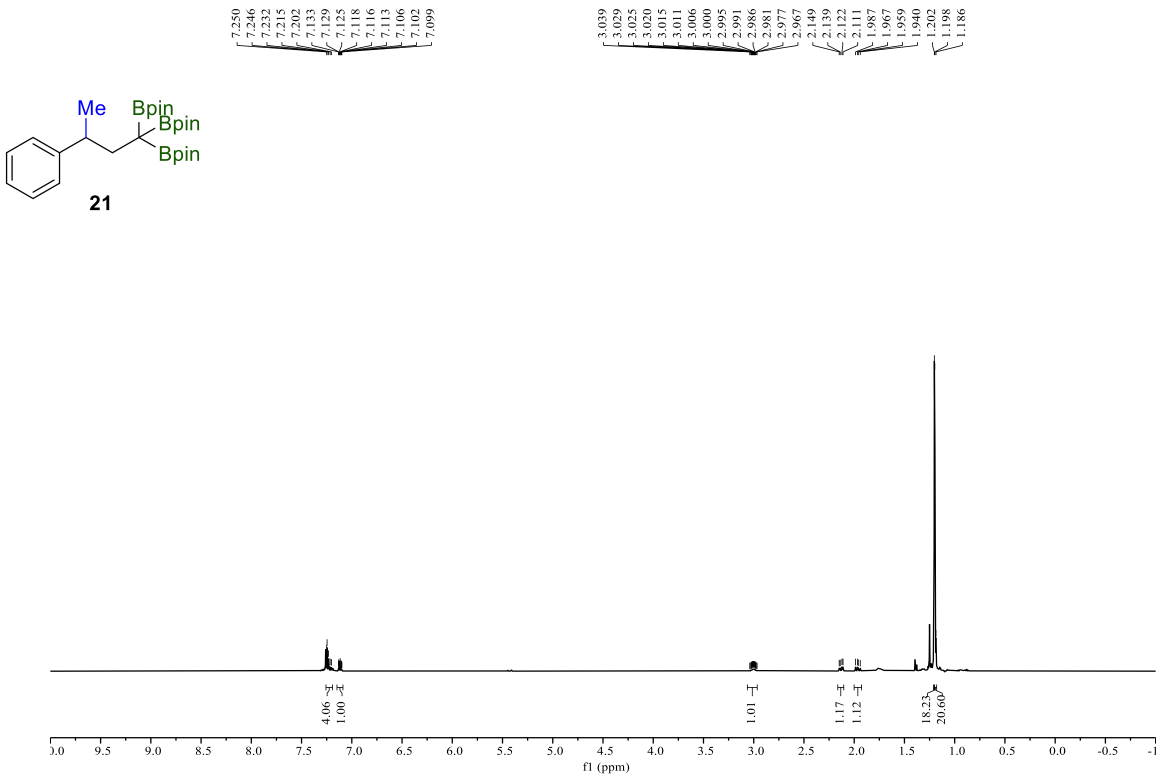

Supplementary Figure 134. ^{1}H NMR spectrum of compound **21**.

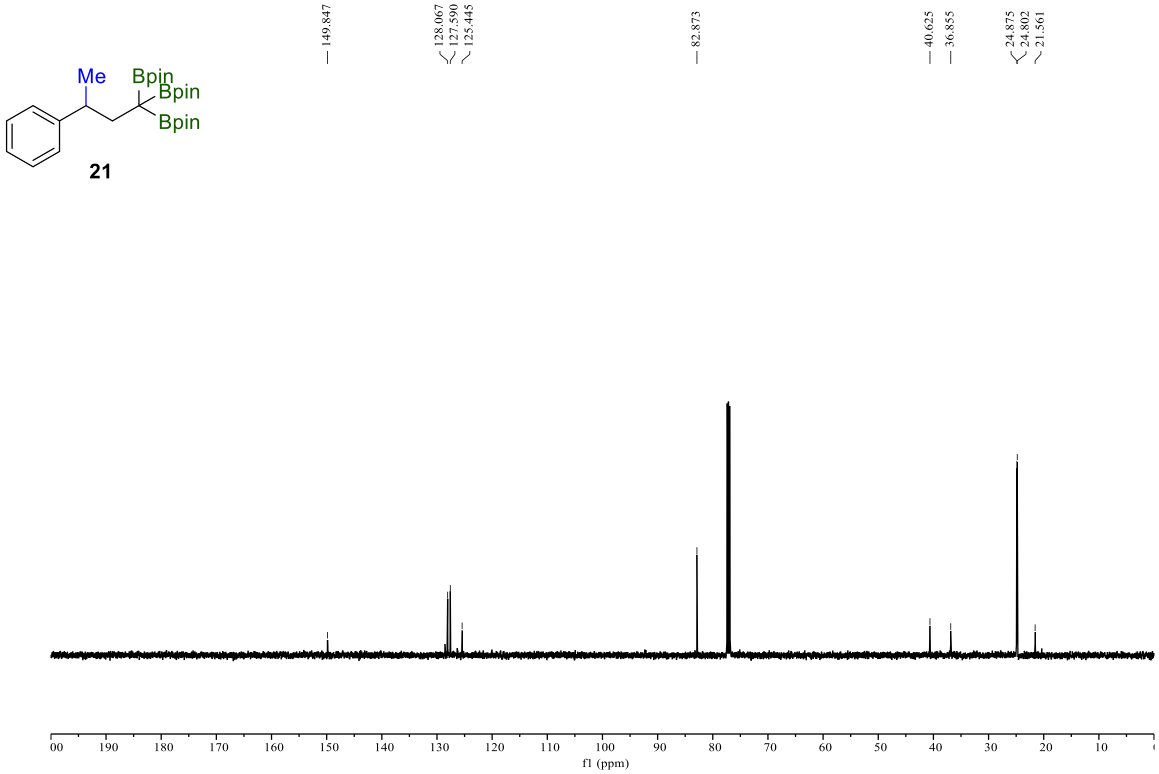

Supplementary Figure 135. $^{13}C\{^{1}H\}$ NMR spectrum of compound **21**.

Supplementary Reference:

1 Kelly, C.B., Ovian, J. M., Cywar, R. M., Gosselin, T. R., Wiles, R. J. & Leadbeater, N. E. Oxidative cleavage of allyl ethers by an oxoammonium salt. *Org. Biomol. Chem.* **13**, 4255-4259 (2015).

2 Sun, X., Frimpong, K. & Tan, K. L. Synthesis of quaternary carbon centers via hydroformylation. *J. Am. Chem. Soc.* **132**, 11841-11843 (2010).

3 Liu, X., Deaton, M., Haeffner, F. & Morken, J. P. A boron alkylidene–alkene cycloaddition reaction: application to the synthesis of aphanamal. *Angew. Chem. Int. Ed.* **56**, 11485-11489 (2017).

4 Coombs, J. R., Zhang, L. & Morken, J. P. Enantiomerically Enriched Tris(boronates): Readily Accessible Conjunctive Reagents for Asymmetric Synthesis. *J. Am. Chem. Soc.* **136**, 16140-16143 (2014).

5 Romano, C. & Mazet, C. Multicatalytic stereoselective synthesis of highly substituted alkenes by sequential isomerization/cross-coupling reactions. *J. Am. Chem. Soc.* **140**, 4743-4750 (2018).

6 Correia, R. & DeShong, P. Palladium-catalyzed arylation of allylic benzoates using hypervalent siloxane derivatives. *J. Org. Chem.* **66**, 7159-7165 (2001).

7 Spoehrle, S. S. M., West, T. H., Taylor, J. E., Slawin, A. M. Z. & Smith, A. D. Tandem palladium and isothiourea relay catalysis: enantioselective synthesis of α-amino acid derivatives via allylic amination and [2,3]-sigmatropic rearrangement. *J. Am. Chem. Soc.* **139**, 11895-11902 (2017).

8 Benoit, G. & Charette, A. B. Diastereoselective borocyclopropanation of allylic ethers using a boromethylzinc carbenoid. *J. Am. Chem. Soc.* **139***,* 1364-1367 (2017).

9 Murray, S. A., Liang, M. Z. & Meek, S. J. Stereoselective tandem bis-electrophile couplings of diborylmethane. *J. Am. Chem. Soc.* **139**, 14061-14064 (2017).

10 Odachowski, M., Bonet, A., Essafi, S., Conti-Ramsden, P., Harvey, J. N., Leonori, D. & Aggarwal, V. K. Development of enantiospecific coupling of secondary and tertiary boronic esters with aromatic compounds. *J. Am. Chem. Soc.* **139***,* 9521-9532 (2016).

11 Mlynarski, S. N., Schuster, C. H. & Morken, J. P. Asymmetric synthesis from terminal alkenes by cascades of diboration and cross-coupling. *Nature* **505**, 386-390 (2014).

12 Ebe, Y., Onoda, M., Nishimura, T. & Yorimitsu, H. Iridium-catalyzed regio- and enantioselective hydroarylation of alkenyl ethers by olefin isomerization. *Angew. Chem. Int. Ed.* **56**, 5607-5611 (2017).

13 Yu, S., Wu, C. & Ge, S. Cobalt-catalyzed asymmetric hydroboration/cyclization of 1, 6-enynes with pinacolborane. *J. Am. Chem. Soc.* **139**, 6526-6529 (2017).

14 Fandrick, K. R., Fandrick, D. R., Gao, J. J., Reeves, J. T., Tan, Z., Li, W., Song, J. J., Lu, B., Yee, N. K. & Senanayake, C. H. Mild and general zinc-alkoxide-catalyzed allylations of ketones with allyl pinacol boronates. *Org. Lett.* **12**, 3748-3751 (2010).

15 Henrion, G. T., Chavas, E. J., Le Goff, X. & Gagosz, F. Biarylphosphonite gold (I) complexes as superior catalysts for oxidative cyclization of propynyl arenes into indan-2-ones. *Angew. Chem. Int. Ed.* **52**, 6277-6282 (2013).

16 Kontokosta, D., Mueller, D. S., Wang, H.-Y. & Anderson, L. L. Preparation of α-imino aldehydes by [1,3]-rearrangements of O-alkenyl oximes. *Org. Lett.* **15**, 4830-4833 (2013).

17 Kumar, N., Reddy, R. R. & Masarwa, A. Stereoselective desymmetrization of gem-diborylalkanes by “trifluorination”. *Chem. Eur. J.* **25**, 8008-8012 (2019).

18 Cao, X., Wang, W., Lu, K., Yao, W., Xue, F. & Ma, M. Magnesium-catalyzed hydroboration of organic carbonates, carbon dioxide and esters. *Dalton Trans.,* **49***,* 2776-2780 (2020).
